# Alantolactone Activates the Extracellular Signal-Regulated Kinase Signaling Pathway to Promote Tumor Necrosis Factor Receptor 1 Ectodomain Shedding

**DOI:** 10.3390/molecules31183296

**Published:** 2026-09-17

**Authors:** Piimwara Yarangsee, Quy Van Vu, Yasunobu Miyake, Takao Kataoka

**Affiliations:** 1 Department of Applied Biology, Kyoto Institute of Technology, Kyoto 606-8585, Japan; d4841502@edu.kit.ac.jp (P.Y.);; 2 Division of Molecular and Cellular Immunoscience, Department of Biomolecular Sciences, Faculty of Medicine, Saga University, Saga 849-8501, Japan; 3 Center for Social and Biomedical Engineering, Kyoto Institute of Technology, Kyoto 606-8585, Japan

**Keywords:** alantolactone, TNF receptor 1, ectodomain shedding, extracellular signal-regulated kinase, RAF1, metalloproteinase, sesquiterpene

## Abstract

Alantolactone is a sesquiterpene lactone that possesses anticancer and anti-inflammatory properties. We previously demonstrated that several sesquiterpenes, including alantolactone, induced the ectodomain shedding of tumor necrosis factor receptor 1 (TNF-R1). In the present study, we investigated the upstream signaling pathway underlying alantolactone-induced TNF-R1 ectodomain shedding. Alantolactone down-regulated the expression of full-length TNF-R1 on the cell surface of human lung adenocarcinoma A549 cells, and this was accompanied by an increase in soluble TNF-R1 in the culture medium. TNF-R1 ectodomain shedding was also detected in human embryonic kidney 293T cells and human fibrosarcoma HT-1080 cells, indicating the conservation of this effect in multiple cell lines. The metalloproteinase inhibitor GM6001 markedly suppressed alantolactone-induced soluble TNF-R1 release and restored cell-surface TNF-R1 expression. Among specific inhibitors targeting mitogen-activated protein kinase (MAPK) signaling pathways, TNF-R1 ectodomain shedding was markedly suppressed by the MAPK/extracellular signal-regulated kinase (ERK) kinase (MEK) inhibitor U0126, whereas the suppressive effects of the c-Jun *N*-terminal kinase (JNK) inhibitor SP600125 or the p38 MAPK inhibitor SB203580 were negligible. Consistent with these results, alantolactone increased phospho-ERK and phospho-RAF1 levels within 60–120 min, while p38 MAPK and JNK were minimally phosphorylated during the 120-min incubation. Collectively, these results indicate that alantolactone-induced TNF-R1 ectodomain shedding is mediated by the activation of the RAF1–ERK signaling pathway.

## 1. Introduction

Inflammation is a fundamental biological response of the immune system that serves as a defense mechanism against harmful stimuli, including irritants, damaged cells, and pathogens [1,2]. Acute inflammation plays an essential role in tissue repair and healing, whereas chronic inflammation often causes tissue damage and contributes to the progression of diverse diseases [3,4]. One of the primary inflammatory mediators is tumor necrosis factor-α (TNF-α), which is mainly produced by macrophages and monocytes, but also by other cell types [5,6,7]. TNF-α exerts pleiotropic effects by interacting with two distinct receptors: TNF receptor 1 (TNF-R1), which is broadly expressed in various tissues and plays a critical role in TNF-α-mediated inflammatory responses, and TNF receptor 2, which is restricted to specific cell types, including immune and endothelial cells [8,9,10,11]. Upon TNF-α stimulation, TNF-R1 initiates multiple cellular signaling pathways, including the nuclear factor κB (NF-κB) and mitogen-activated protein kinase (MAPK) pathways [12,13]. The activation of these pathways leads to the nuclear translocation and activation of transcription factors, including NF-κB and activator protein-1 (AP-1), thereby promoting inflammatory responses and cell survival [8,10].

MAPK signaling pathways are major downstream effectors of pro-inflammatory cytokine receptors and play vital roles in inflammatory responses [14,15]. The activation of pro-inflammatory cytokine receptors triggers several MAPK cascades, including extracellular signal-regulated kinase (ERK), p38 MAPK, and c-Jun *N*-terminal kinase (JNK) [16,17]. The ERK pathway is initiated by the phosphorylation of rapidly accelerated fibrosarcoma (RAF) kinases, including RAF1, followed by the sequential activation of MAPK/ERK kinase (MEK) 1/2 and ERK1/2 [18,19,20]. This cascade regulates downstream transcription factors, including AP-1, and, in turn, pro-inflammatory gene expression [15,17,18]. In addition to transcriptional regulation, MAPK signaling modulates protease activation and receptor processing at the plasma membrane [21,22,23]. Through these mechanisms, MAPK pathways regulate cell-surface receptor availability and responsiveness [24,25,26,27]. Collectively, these studies suggest that MAPK signaling affects inflammatory responses not only through transcriptional regulation, but also through the post-translational processing of cell-surface receptors.

Ectodomain shedding represents an important post-translational mechanism that regulates the abundance and function of many receptors at the cell membrane [28]. This process reduces cell-surface TNF-R1 expression and generates soluble TNF-R1, thereby contributing to the fine-tuning of TNF-α signaling [29]. TNF-R1 shedding is primarily mediated by metalloproteinases, including members of the a disintegrin and metalloproteinase (ADAM) family, which are responsible for the proteolytic release of numerous membrane-bound substrates [29,30,31]. MAPK signaling pathways have been implicated in the regulation of ADAM activity and substrate shedding, linking intracellular signaling to the membrane-proximal regulation of inflammatory responses [21,22,23]. Consistent with this concept, we previously demonstrated that ribotoxic stress activated ERK- and p38 MAPK-dependent pathways, resulting in TNF-R1 ectodomain shedding and the subsequent attenuation of TNF-α-induced NF-κB signaling [32,33,34,35,36,37].

**Figure 1:**
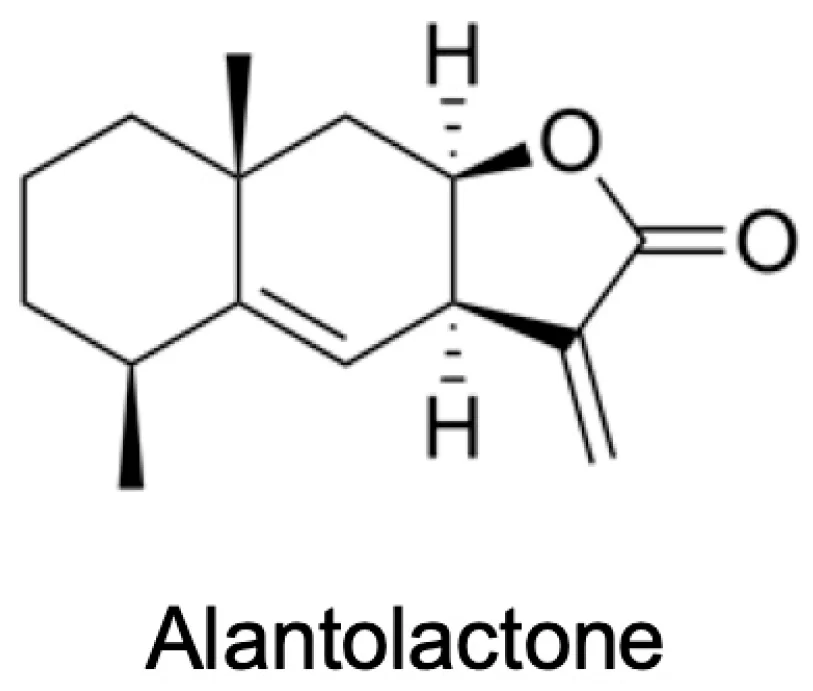
Structure of alantolactone.

Alantolactone (Figure 1) is a naturally occurring sesquiterpene lactone isolated from the medicinal plant *Inula helenium* L. (Asteraceae) [38,39]. Together with its structural isomer isoalantolactone, alantolactone forms a naturally occurring mixture known as helenin [40]. Alantolactone contains an α-methylene-γ-lactone group and exhibits various biological activities, including anti-inflammatory and anticancer effects [39,41,42,43]. It has been shown to suppress TNF-α-dependent NF-κB signaling in human leukemia cells and inhibit constitutive NF-κB signaling in glioblastoma cells [44,45]. We previously demonstrated that alantolactone and its derivatives inhibited NF-κB activation in human lung adenocarcinoma A549 cells upon TNF-α stimulation [46]. We also showed that sesquiterpene lactones bearing an α-methylene-γ-lactone group, including alantolactone, selectively reduced TNF-R1 expression by promoting ectodomain shedding [47]. However, the intracellular signaling mechanisms responsible for alantolactone-induced TNF-R1 shedding remain unclear. Therefore, we herein investigated the upstream signaling pathway involved in alantolactone-induced TNF-R1 ectodomain shedding and demonstrated that the RAF1–ERK signaling pathway plays a role in this process.

## 2. Results

### 2.1. Alantolactone Reduced Cell-Surface TNF-R1 Expression in A549 Cells

**Figure 2:**
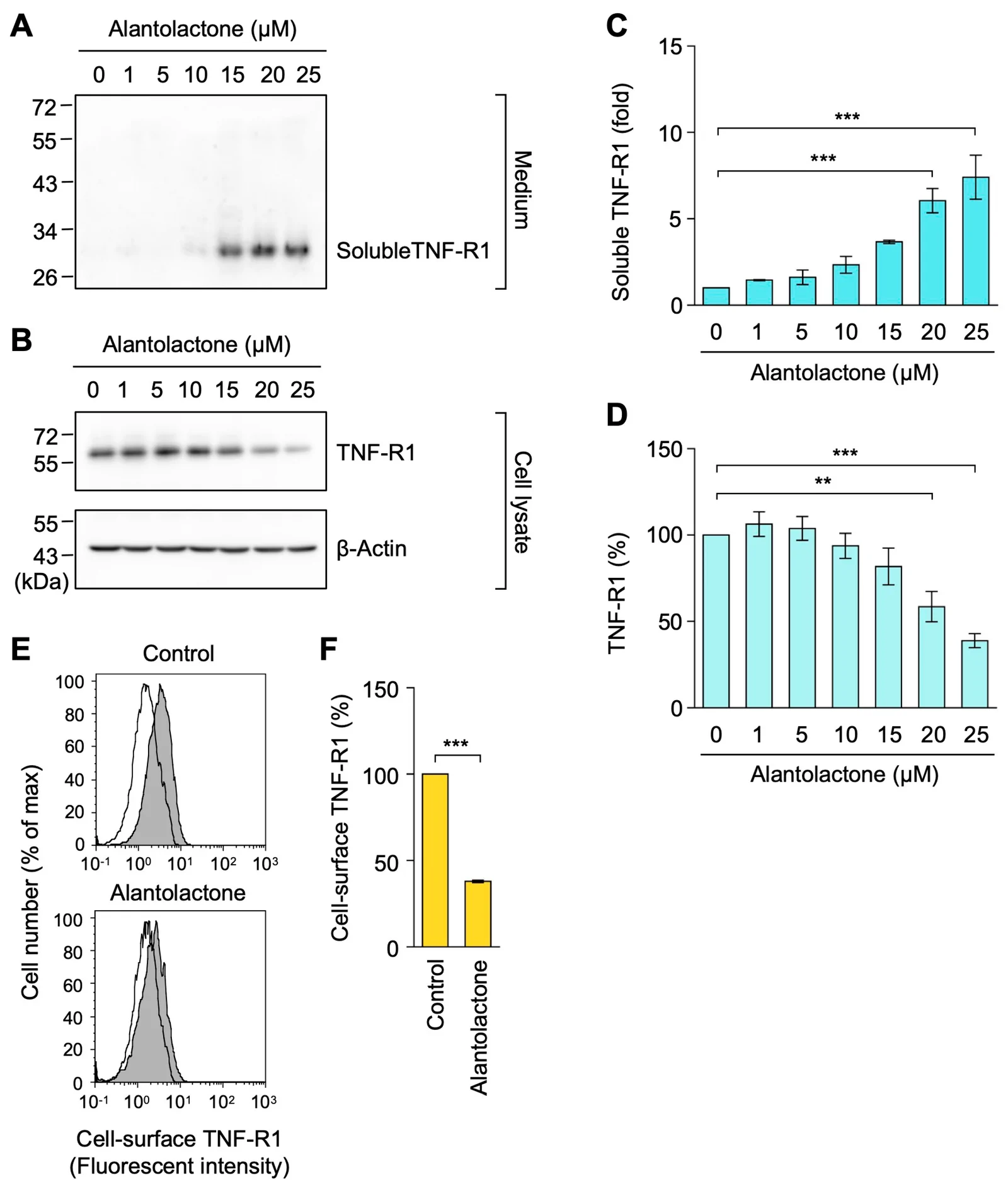
Alantolactone promoted TNF-R1 ectodomain shedding in A549 cells. (**A**–**D**) A549 cells were incubated with or without alantolactone (1–25 µM) for 1 h. Protein levels in the culture medium and cell lysate were assessed by Western blotting. Panels (**A**,**B**) show blot images representative of three and five independent experiments, respectively. Panels (**C**,**D**) show the quantification of soluble TNF-R1 (fold) and total TNF-R1 (%), respectively. Data are presented as the mean ± SEM (*n* = 3 for (**C**) and *n* = 5 for (**D**)). (**E**,**F**) A549 cells were incubated with (+) or without (−) alantolactone at 25 µM for 1 h, and cell-surface TNF-R1 expression was analyzed by flow cytometry using an anti-TNF-R1 antibody (gray area) or isotype control antibody (white area). Panel (**E**) shows representative histograms from three independent experiments. Panel (**F**) shows the quantification of cell-surface TNF-R1 (%). Data are expressed as the mean ± SEM (*n* = 3). ** *p* < 0.01 and *** *p* < 0.001. Original blots are shown in Figures S1–S10.

We previously showed that the treatment of A549 cells with alantolactone for 1 h dose-dependently increased the amount of soluble TNF-R1 in the culture medium, and this was accompanied by a reduction in full-length TNF-R1 in the cell lysate [47]. In the present study, we confirmed that alantolactone increased soluble TNF-R1 levels at concentrations of 20 µM or higher (Figure 2A,C) and concomitantly decreased full-length TNF-R1 levels at similar concentrations (Figure 2B,D). To further examine cell-surface TNF-R1 expression, A549 cells were treated with alantolactone for 1 h and then analyzed by flow cytometry. Alantolactone reduced cell-surface TNF-R1 expression (Figure 2E,F). These results suggest that alantolactone promoted TNF-R1 ectodomain shedding, thereby reducing cell-surface TNF-R1 and increasing soluble TNF-R1.

### 2.2. A Broad-Spectrum Metalloproteinase Inhibitor Decreased Soluble TNF-R1 Levels in Alantolactone-Treated A549 Cells

**Figure 3:**
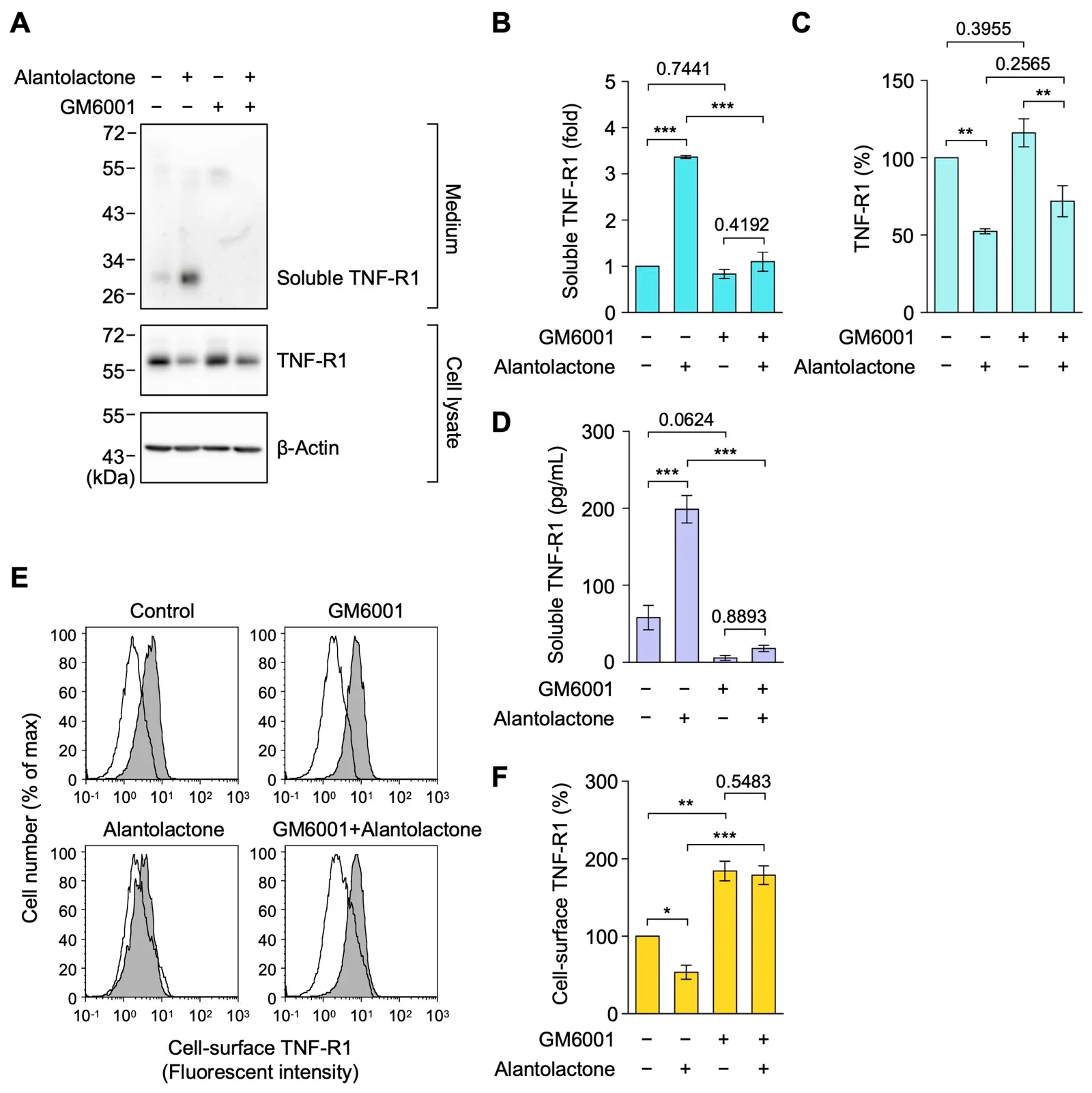
GM6001 inhibited alantolactone-induced TNF-R1 ectodomain shedding in A549 cells. (**A**–**F**) A549 cells were treated with (+) or without (−) GM6001 at 25 µM for 1 h, followed by a treatment with (+) or without (−) alantolactone at 25 µM for 1 h. Protein levels in the culture medium and cell lysate were assessed by Western blotting. Panel (**A**) shows blot images representative of three independent experiments. Panels (**B**,**C**) show the quantification of soluble TNF-R1 (fold) and total TNF-R1 (%). Data are presented as the mean ± SEM (*n* = 3). Panel (**D**) shows the quantification of soluble TNF-R1 (pg/mL) in the culture medium as measured by ELISA. Data are presented as the mean ± SEM (*n* = 3). Cell-surface TNF-R1 expression was analyzed by flow cytometry using an anti-TNF-R1 antibody (gray area) or isotype control antibody (white area) (**E**). Panel (**E**) shows representative histograms from three independent experiments. Panel (**F**) shows the quantification of cell-surface TNF-R1 (%). Data are presented as the mean ± SEM (*n* = 3). * *p* < 0.05, ** *p* < 0.01 and *** *p* < 0.001. Original blots are shown in Figures S11–S18.

In addition to ADAM17 [29,30,31], previous studies have implicated ADAM8 and ADAM10 in TNF-R1 cleavage [48,49,50,51]. To establish whether alantolactone-induced TNF-R1 ectodomain shedding is dependent on metalloproteinase activity, we examined the effects of the broad-spectrum metalloproteinase inhibitor GM6001, also known as ilomastat, which suppresses a broad range of matrix metalloproteinases [52]. It also inhibits several ADAM proteases, including ADAM10 and ADAM17 [53]. A549 cells were treated with or without GM6001 for 1 h, followed by a treatment with alantolactone for an additional 1 h. Alantolactone augmented soluble TNF-R1 levels in the medium (Figure 3A,B; *p* < 0.0001), and GM6001 markedly attenuated this increase (Figure 3B; *p* < 0.0001). In contrast, alantolactone decreased TNF-R1 levels in the cell lysate (Figure 3A,C; *p* = 0.005), while GM6001 did not markedly affect this reduction (Figure 3C; *p* = 0.2565). An enzyme-linked immunosorbent assay (ELISA) confirmed both the alantolactone-induced increase in soluble TNF-R1 (Figure 3D; *p* = 0.0002) and its suppression by GM6001 (Figure 3D; *p* < 0.0001). A flow cytometric analysis further demonstrated that GM6001 attenuated the alantolactone-induced reduction in cell-surface TNF-R1 expression (Figure 3E,F; *p* = 0.0411). GM6001 also increased basal cell-surface TNF-R1 levels in the absence of alantolactone (Figure 3F; *p* = 0.0014), but did not significantly affect soluble TNF-R1 levels (Figure 3B; *p* = 0.7441) or TNF-R1 levels in cell lysates (Figure 3C; *p* = 0.3955), as assessed by Western blotting. In ELISA, GM6001 slightly reduced basal soluble TNF-R1 levels (Figure 3D; *p* = 0.0624). These results show that metalloproteinases may contribute to constitutive TNF-R1 ectodomain shedding and clearly support their involvement in alantolactone-induced TNF-R1 ectodomain shedding.

### 2.3. Alantolactone Promoted TNF-R1 Ectodomain Shedding in 293T Cells and HT-1080 Cells

**Figure 4:**
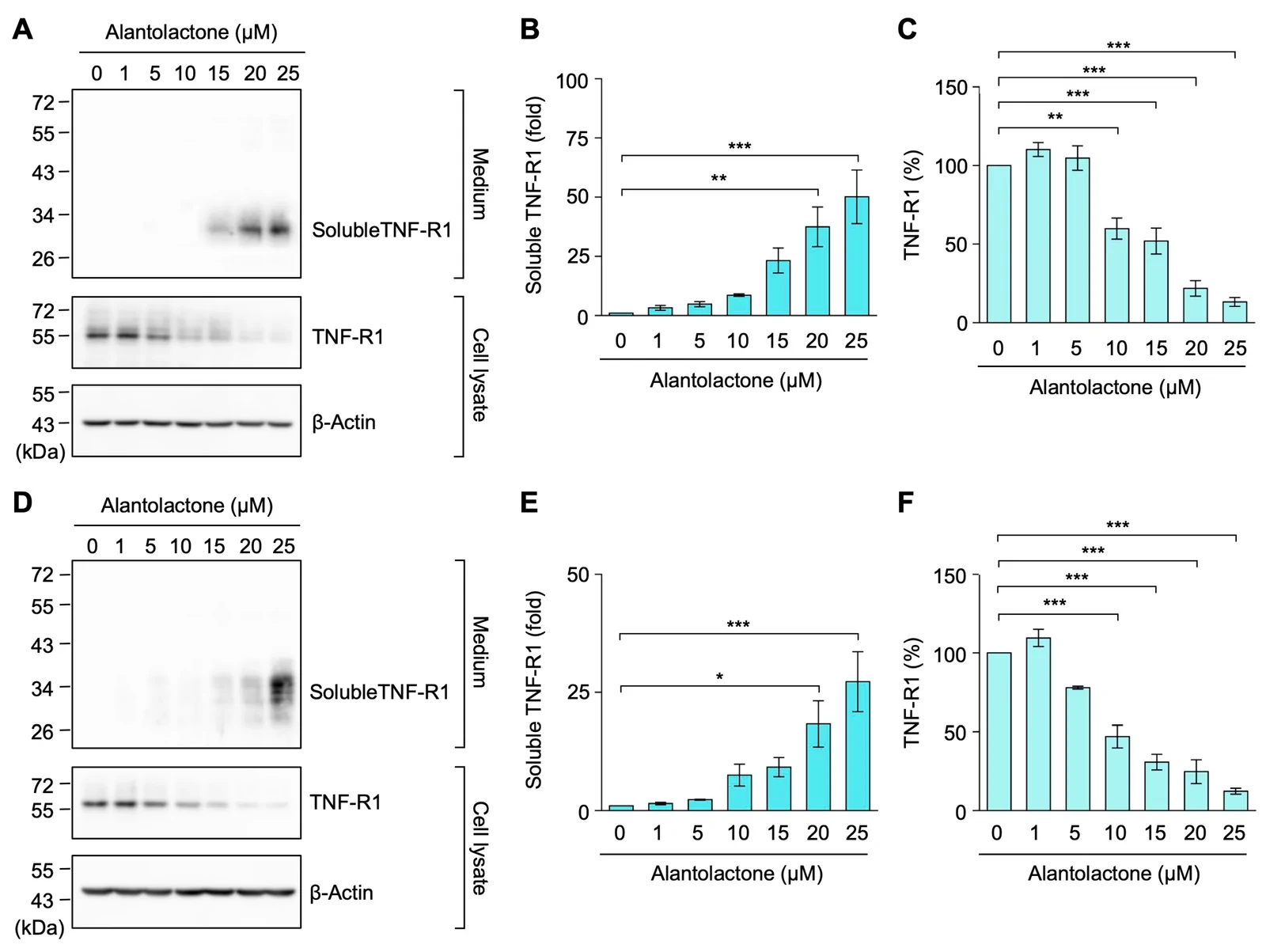
Alantolactone promoted TNF-R1 ectodomain shedding in 293T and HT-1080 cells. (**A**–**F**) 293T cells (**A**–**C**) and HT-1080 cells (**D**–**F**) were incubated with alantolactone at the indicated concentrations for 1 h. Protein levels in culture media and cell lysates were assessed by Western blotting. Panels (**A**,**D**) show blot images representative of three independent experiments. Panels (**B**,**C**,**E**,**F**) show the quantification of soluble TNF-R1 (fold) and total TNF-R1 (%). Data are presented as the mean ± SEM (*n* = 3). * *p* < 0.05, ** *p* < 0.01, and *** *p* < 0.001. Original blots are shown in Figures S19–S34.

To clarify whether alantolactone-induced TNF-R1 ectodomain shedding is restricted to A549 cells, we examined its effects in two additional cell lines: 293T cells and HT-1080 cells. Cells were incubated with the indicated concentrations of alantolactone, after which TNF-R1 levels in culture media and cell lysates were assessed by Western blotting. Alantolactone increased soluble TNF-R1 levels in a dose-dependent manner in both 293T (Figure 4A,B) and HT-1080 cells (Figure 4D,E). In contrast, alantolactone decreased full-length TNF-R1 levels in the cell lysates of both cell lines (Figure 4A,C,D,F). These results suggest that alantolactone-induced TNF-R1 ectodomain shedding is not restricted to A549 cells, but also occurs in multiple cell types.

### 2.4. A MEK Inhibitor Suppressed Alantolactone-Induced TNF-R1 Ectodomain Shedding in A549 Cells

We previously reported that translation inhibitors activated ERK, p38 MAPK, and JNK in A549 cells and that under these experimental conditions, ERK and p38 MAPK, but not JNK, contributed to TNF-R1 ectodomain shedding [32,33,34,35,36,37]. The inhibition of either the ERK or p38 MAPK pathway was not sufficient to inhibit TNF-R1 shedding [33,34,35]. To examine the involvement of MAPK signaling pathways in alantolactone-induced TNF-R1 ectodomain shedding, we evaluated the effects of specific inhibitors targeting the MAPK pathways. A549 cells were preincubated with the MEK inhibitor U0126, the p38 MAPK inhibitor SB203580, or the JNK inhibitor SP600125 for 1 h before being treated with alantolactone for 1 h. To simultaneously block both the ERK and p38 MAPK pathways, we also examined the combination of U0126 and SB203580.

**Figure 5:**
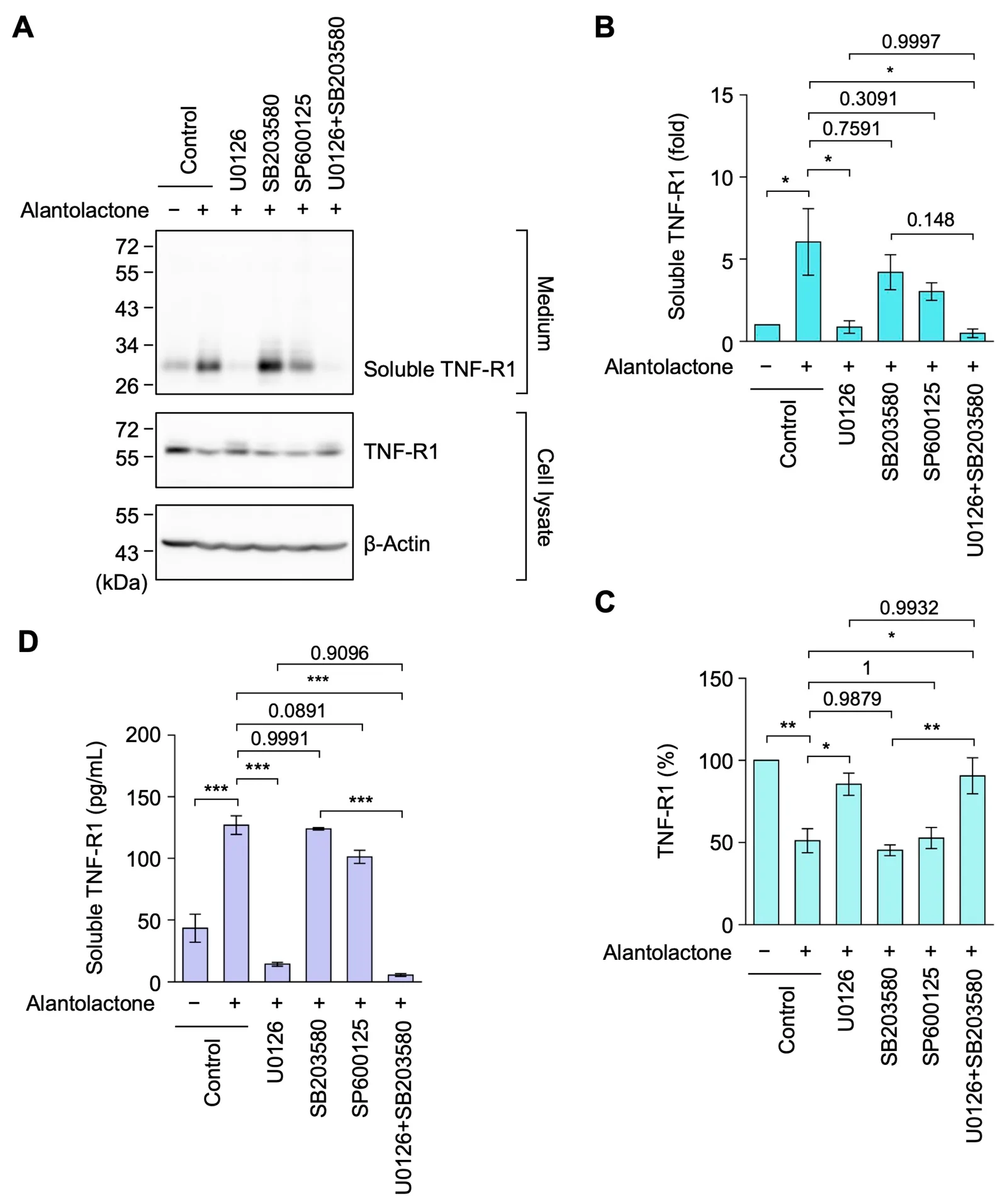
U0126 inhibited alantolactone-induced TNF-R1 ectodomain shedding in A549 cells. (**A**–**D**) A549 cells were preincubated with or without U0126, SB203580, SP600125, or the combination of U0126 and SB203580 at 10 µM each for 1 h, followed by a treatment with (+) or without (−) alantolactone at 25 µM for 1 h. Protein levels in the culture medium and cell lysate were evaluated by Western blotting. Panel (**A**) shows blot images representative of three independent experiments. Panels (**B**,**C**) show the quantification of soluble TNF-R1 (fold) and total TNF-R1 (%). Data are presented as the mean ± SEM (*n* = 3). Panel (**D**) shows the quantification of soluble TNF-R1 (pg/mL) in the culture medium as measured by ELISA. Data are presented as the mean ± SEM (*n* = 3). * *p* < 0.05, ** *p* < 0.01, and *** *p* < 0.001. Original blots are shown in Figures S35–S42.

MEK inhibition by U0126 or by the combination of U0126 and SB203580 significantly attenuated the alantolactone-induced increase in soluble TNF-R1 (Figure 5A,B; *p* = 0.0255 and 0.0161, respectively). In contrast, SB203580 or SP600125 alone did not significantly affect the alantolactone-induced increase in soluble TNF-R1 (Figure 5B; *p* = 0.7591 and 0.3091, respectively). Alantolactone decreased total TNF-R1 levels in cell lysates (Figure 5A,C; *p* = 0.0026), and this reduction was reversed by U0126 and the combination of U0126 and SB203580 (Figure 5C; *p* = 0.0326 and 0.0131, respectively), but not by SB203580 or SP600125 (Figure 5C; *p* = 0.9879 and 1, respectively). ELISA further confirmed that alantolactone increased soluble TNF-R1 levels from approximately 40 to 120 pg/mL (Figure 5D; *p* < 0.0001). This increase was markedly suppressed by U0126 alone or in combination with SB203580 (Figure 5D; *p* < 0.0001 and *p* < 0.0001, respectively), whereas SB203580 or SP600125 alone exerted minimal effects (Figure 5D; *p* = 0.9991 and 0.0891, respectively). These results indicate that MEK–ERK signaling was required for alantolactone-induced TNF-R1 ectodomain shedding, while the p38 MAPK and JNK pathways did not appear to make major contributions under these experimental conditions.

### 2.5. Alantolactone Induced the Phosphorylation of ERK, but Exerted Minimal Effects on the Phosphorylation of p38 MAPK or JNK in A549 Cells

**Figure 6:**
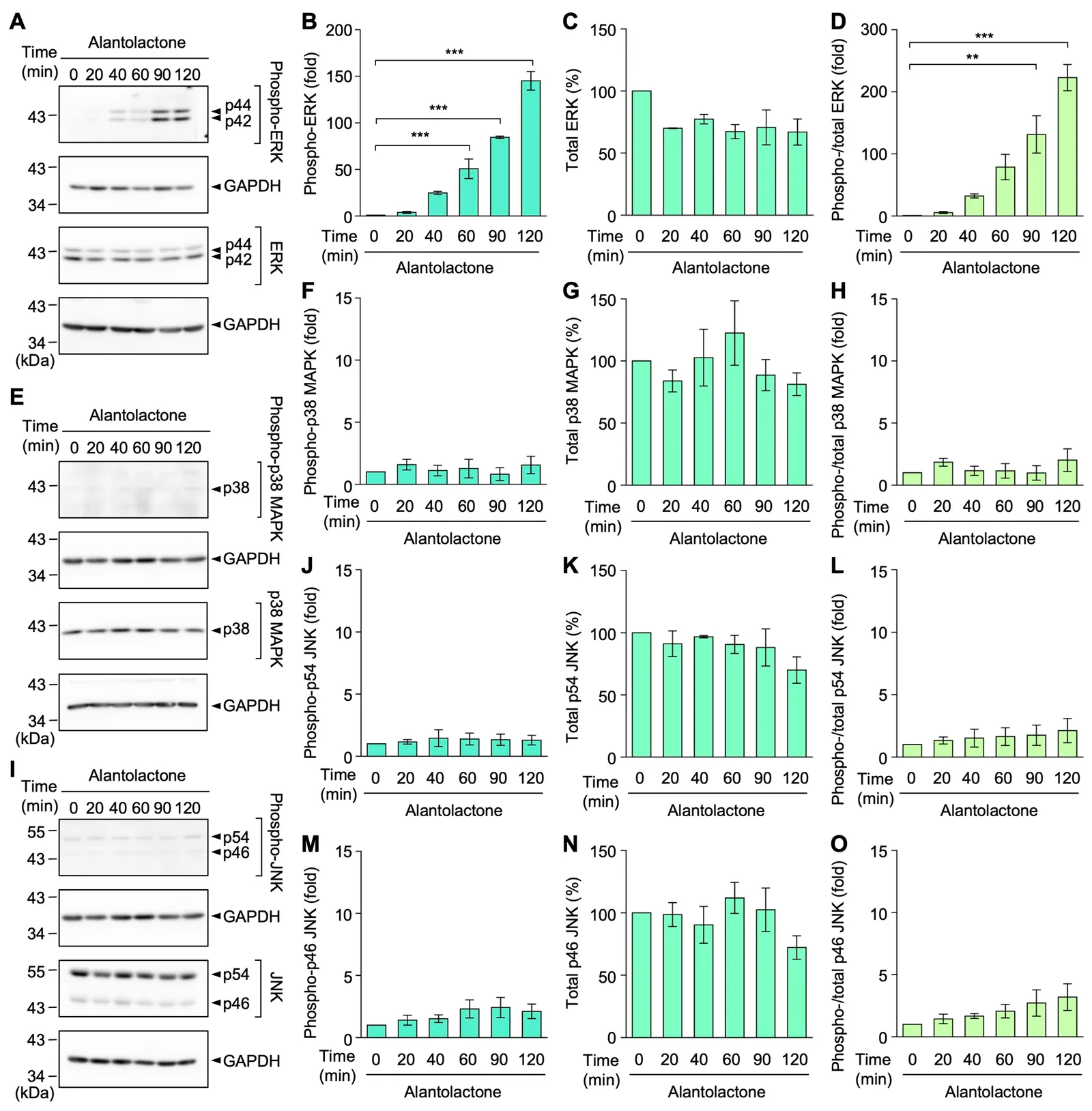
Alantolactone induced ERK phosphorylation in A549 cells. (**A**–**O**) A549 cells were incubated with alantolactone at 25 µM for 0, 20, 40, 60, 90, and 120 min. Protein levels in cell lysates were assessed by Western blotting. Panels (**A**,**E**,**I**) show blot images representative of three independent experiments. Panels (**B**–**D**) show the quantification of phospho-ERK (fold), total ERK (%), and phospho-ERK/total ERK (fold). Panels (**F**–**H**) show the quantification of phospho-p38 MAPK (fold), total p38 MAPK (%), and phospho-p38 MAPK/total p38 MAPK (fold). Panels (**J**–**O**) show the quantification of phospho-p54 JNK (fold), total p54 JNK (%), phospho-p54 JNK/total p54 JNK (fold), phospho-p46 JNK (fold), total p46 JNK (%), and phospho-p46 JNK/total p46 JNK (fold). Data are expressed as the mean ± SEM (*n* = 3). ** *p* < 0.01 and *** *p* < 0.001. Original blots are shown in Figures S43–S66.

To address whether alantolactone activates the ERK, p38 MAPK, or JNK signaling pathways, A549 cells were incubated with alantolactone for the indicated times, and the phosphorylation levels of these MAPKs were analyzed by Western blotting. Alantolactone strongly induced ERK phosphorylation at 60, 90, and 120 min (Figure 6A,B), whereas total ERK protein levels remained unchanged (Figure 6A,C). Accordingly, the ratio of phospho-ERK to total ERK increased 90 and 120 min after the alantolactone treatment (Figure 6D).

In contrast, alantolactone exerted minimal effects on the phosphorylation of p38 MAPK, p54 JNK, or p46 JNK at any of the time points examined between 20 and 120 min (Figure 6E,F,I,J,M). The total protein levels of p38 MAPK, p46 JNK, and p54 JNK also remained unchanged during the 120-min treatment (Figure 6E,G,I,K,N), and the corresponding phospho-to-total protein ratios were minimally affected (Figure 6H,L,O). These results indicate that alantolactone preferentially induced ERK phosphorylation, without strongly activating p38 MAPK or JNK.

### 2.6. Alantolactone Induced RAF1 Phosphorylation in A549 Cells

**Figure 7:**
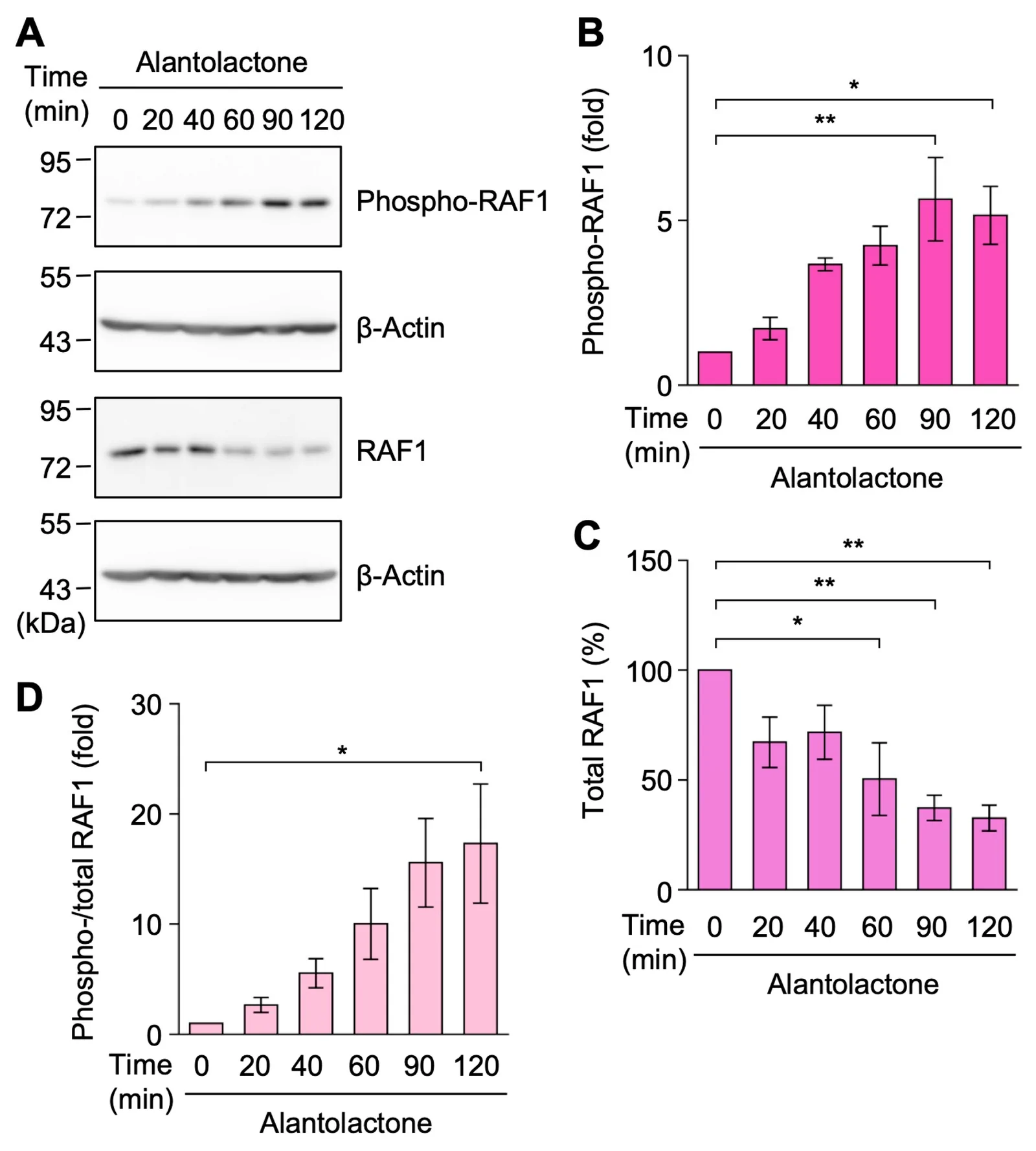
Alantolactone induced RAF1 phosphorylation in A549 cells. (**A**–**D**) A549 cells were incubated with alantolactone at 25 µM for 0, 20, 40, 60, 90, and 120 min. Protein levels in the cell lysate were assessed by Western blotting. Panel (**A**) shows blot images representative of three independent experiments. Panels (**B**–**D**) show the quantification of phospho-RAF1 (fold), total RAF1 (%), and phospho-RAF1/total RAF1 (fold). Data are presented as the mean ± SEM (*n* = 3). * *p* < 0.05 and ** *p* < 0.01. Original blots are shown in Figures S67–S74.

To further elucidate the mechanisms underlying ERK activation, we examined the phosphorylation status of RAF1, an upstream kinase in the ERK signaling pathway. RAF1 is expressed in A549 cells and has been implicated in proliferation, metastasis, and invasion [54,55]. A549 cells were incubated with alantolactone for the indicated times between 20 and 120 min, and Western blotting was used to analyze RAF1 phosphorylation. Alantolactone induced RAF1 phosphorylation in a time-dependent manner, reaching a plateau at 90–120 min (Figure 7A,B). In contrast, total RAF1 protein levels decreased following the alantolactone treatment (Figure 7A,C). Consequently, the ratio of phospho-RAF1 to total RAF1 gradually increased (Figure 7D). Collectively, these results suggest that RAF1 activation is associated with, and may contribute to, alantolactone-induced ERK activation.

## 3. Discussion

**Figure 8:**
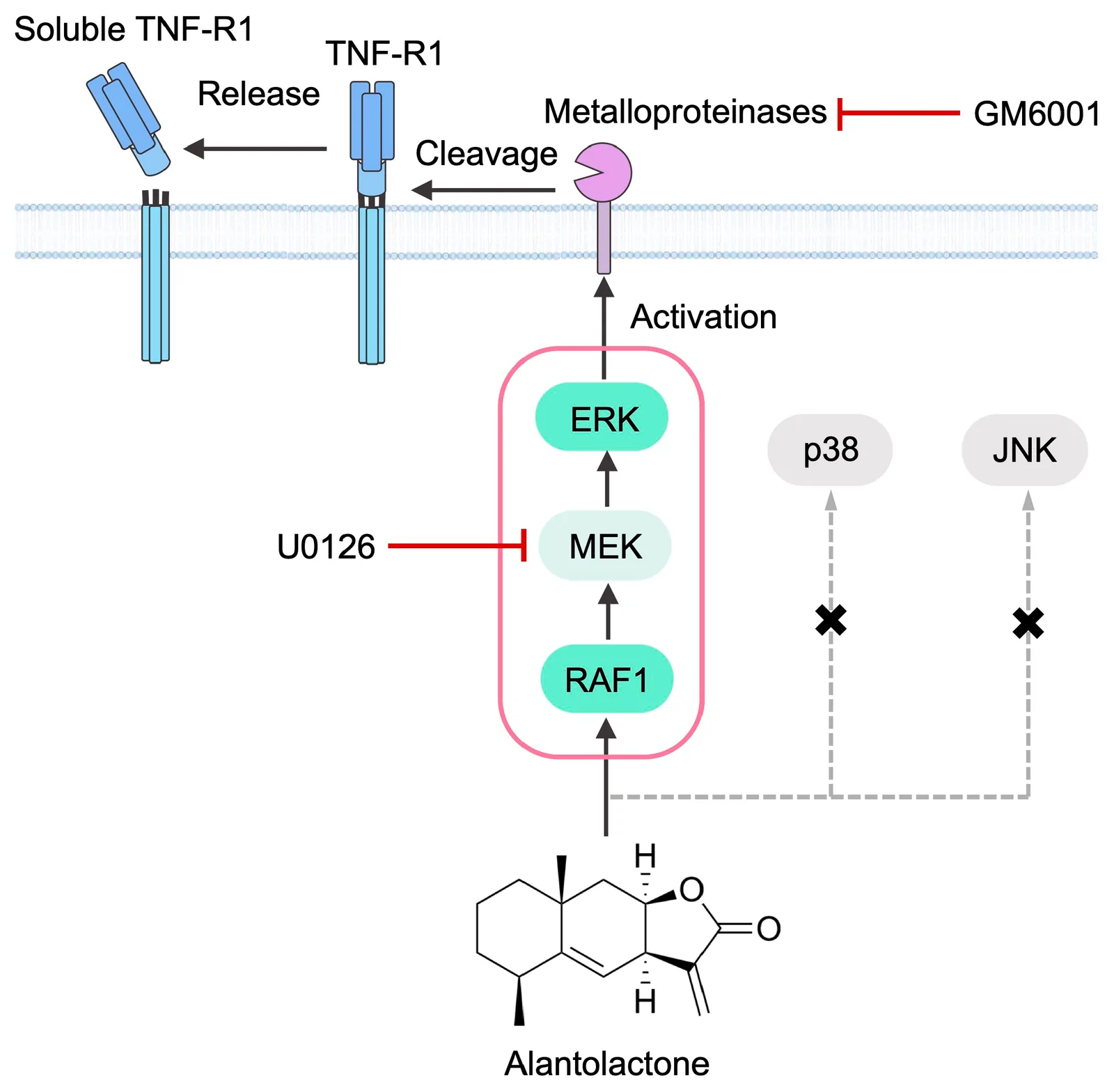
Schematic illustration of the proposed mechanism of alantolactone-induced TNF-R1 ectodomain shedding. Alantolactone preferentially activates the RAF1–ERK signaling pathway (dark gray arrows), while exerting a minimal effect on the JNK and p38 MAPK pathways (light gray dotted arrows). ERK activation is associated with metalloproteinase-dependent TNF-R1 ectodomain shedding, resulting in the release of soluble TNF-R1 into the culture medium and a reduction in cell-surface TNF-R1 expression (dark gray arrows). Alantolactone-induced TNF-R1 shedding is suppressed by the metalloproteinase inhibitor GM6001 and the MEK inhibitor U0126 (red T-bars).

We previously showed that alantolactone selectively reduced TNF-R1 expression without detectable decreases in TNF-R1-associated signaling components, including receptor-interacting kinase 1, TNF receptor-associated factor 2, and TNF receptor-associated death domain protein [46]. The findings of Western blotting further demonstrated that alantolactone increased soluble TNF-R1 levels in the culture medium [47], suggesting the induction of TNF-R1 ectodomain shedding. In the present study, ELISA and flow cytometric analyses provided additional evidence to show that alantolactone promotes TNF-R1 ectodomain shedding. More importantly, we investigated the signaling mechanism underlying this response. The results obtained herein indicate that alantolactone induced RAF1 and ERK phosphorylation and promoted TNF-R1 ectodomain shedding through a MEK-dependent, metalloproteinase-mediated mechanism, whereas p38 MAPK and JNK appeared to be dispensable under the present experimental conditions (Figure 8).

GM6001 is a broad-spectrum metalloproteinase inhibitor that is widely used to suppress many matrix metalloproteinases and several ADAM proteases [52,53]. ADAM8, ADAM10, and ADAM17 have been reported to mediate TNF-R1 ectodomain shedding, and ADAM17 appears to play a major role in this process in several cell types [29,30,31,48,49,50,51]. Previous studies demonstrated that GM6001 effectively inhibited the stimulated ectodomain shedding of various transmembrane proteins in multiple cell types [56,57,58]. In the present study, alantolactone markedly enhanced the release of soluble TNF-R1 into the culture medium, which is consistent with the proteolytic cleavage of membrane-bound TNF-R1. GM6001 strongly suppressed this effect, supporting the involvement of metalloproteinases in alantolactone-induced TNF-R1 shedding. However, despite its pronounced inhibition of soluble TNF-R1 release, GM6001 did not fully restore cell-associated TNF-R1 levels in alantolactone-treated cells. This result suggests that alantolactone reduces TNF-R1 levels through mechanisms besides metalloproteinase-mediated ectodomain shedding. These mechanisms may involve changes in translation, receptor trafficking, intracellular degradation, or other post-translational regulatory processes. Therefore, metalloproteinase-dependent shedding appears to be a major, but not exclusive, mechanism by which alantolactone modulates intracellular TNF-R1 expression.

Alantolactone-induced TNF-R1 ectodomain shedding was also observed in 293T and HT-1080 cells, indicating that this response is not restricted to A549 cells. Similar increases in soluble TNF-R1 were detected in all three cell lines, suggesting that the effects of alantolactone are reproducible across different cellular backgrounds. Although the apparent magnitude of TNF-R1 shedding differed among the cell lines, the present study was not designed to quantitatively compare shedding efficiency between them. Our objective was to establish whether alantolactone was capable of inducing TNF-R1 ectodomain shedding in multiple cell types. These results are consistent with our previous finding showing that cucurbitacin B promoted the ectodomain shedding of TNF-R1 in both A549 and 293T cells [59], as well as with previous studies demonstrating that regulated TNF-R1 ectodomain shedding may occur in multiple cell types in response to distinct stimuli [60,61,62]. Collectively, these findings and the present results suggest that alantolactone-induced TNF-R1 ectodomain shedding represents a reproducible biological response rather than an A549 cell-specific phenomenon.

In the present study, we did not define individual metalloproteinases involved in alantolactone-induced TNF-R1 ectodomain shedding. However, ADAM10 and ADAM17 are plausible candidate sheddases because both proteases mediate the ectodomain shedding of various cell-surface ligands and receptors and thereby regulate cellular signaling pathways [63,64]. ADAM10 and ADAM17 have been shown to mediate the shedding of several epidermal growth factor receptor (EGFR) ligands [63,64]. ADAM10 also plays a major role in the proteolytic processing of Notch and in HER2 ectodomain shedding [65,66]. Therefore, alantolactone may affect the Notch and EGFR/HER2 signaling pathways. Further studies are needed to establish whether alantolactone affects these signaling pathways.

Alantolactone dose-dependently reduced cell-associated TNF-R1 levels in all three cell lines examined. The apparent 50% inhibitory concentration (IC_50_) values for the reduction in cell-associated TNF-R1 were estimated to be approximately 10 µM in HT-1080 cells, 15 µM in 293T cells, and 22 µM in A549 cells. However, cell-associated TNF-R1 levels may be regulated not only by shedding, but also by other processes, including protein synthesis and degradation. Therefore, these differences in apparent IC_50_ values may not solely reflect differences in metalloproteinase-dependent shedding among the three cell lines. TNF-R1 was previously shown to be cleaved by ADAM17, ADAM10, and ADAM8 [29,30,31,48,49,50,51]. According to the Human Protein Atlas [67], ADAM10 and ADAM17 are expressed in A549 and HT-1080 cells, as well as in 293 cells; however, data are not available on 293T cells, and ADAM8 is expressed at markedly lower levels. Although the relative expression profiles of these metalloproteinases differ among the cell lines, their expression levels alone do not necessarily indicate their contribution to TNF-R1 shedding. In the present study and our previous studies [32,34,35,36,59], we used TNF-α protease inhibitor 2 and GM6001 to inhibit metalloproteinase activity; however, both compounds inhibit multiple ADAM proteases and MMPs [23,52,53,68]. In contrast, KP-457 is a more selective ADAM17 inhibitor, exhibiting 50-fold greater selectivity for ADAM17 than for ADAM10 and several MMPs [69]. Therefore, studies using more selective pharmacological inhibitors, together with the genetic silencing of individual ADAM proteases, will be required to define the specific contribution of each ADAM protease to alantolactone-induced TNF-R1 shedding.

We previously demonstrated that several translation inhibitors triggered the ribotoxic stress response and activated the phosphorylation of ERK, JNK, and p38 MAPK [33,35,37]. Accumulating evidence suggests that ERK and p38 MAPK directly regulate ADAM17 activity, thereby facilitating the ectodomain shedding of multiple membrane-bound receptors [24,25,26,27]. The specific activation of ADAM17 via ERK-mediated phosphorylation has been demonstrated in multiple experimental systems, further linking MAPK signaling to regulated ectodomain shedding [24,25,70,71]. Consistent with these findings, we showed that inhibitors targeting the ERK and p38 MAPK pathways attenuated the ectodomain shedding of TNF-R1 in cells treated with translation inhibitors [33,34,35,37]. In the present study, the MEK inhibitor U0126 markedly suppressed the alantolactone-induced increase in soluble TNF-R1 levels, supporting a critical role for ERK activation in this process. In contrast, the effects of inhibiting p38 MAPK with SB203580 or JNK with SP600125 were negligible. These results indicate that alantolactone-induced TNF-R1 ectodomain shedding differs from ribotoxic stress-induced shedding in its preferential dependence on the MEK-ERK pathway.

Consistent with the inhibitor experiments, alantolactone selectively induced ERK phosphorylation in a time-dependent manner, while the phosphorylation of JNK and p38 MAPK remained largely unchanged. These results are in line with previous findings showing that sesquiterpene lactones, including alantolactone, differentially modulated MAPK phosphorylation depending on the cellular context [39,41,42,43,72,73,74]. Alantolactone simultaneously activated ERK, JNK, and p38 MAPK in human breast cancer MDA-MB-231 cells treated with 15 µM alantolactone for 3–24 h [75] and in human colorectal cancer HCT-116 cells treated with 30 µM alantolactone for 1–6 h [76]. In contrast, alantolactone at 8–10 µM inhibited the phosphorylation of ERK, JNK, and p38 MAPK in human osteosarcoma cells [77]. Alantolactone at 2–4 µM also suppressed ERK phosphorylation in human T-cell lymphoma cells after 48 h of treatment [78]. In the present study, alantolactone at 25 µM selectively activated ERK within 1–2 h. Taken together, these studies suggest that the effects of alantolactone on ERK signaling depend on experimental conditions, including its concentration, treatment duration, and cellular context. Therefore, the extent of alantolactone-induced TNF-R1 ectodomain shedding may also vary depending on these experimental conditions.

In the canonical ERK cascade, RAF family kinases activate MEK, which, in turn, phosphorylates and activates ERK [18,19,20]. Among RAF isoforms, RAF1 is ubiquitously expressed [79] and plays a critical role in transmitting extracellular signals to ERK in lung epithelial and cancer cells [54,55]. Alantolactone markedly enhanced RAF1 phosphorylation, which was accompanied by increased ERK activation, while U0126 suppressed alantolactone-induced TNF-R1 shedding. Although MEK phosphorylation was not directly examined in the present study, these findings collectively support the involvement of a RAF1–MEK–ERK signaling axis in alantolactone-induced TNF-R1 ectodomain shedding. Time-course experiments further showed that alantolactone rapidly induced RAF1 phosphorylation. RAF family proteins are generally activated through RAS-dependent recruitment to the plasma membrane and phosphorylation by protein kinases [19,20]. We did not investigate whether alantolactone promotes the activation of RAS or upstream protein kinases. The rapid induction of RAF1 phosphorylation is in line with the involvement of a membrane-associated or membrane-proximal signaling process. Further experiments are required to identify the primary molecular target of alantolactone that triggers RAF1-MEK-ERK signaling. Overall, the present results provide a mechanistic basis for the alantolactone-induced reduction in cell-surface TNF-R1 and extend our previous finding showing that alantolactone promotes TNF-R1 shedding.

## 4. Materials and Methods

### 4.1. Cell Culture

Human lung adenocarcinoma A549 cells (JCRB0076; JCRB Cell Bank, National Institutes of Biomedical Innovation, Health, and Nutrition, Osaka, Japan), human fibrosarcoma HT-1080 cells (JCRB9113; JCRB Cell Bank), and human embryonic kidney 293T cells (RCB2202; RIKEN BioResource Research Center, Tsukuba, Japan) were used in the present study. Cell culture conditions have been described in our previous studies [80]. A549 cells and HT-1080 cells were maintained in RPMI 1640 medium (Thermo Fisher Scientific, Waltham, MA, USA), and 293T cells in DMEM (Thermo Fisher Scientific). Both media were supplemented with 0.9% Penicillin-Streptomycin Mixed Solution (Stabilized) (Nacalai Tesque, Kyoto, Japan) and 8.2% heat-inactivated fetal bovine serum (FBS) (Nichirei Bioscience, Tokyo, Japan, and Sigma-Aldrich, St. Louis, MO, USA). Cells were subcultured every 2–3 days. Serum-free medium was used in experiments in which soluble TNF-R1 was measured and in analyses of the phosphorylated and total forms of ERK, JNK, p38 MAPK, and RAF1 in cell lysates.

### 4.2. Reagents

The reagents used in the present study included alantolactone (S8318; Selleck Chemicals, Houston, TX, USA), GM6001 (T2743; TargetMol, Boston, MA, USA), U0126 (211-01051; Wako Pure Chemical Industries, Osaka, Japan), SB203580 (13067; Cayman Chemical, Ann Arbor, MI, USA), and SP600125 (197-16591; Fujifilm Wako Pure Chemical Corporation, Osaka, Japan).

### 4.3. Antibodies

The following antibodies were used: TNF-R1 (H-5; Santa Cruz Biotechnology, Dallas, TX, USA), ERK (137F5; Cell Signaling Technology, Danvers, MA, USA), JNK (#9252; Cell Signaling Technology), p38 MAPK (#9212; Cell Signaling Technology), RAF1 (D5X6R; Cell Signaling Technology), phospho-ERK (Thr202/Tyr204) (#9101; Cell Signaling Technology), phospho-JNK (Thr183/Tyr185) (#9251; Cell Signaling Technology), phospho-p38 MAPK (Thr180/Tyr182) (D3F9; Cell Signaling Technology), phospho-RAF1 (Ser289/296/301) (#9431; Cell Signaling Technology), β-actin (AC-15; Sigma-Aldrich), glyceraldehyde-3-phosphate dehydrogenase (GAPDH) (sc-32233; Santa Cruz Biotechnology), purified mouse IgG2b, κ Isotype Control Antibody (MPC-11; BioLegend, San Diego, CA, USA), peroxidase-conjugated goat anti-rabbit IgG (H + L) (111-035-144; Jackson ImmunoResearch Laboratories, West Grove, PA, USA), peroxidase-conjugated goat anti-mouse IgG (H + L) (115-035-146; Jackson ImmunoResearch Laboratories), and phycoerythrin-conjugated donkey anti-mouse IgG (715-116-115; Jackson ImmunoResearch Laboratories).

### 4.4. Preparation of Cell Lysates and Medium Fractions

Cell lysates and medium fractions were prepared as previously described [46,47]. Cells were treated with the indicated compounds in the corresponding FBS-free medium. Culture medium was subjected to methanol/chloroform precipitation, and precipitated proteins were collected by centrifugation. Cells were harvested and lysed in 1% Triton X-100 lysis buffer supplemented with complete protease inhibitor cocktail (Merck, Darmstadt, Germany) and phosphatase inhibitor cocktail (Nacalai Tesque). The lysates were clarified by centrifugation, and the resulting supernatants were collected. Protein concentrations were measured using Protein Assay CBB Solution (Nacalai Tesque).

### 4.5. Western Blotting

Western blotting was conducted as previously described [46,47]. Proteins were separated by SDS-PAGE and transferred onto nitrocellulose membranes (Pall Corporation, Pensacola, FL, USA, and Fujifilm Wako Pure Chemical Corporation). The membranes were blocked with 5% skim milk at 4 °C overnight and then incubated with primary and secondary antibodies, with washing in 0.5% Tween 20–phosphate-buffered saline between incubations. Protein bands were visualized by chemiluminescence using Amersham ECL Western Blotting Detection Reagent (GE Healthcare Japan, Tokyo, Japan). Blot images were acquired using Amersham Imager 680 (GE Healthcare Japan). Band intensities were quantified using ImageQuant TL software 7.0.1.0 (GE Healthcare Japan). The membranes were incubated with Stripping Solution (Fujifilm Wako Pure Chemical Corporation) and reprobed with antibodies against β-actin or GAPDH as loading controls. The band intensities of total and phosphorylated proteins were normalized to the corresponding loading controls. Phosphorylated to total protein ratios were subsequently calculated.

### 4.6. ELISA

Soluble TNF-R1 in the culture medium was quantified using the Duoset^TM^ ELISA kit for human TNF-R1 (DY225-05; R&D Systems, Minneapolis, MN, USA). Culture medium was collected as previously described [59]. The horseradish peroxidase reaction was developed using 1,2-diaminobenzene and H_2_O_2_. Absorbance was measured at 450 nm using an iMark microplate reader (Bio-Rad, Hercules, CA, USA). The concentration of soluble TNF-R1 was measured from a standard curve generated using recombinant human TNF-R1.

### 4.7. Flow Cytometry

Cells were collected and incubated with either mouse anti-human TNF-R1 (H-5; 10 µg/mL) or a mouse control antibody (MPC-11; 10 µg/mL), followed by incubation with a phycoerythrin-conjugated anti-mouse IgG secondary antibody. Cells were washed between antibody incubations. Fluorescence was measured using a MACSQuant flow cytometer (Miltenyi Biotec, San Jose, CA, USA). Acquired data were processed using FlowJo software 10.8 (Tomy Digital Biology, Tokyo, Japan).

### 4.8. Statistical Analysis

Data are presented as the mean ± standard error of the mean (SEM) from at least three independent experiments. KaleidaGraph 4.5 software (Hulinks, Tokyo, Japan) was used for statistical analyses and graph generation. Differences among multiple groups were assessed by a one-way ANOVA followed by Tukey’s post hoc test. The significance of differences was defined as *p* < 0.05.

## 5. Conclusions

The present results demonstrate that alantolactone promoted the ectodomain shedding of TNF-R1 in A549 cells as well as in 293T and HT-1080 cells through a metalloproteinase-dependent mechanism involving the activation of the RAF1–ERK signaling pathway. Alantolactone reduced cell-surface TNF-R1 expression while increasing the release of soluble TNF-R1 into the culture medium, and both effects were reversed by the metalloproteinase inhibitor GM6001. Among the MAPK family members examined, alantolactone selectively induced RAF1 and ERK phosphorylation without markedly affecting p38 MAPK or JNK, and the pharmacological inhibition of the MEK–ERK axis, but not p38 MAPK or JNK, attenuated alantolactone-induced TNF-R1 shedding. These results provide mechanistic insights into how alantolactone modulates TNF-α receptor availability through the RAF1–ERK signaling pathway. Future studies are needed to identify the specific ADAM protease responsible for alantolactone-induced TNF-R1 shedding, to clarify how ERK activation regulates proteolytic activity, and to elucidate how alantolactone activates the RAF1–ERK signaling axis.

## Data Availability

The data presented in this study are available upon reasonable request from the corresponding author.

## Supplementary Materials

The following supporting information can be downloaded at: https://www.mdpi.com/article/10.3390/molecules31183296/s1, Figure S1: Original blots in Figure 2A (Medium); Figure S2: Original blots in Figure 2B (Cell lysate); Figure S3: Original blots (1) in Figure 2C; Figure S4: Original blots (2) in Figure 2C; Figure S5: Original blots (3) in Figure 2C; Figure S6: Original blots (1) in Figure 2D; Figure S7: Original blots (2) in Figure 2D; Figure S8: Original blots (3) in Figure 2D; Figure S9: Original blots (4) in Figure 2D; Figure S10: Original blots (5) in Figure 2D; Figure S11: Original blots in Figure 3A (Medium); Figure S12: Original blots in Figure 3A (Cell lysate); Figure S13: Original blots (1) in Figure 3B; Figure S14: Original blots (2) in Figure 3B; Figure S15: Original blots (3) in Figure 3B; Figure S16: Original blots (1) in Figure 3C; Figure S17: Original blots (2) in Figure 3C; Figure S18: Original blots (3) in Figure 3C; Figure S19: Original blots in Figure 4A (Medium); Figure S20: Original blots in Figure 4A (Cell lysate); Figure S21: Original blots (1) in Figure 4B; Figure S22: Original blots (2) in Figure 4B; Figure S23: Original blots (3) in Figure 4B; Figure S24: Original blots (1) in Figure 4C; Figure S25: Original blots (2) in Figure 4C; Figure S26: Original blots (3) in Figure 4C; Figure S27: Original blots in Figure 4D (Medium); Figure S28: Original blots in Figure 4D (Cell lysate); Figure S29: Original blots (1) in Figure 4E; Figure S30: Original blots (2) in Figure 4E; Figure S31: Original blots (3) in Figure 4E; Figure S32: Original blots (1) in Figure 4F; Figure S33: Original blots (2) in Figure 4F; Figure S34: Original blots (3) in Figure 4F; Figure S35: Original blots in Figure 5A (Medium); Figure S36: Original blots in Figure 5A (Cell lysate); Figure S37: Original blots (1) in Figure 5B; Figure S38: Original blots (2) in Figure 5B; Figure S39: Original blots (3) in Figure 5B; Figure S40: Original blots (1) in Figure 5C; Figure S41: Original blots (2) in Figure 5C; Figure S42: Original blots (3) in Figure 5C; Figure S43: Original blots in Figure 6A (Phospho-ERK); Figure S44: Original blots in Figure 6A (ERK); Figure S45: Original blots (1) in Figure 6B,D; Figure S46: Original blots (2) in Figure 6B,D; Figure S47: Original blots (3) in Figure 6B,D; Figure S48: Original blots (1) in Figure 6C,D; Figure S49: Original blots (2) in Figure 6C,D; Figure S50: Original blots (3) in Figure 6C,D; Figure S51: Original blots in Figure 6E (Phospho-p38 MAPK); Figure S52: Original blots in Figure 6E (p38 MAPK); Figure S53: Original blots (1) in Figure 6F,H; Figure S54: Original blots (2) in Figure 6F,H; Figure S55: Original blots (3) in Figure 6F,H; Figure S56: Original blots (1) in Figure 6G,H; Figure S57: Original blots (2) in Figure 6G,H; Figure S58: Original blots (3) in Figure 6G,H; Figure S59: Original blots in Figure 6I (Phospho-JNK); Figure S60: Original blots in Figure 6I (JNK); Figure S61: Original blots (1) in Figure 6J,L,M,O; Figure S62: Original blots (2) in Figure 6J,L,M,O; Figure S63: Original blots (3) in Figure 6J,L,M,O; Figure S64: Original blots (1) in Figure 6K,L,N,O; Figure S65: Original blots (2) in Figure 6K,L,N,O; Figure S66: Original blots (3) in Figure 6K,L,N,O; Figure S67: Original blots in Figure 7A (Phospho-RAF1); Figure S68: Original blots in Figure 7A (RAF1); Figure S69: Original blots (1) in Figure 7B,D; Figure S70: Original blots (2) in Figure 7B,D; Figure S71: Original blots (3) in Figure 7B,D; Figure S72: Original blots (1) in Figure 7C,D; Figure S73: Original blots (2) in Figure 7C,D; Figure S74: Original blots (3) in Figure 7C,D.

## Abbreviations

The following abbreviations are used in this manuscript:

ADAM	A disintegrin and metalloproteinase
AP-1	Activator protein-1
EGFR	Epidermal growth factor receptor
ELISA	Enzyme-linked immunosorbent assay
ERK	Extracellular signal-regulated kinase
FBS	Fetal bovine serum
GAPDH	Glyceraldehyde-3-phosphate dehydrogenase
IC_50_	50% inhibitory concentration
JNK	c-Jun *N*-terminal kinase
MAPK	Mitogen-activated protein kinase
MEK	Mitogen-activated protein kinase/extracellular signal-regulated kinase kinase
NF-κB	Nuclear factor κB
RAF	Rapidly accelerated fibrosarcoma
SEM	Standard error of the mean
TNF-α	Tumor necrosis factor α
TNF-R1	Tumor necrosis factor receptor 1
