# Supplementary material for: Alantolactone Activates the Extracellular Signal-Regulated Kinase Signaling Pathway to Promote Tumor Necrosis Factor Receptor 1 Ectodomain Shedding
Source: Molecules. 2026 Sep 17;31(18):3296. doi: 10.3390/molecules31183296 (PMC13609282; doi:10.3390/molecules31183296)

Figure S1: Original blots in Figure 2A (Medium)

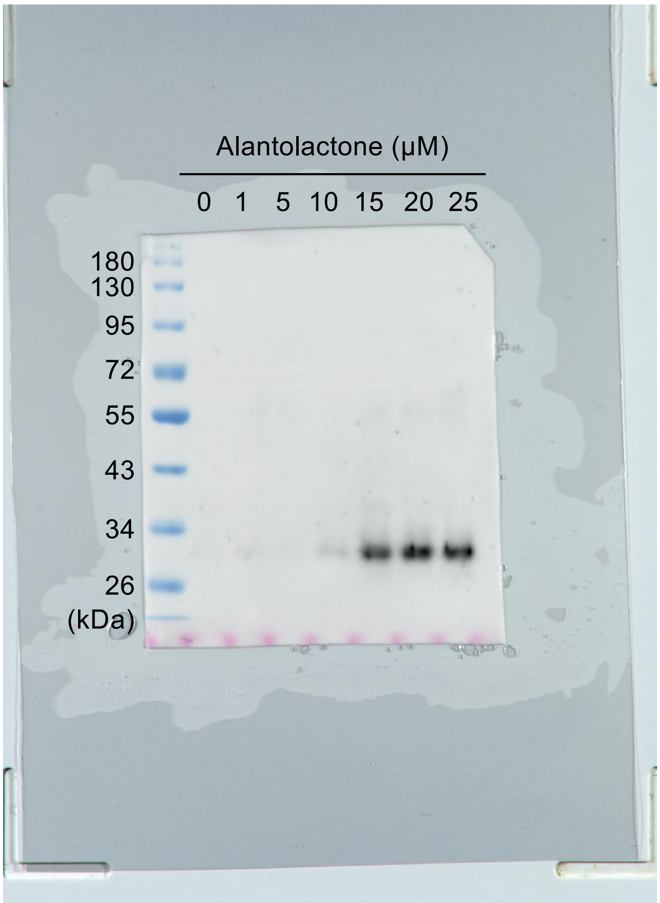

WB: TNF-R1

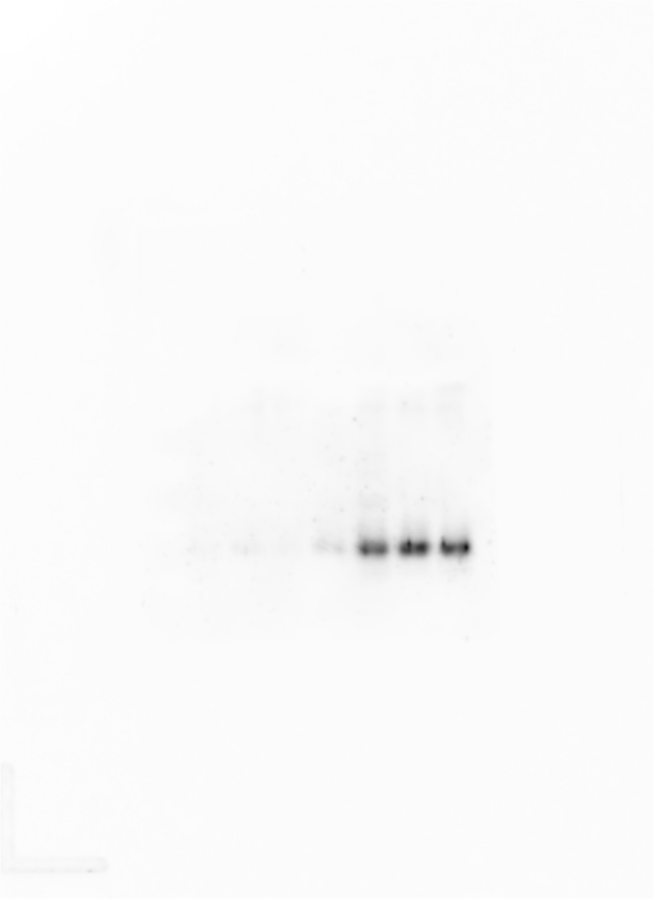

Figure S2: Original blots in Figure 2B (Cell lysate)

WB: TNF-R1

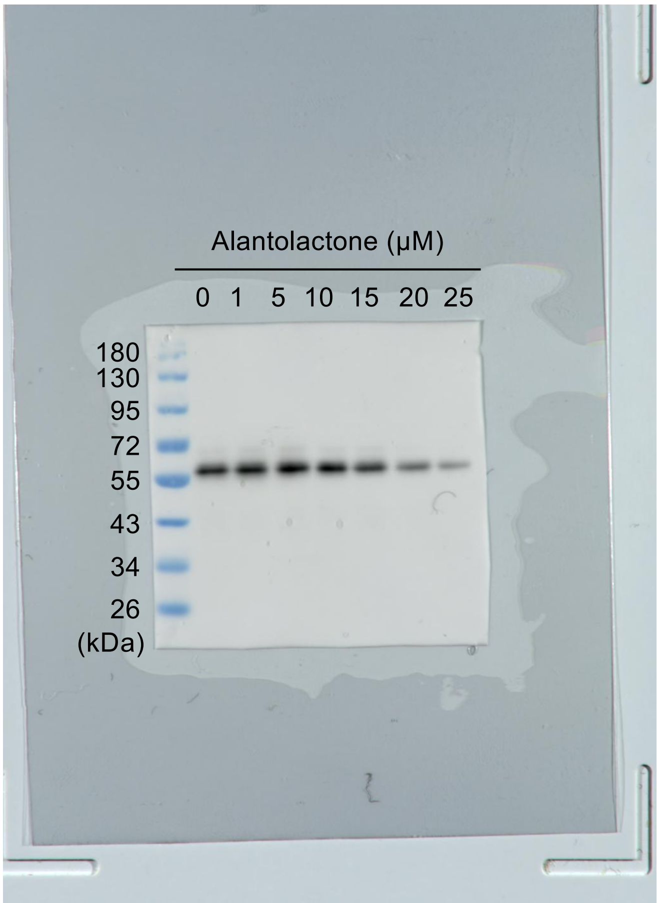

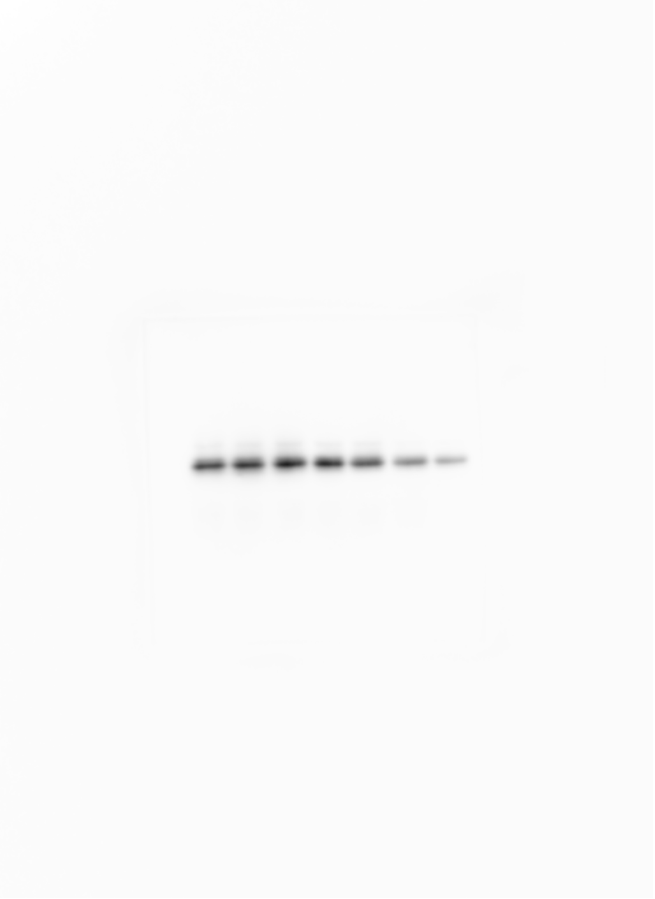

WB: β-Actin (reprobed)

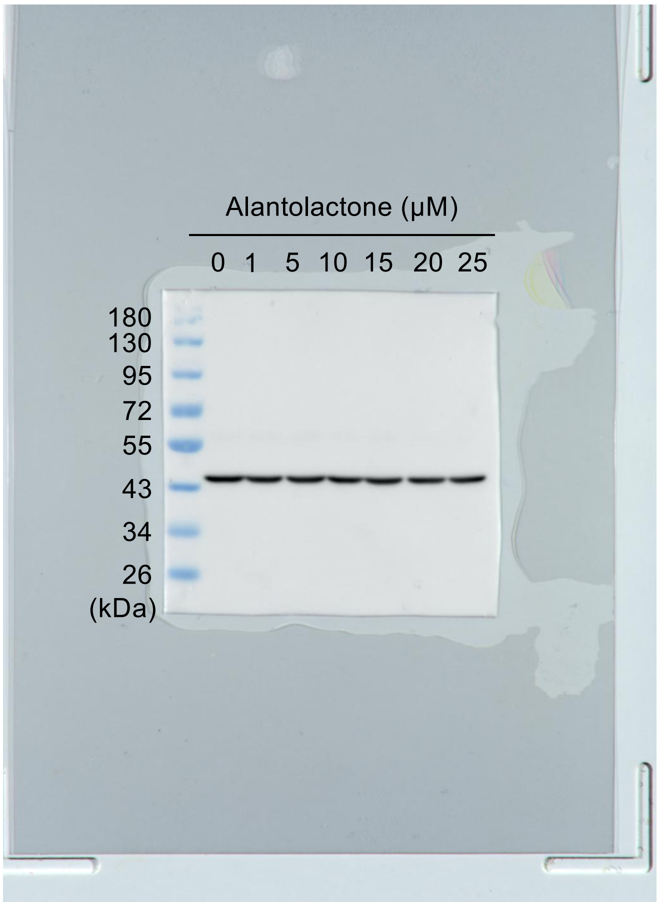

Figure S3: Original blots (1) in Figure 2C

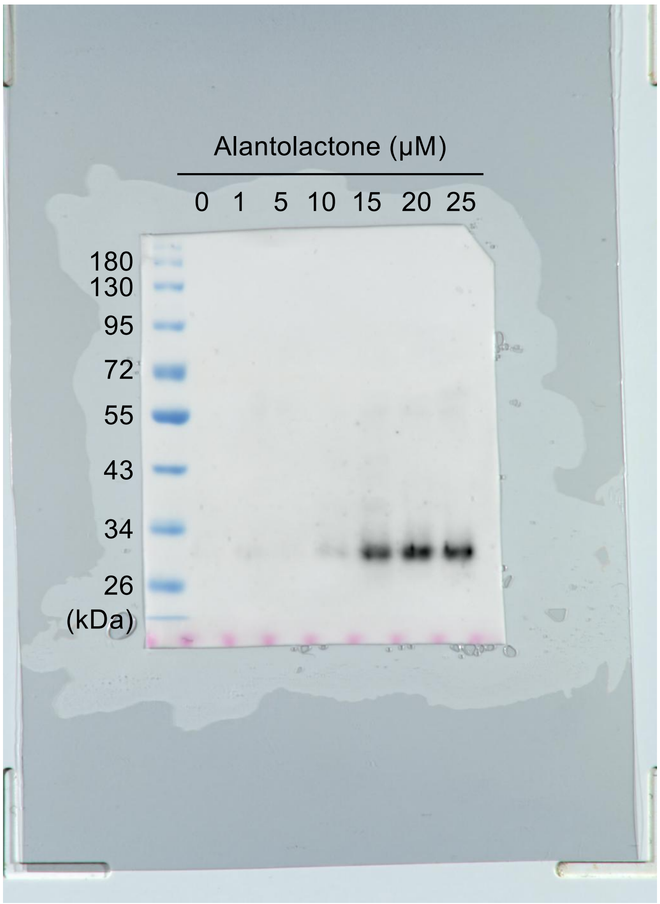

WB: TNF-R1

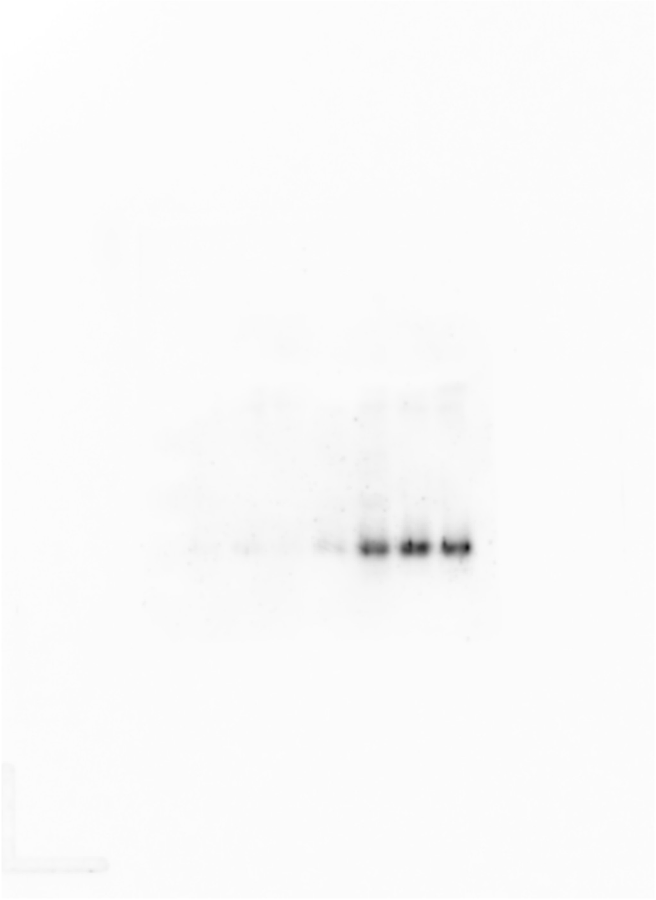

Figure S4: Original blots (2) in Figure 2C

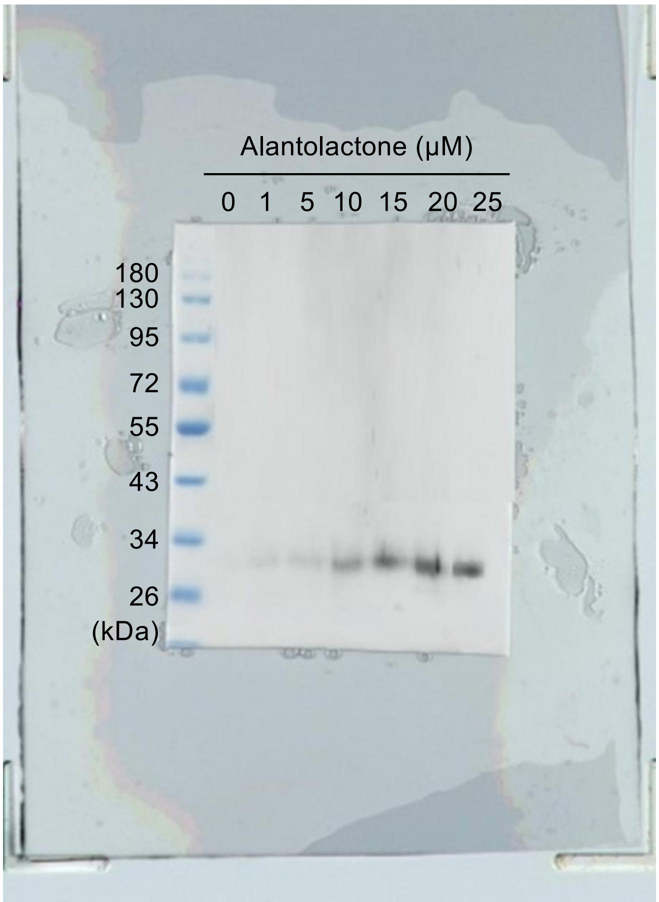

WB: TNF-R1

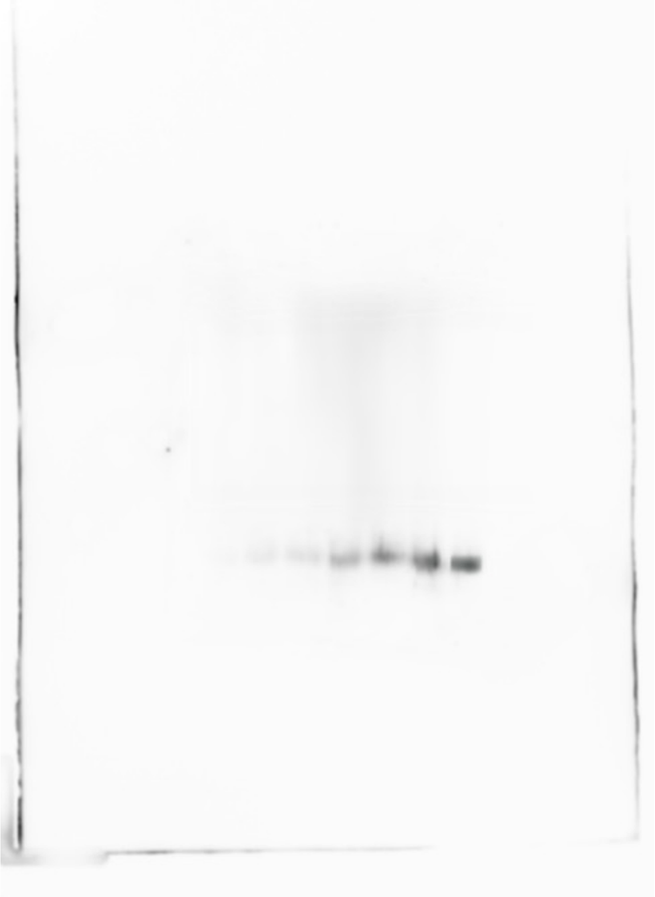

Figure S5: Original blots (3) in Figure 2C

WB: TNF-R1

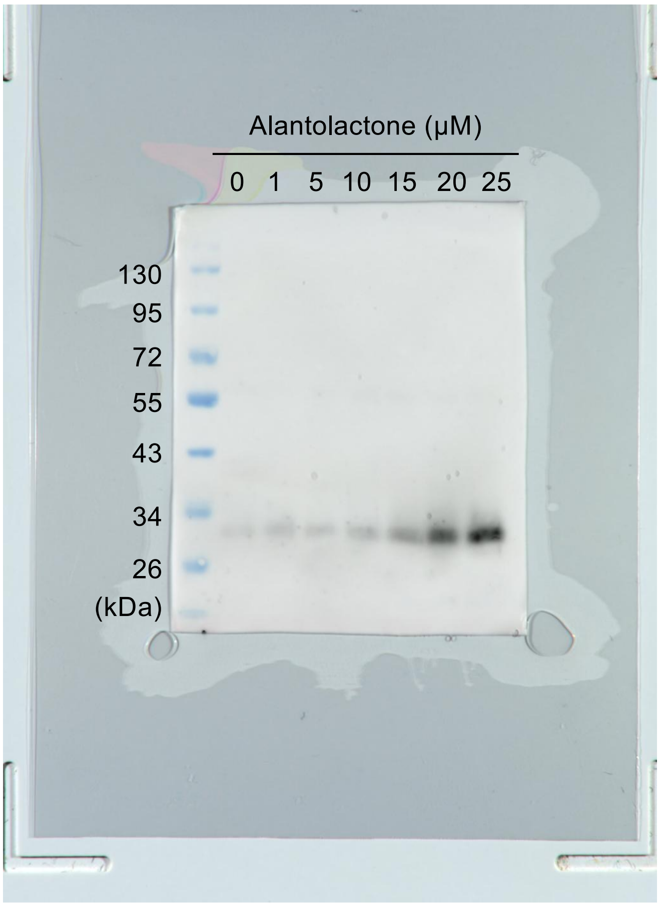

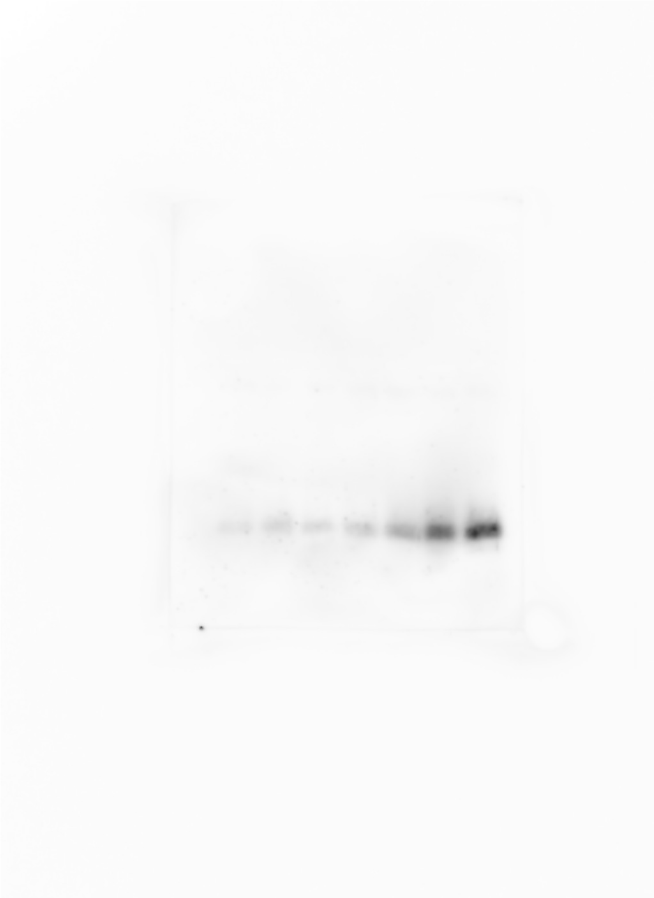

Figure S6: Original blots (1) in Figure 2D

WB: TNF-R1

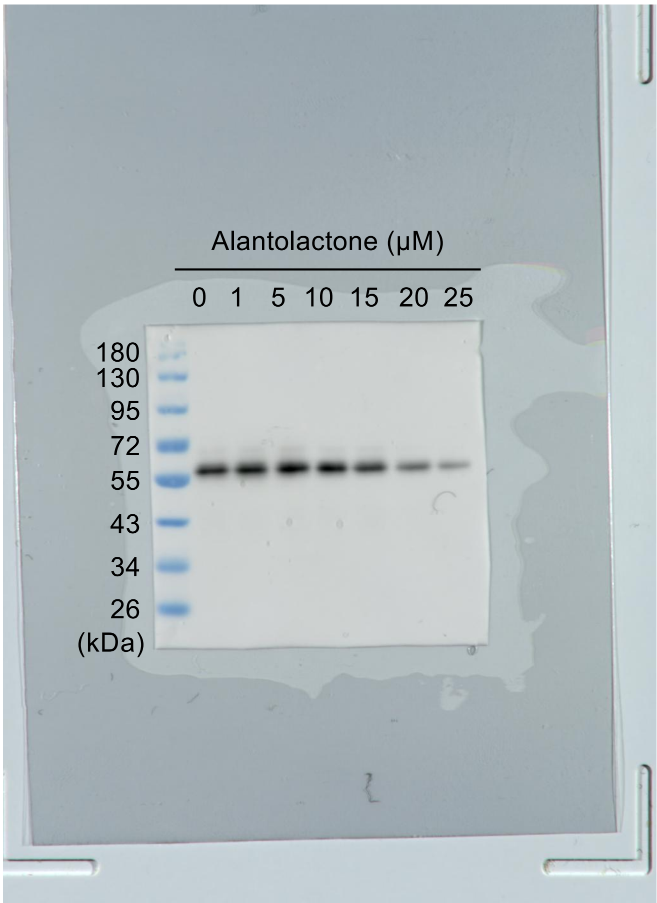

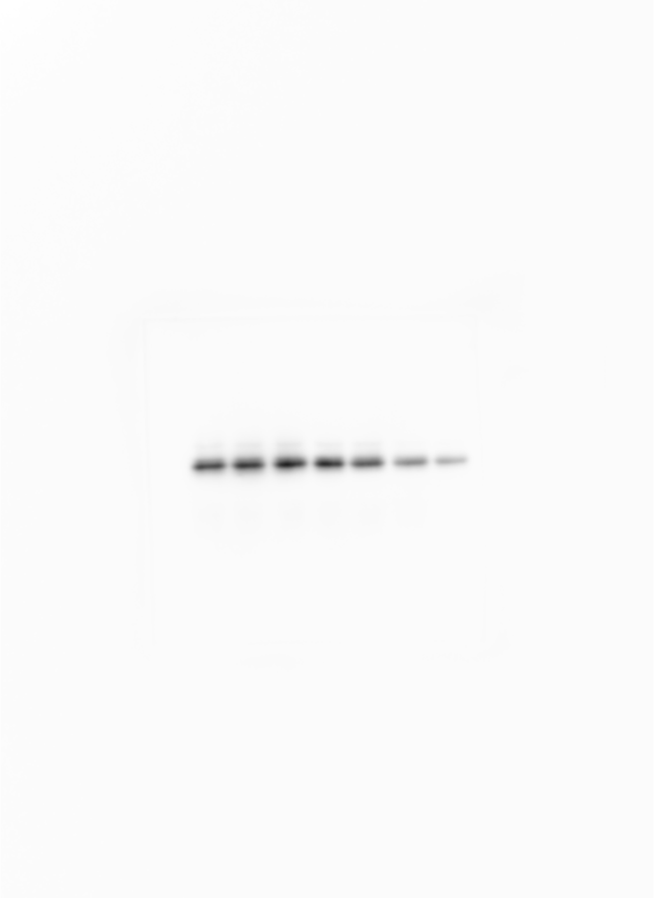

WB: β-Actin (reprobed)

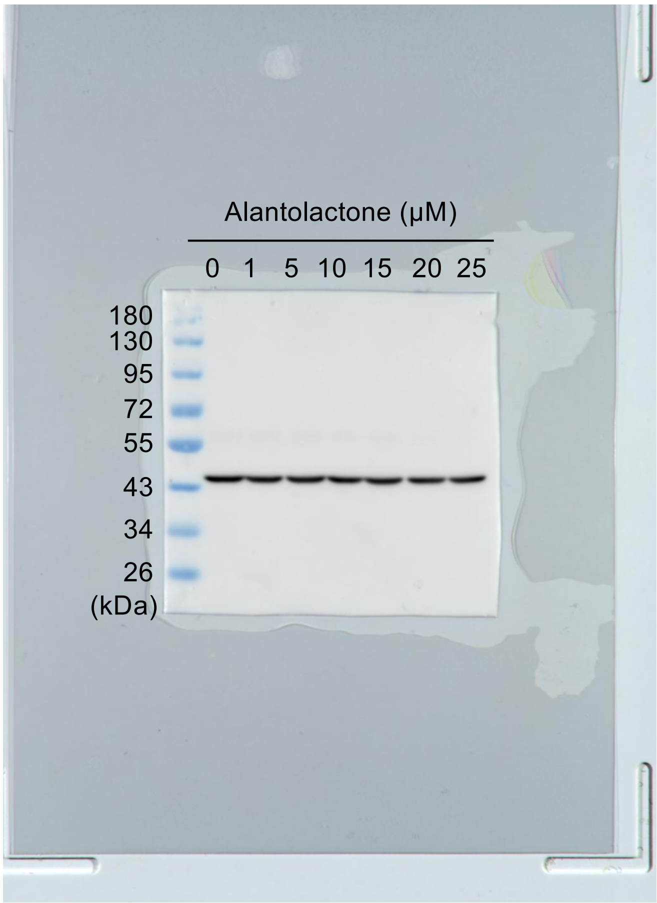

Figure S7: Original blots (2) in Figure 2D

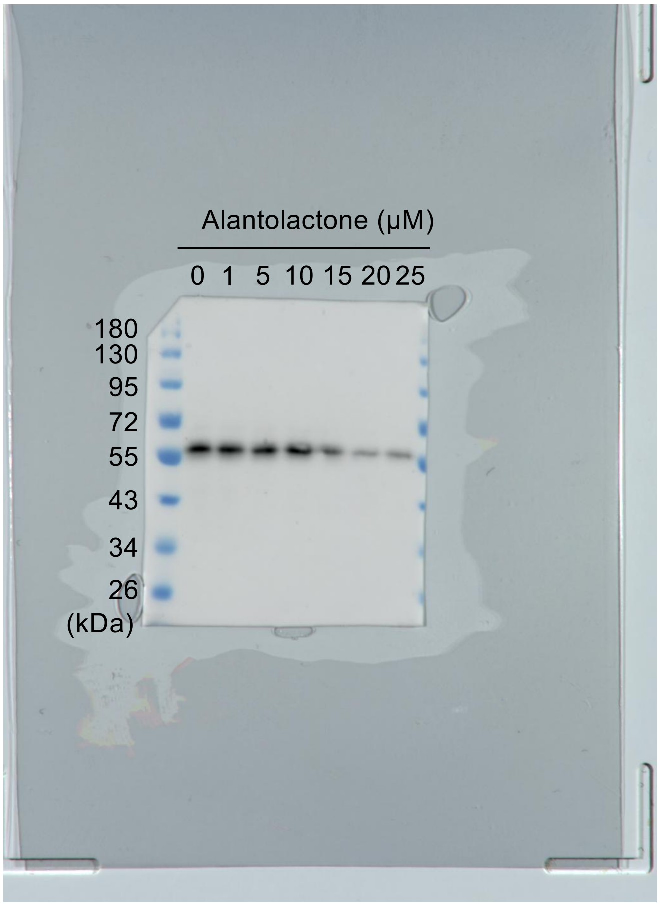

WB: TNF-R1

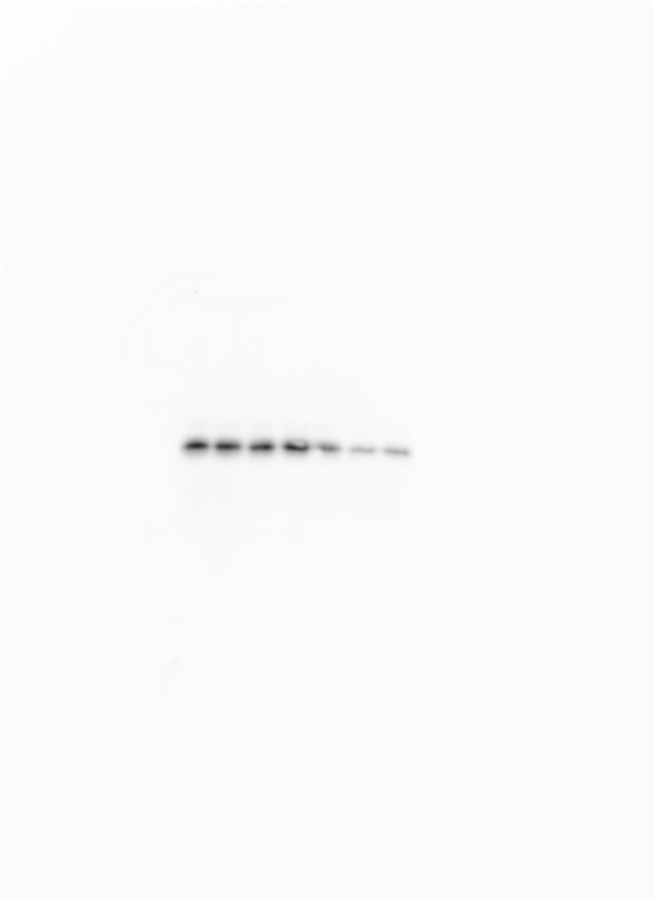

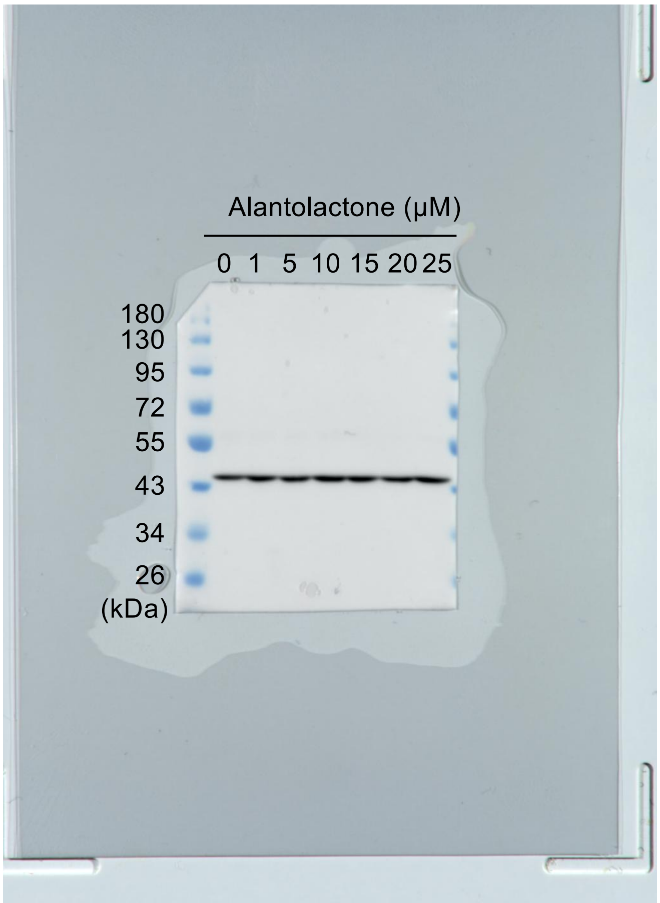

WB: β-Actin (reprobed)

Figure S8: Original blots (3) in Figure 2D

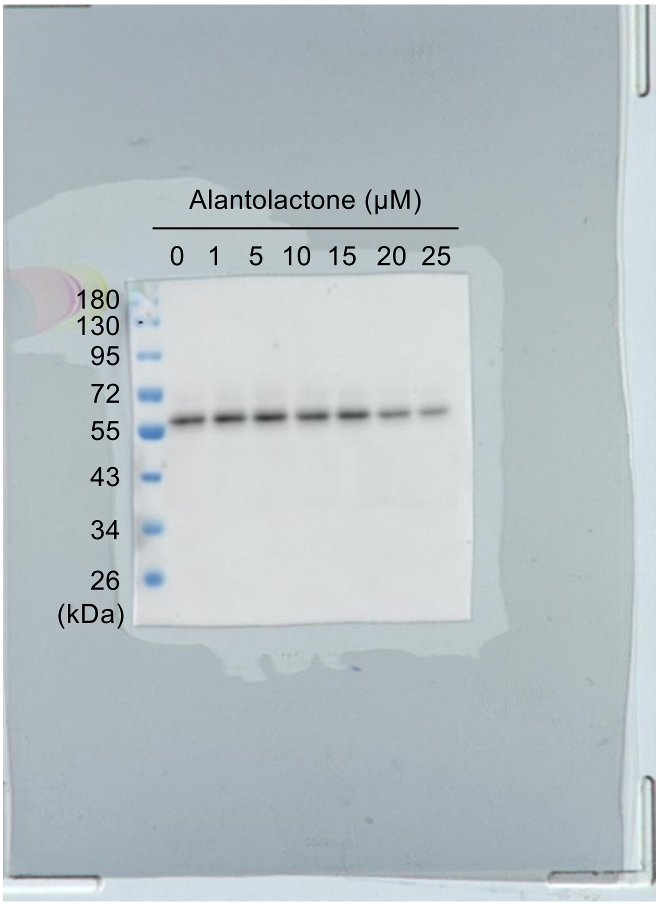

WB: TNF-R1

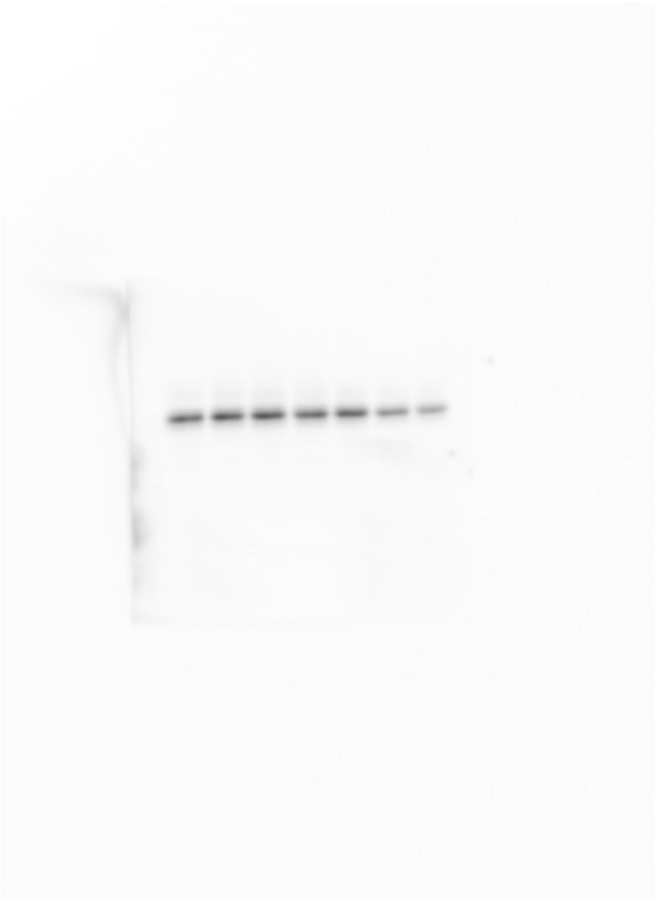

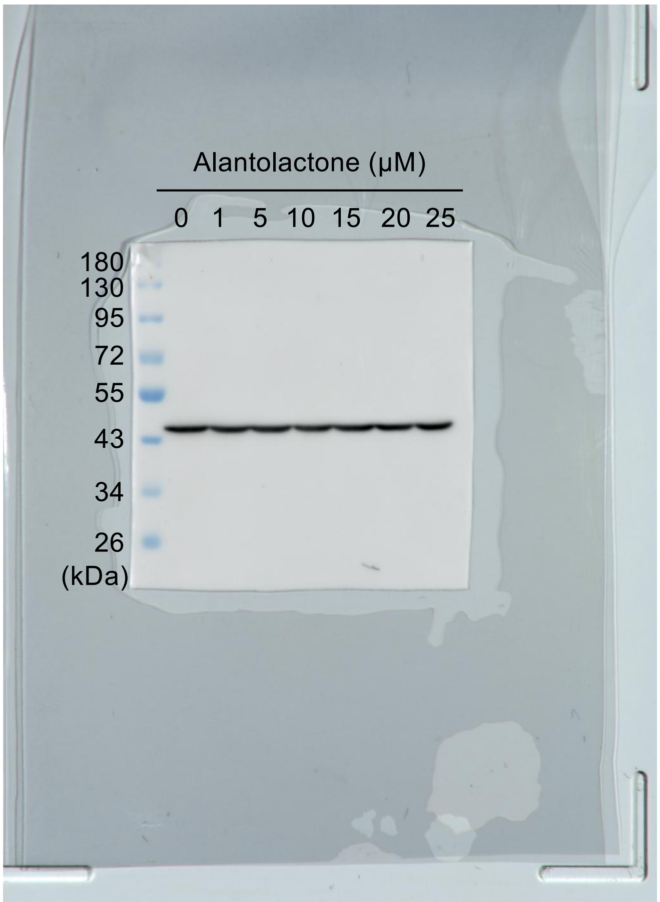

WB: β-Actin (reprobed)

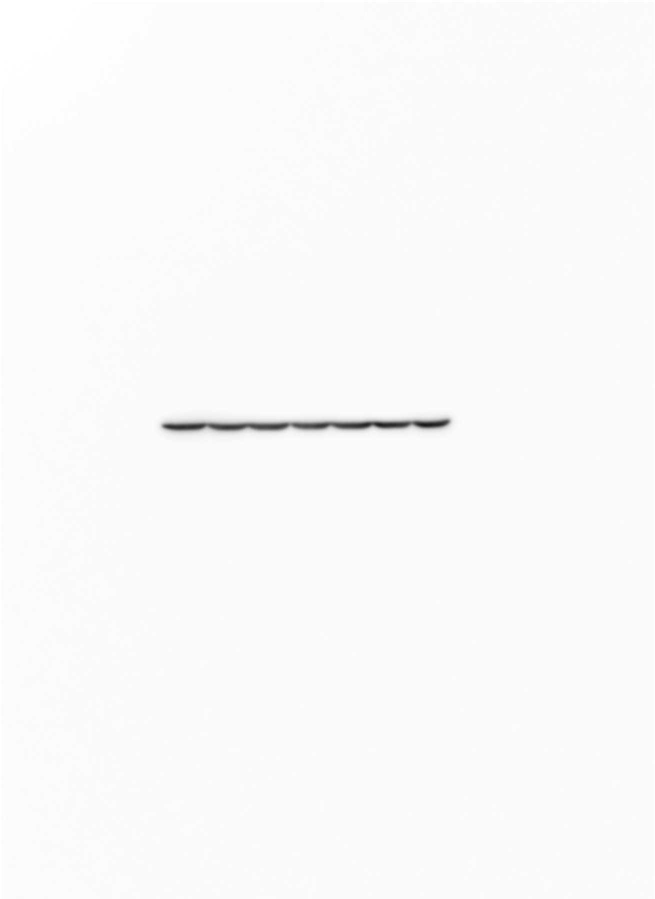

Figure S9: Original blots (4) in Figure 2D

WB: TNF-R1

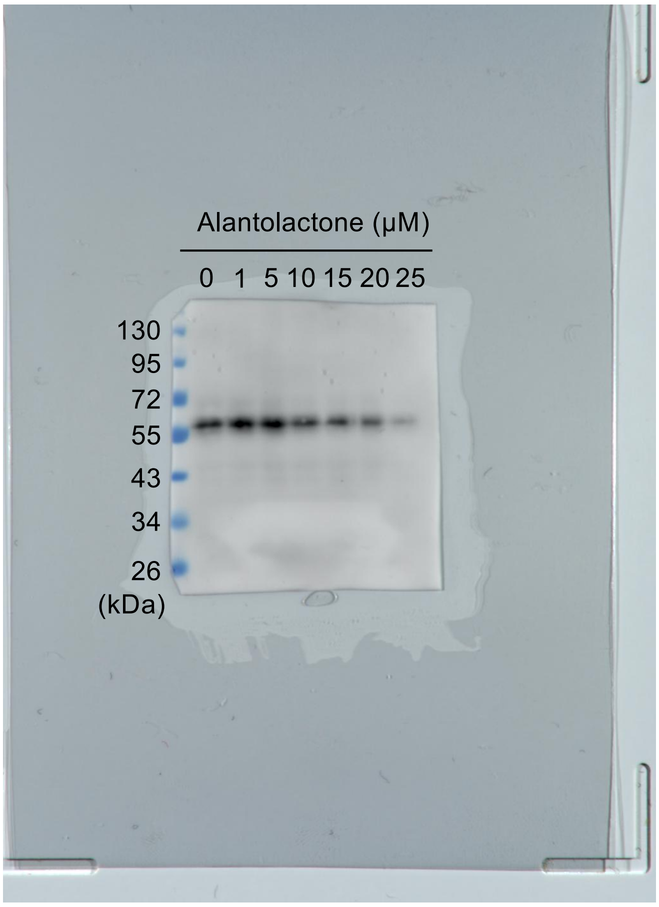

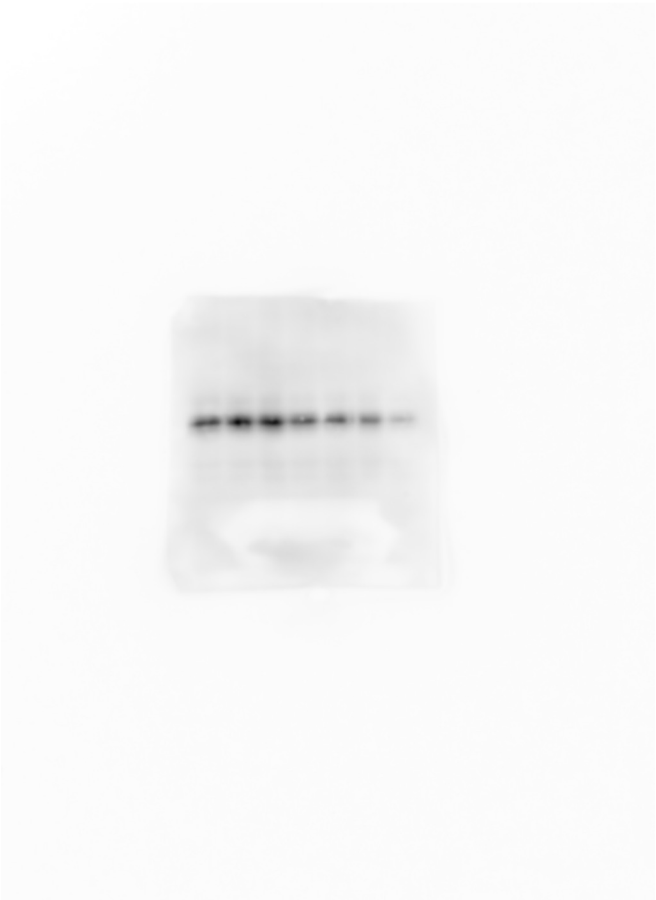

WB: β-Actin (reprobed)

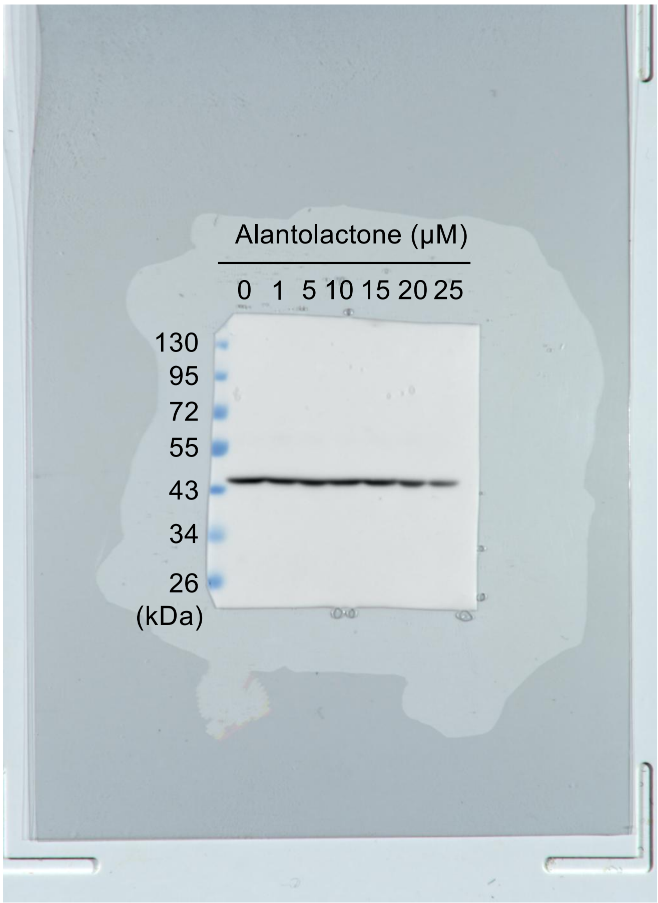

Figure S10: Original blots (5) in Figure 2D

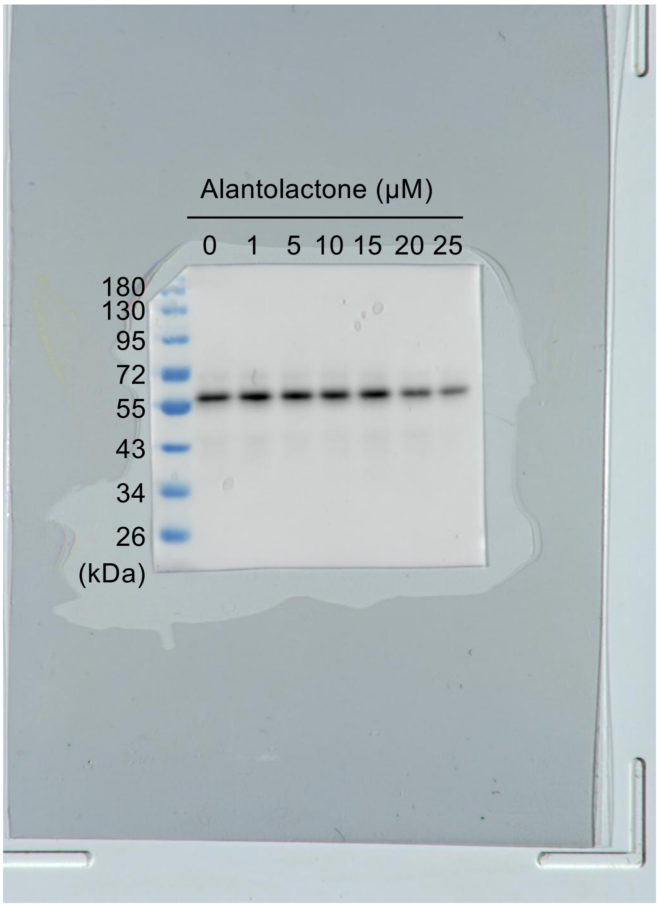

WB: TNF-R1

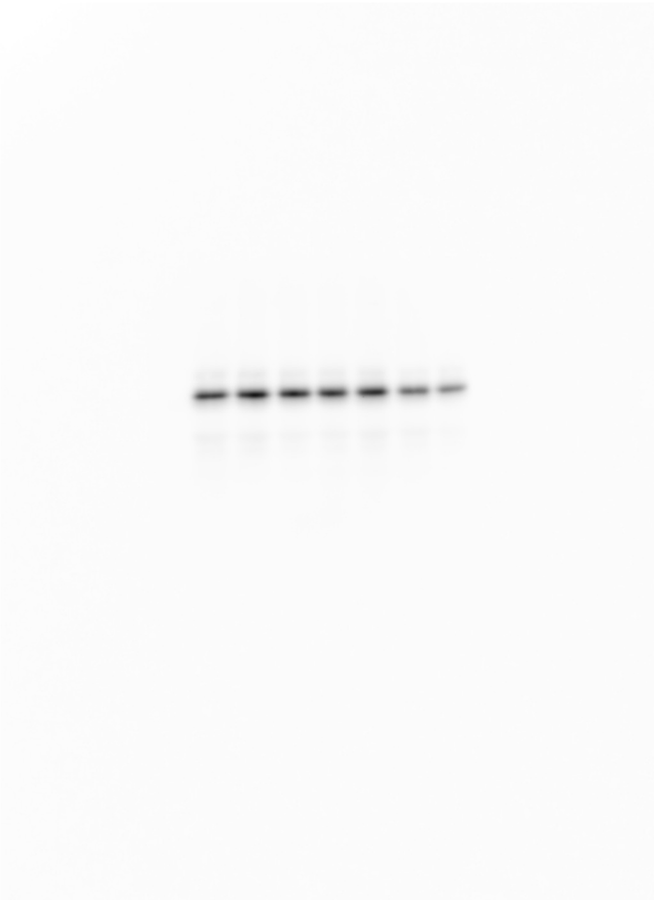

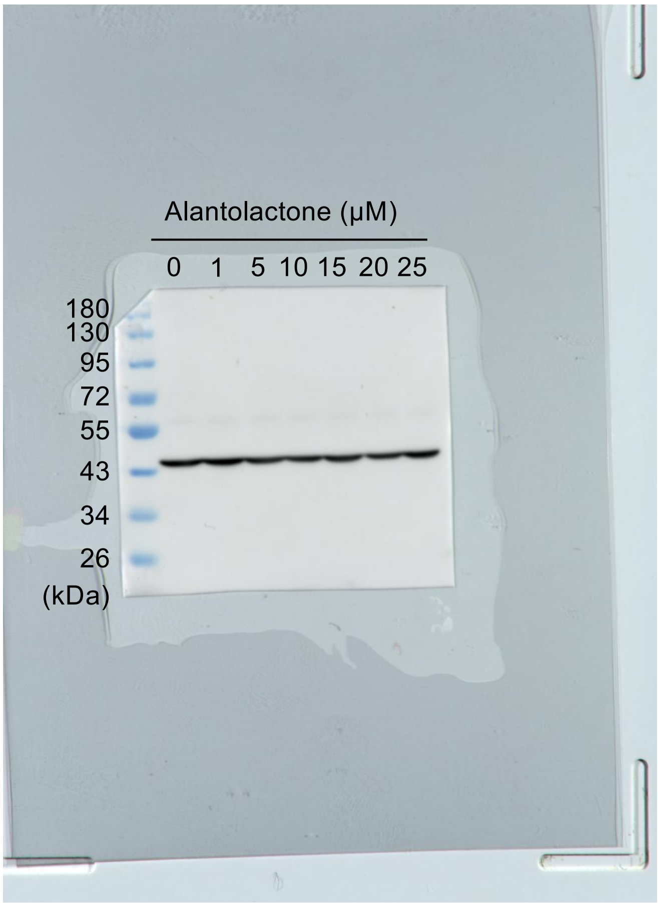

WB: β-Actin (reprobed)

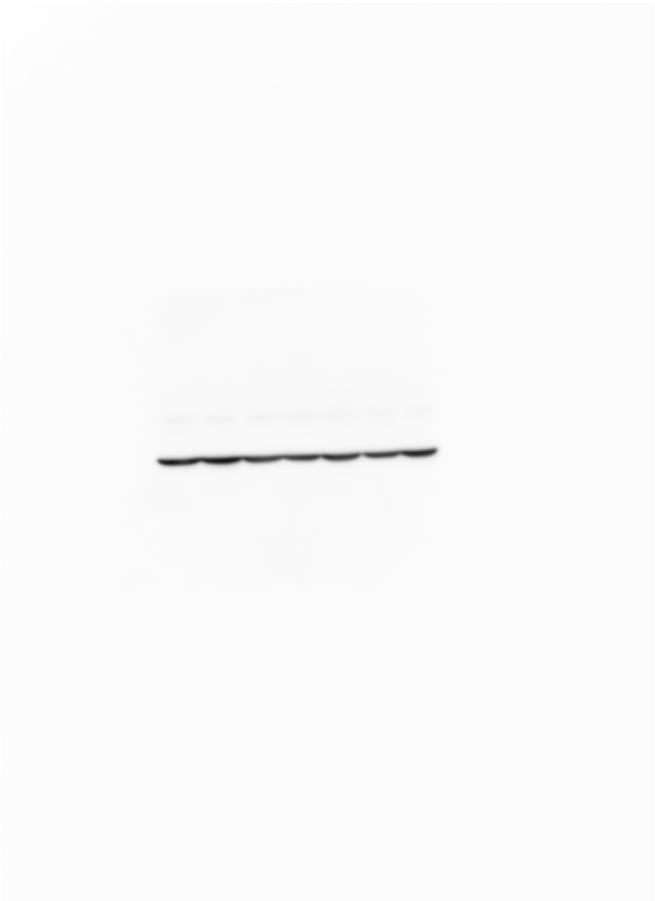

Figure S11: Original blots in Figure 3A (Medium)

WB: TNF-R1

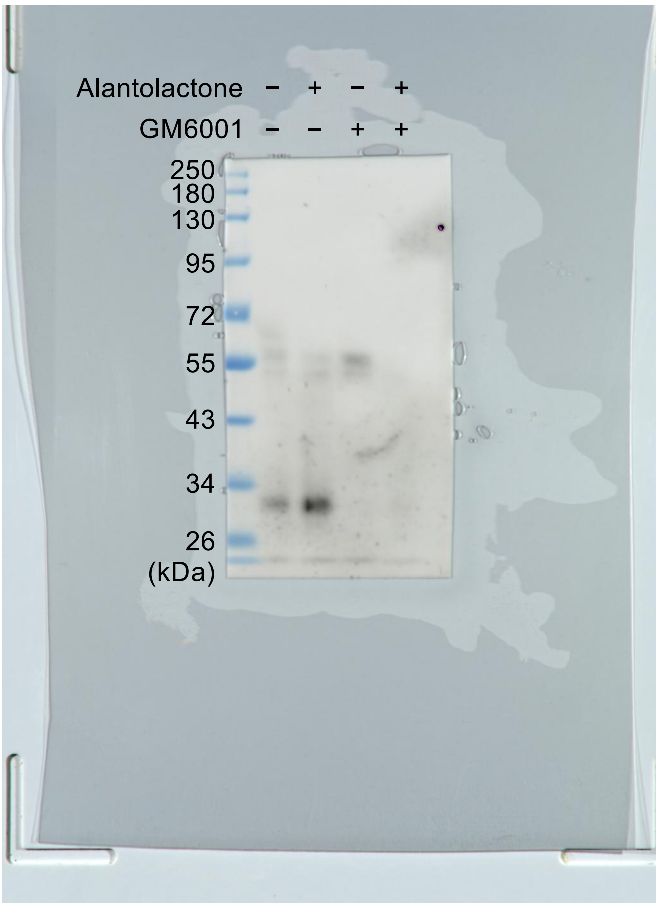

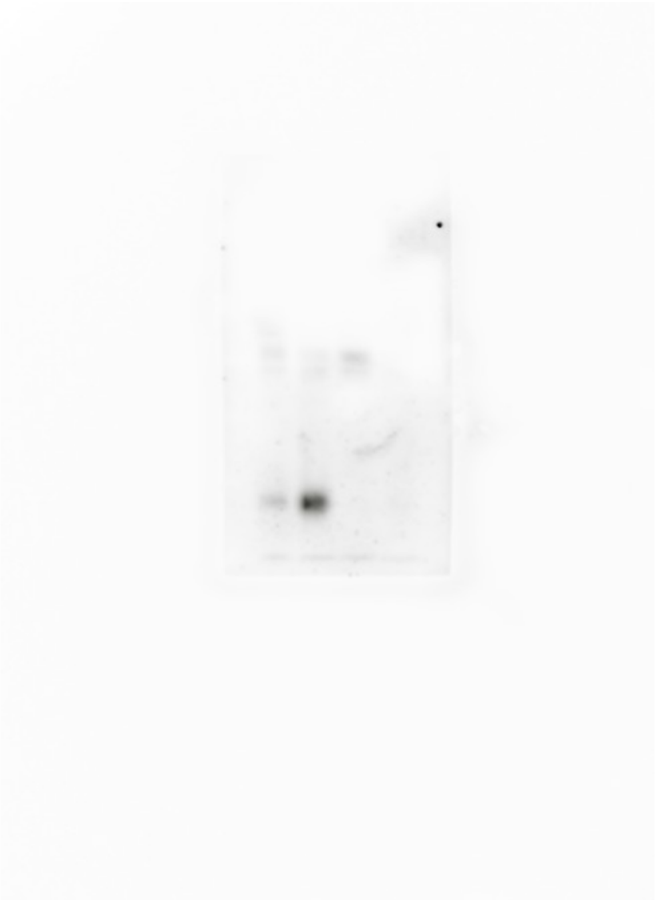

Figure S12: Original blots in Figure 3A (Cell lysate)

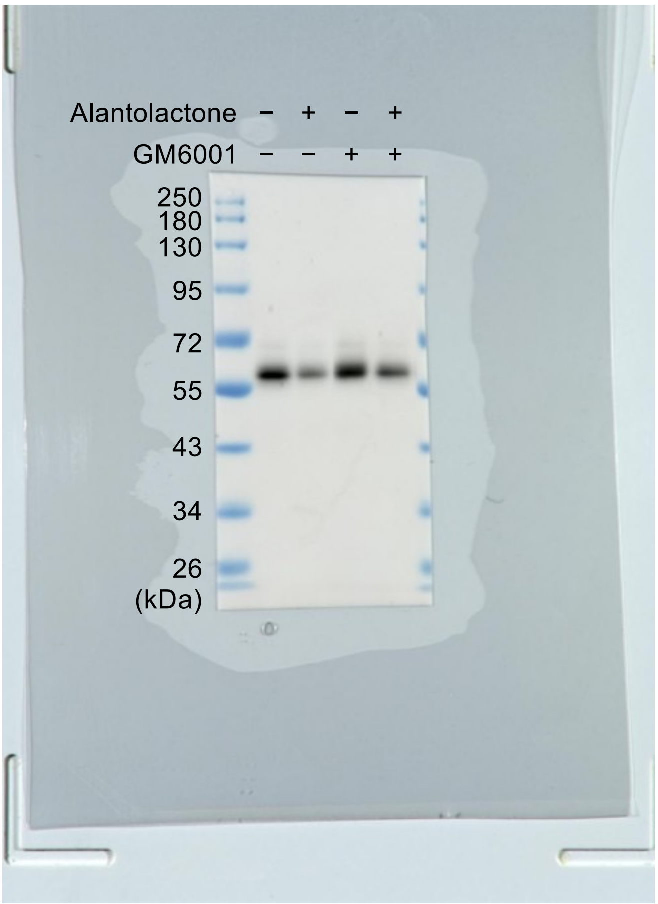

WB: TNF-R1

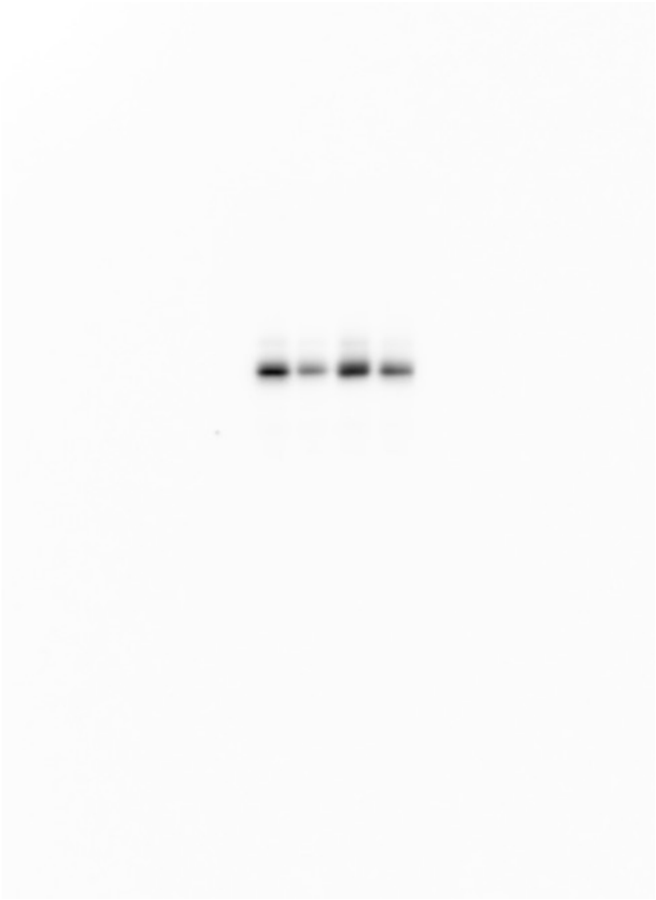

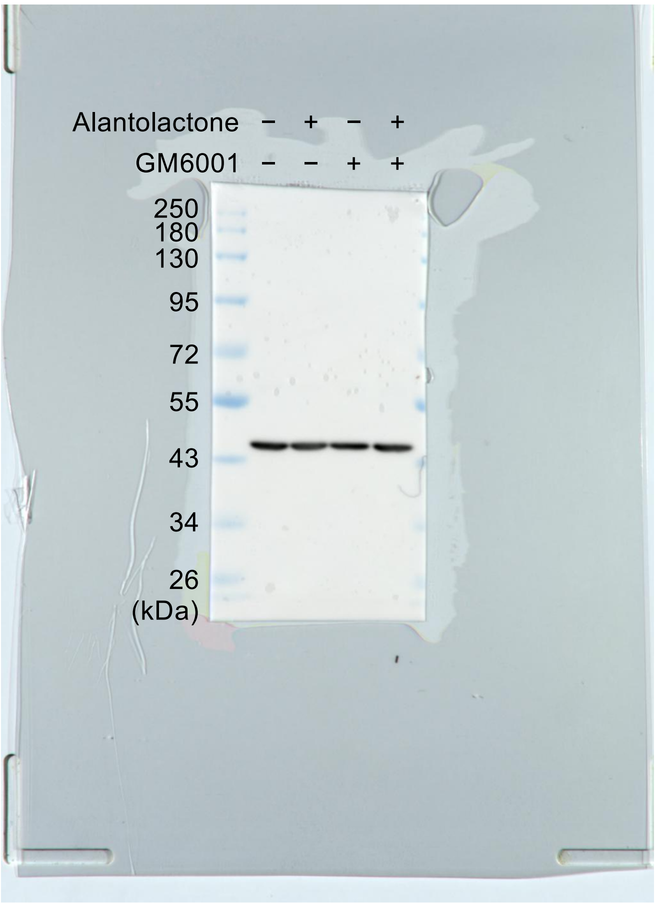

WB: β-Actin (reprobed)

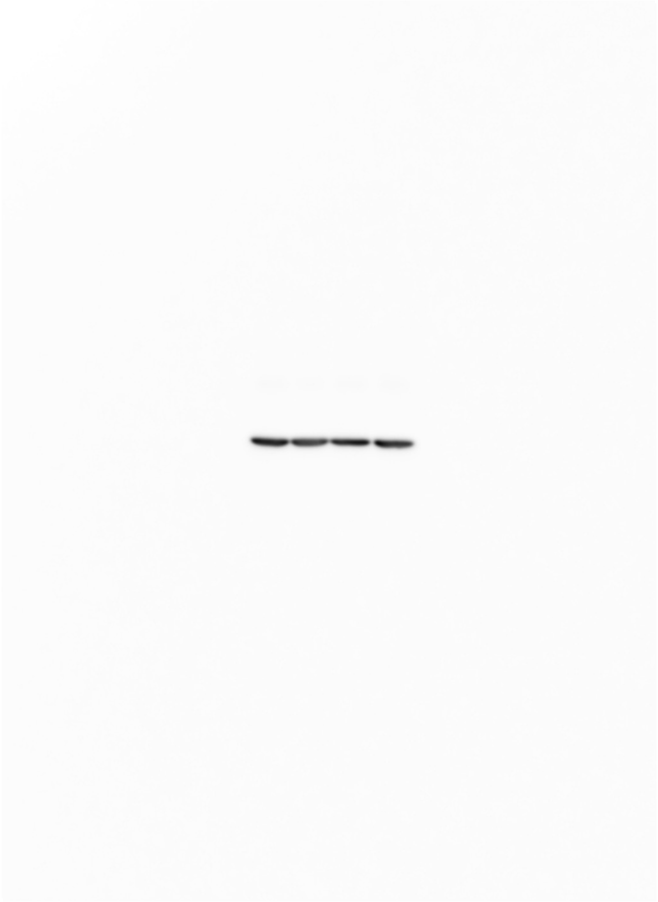

Figure S13: Original blots (1) in Figure 3B

WB: TNF-R1

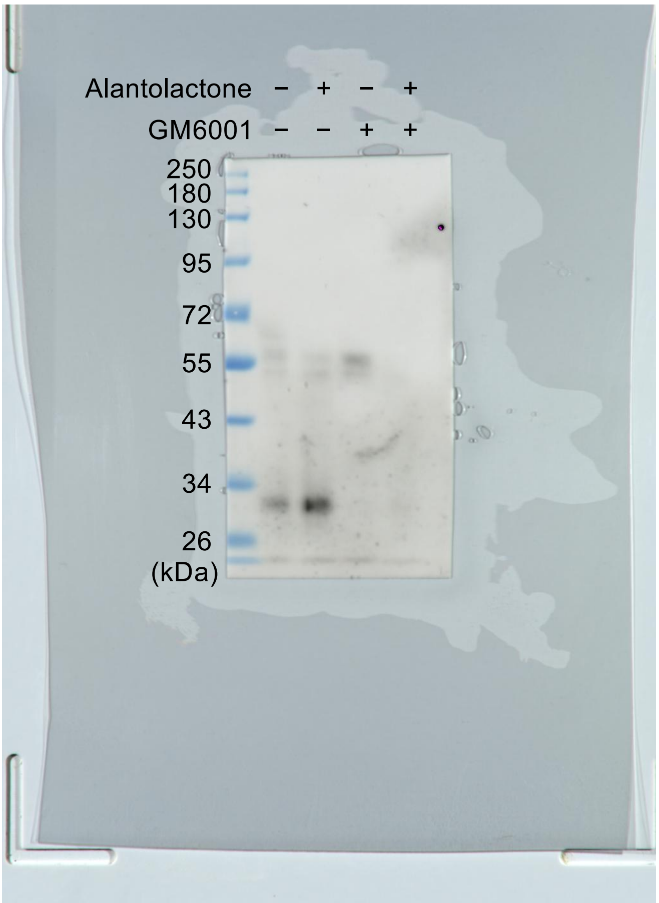

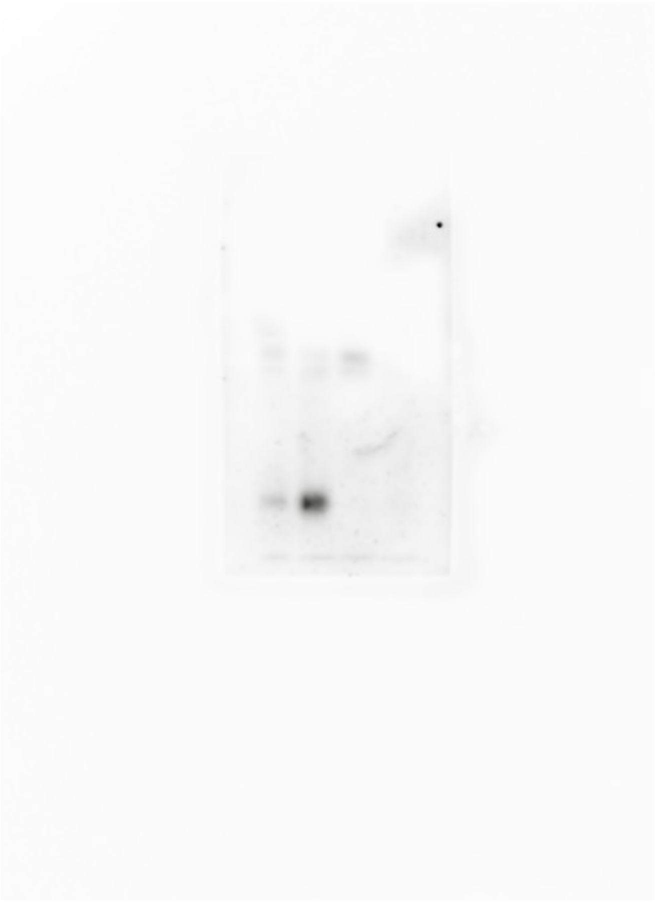

Figure S14: Original blots (2) in Figure 3B

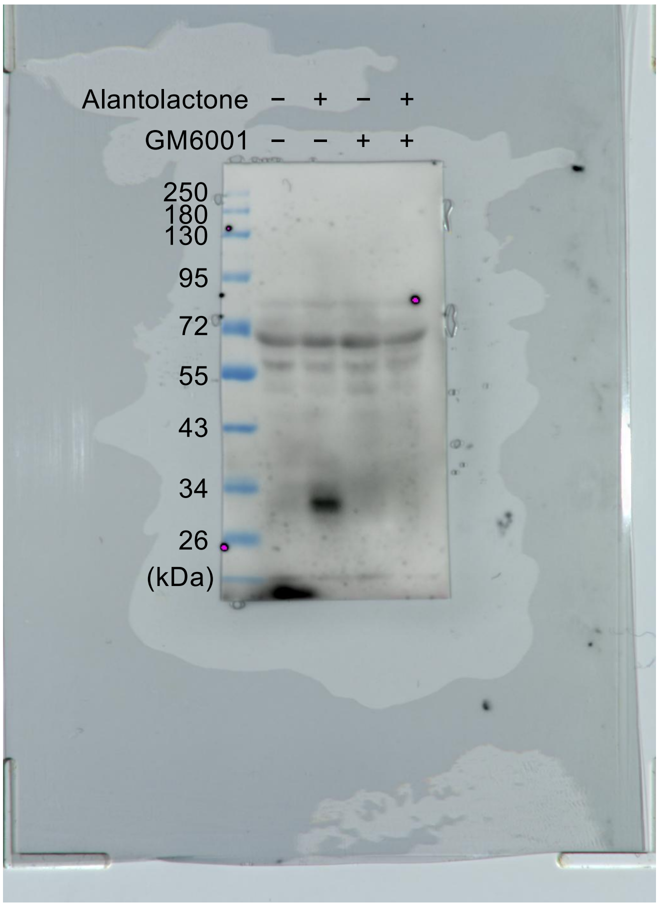

WB: TNF-R1

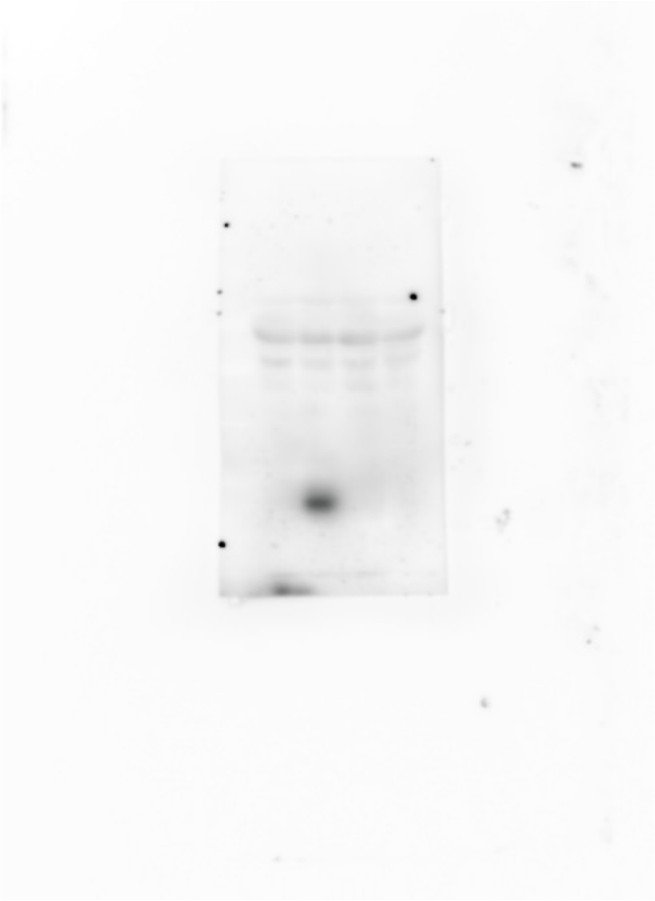

Figure S15: Original blots (3) in Figure 3B

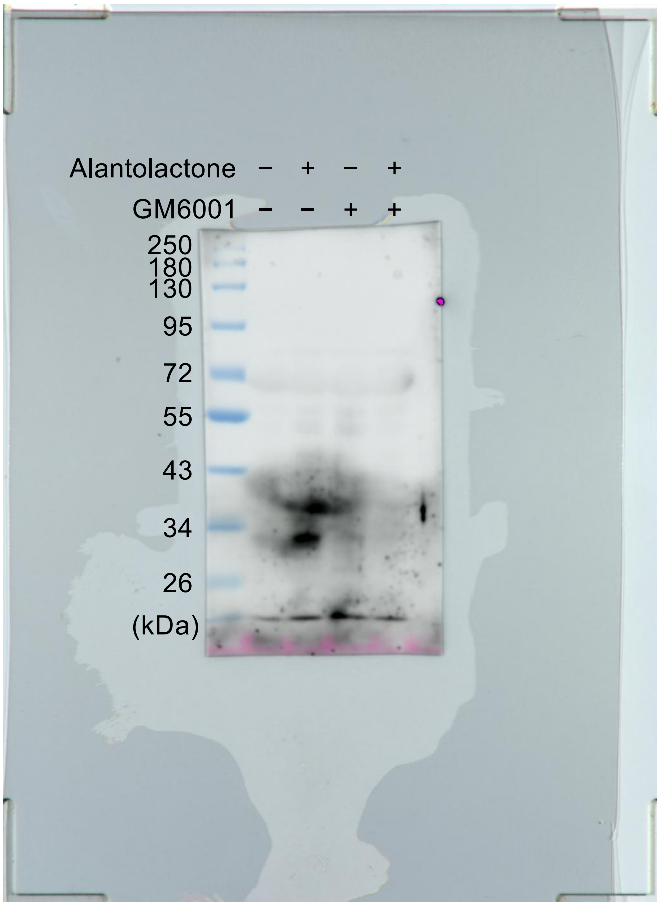

WB: TNF-R1

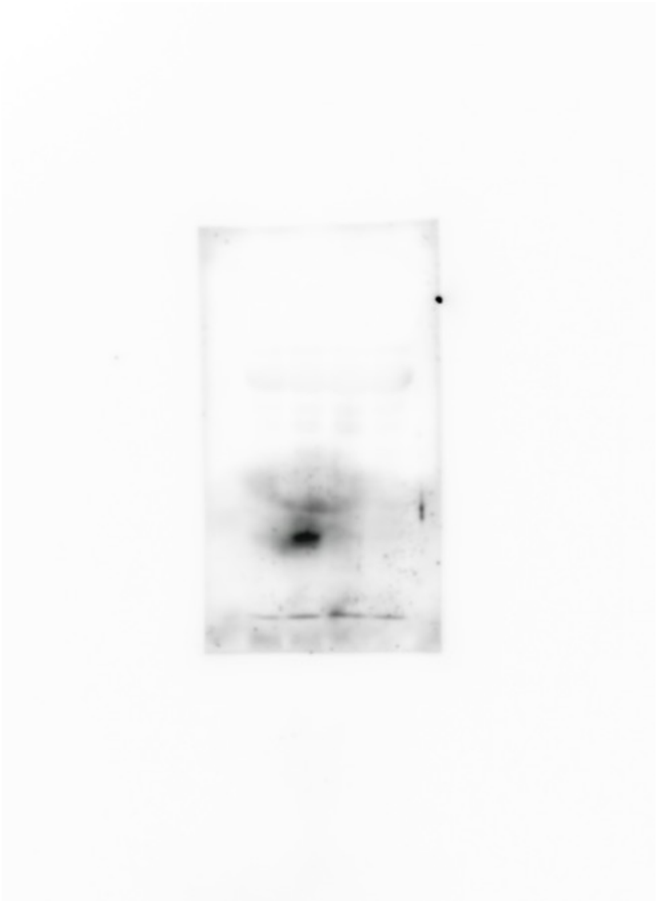

Figure S16: Original blots (1) in Figure 3C

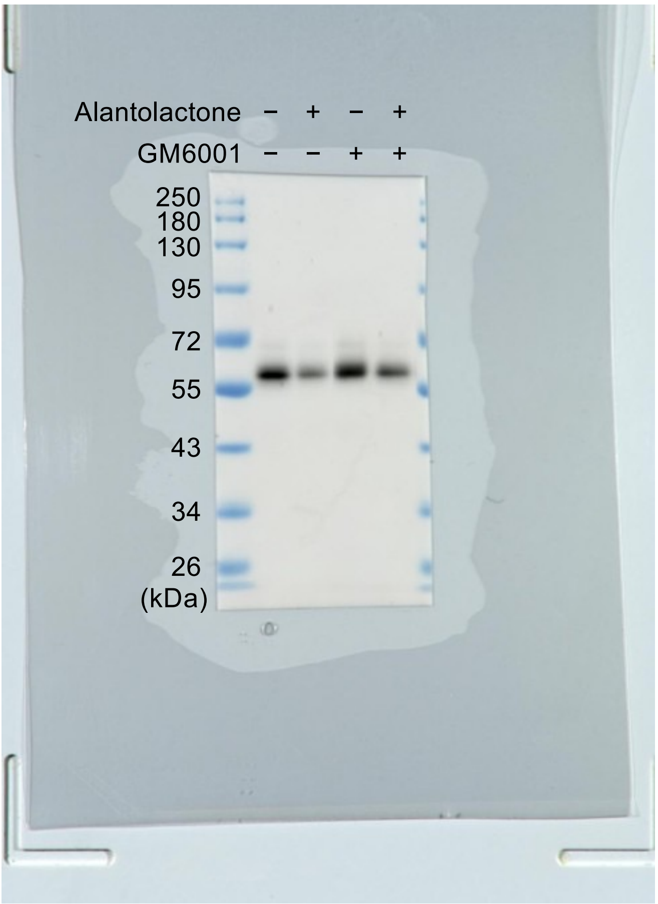

WB: TNF-R1

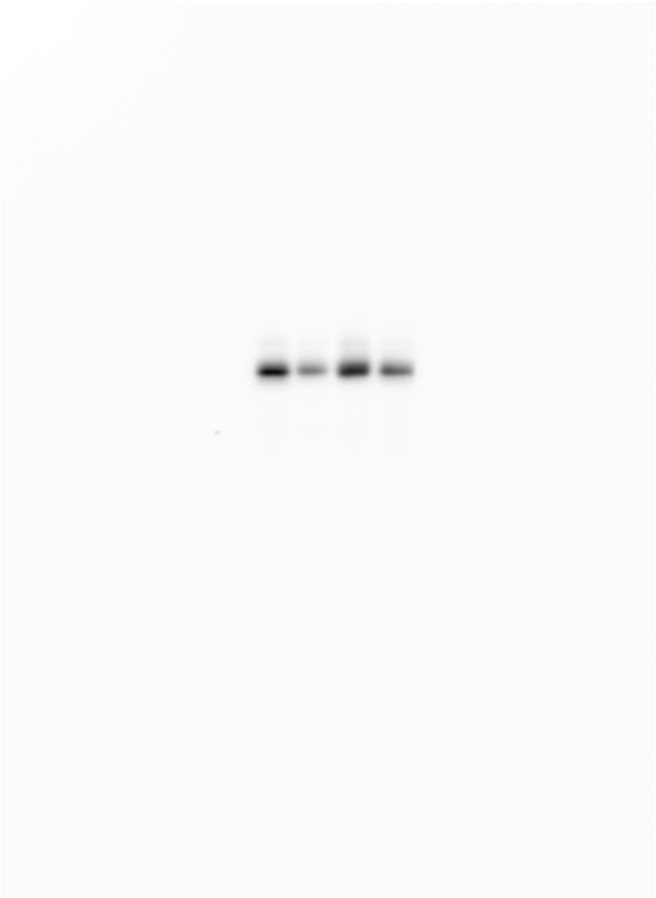

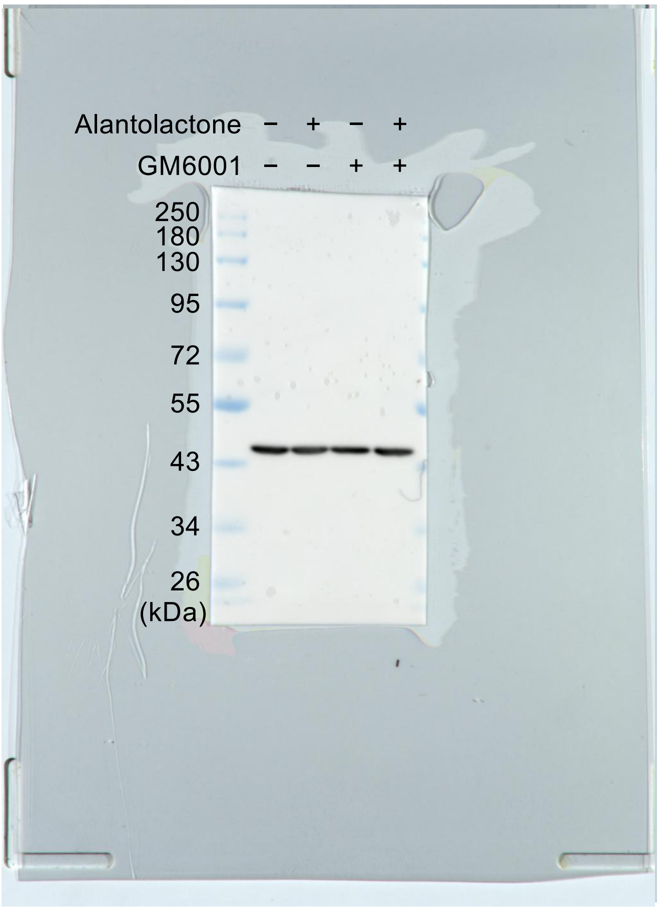

WB: β-Actin (reprobed)

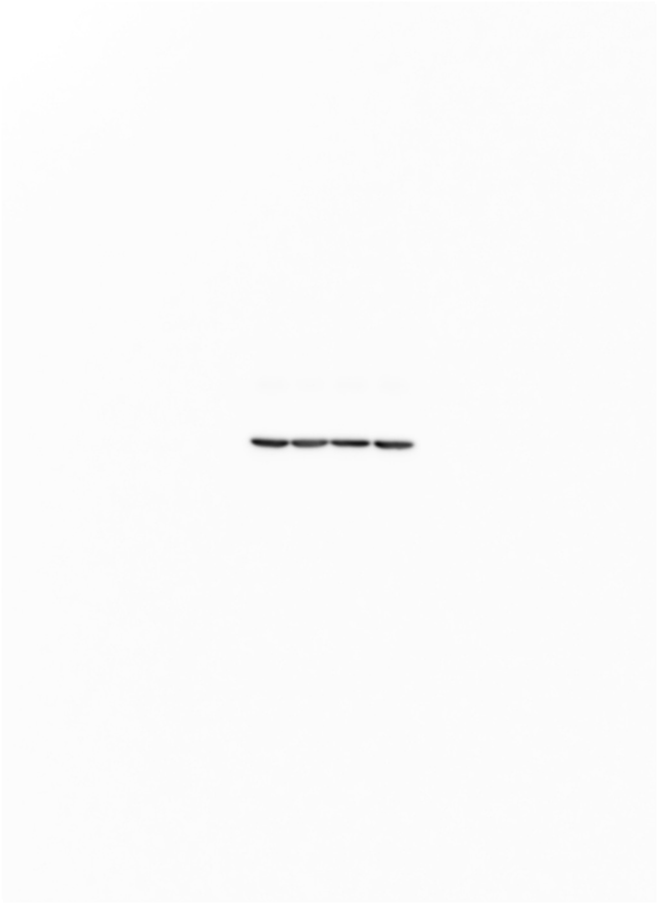

Figure S17: Original blots (2) in Figure 3C

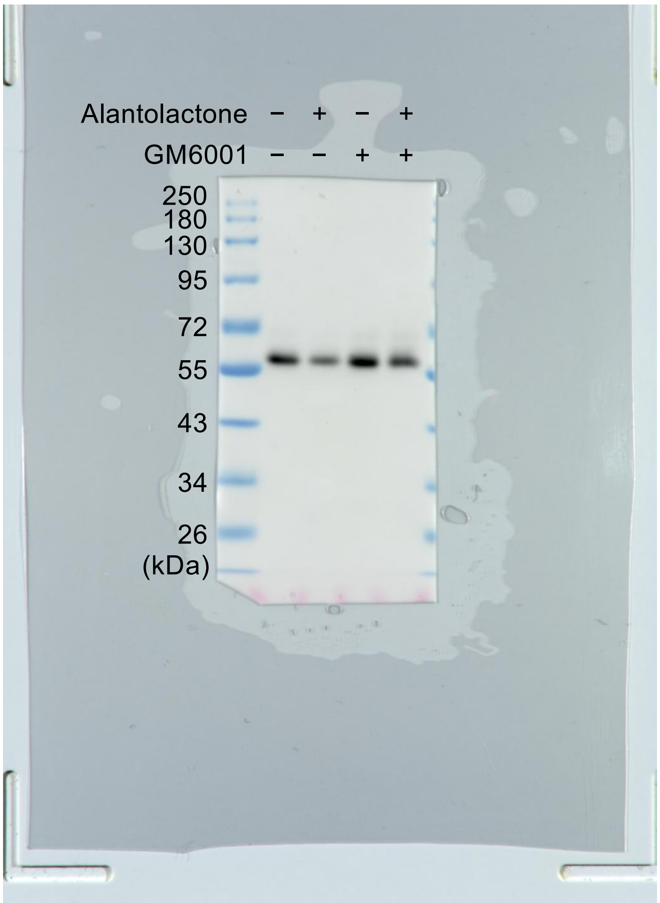

WB: TNF-R1

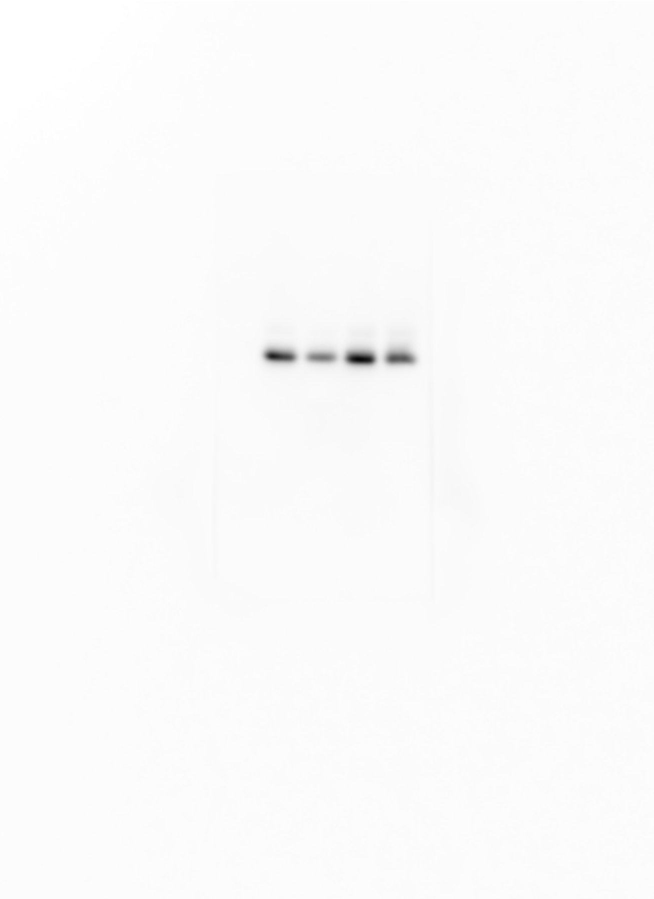

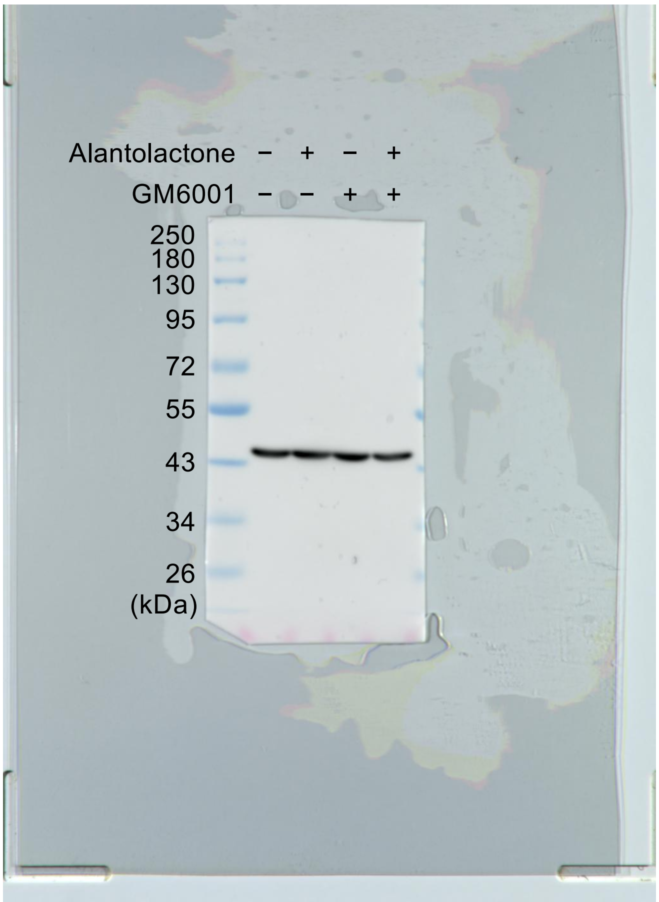

WB: β-Actin (reprobed)

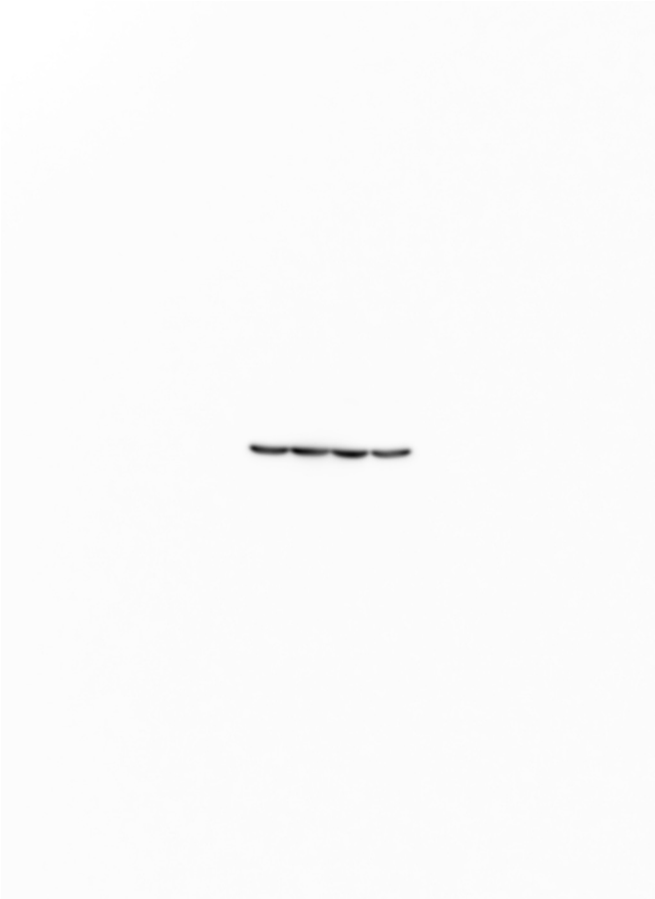

Figure S18: Original blots (3) in Figure 3C

WB: TNF-R1

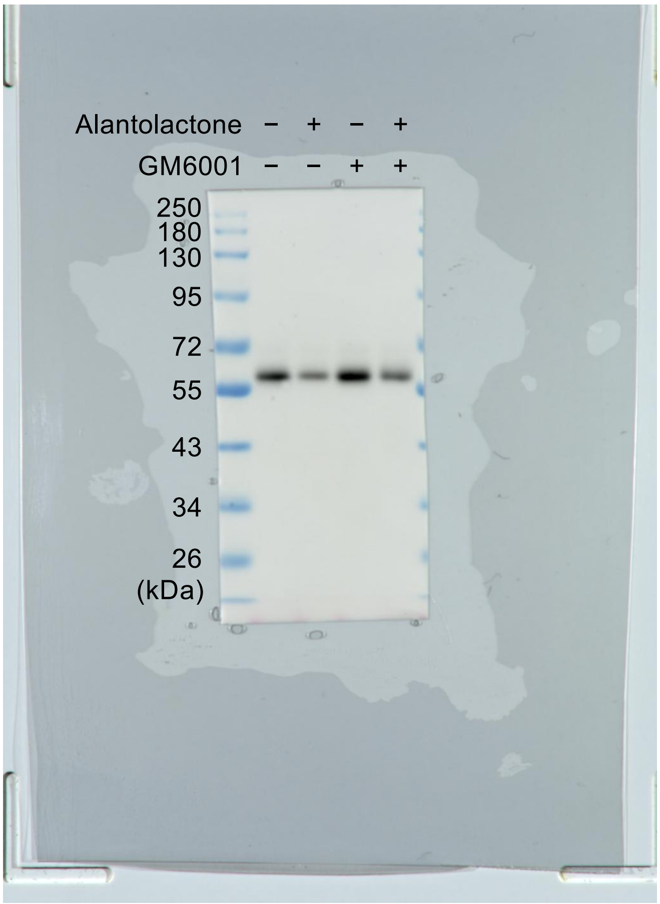

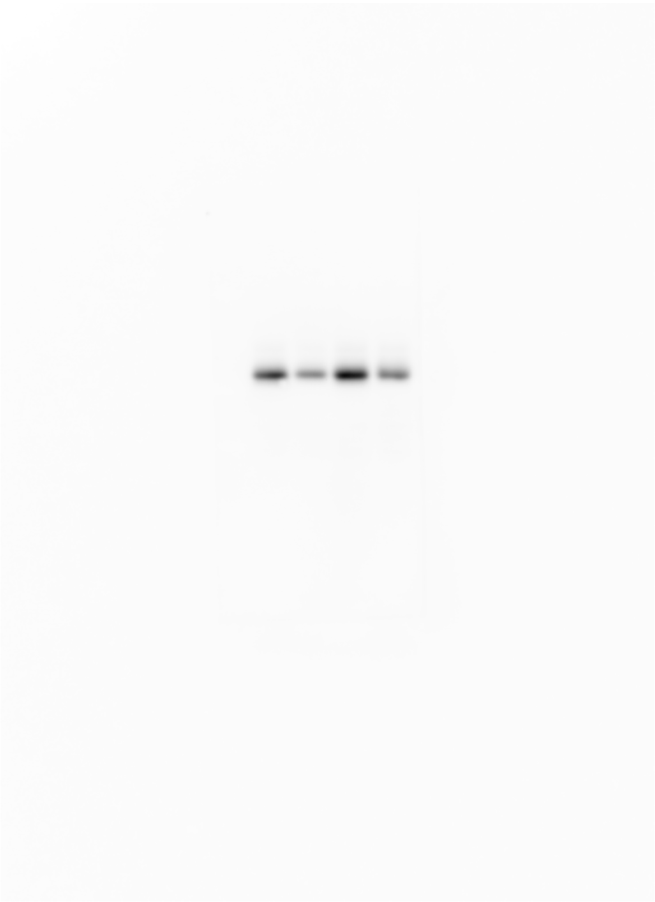

WB: β-Actin (reprobed)

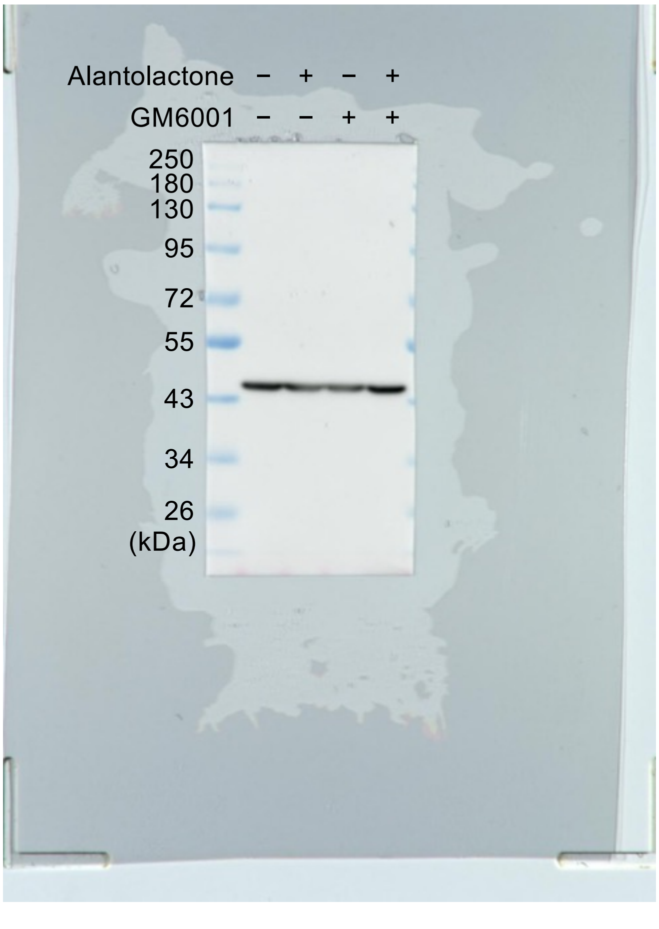

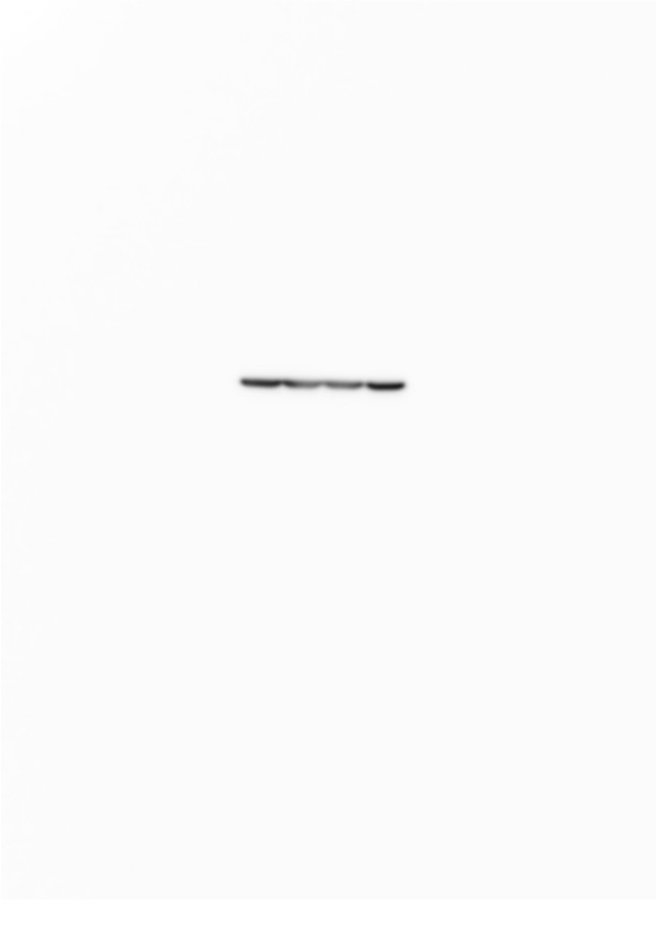

Figure S19: Original blots in Figure 4A (Medium)

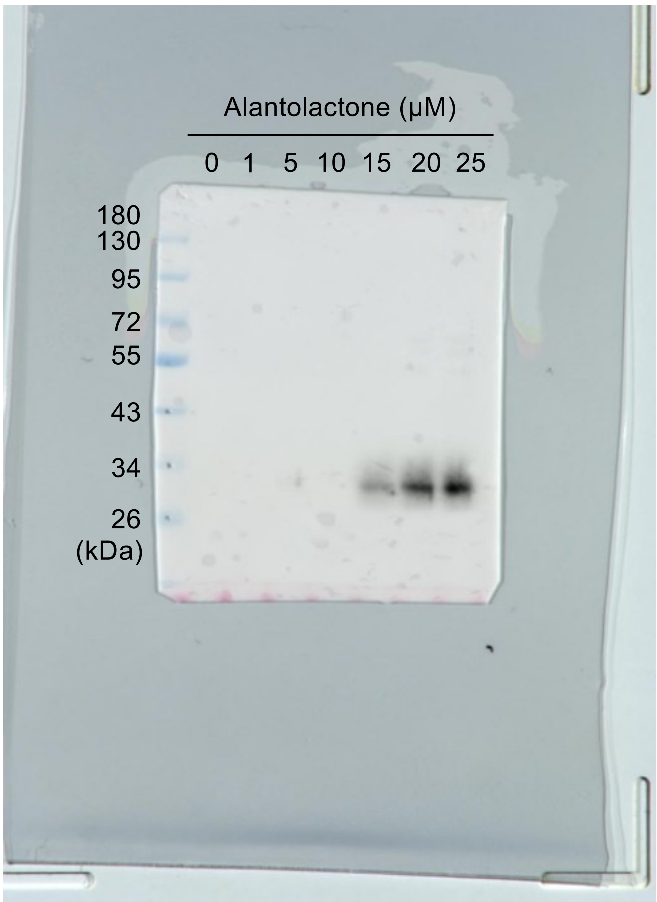

WB: TNF-R1

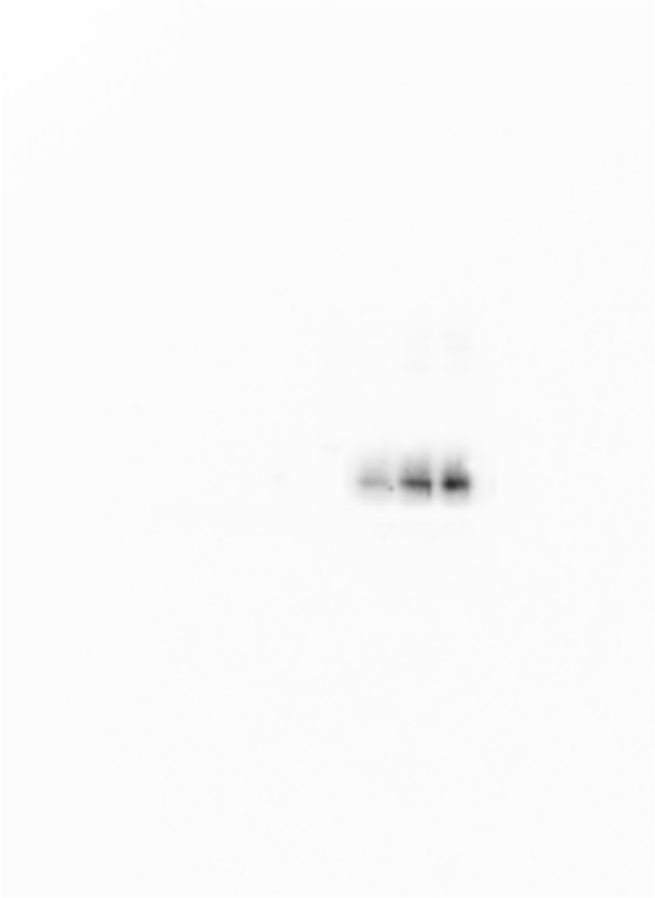

Figure S20: Original blots in Figure 4A (Cell lysate)

WB: TNF-R1

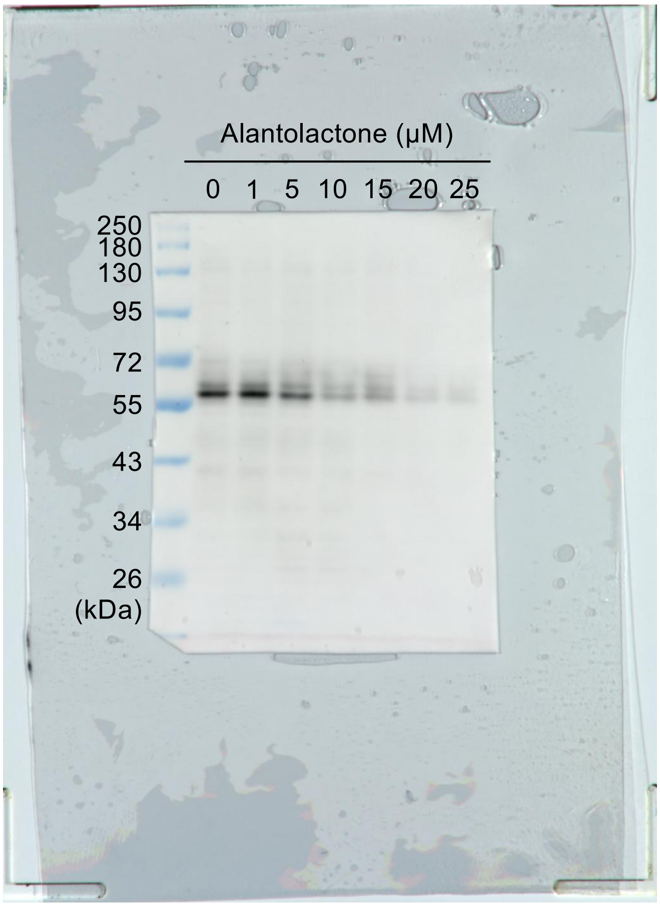

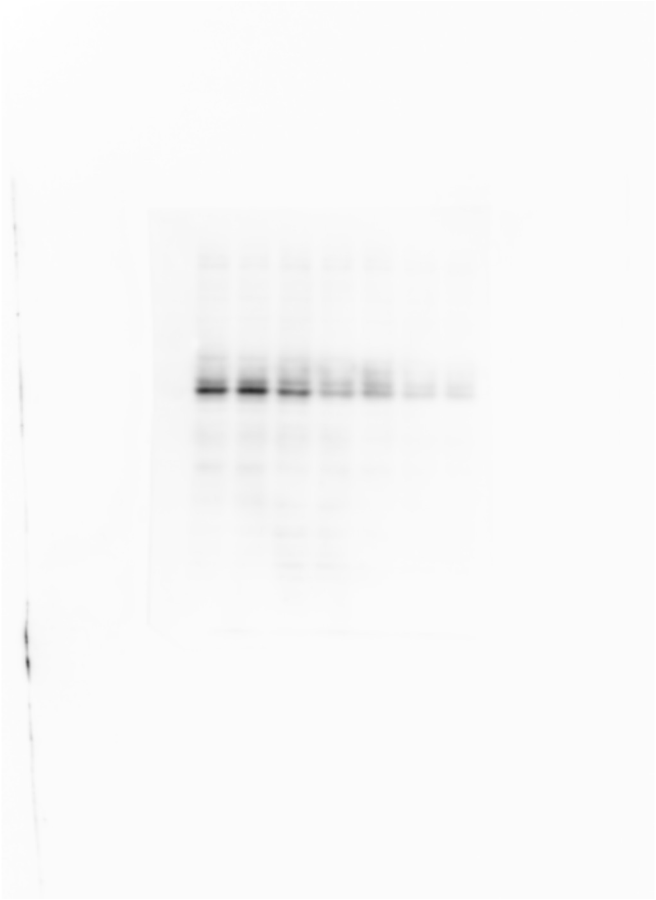

WB: β-Actin (reprobed)

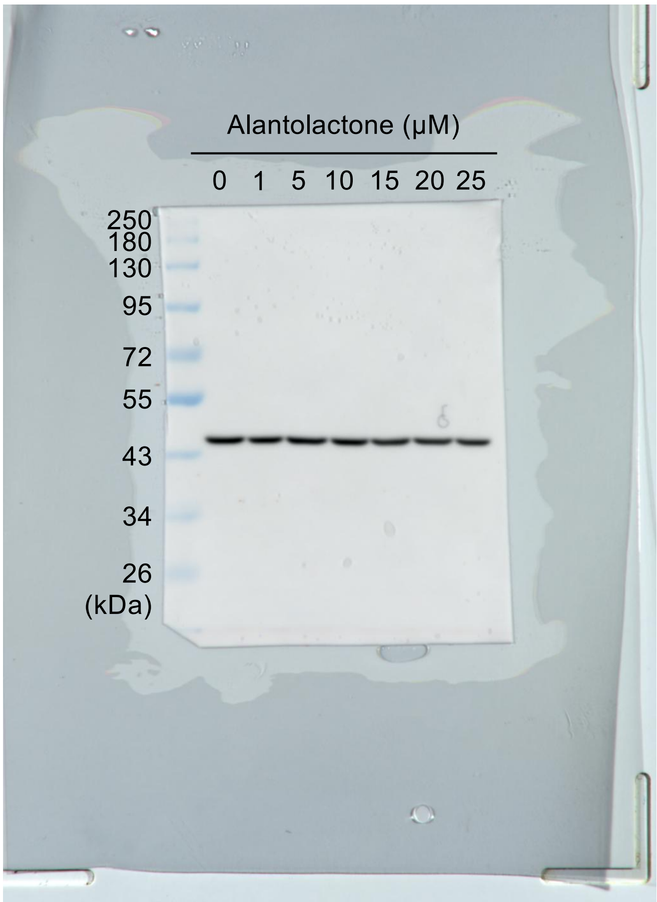

Figure S21: Original blots (1) in Figure 4B

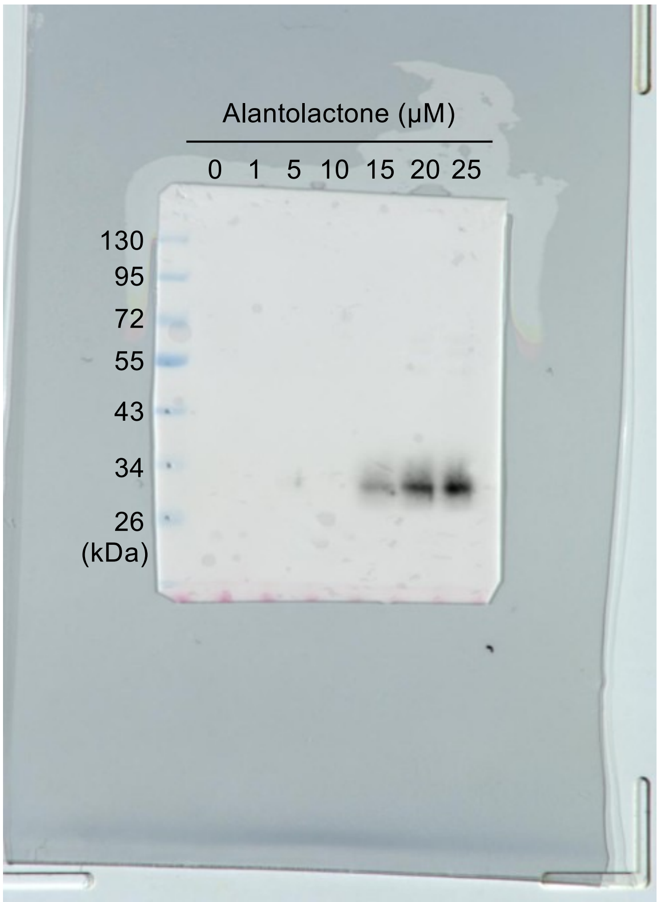

WB: TNF-R1

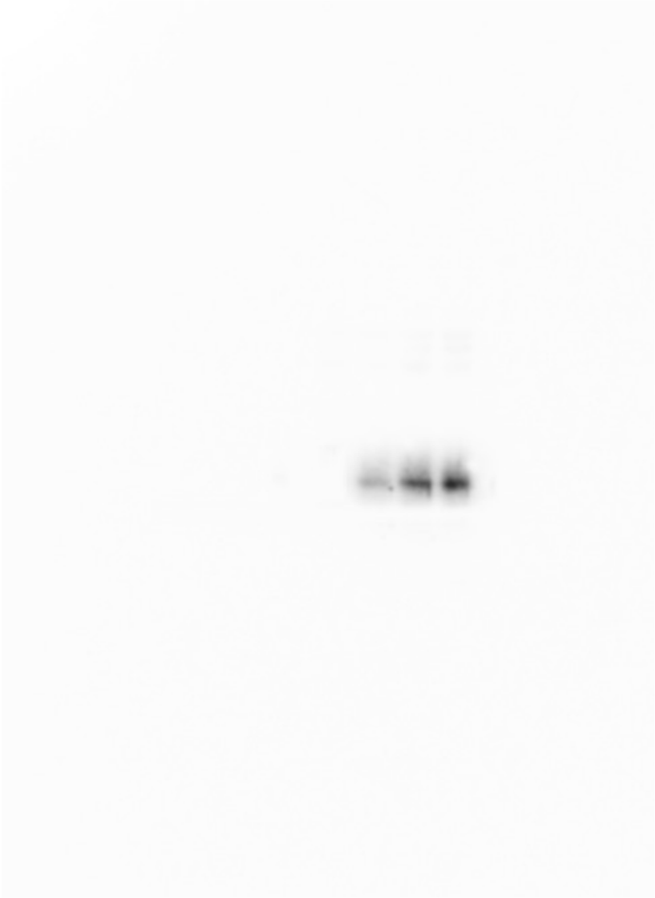

Figure S22: Original blots (2) in Figure 4B

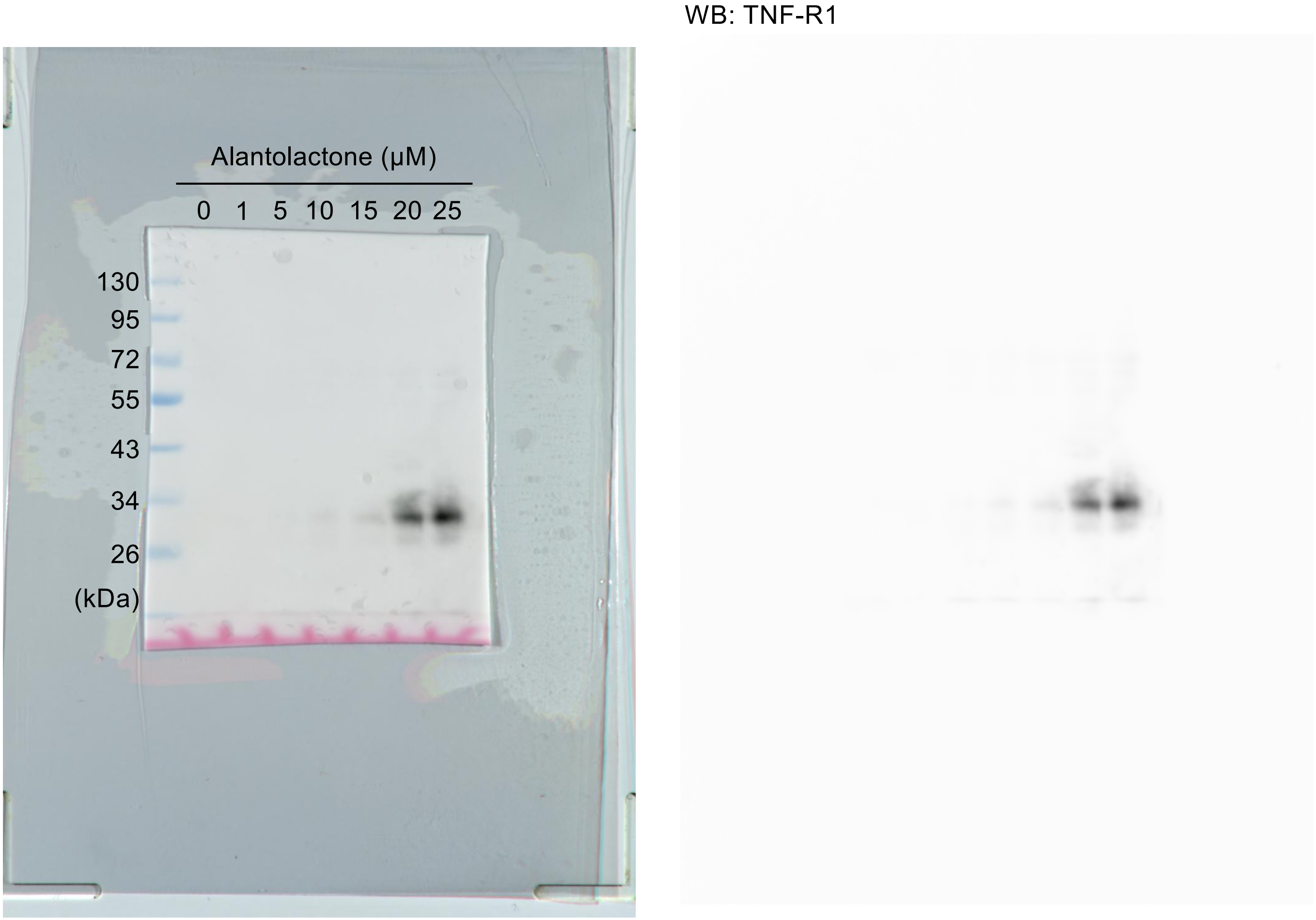

Figure S23: Original blots (3) in Figure 4B

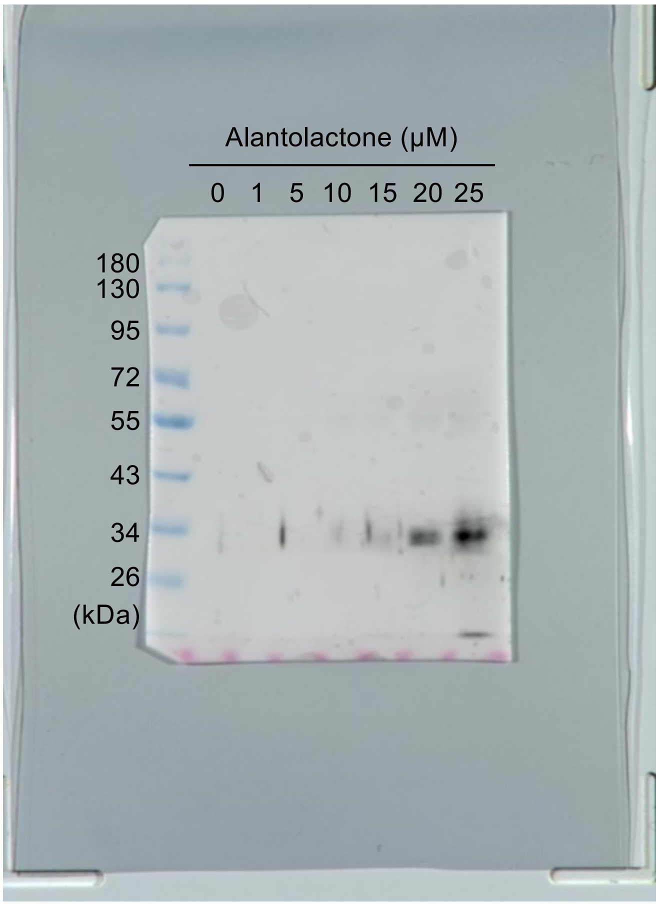

WB: TNF-R1

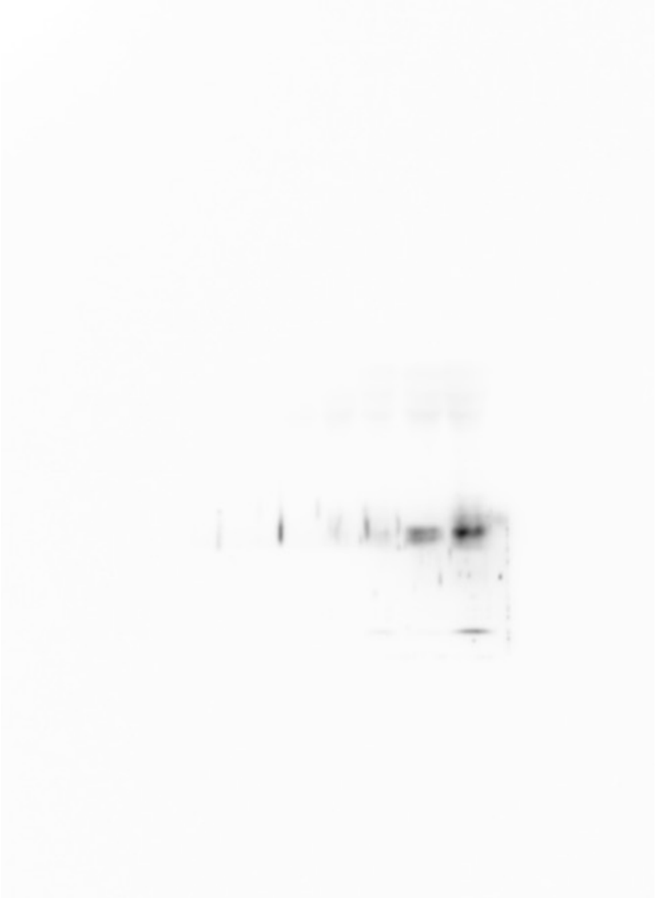

Figure S24: Original blots (1) in Figure 4C

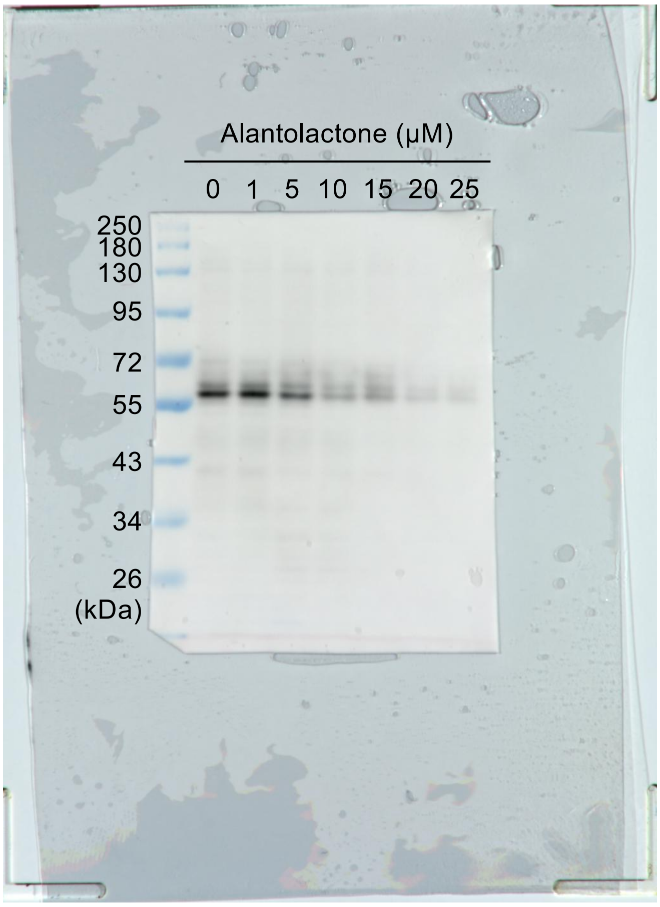

WB: TNF-R1

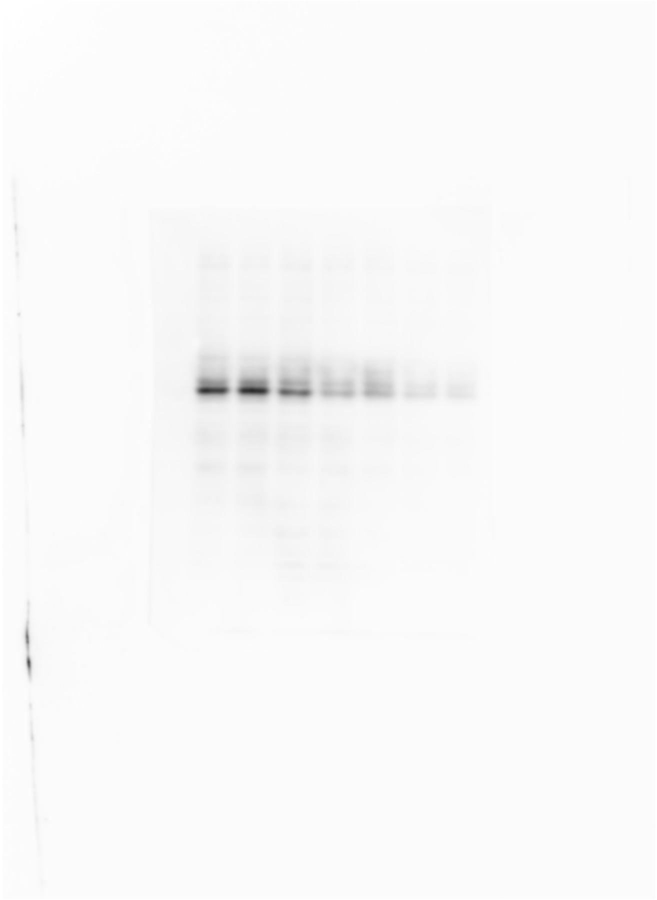

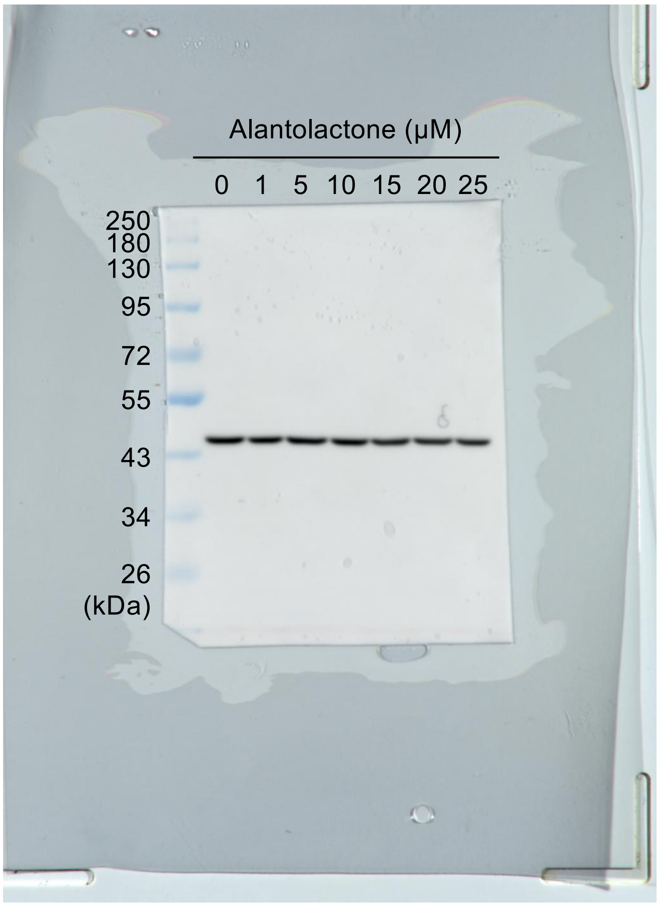

WB: β-Actin (reprobed)

Figure S25: Original blots (2) in Figure 4C

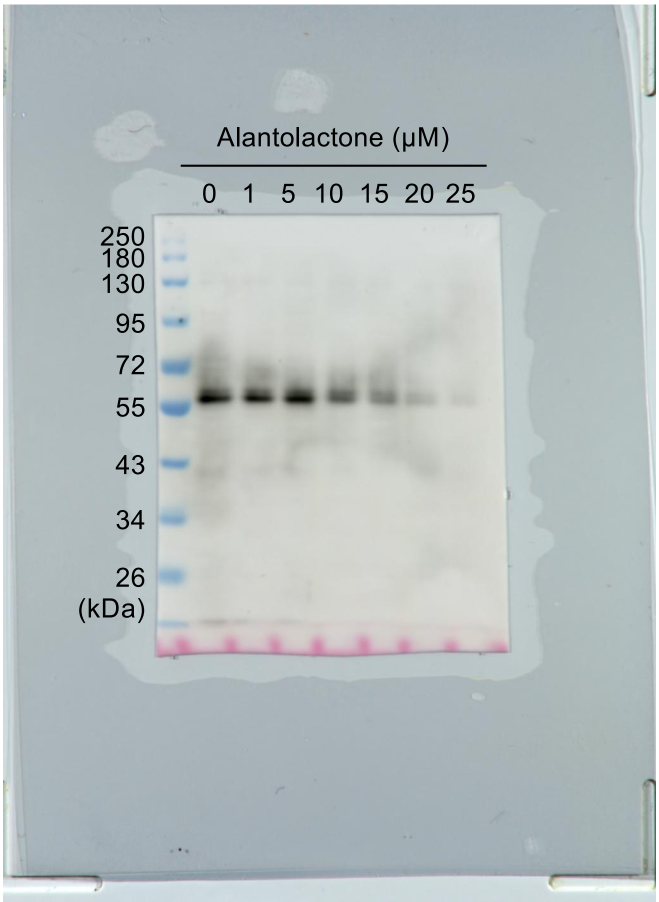

WB: TNF-R1

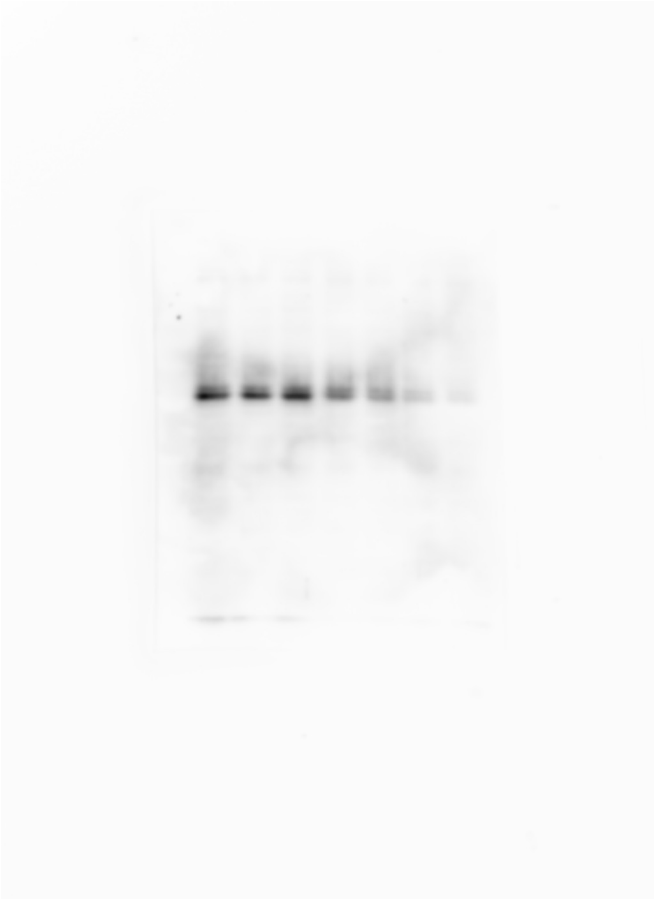

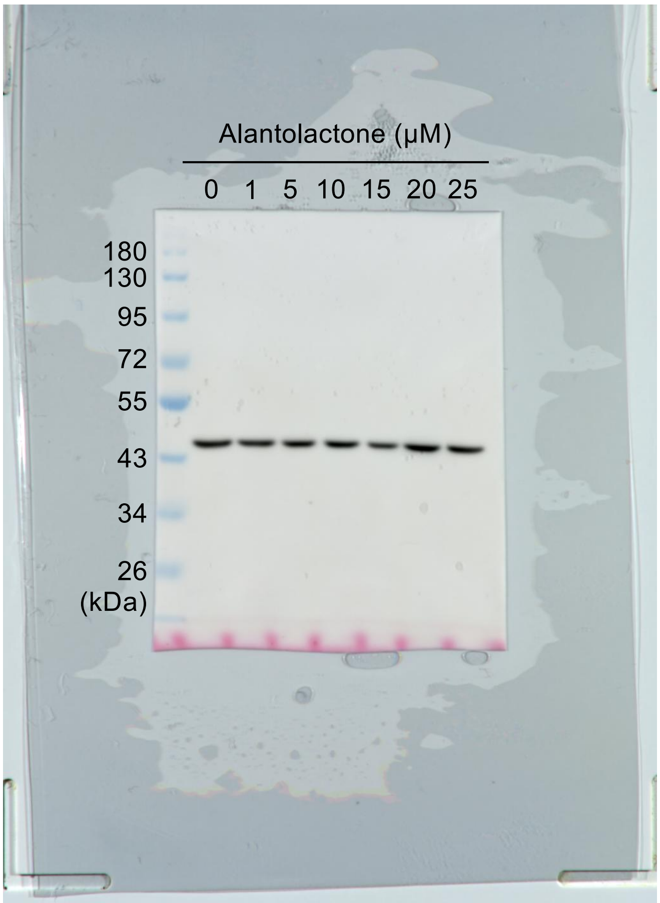

WB: β-Actin (reprobed)

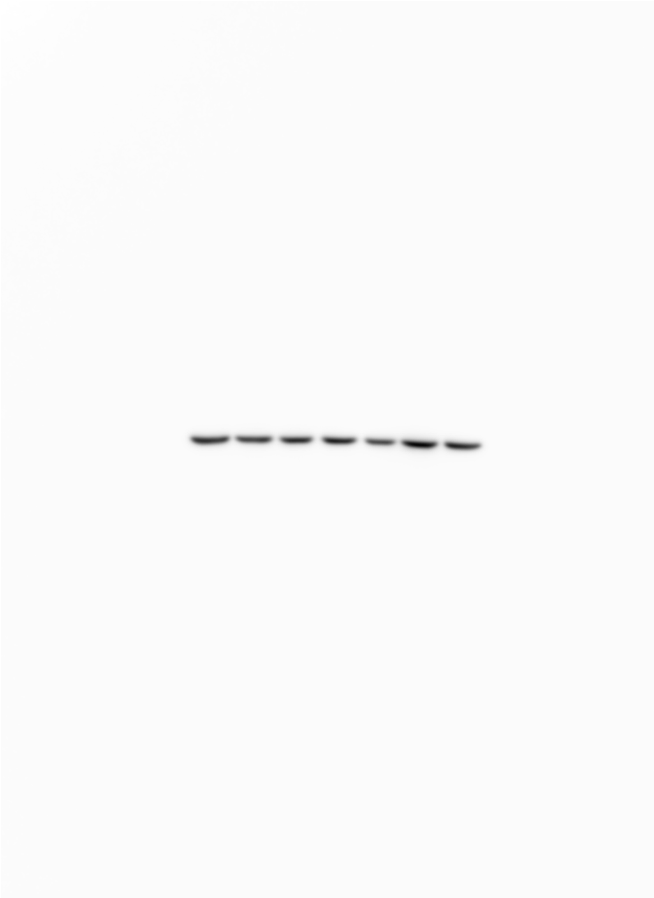

Figure S26: Original blots (3) in Figure 4C

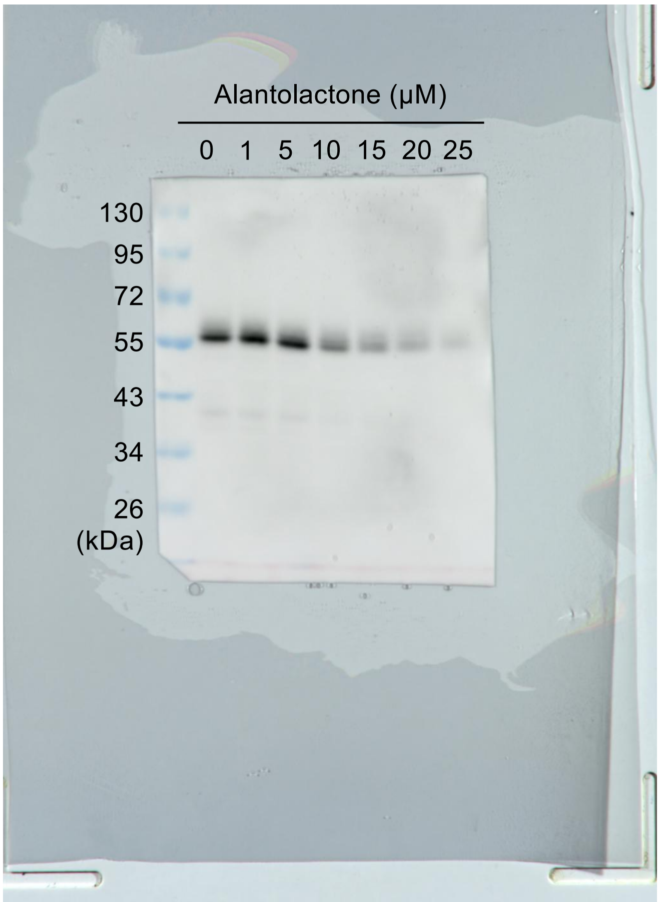

WB: TNF-R1

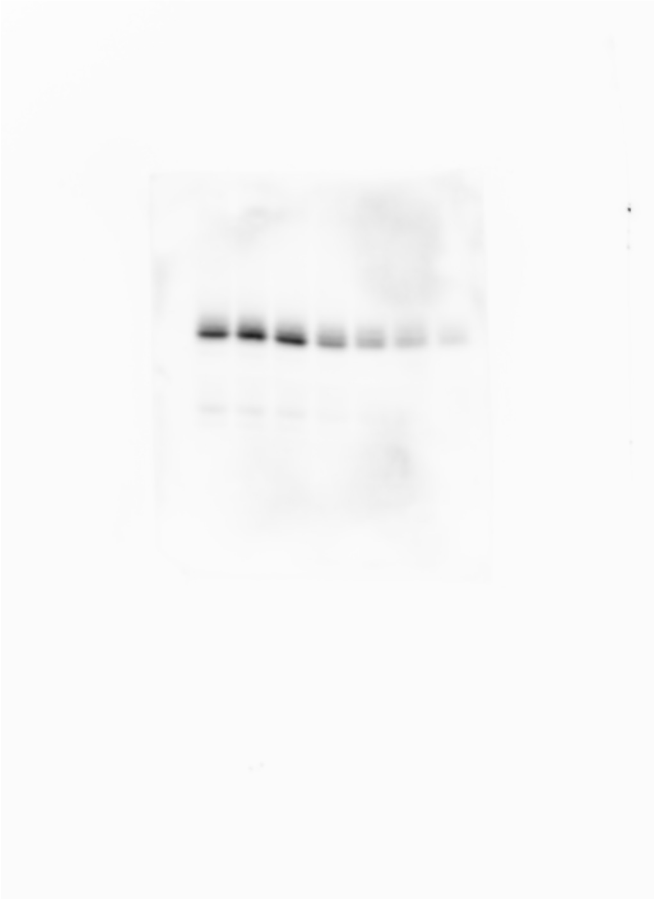

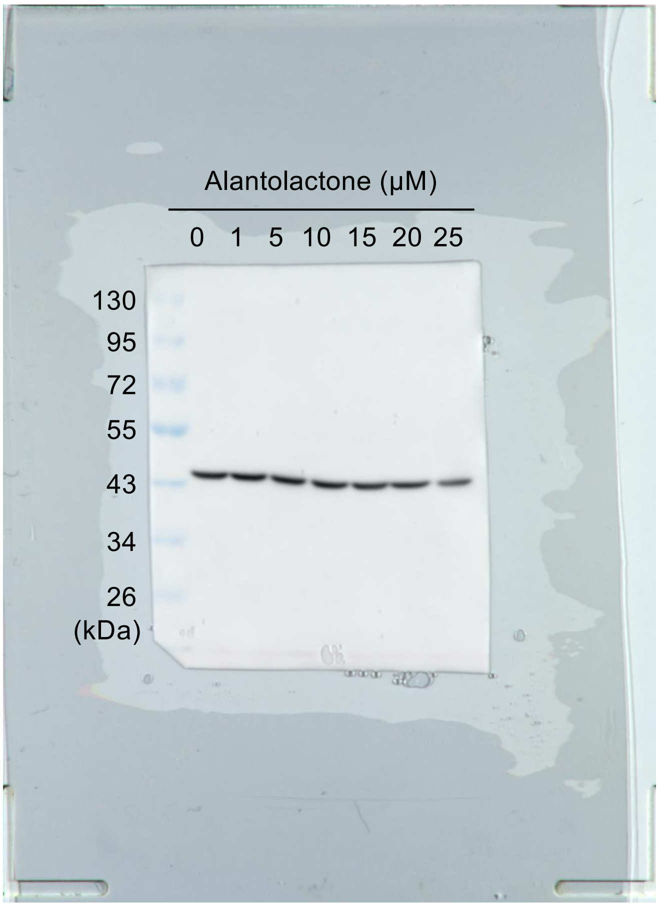

WB: β-Actin (reprobed)

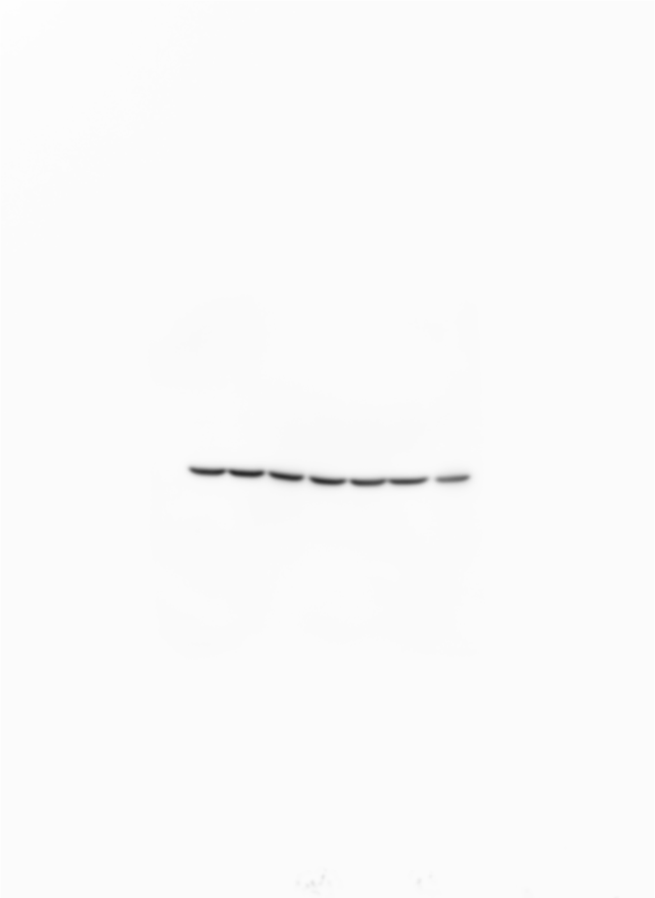

Figure S27: Original blots in Figure 4D (Medium)

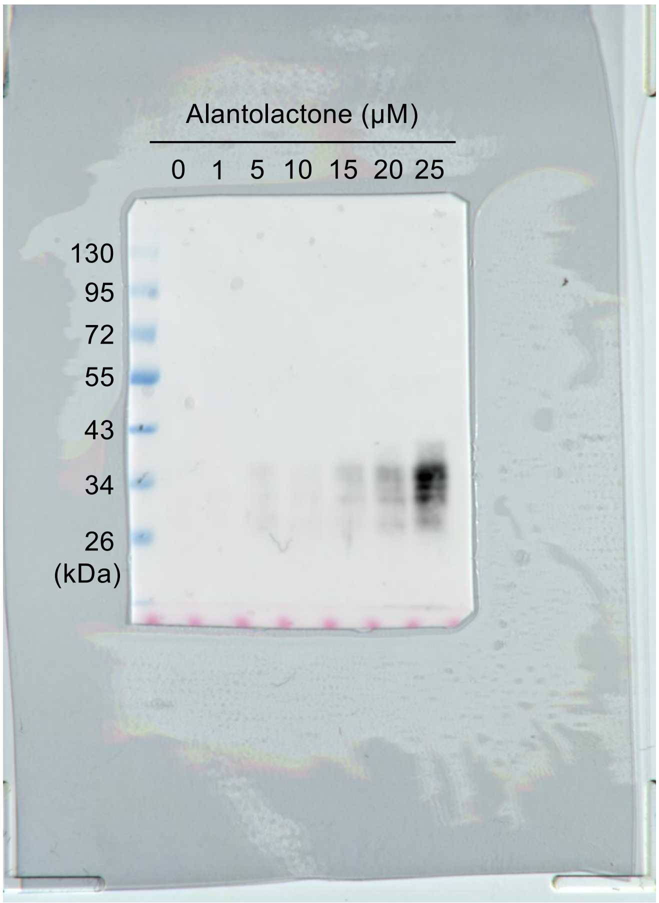

WB: TNF-R1

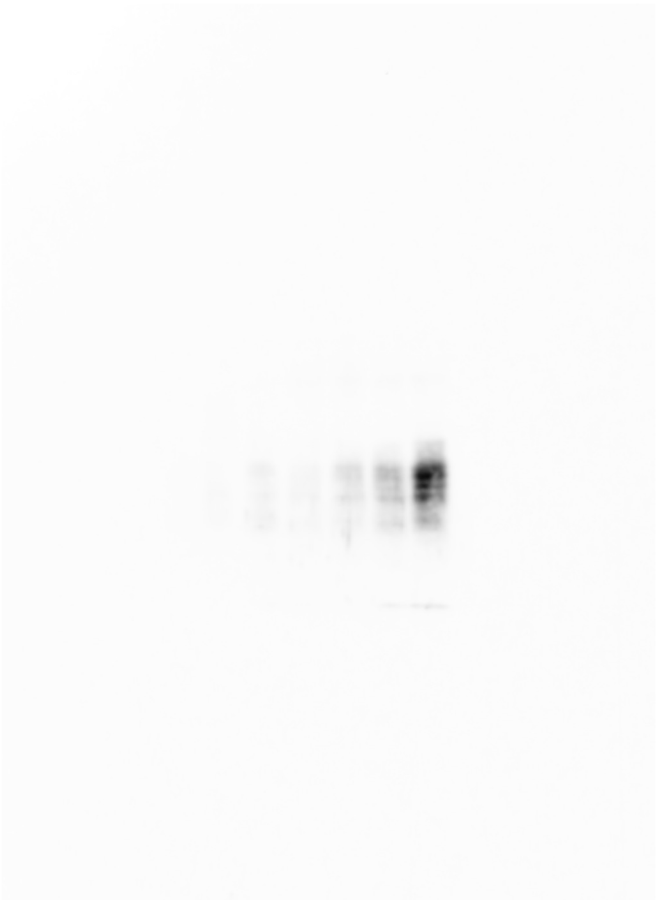

Figure S28: Original blots in Figure 4D (Cell lysate)

WB: TNF-R1

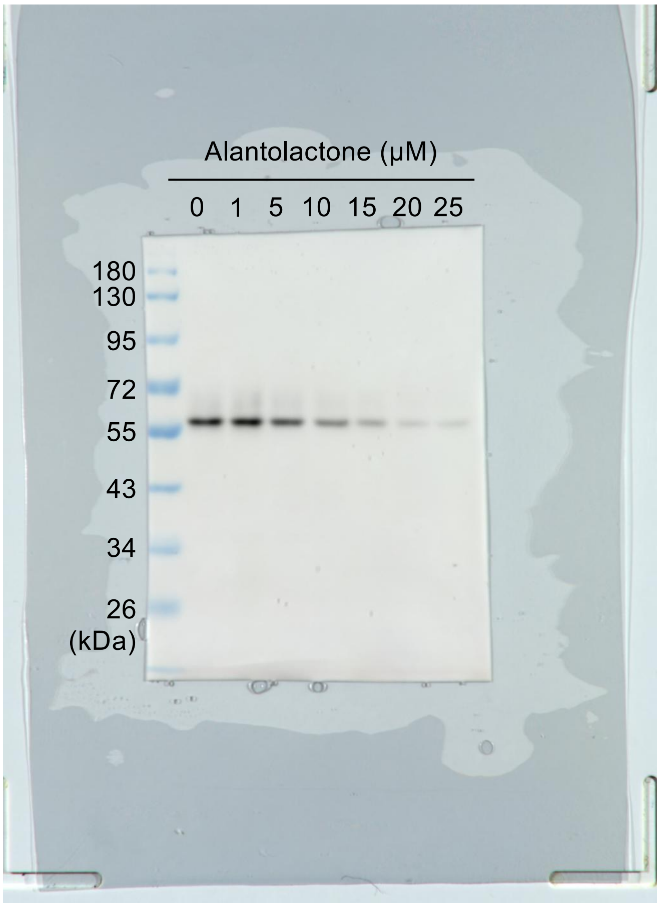

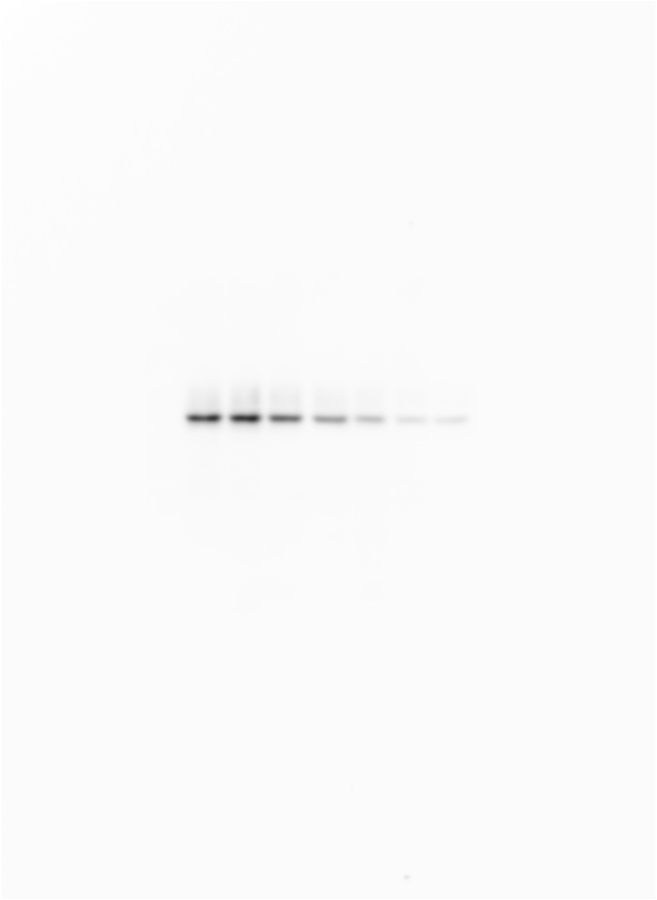

WB: β-Actin (reprobed)

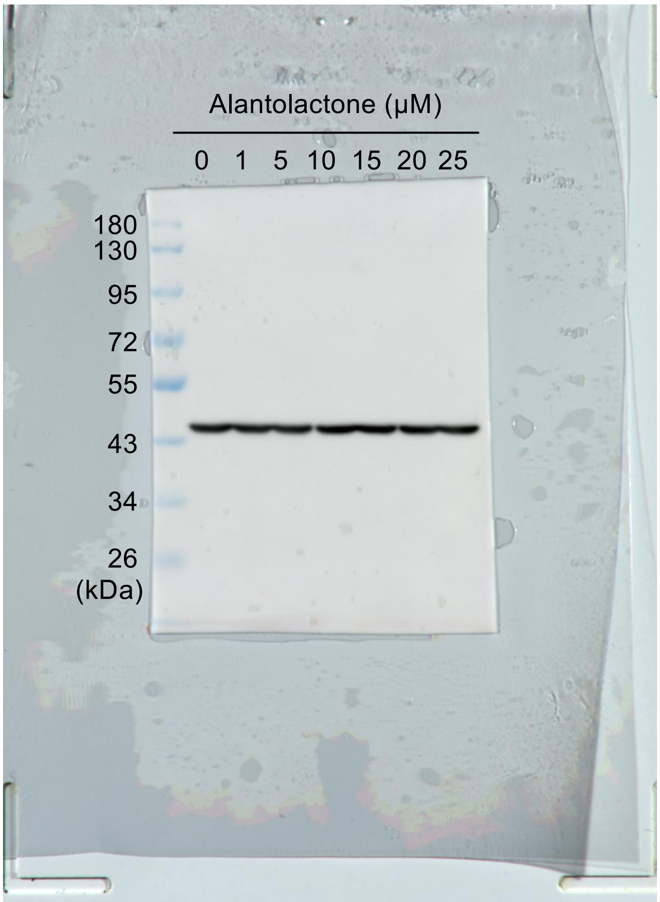

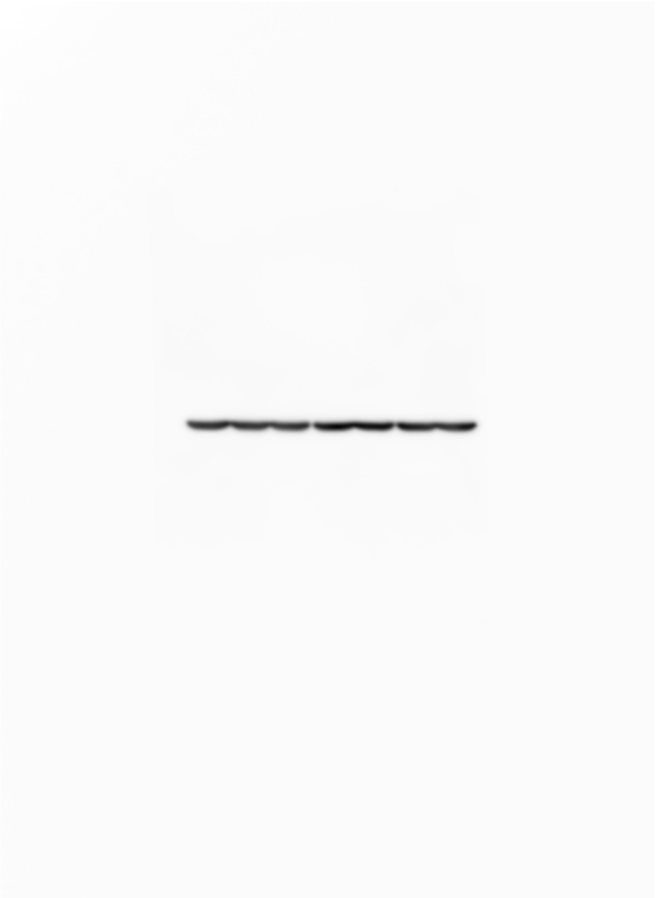

Figure S29: Original blots (1) in Figure 4E

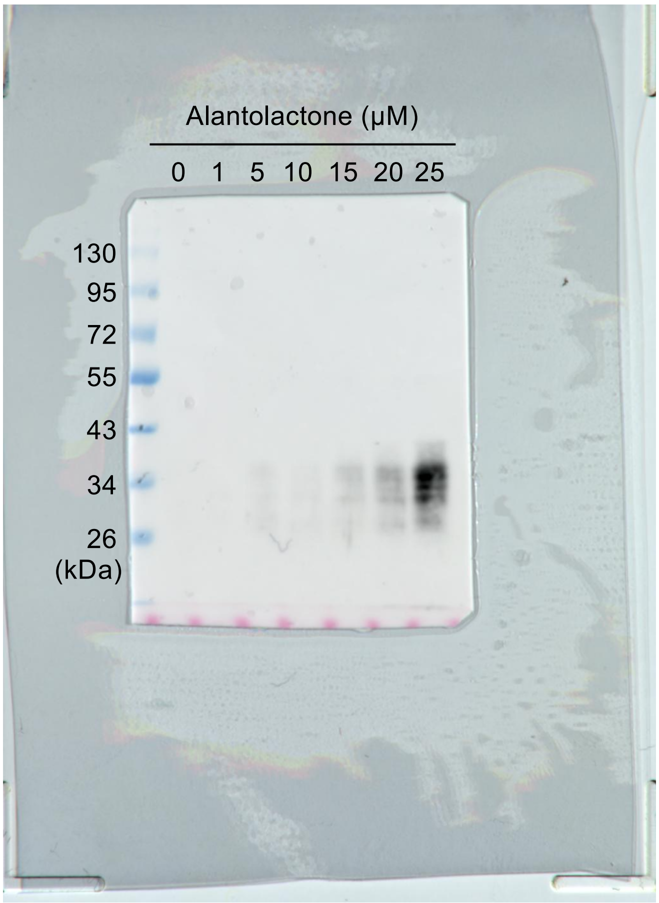

WB: TNF-R1

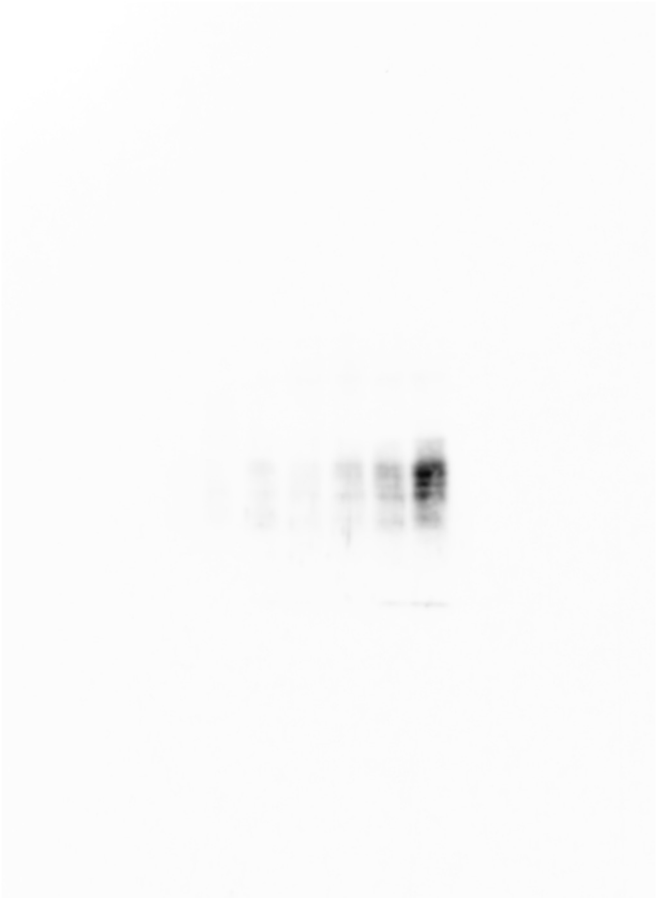

Figure S30: Original blots (2) in Figure 4E

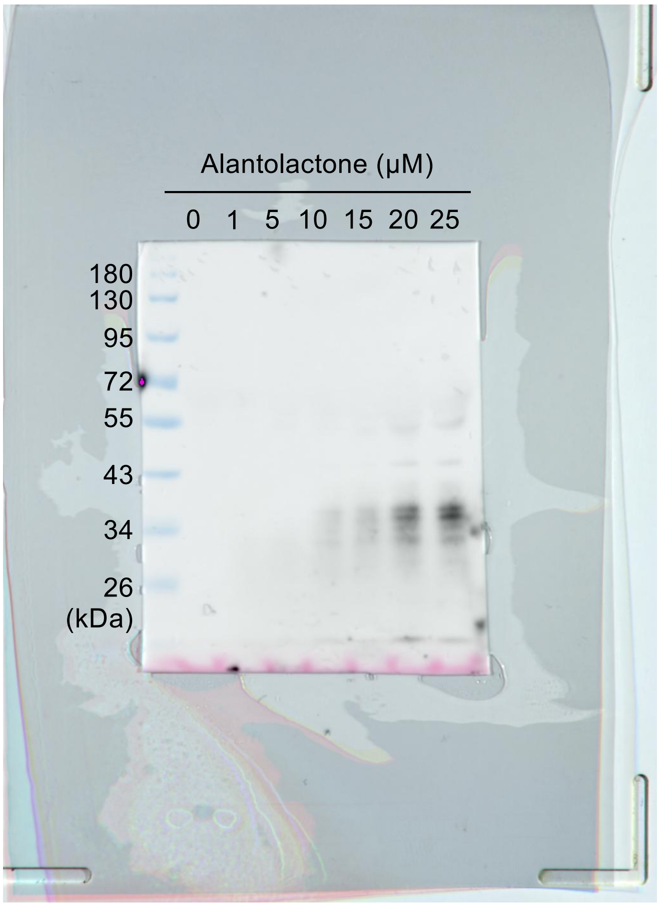

WB: TNF-R1

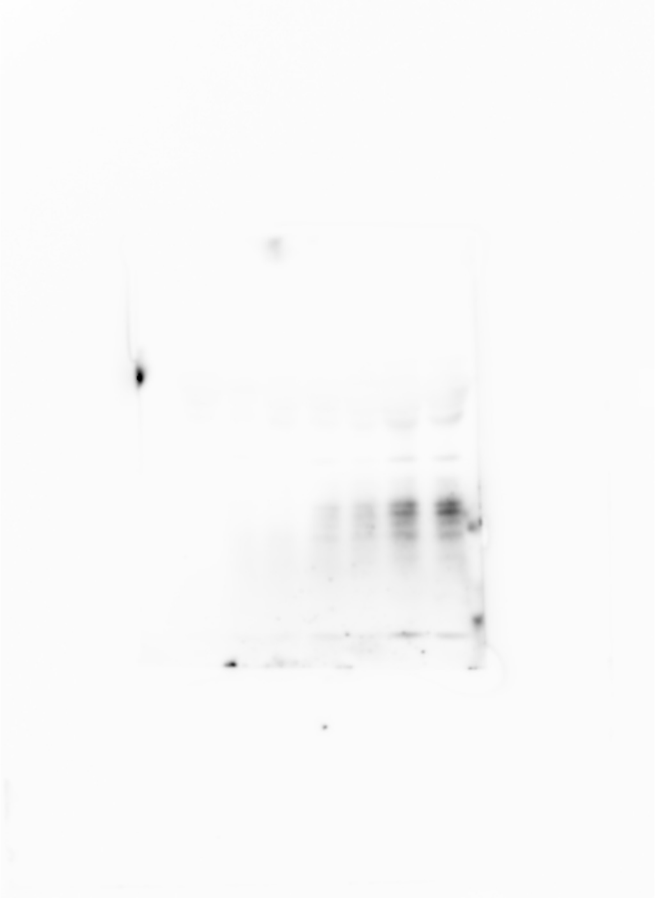

Figure S31: Original blots (3) in Figure 4E

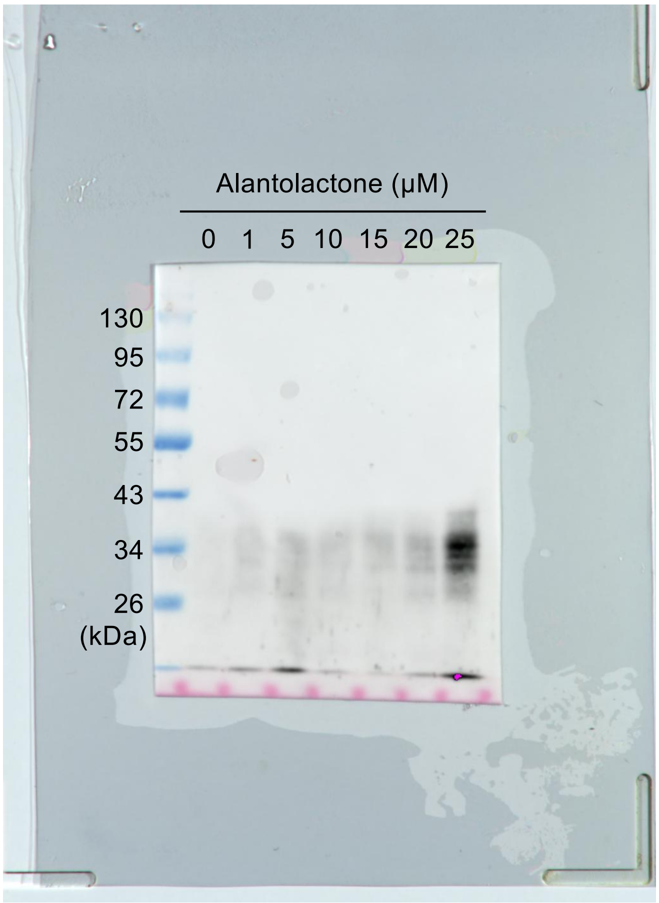

WB: TNF-R1

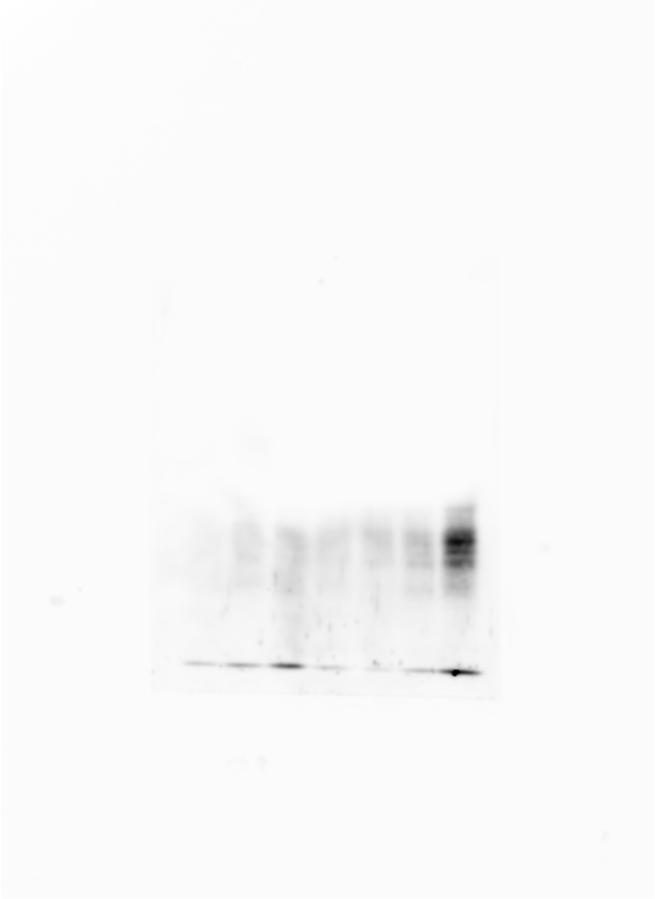

Figure S32: Original blots (1) in Figure 4F

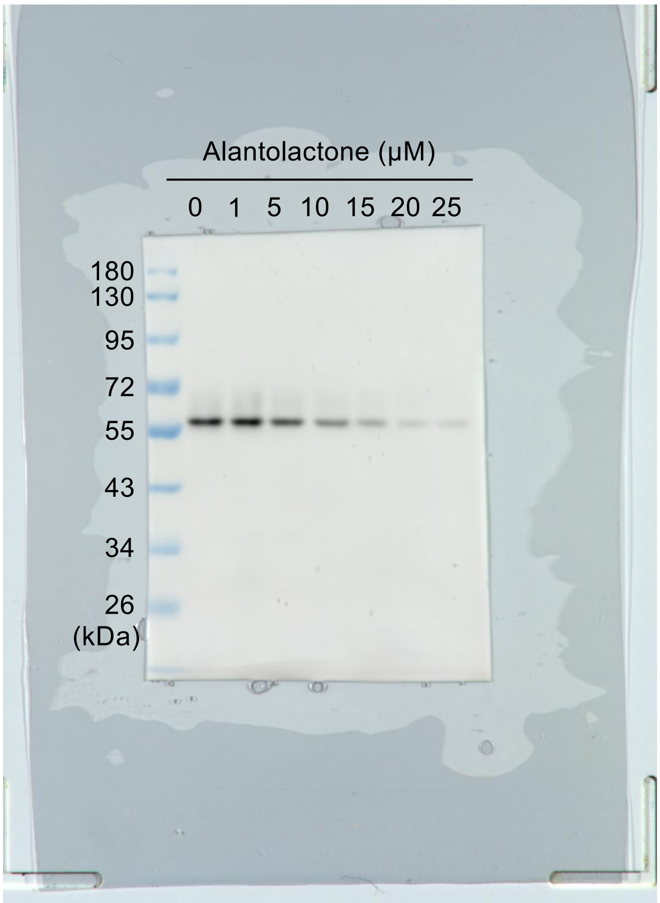

WB: TNF-R1

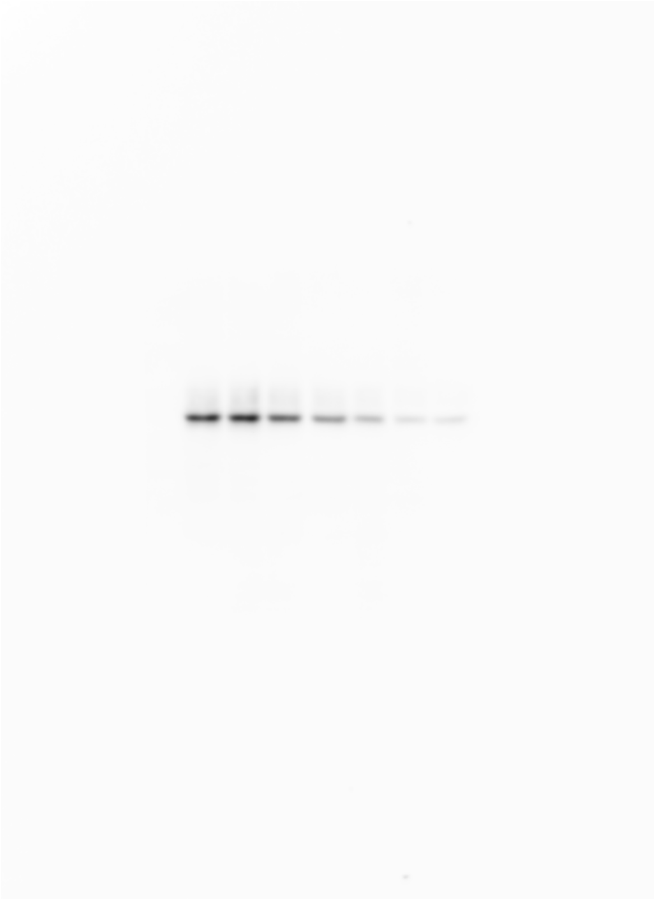

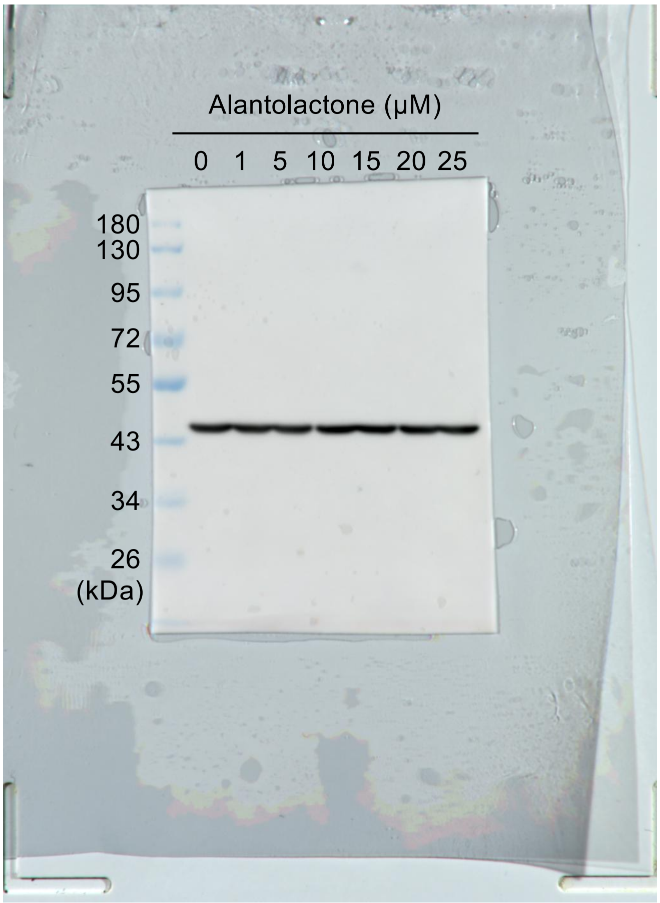

WB: β-Actin (reprobed)

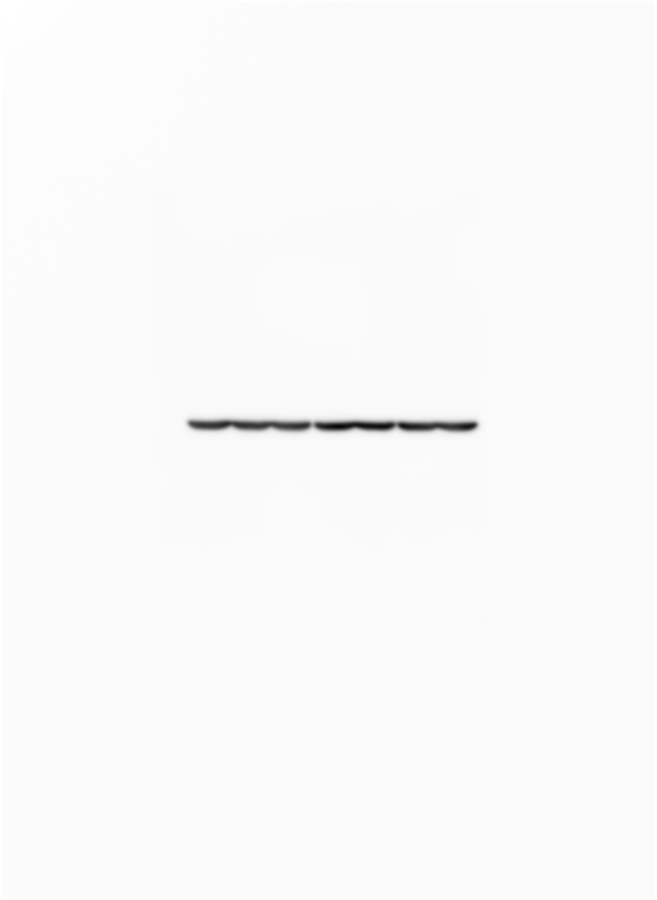

Figure S33: Original blots (2) in Figure 4F

WB: TNF-R1

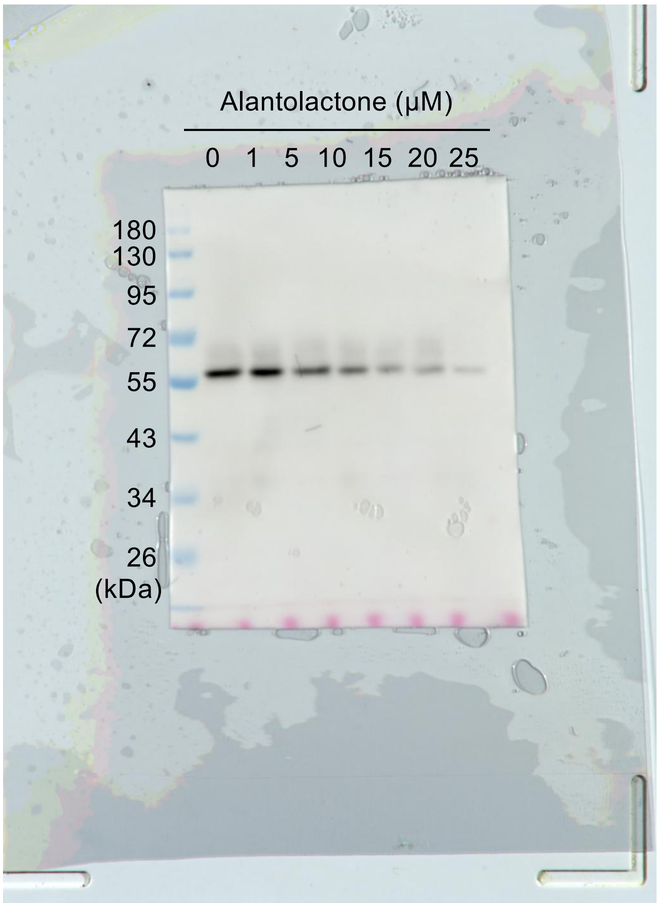

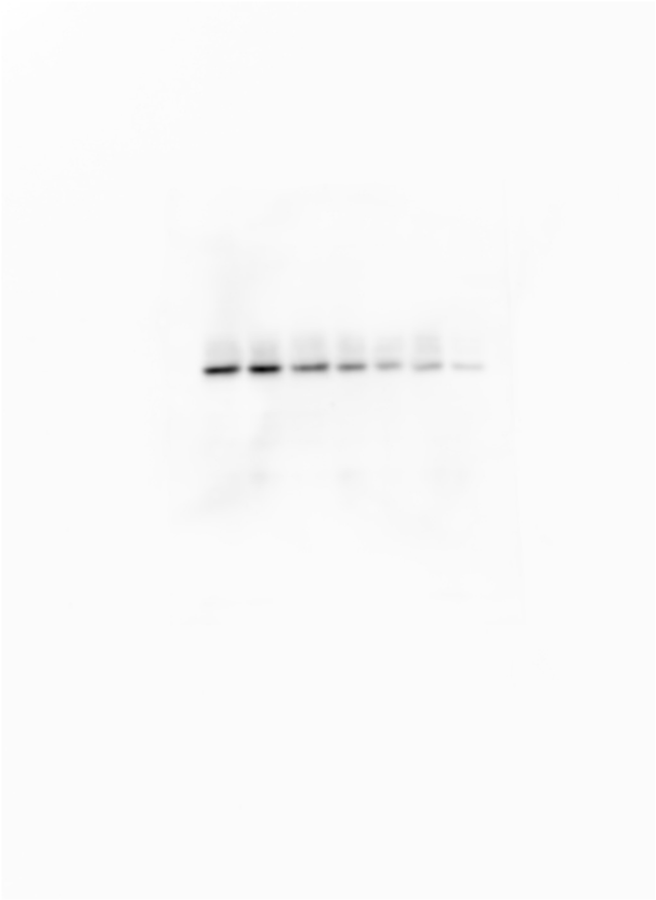

WB: β-Actin (reprobed)

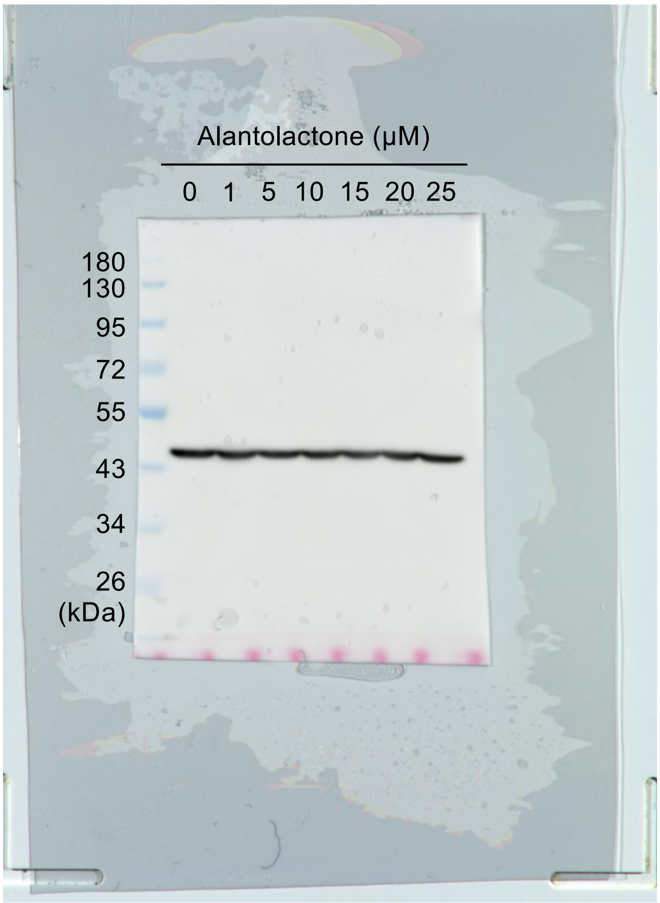

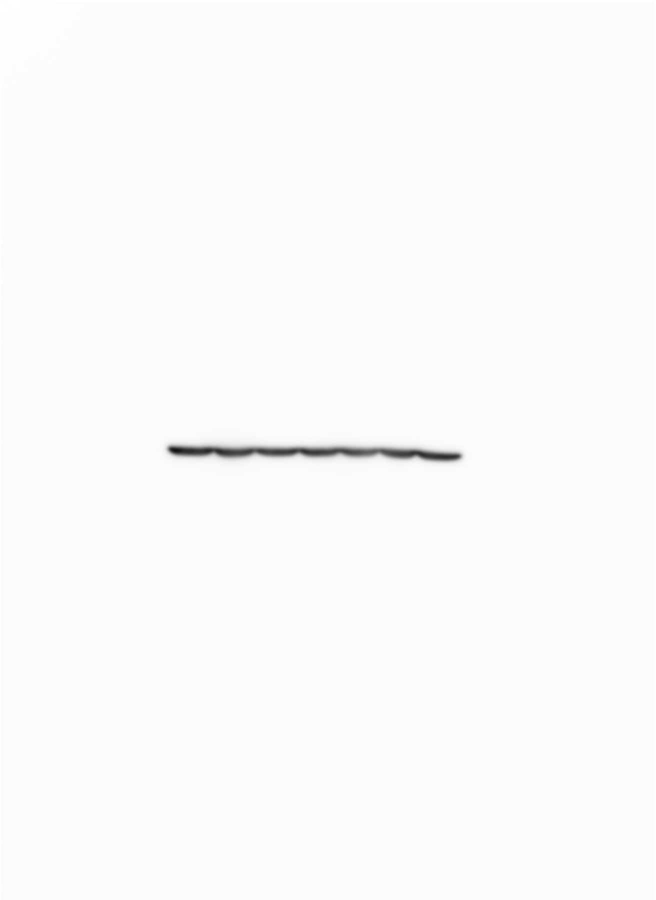

Figure S34: Original blots (3) in Figure 4F

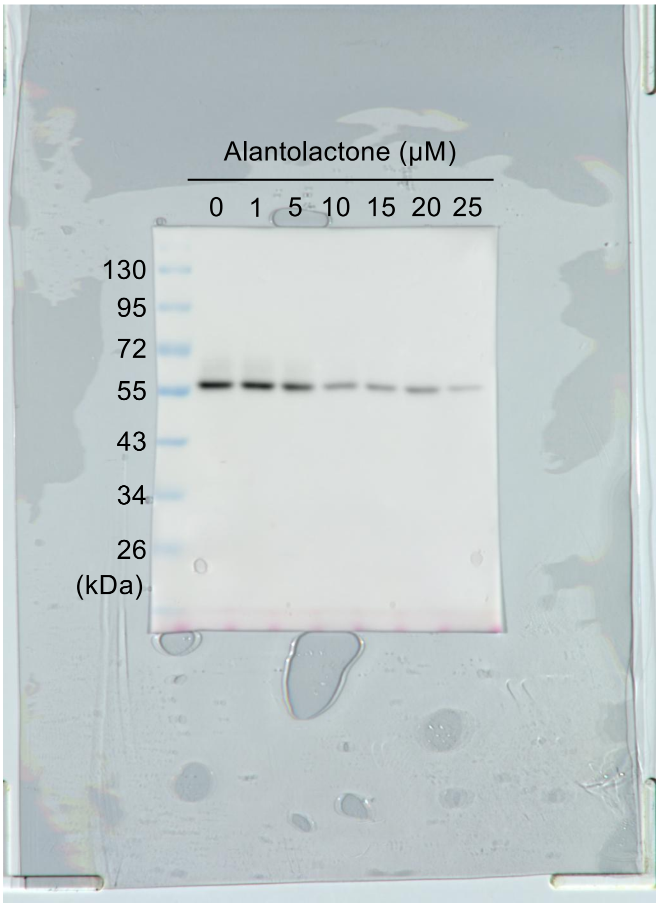

WB: TNF-R1

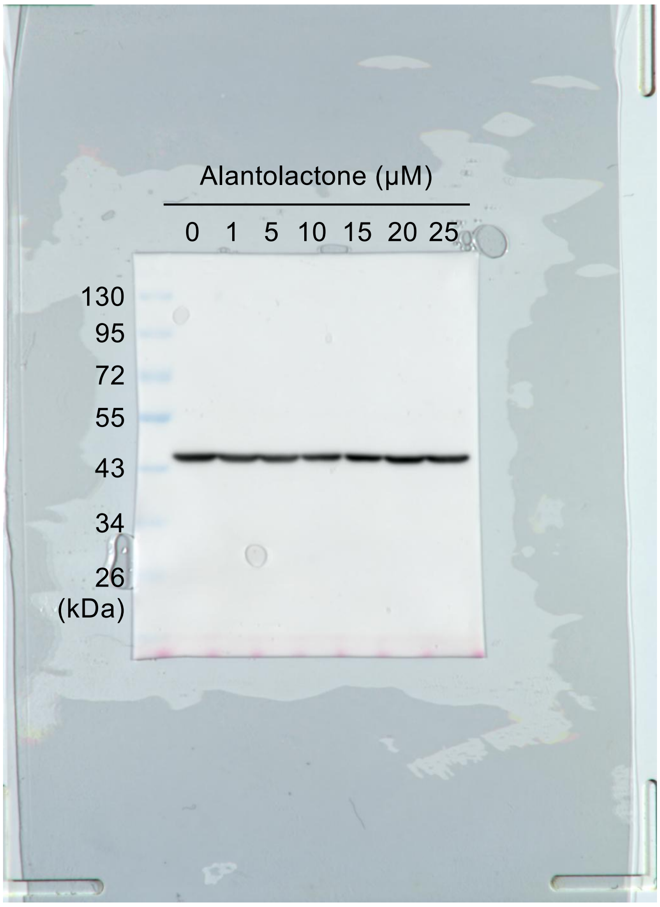

WB: β-Actin (reprobed)

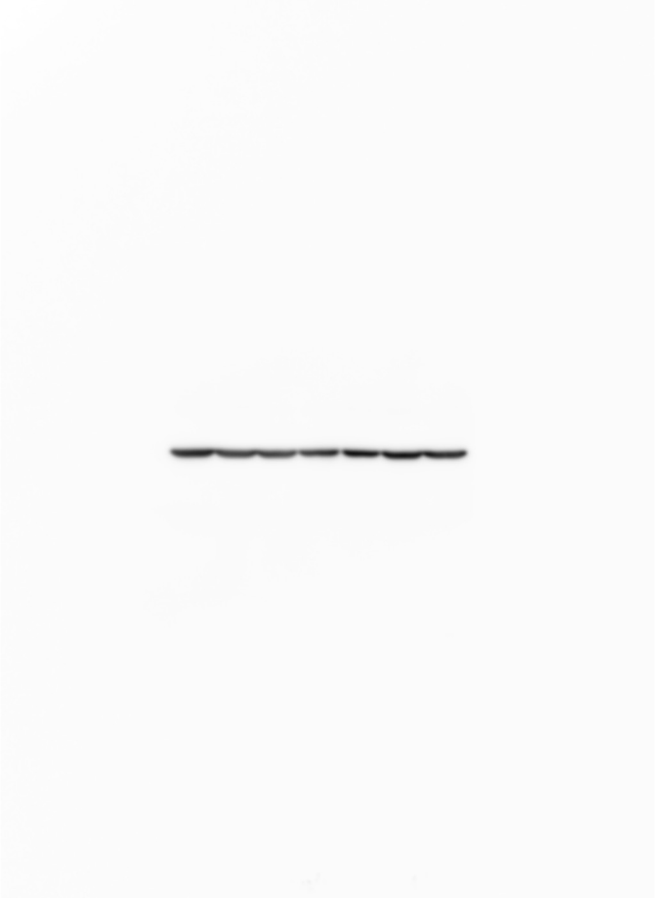

Figure S35: Original blots in Figure 5A (Medium)

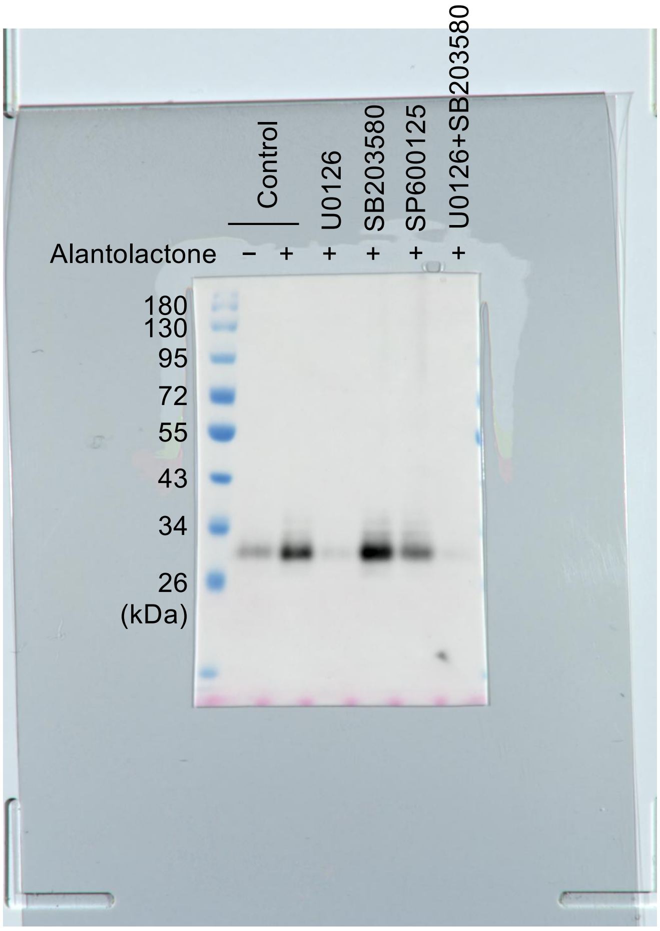

WB: TNF-R1

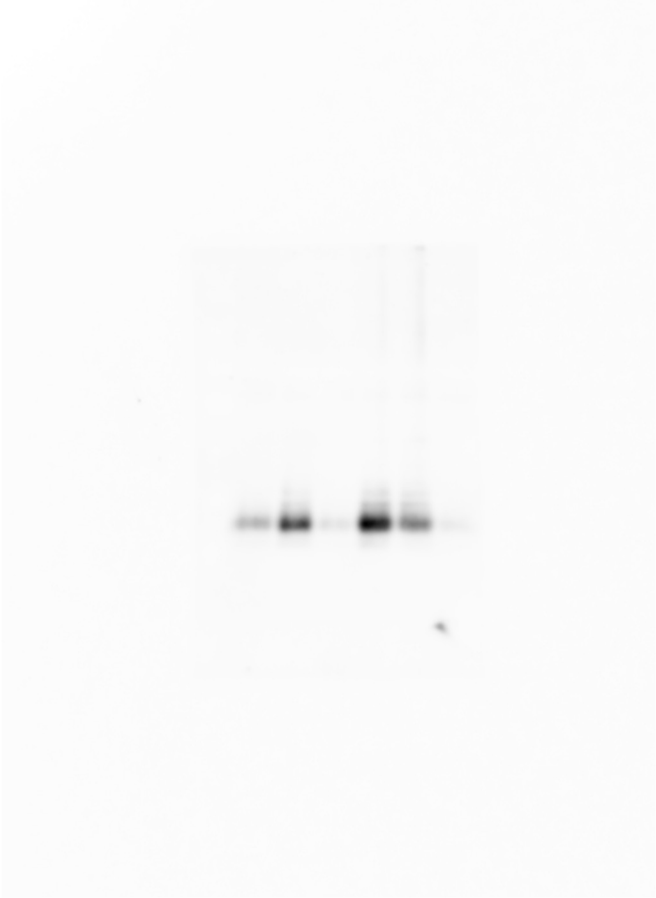

Figure S36: Original blots in Figure 5A (Cell lysate)

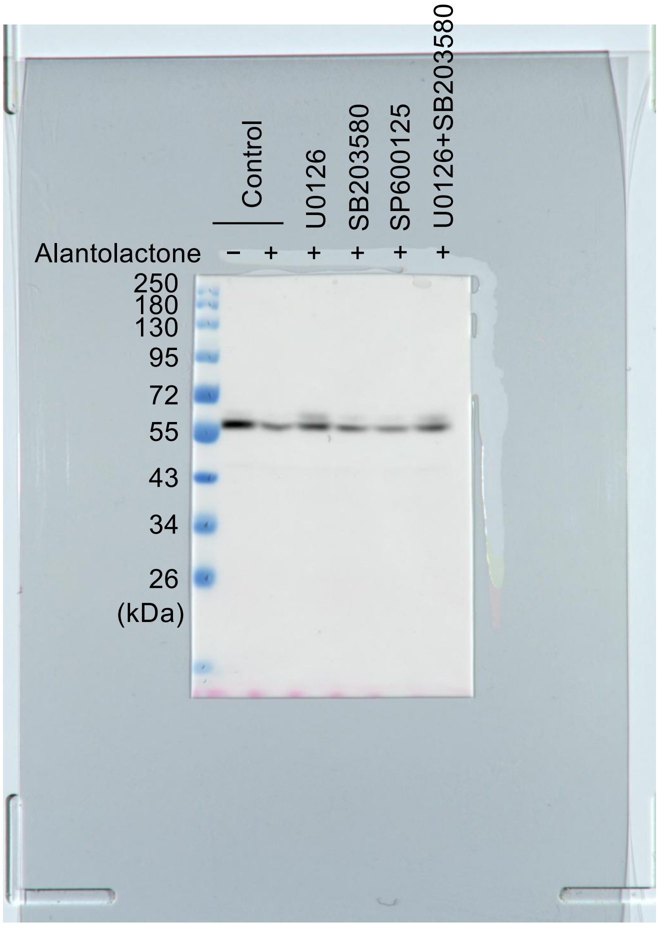

WB: TNF-R1

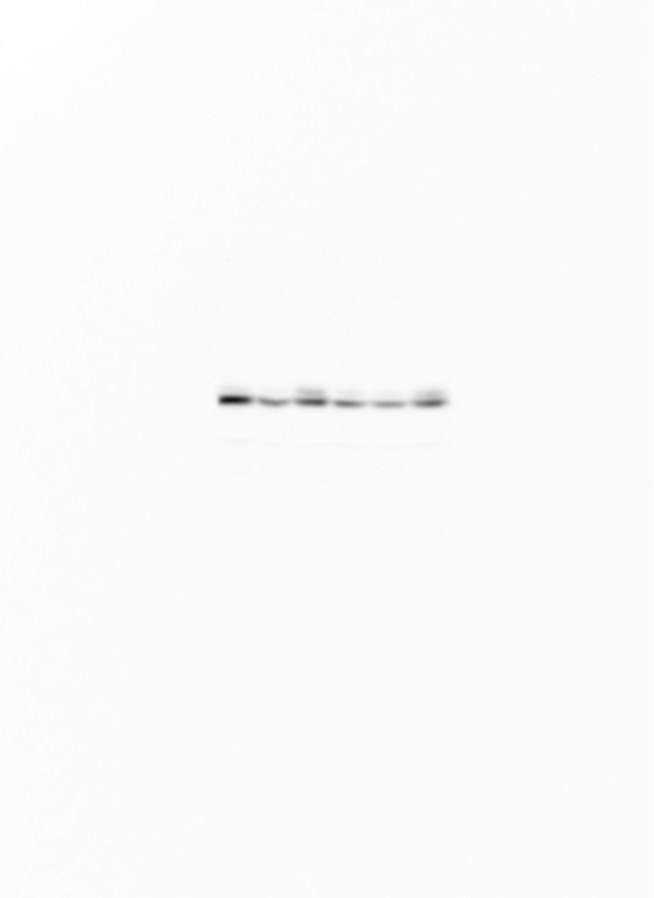

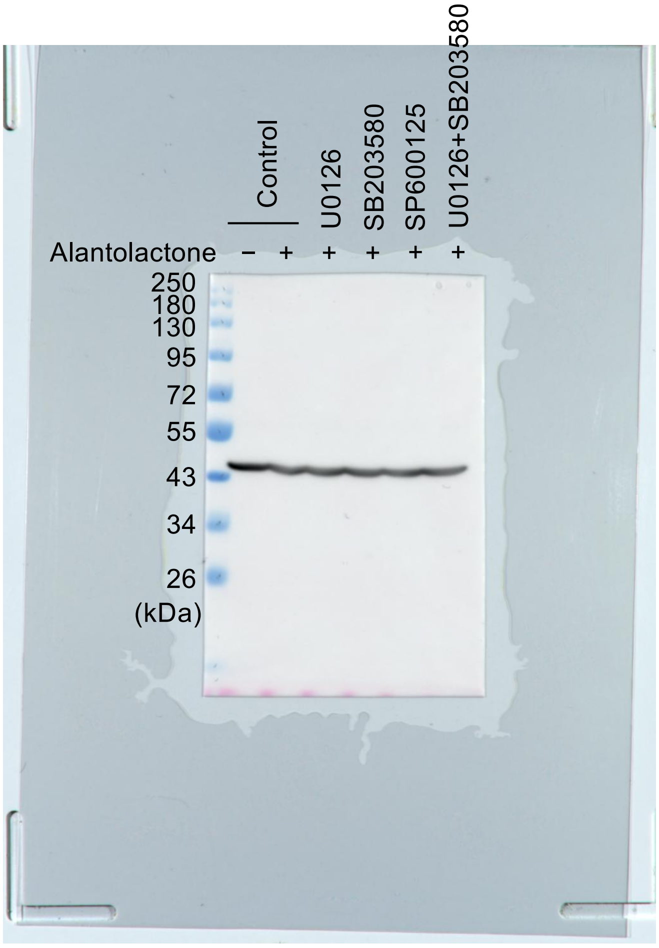

WB: β-Actin (reprobed)

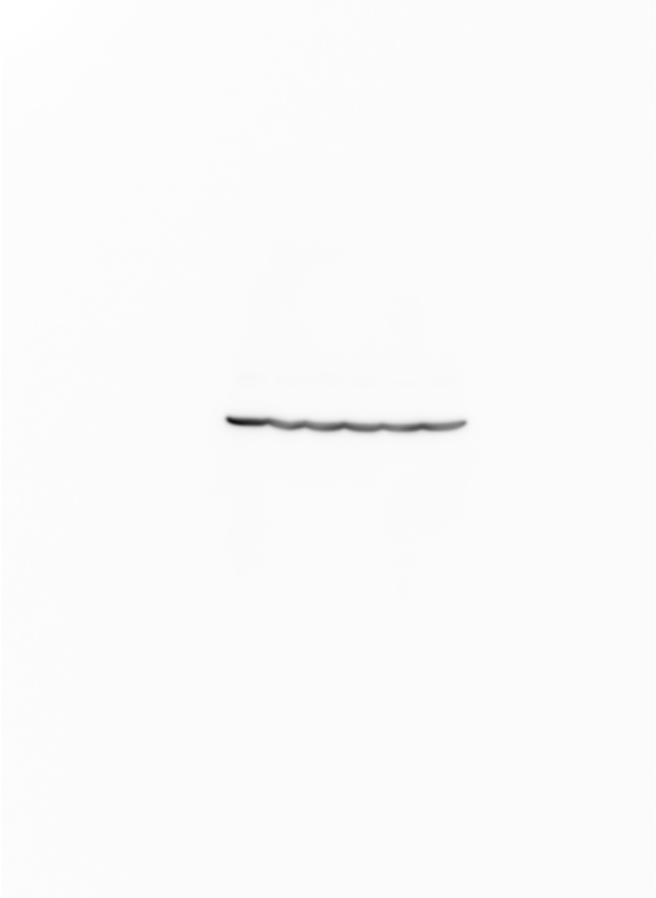

Figure S37: Original blots (1) in Figure 5B

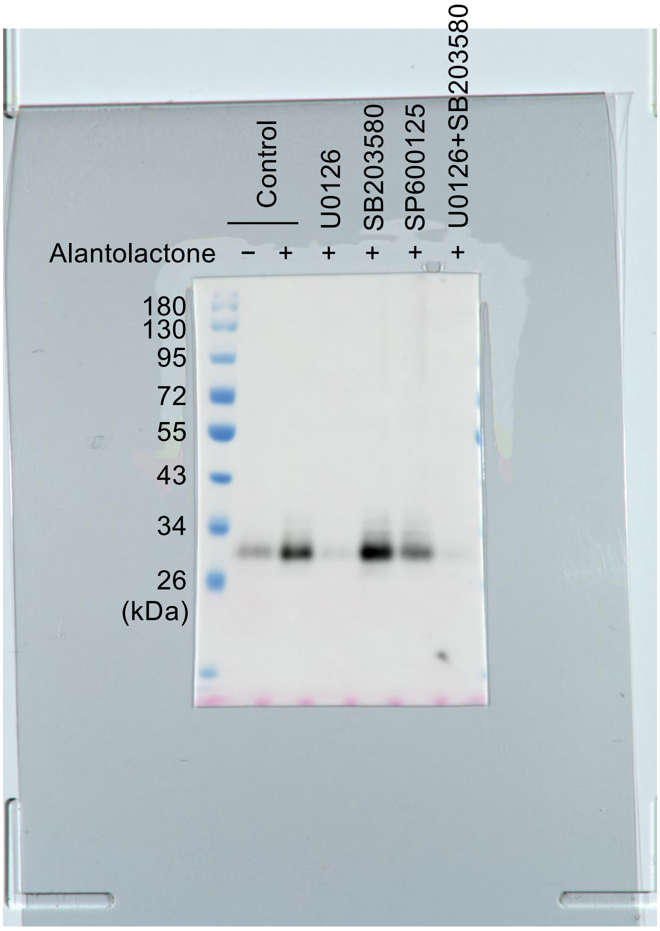

WB: TNF-R1

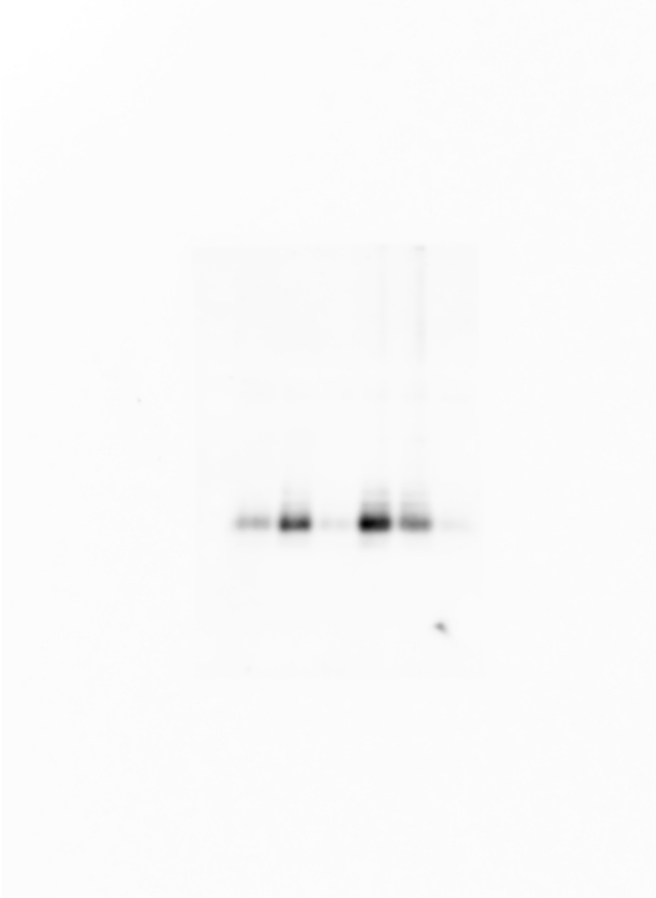

Figure S38: Original blots (2) in Figure 5B

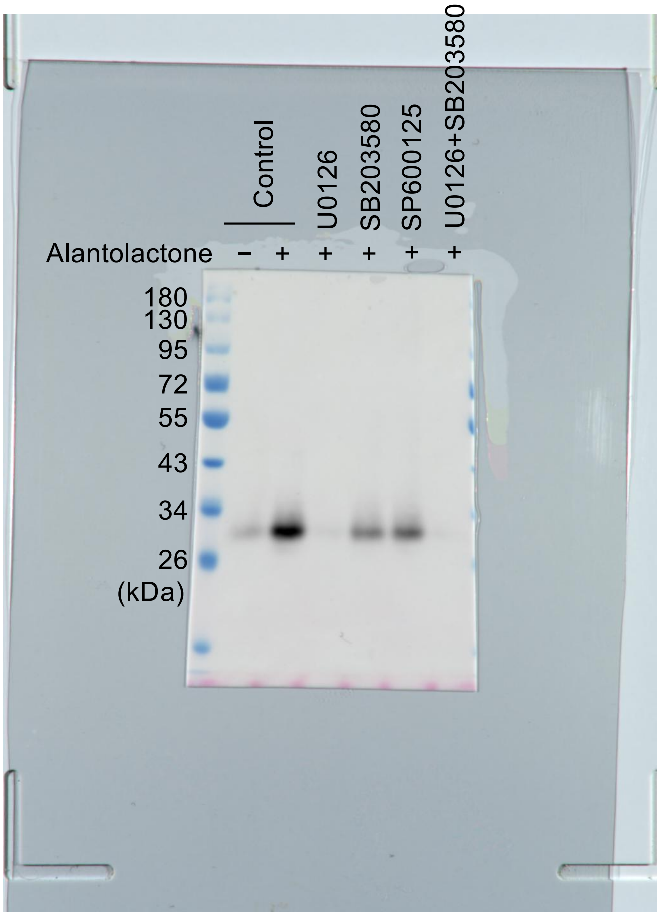

WB: TNF-R1

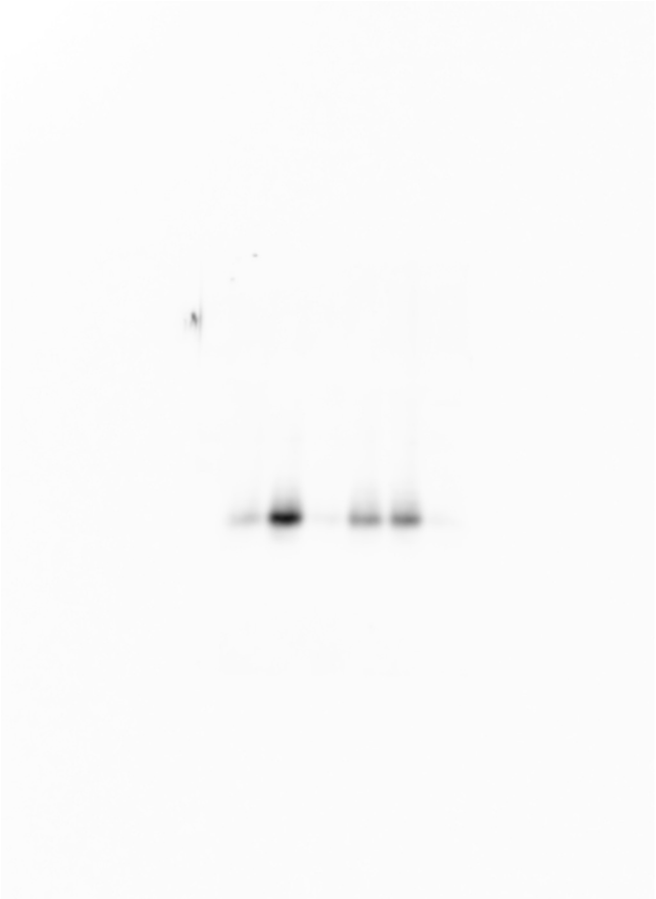

Figure S39: Original blots (3) in Figure 5B

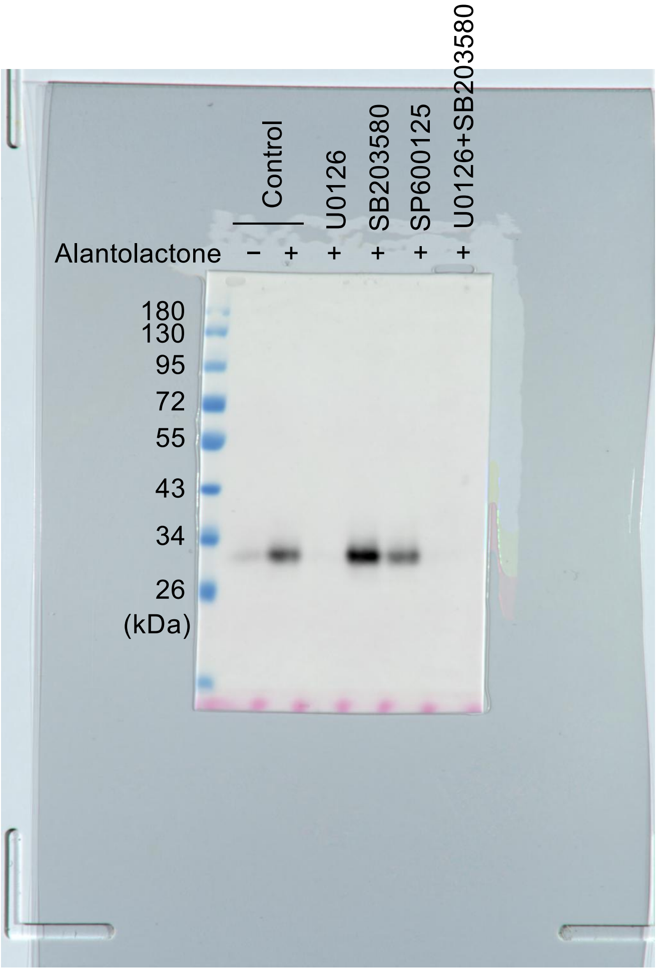

WB: TNF-R1

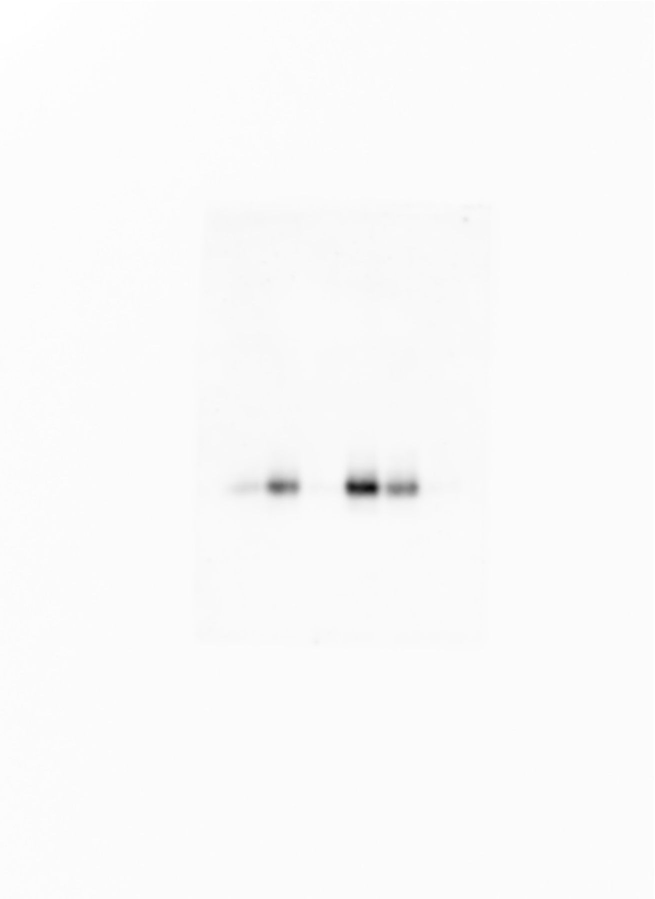

Figure S40: Original blots (1) in Figure 5C

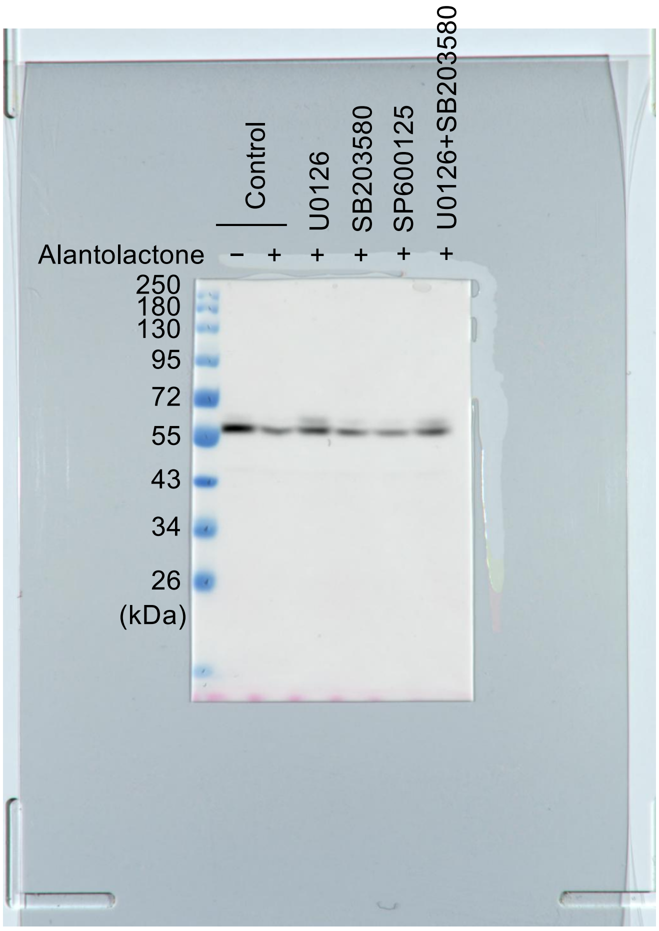

WB: TNF-R1

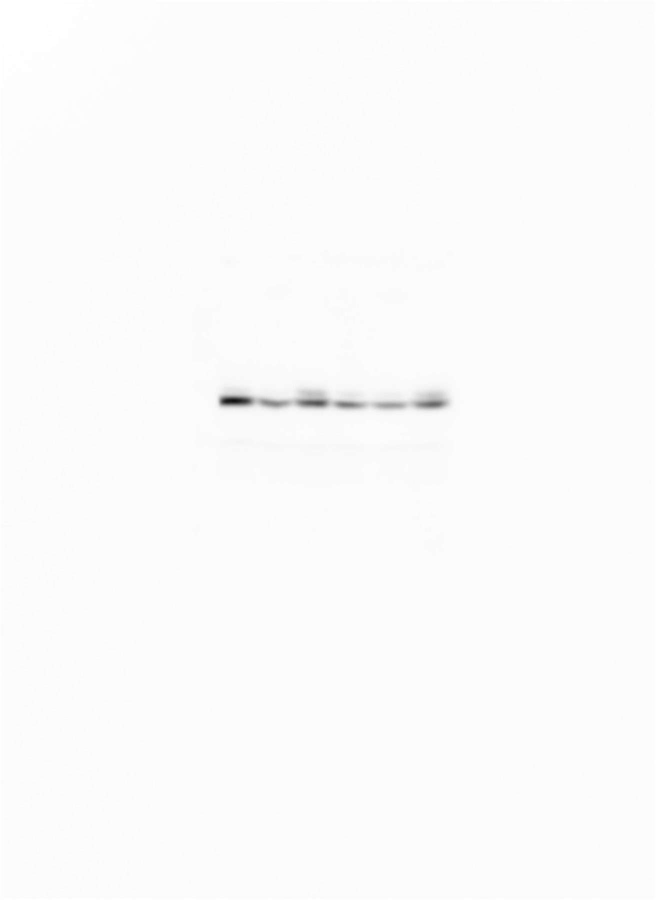

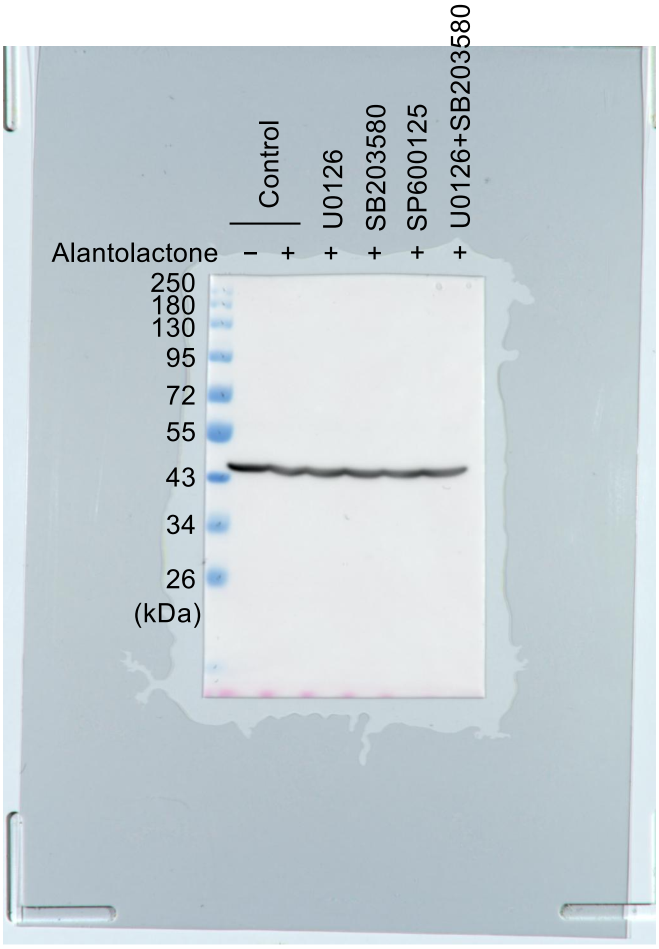

WB: β-Actin (reprobed)

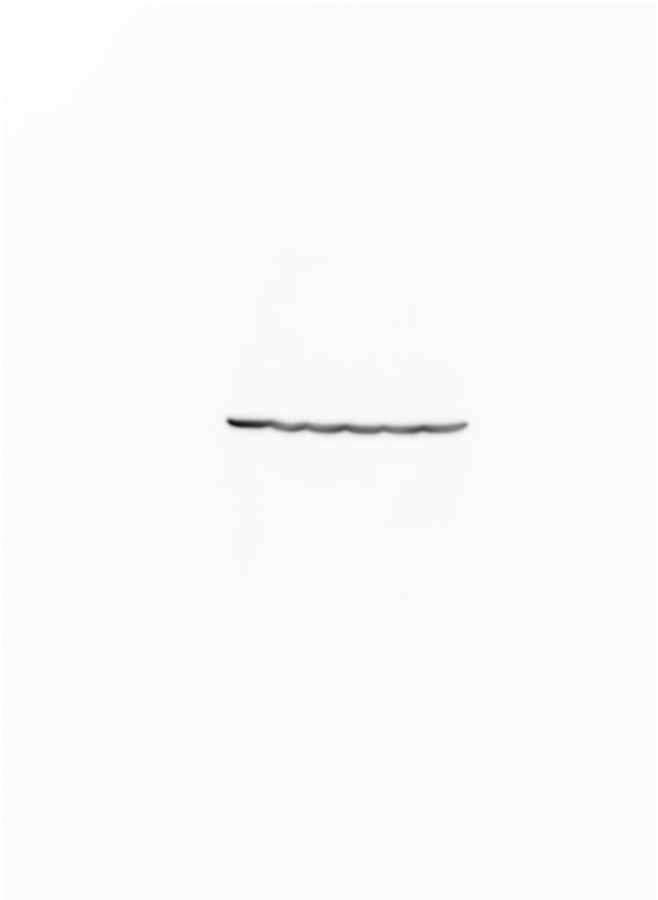

Figure S41: Original blots (2) in Figure 5C

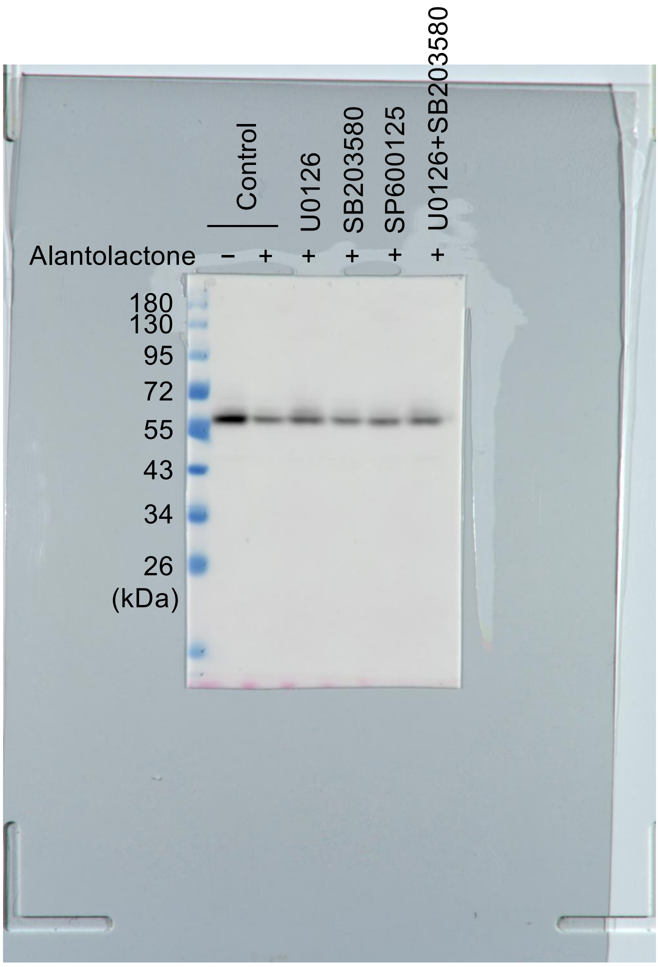

WB: TNF-R1

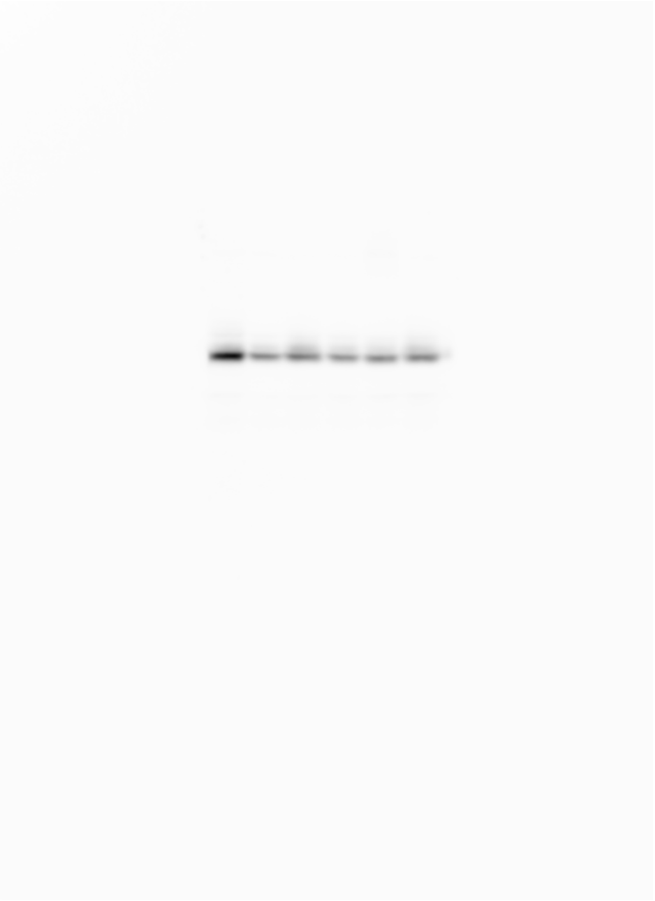

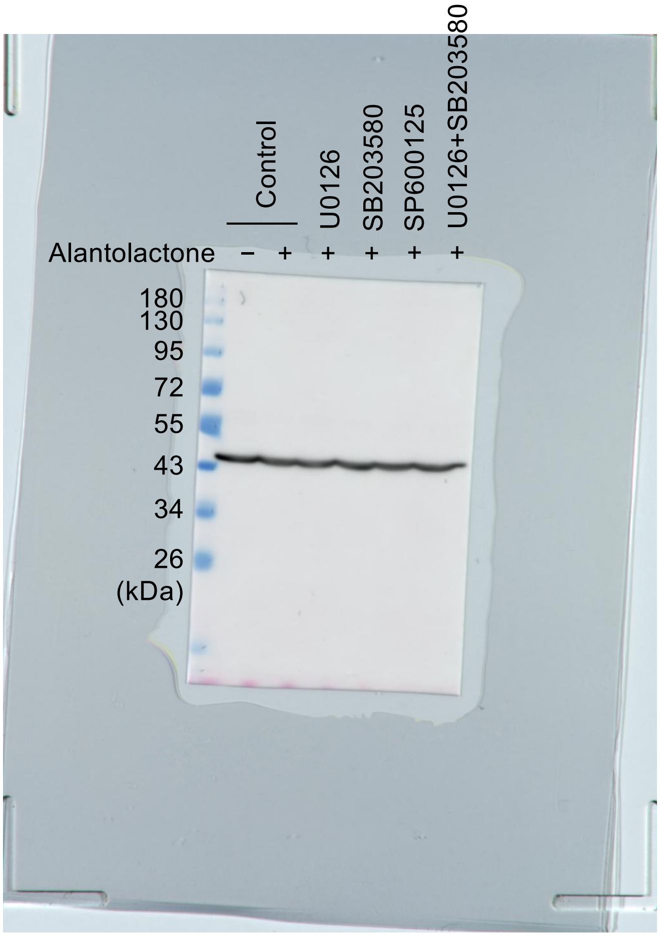

WB: β-Actin (reprobed)

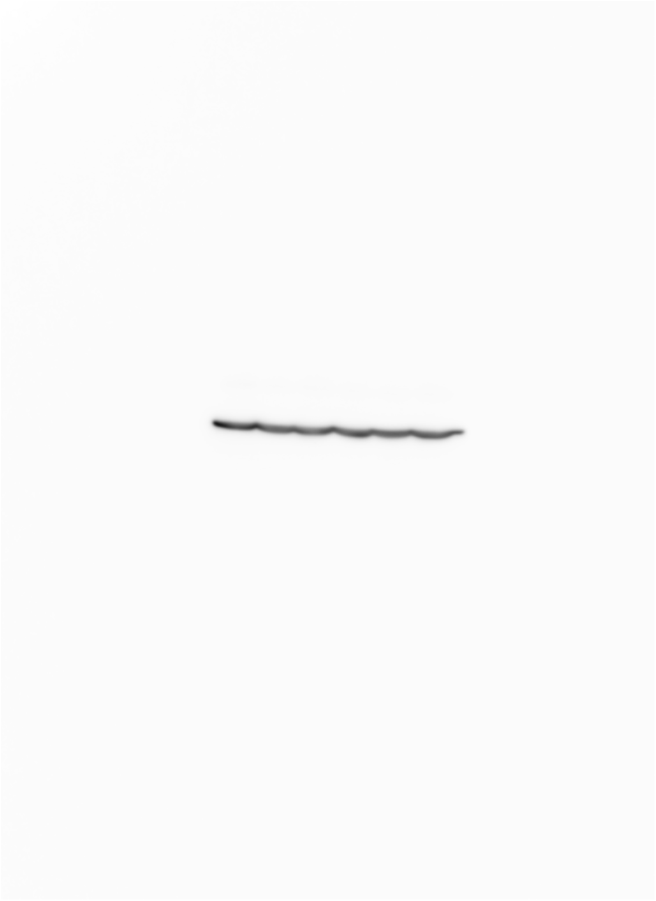

Figure S42: Original blots (3) in Figure 5C

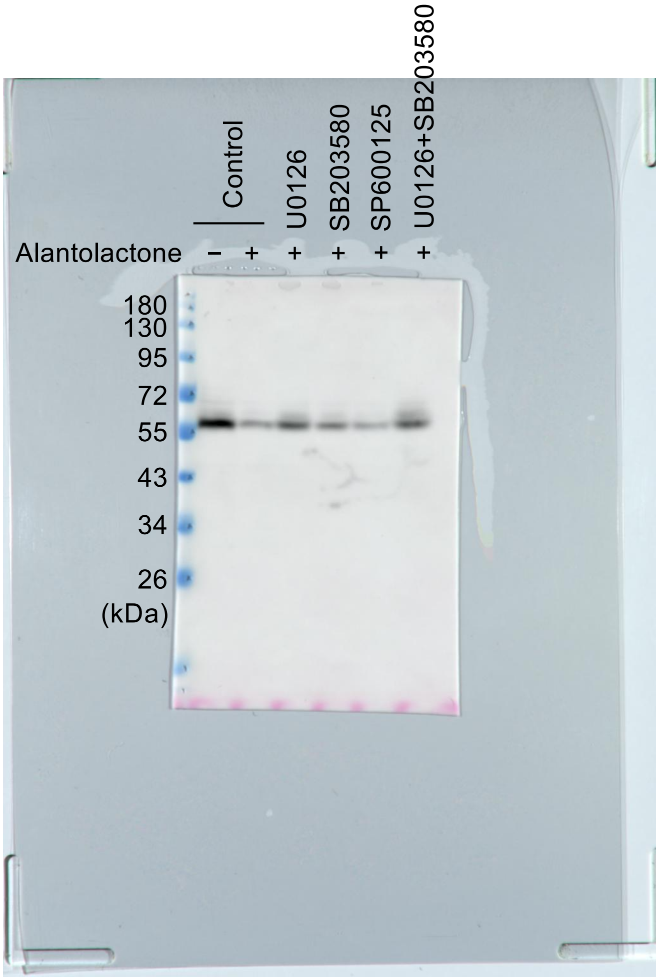

WB: TNF-R1

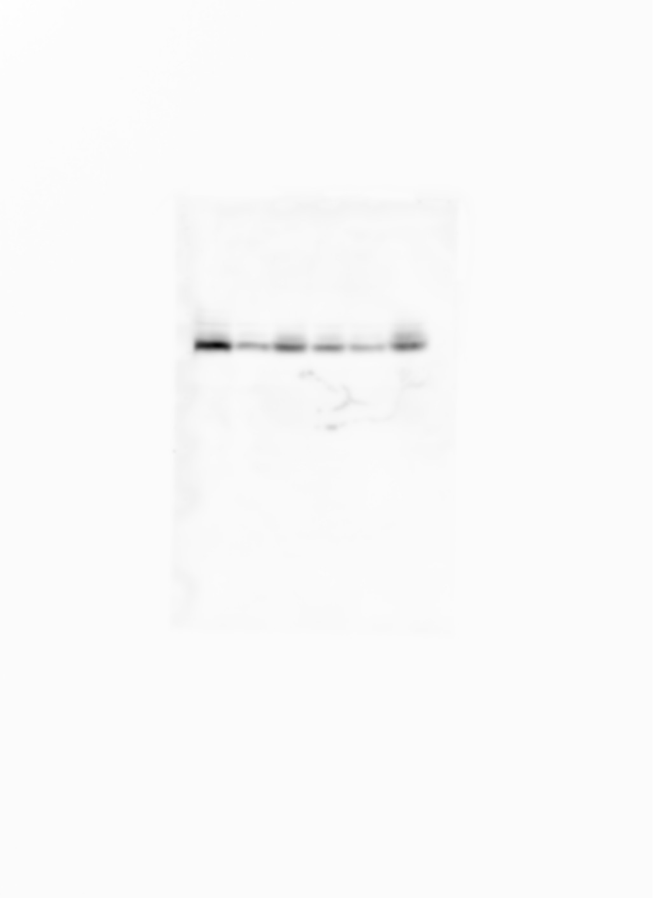

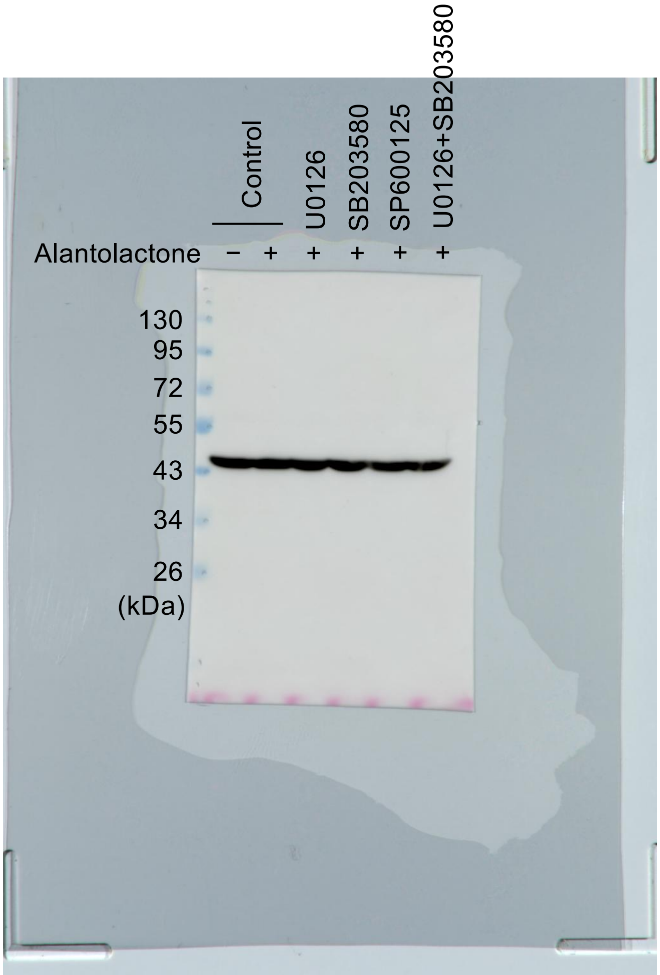

WB: β-Actin (reprobed)

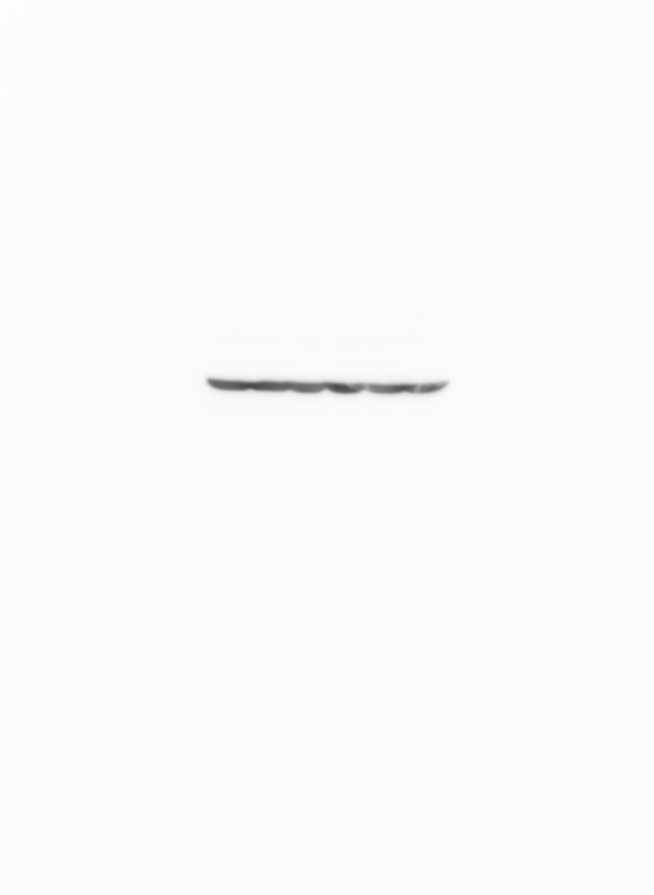

Figure S43: Original blots in Figure 6A (Phospho-ERK)

WB: Phospho-ERK

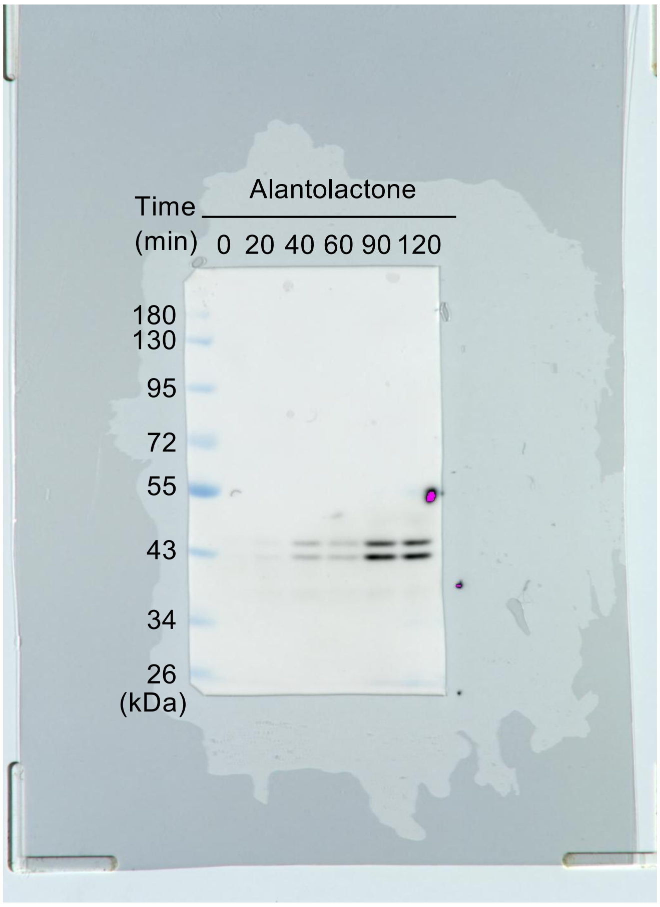

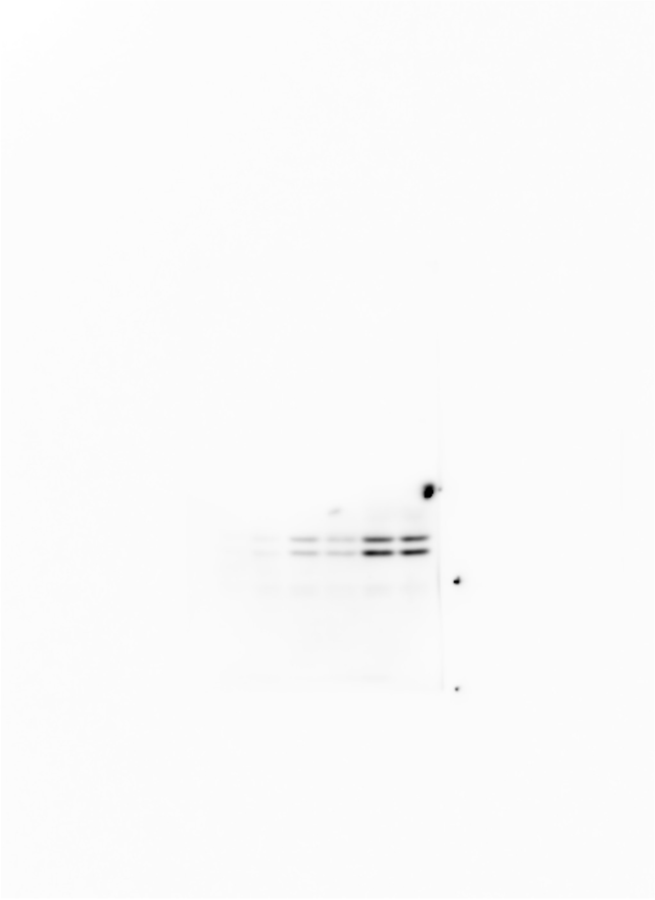

WB: GAPDH (reprobed)

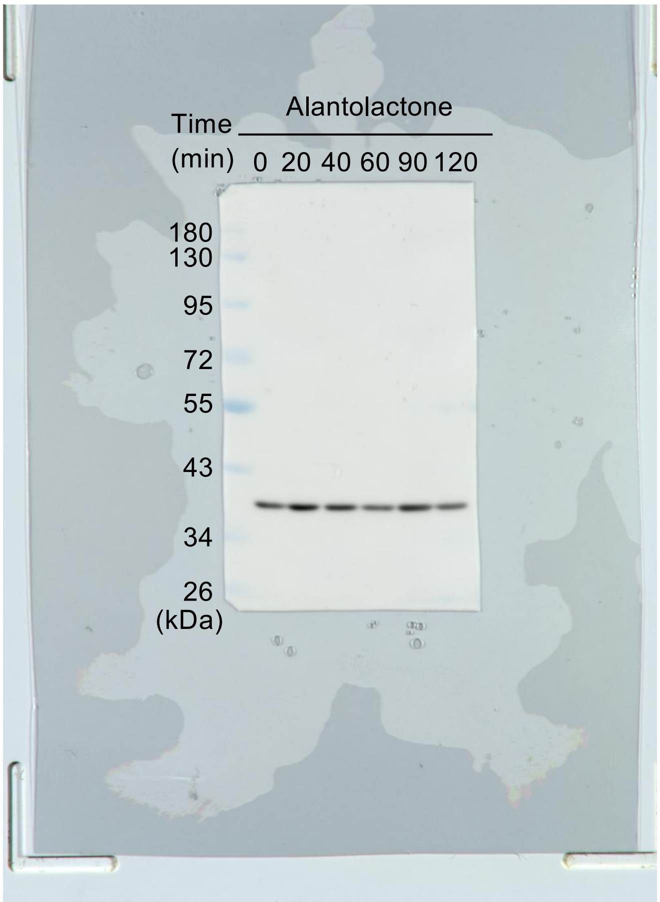

Figure S44: Original blots in Figure 6A (ERK)

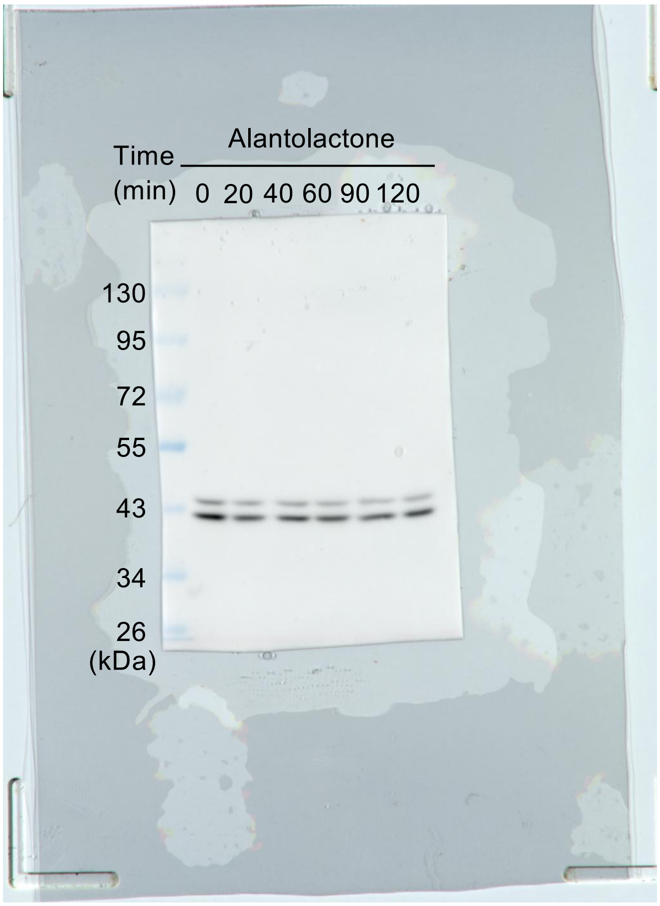

WB: ERK

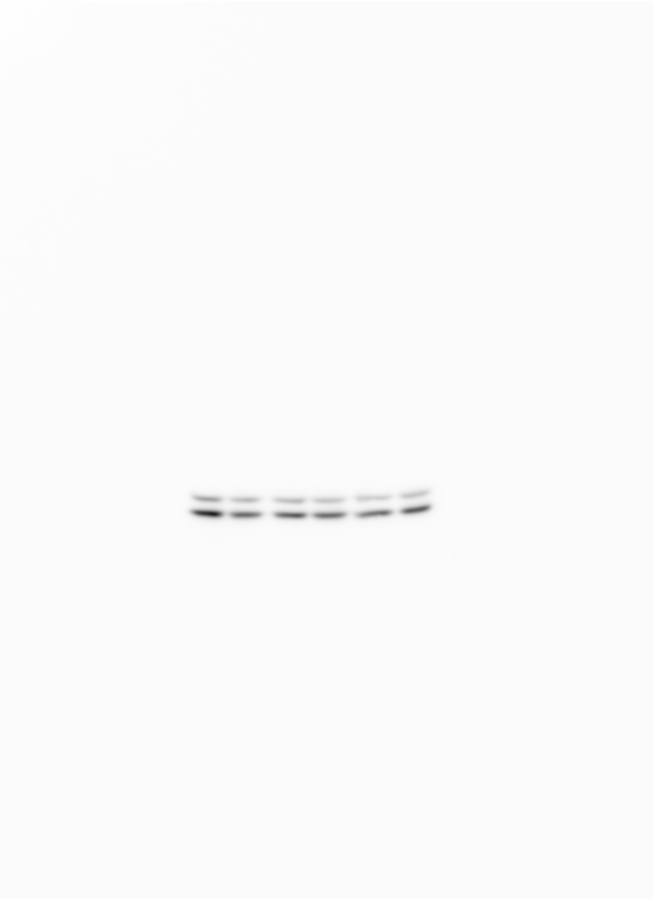

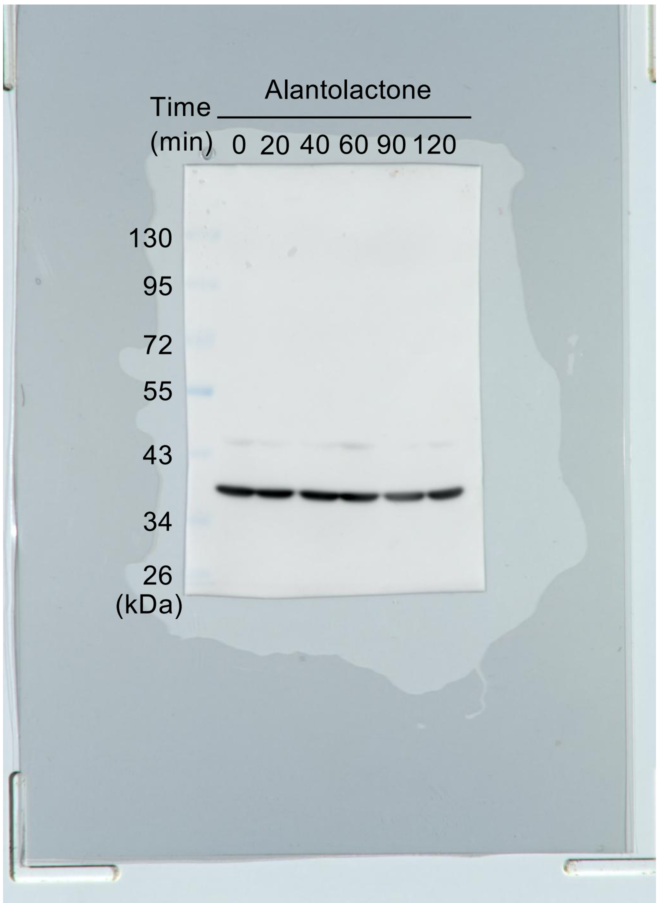

WB: GAPDH (reprobed)

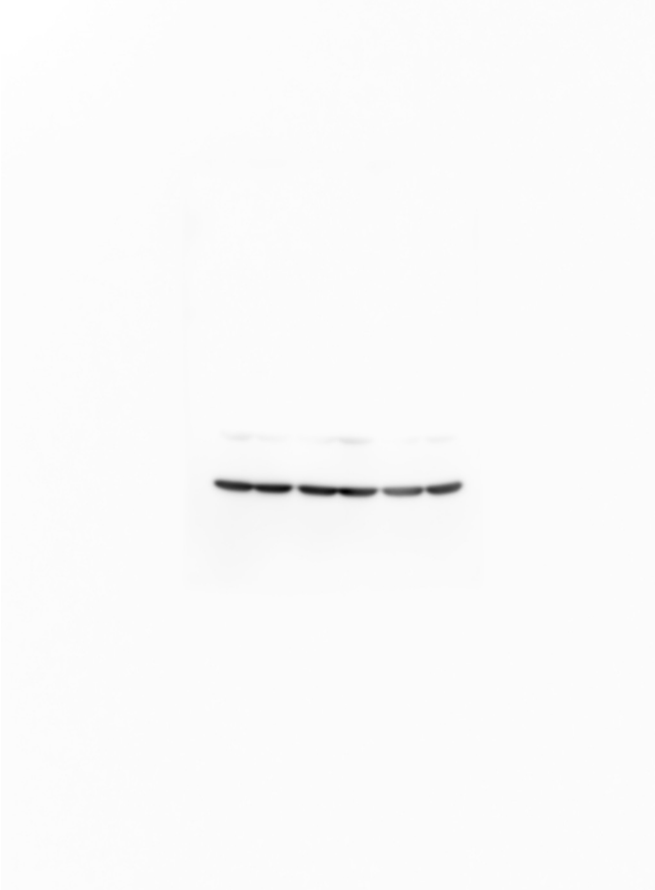

Figure S45: Original blots (1) in Figure 6B,D

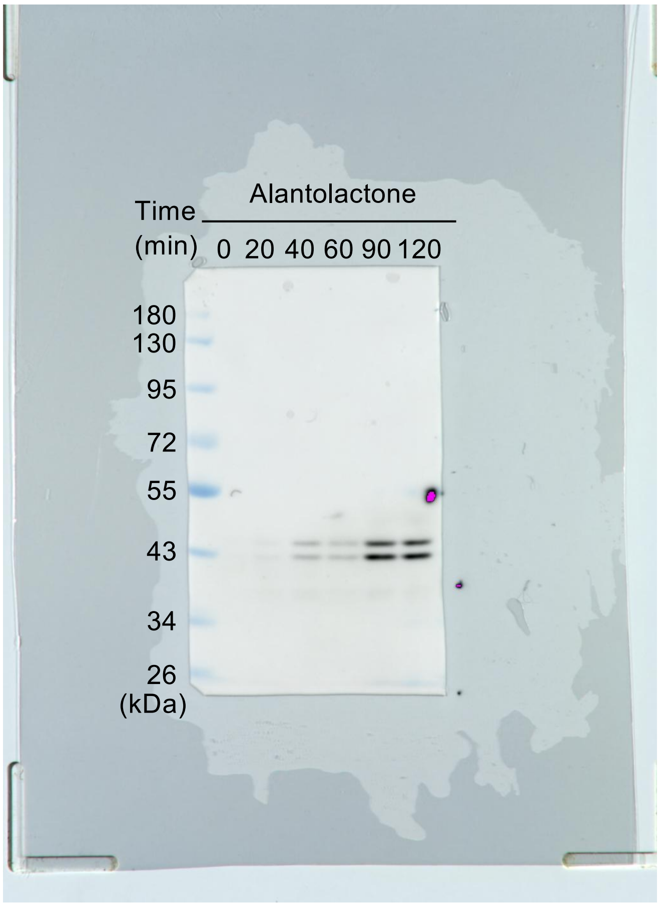

WB: Phospho-ERK

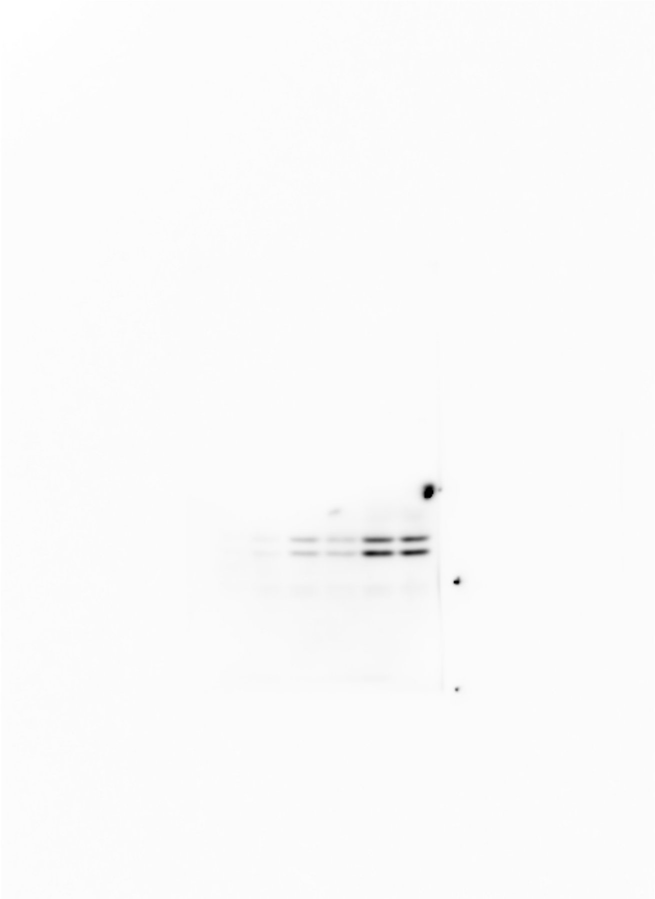

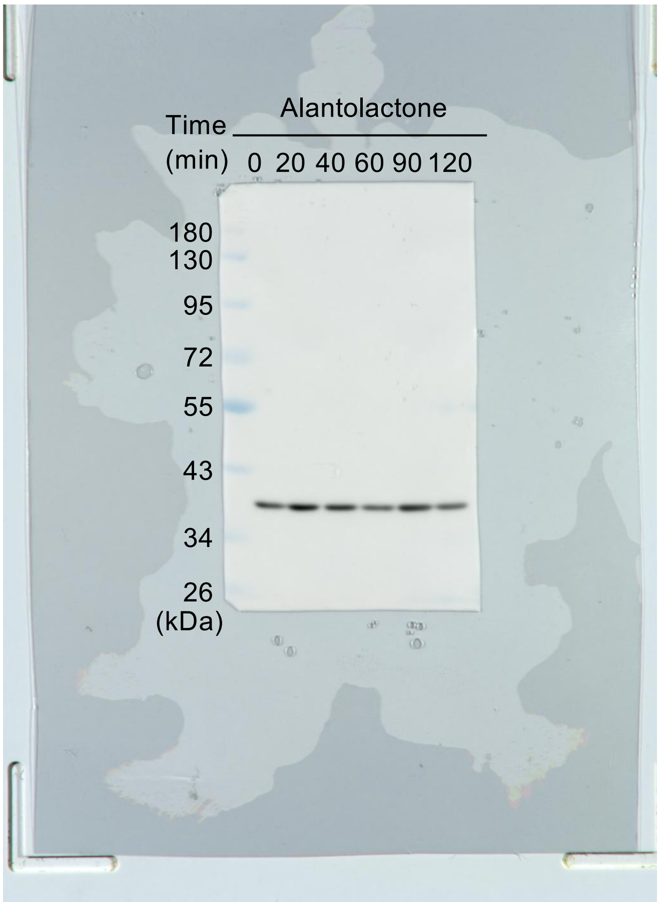

WB: GAPDH (reprobed)

Figure S46: Original blots (2) in Figure 6B,D

WB: Phospho-ERK

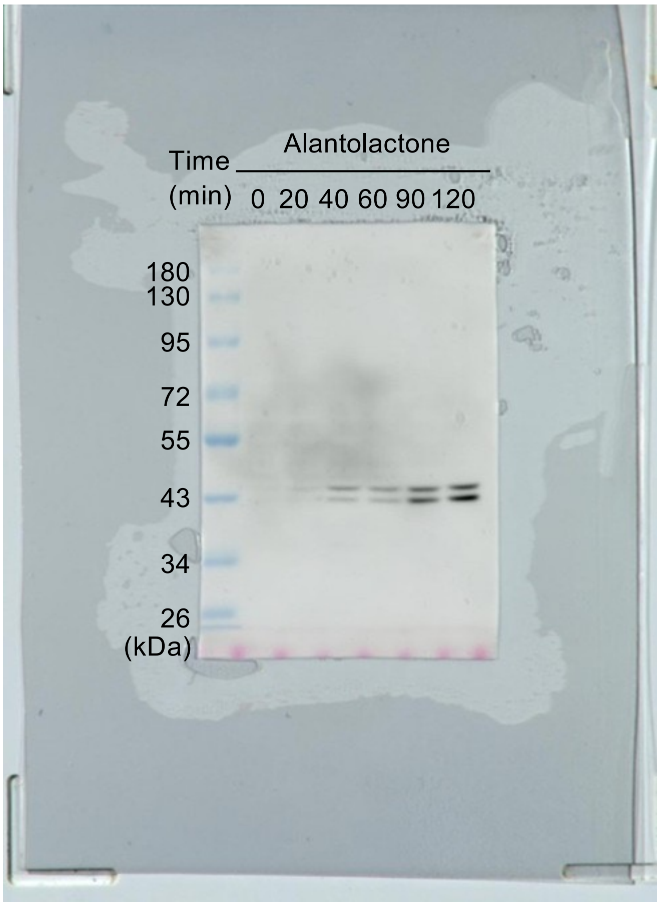

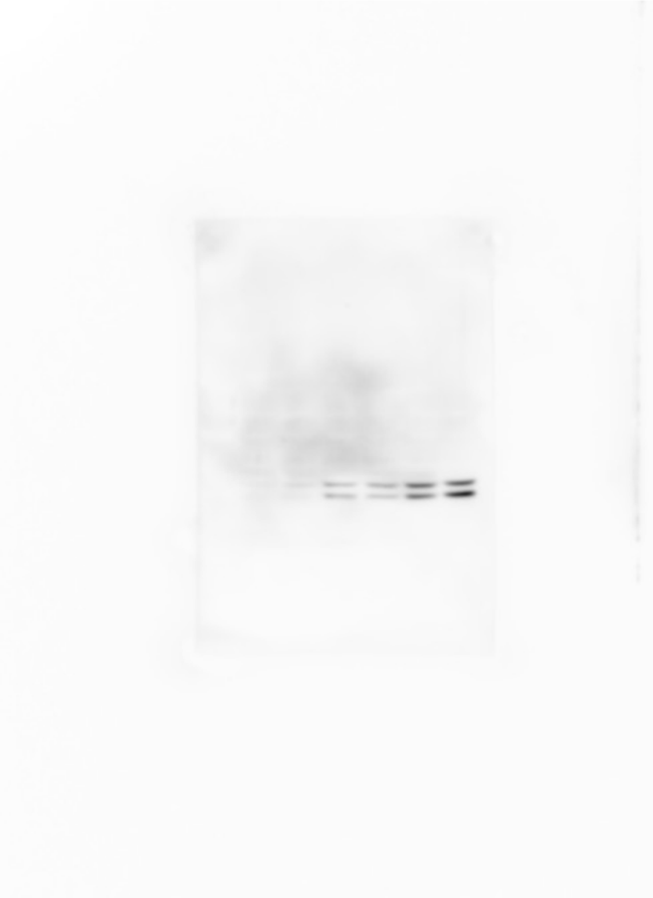

WB: GAPDH (reprobed)

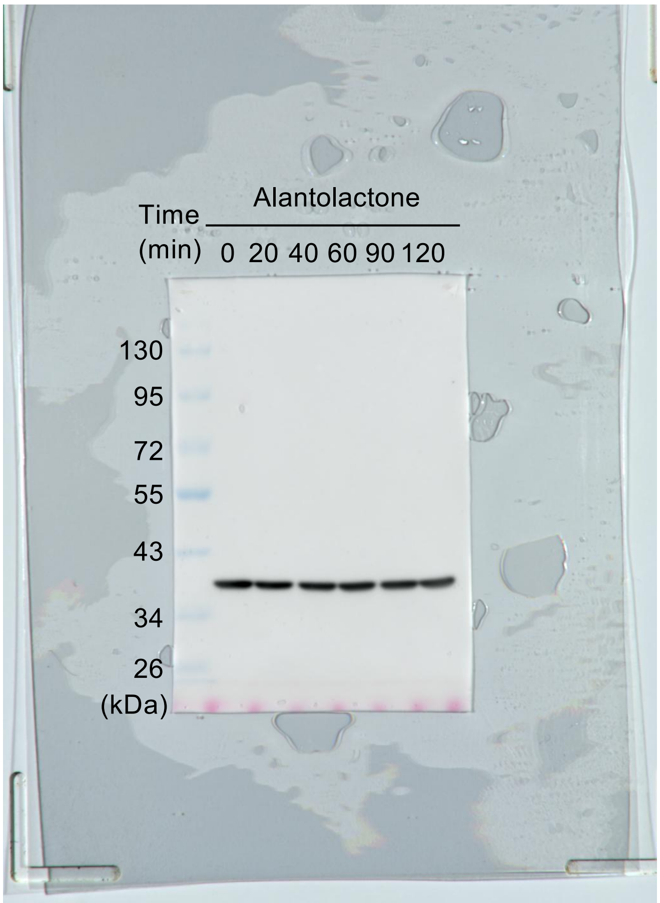

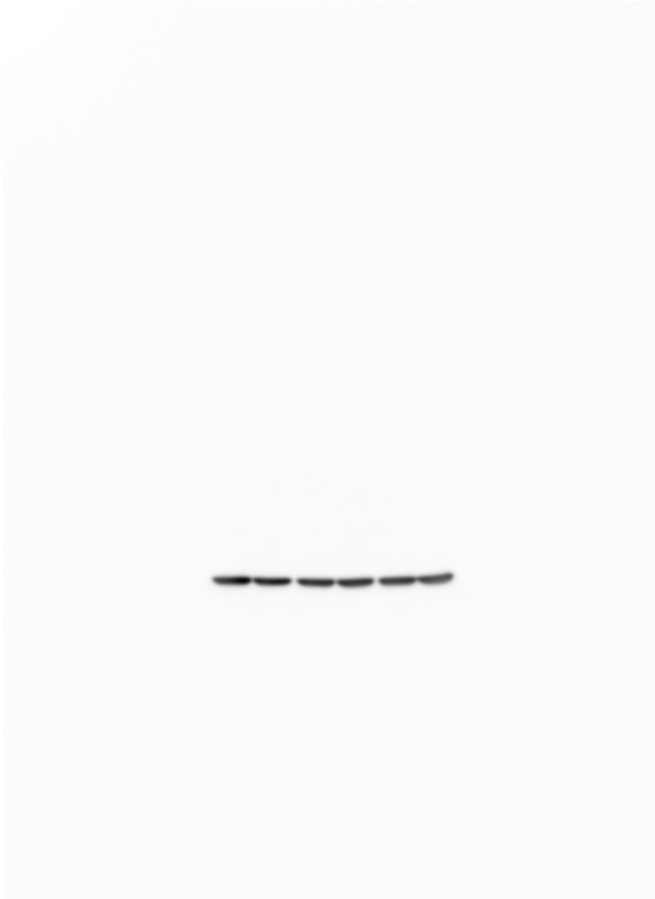

Figure S47: Original blots (3) in Figure 6B,D

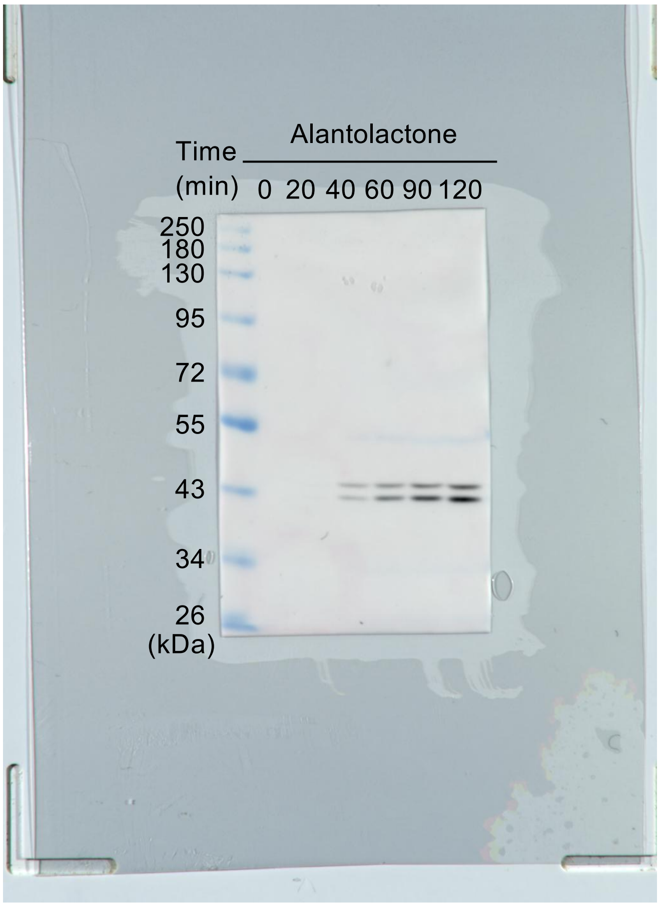

WB: Phospho-ERK

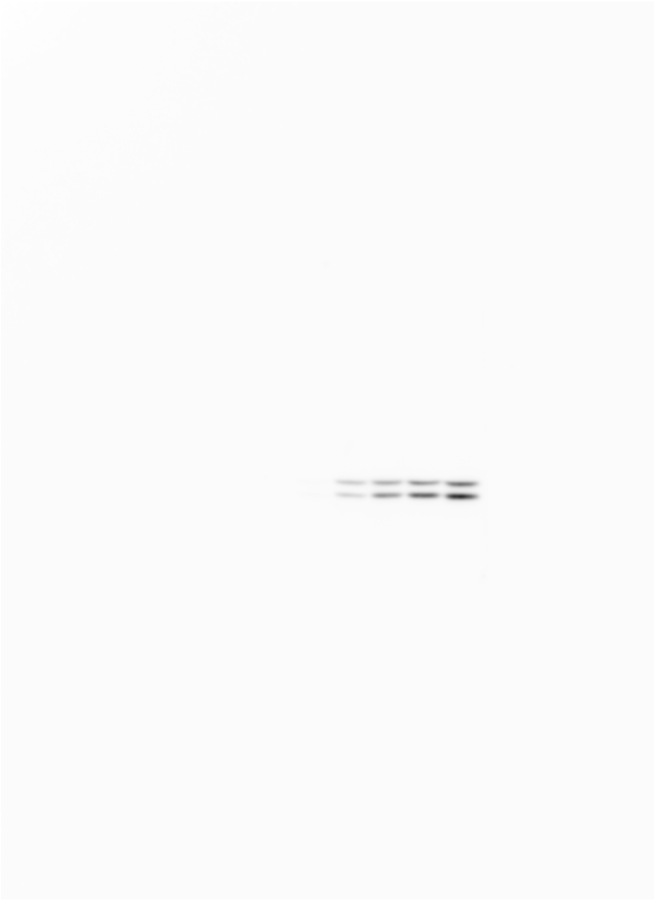

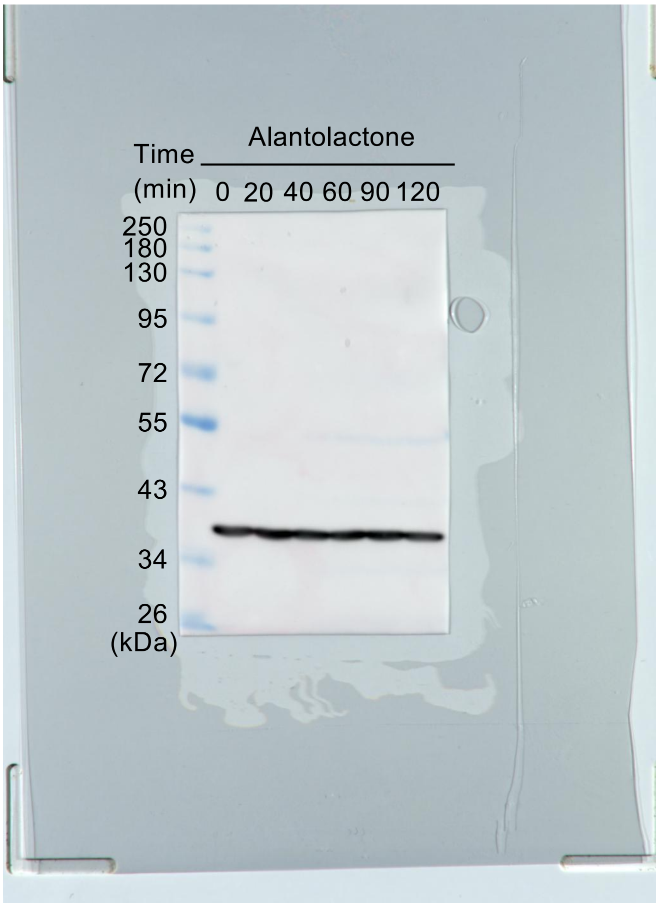

WB: GAPDH (reprobed)

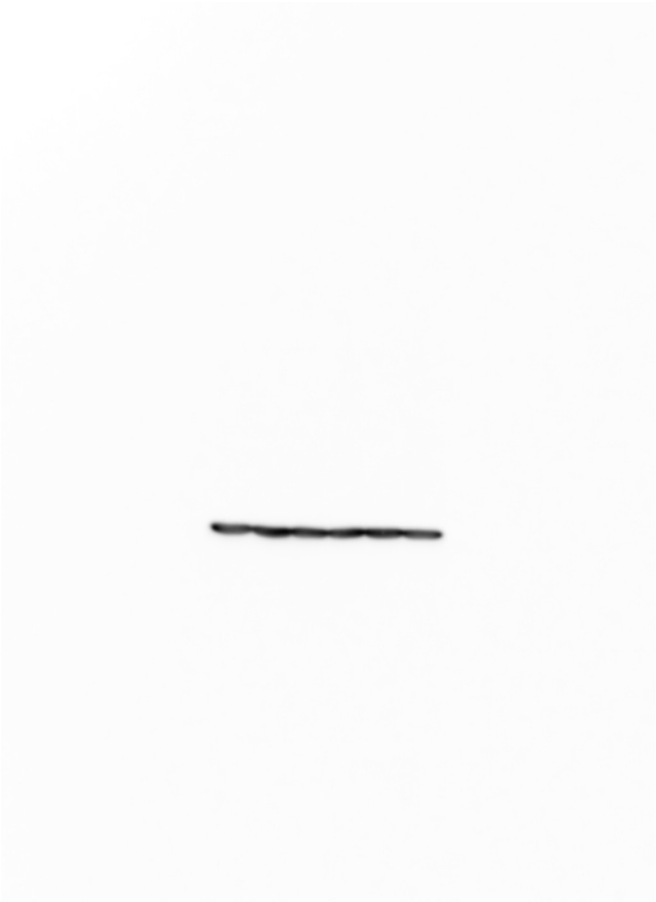

Figure S48: Original blots (1) in Figure 6C,D

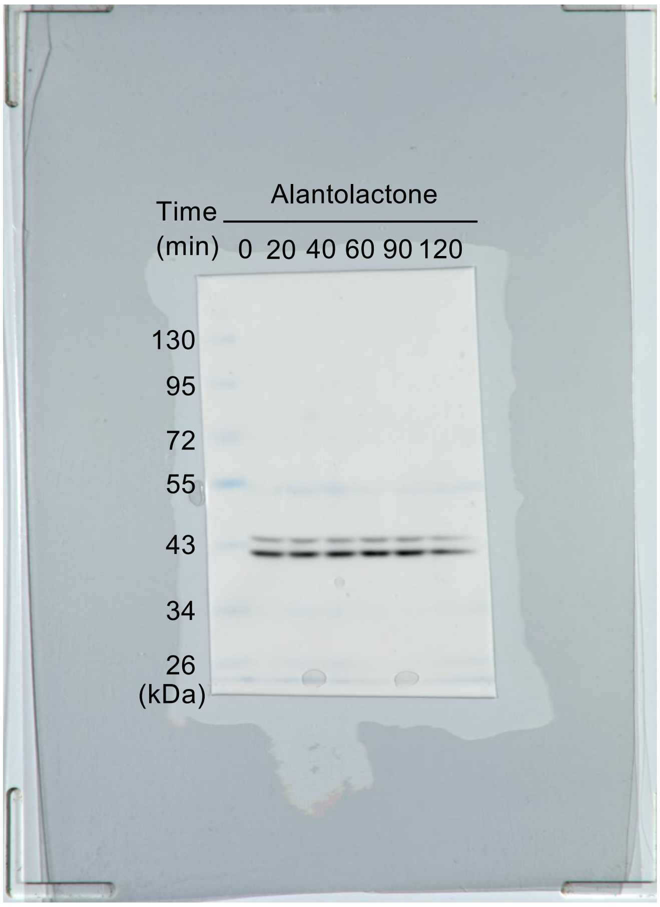

WB: ERK

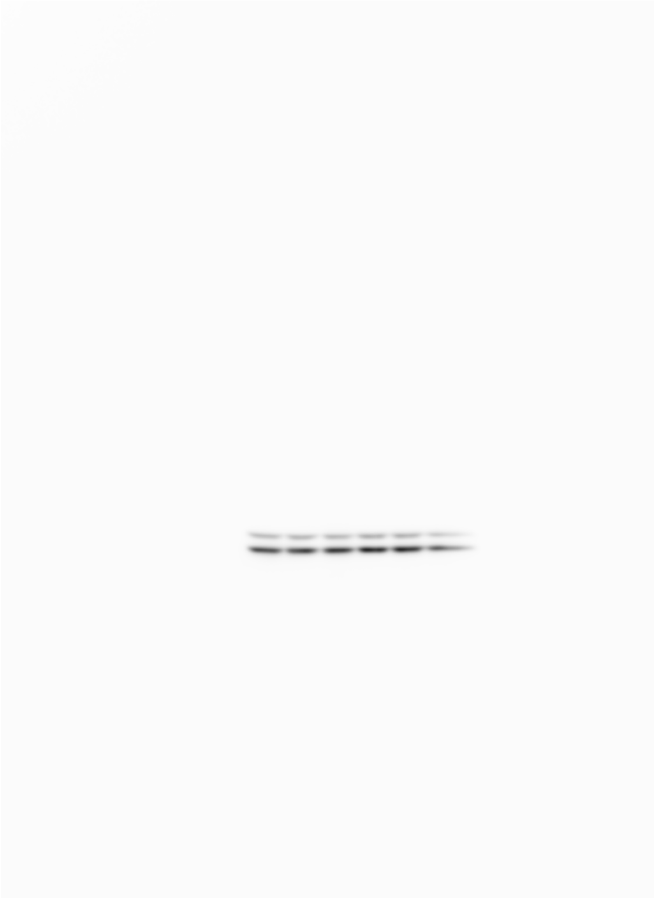

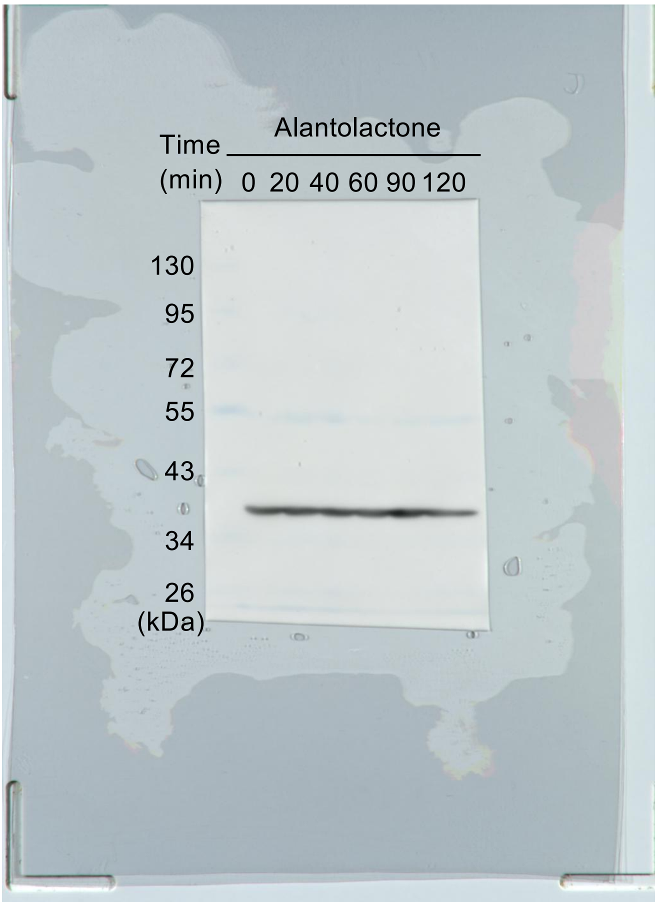

WB: GAPDH (reprobed)

Figure S49: Original blots (2) in Figure 6C,D

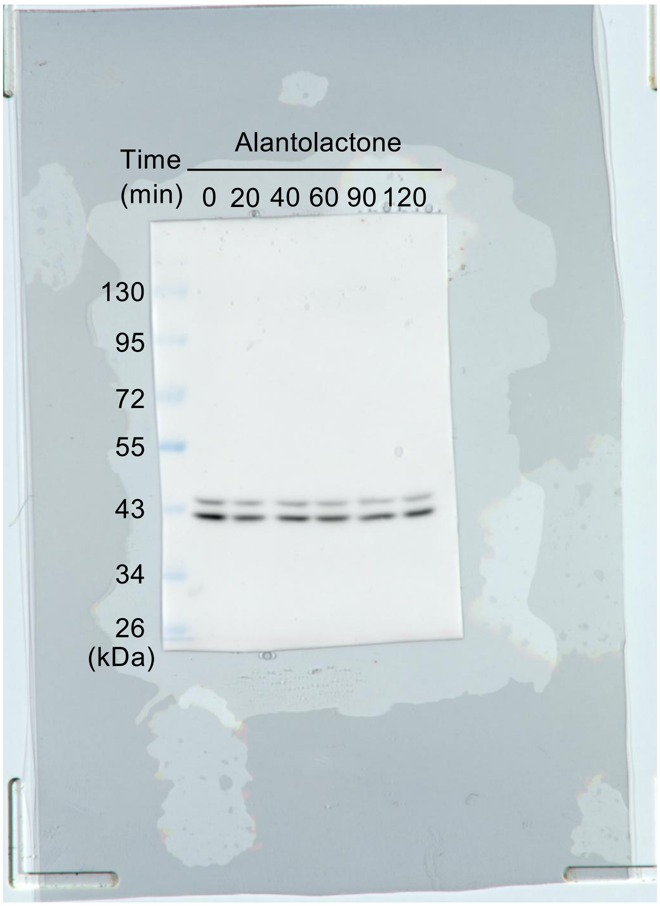

WB: ERK

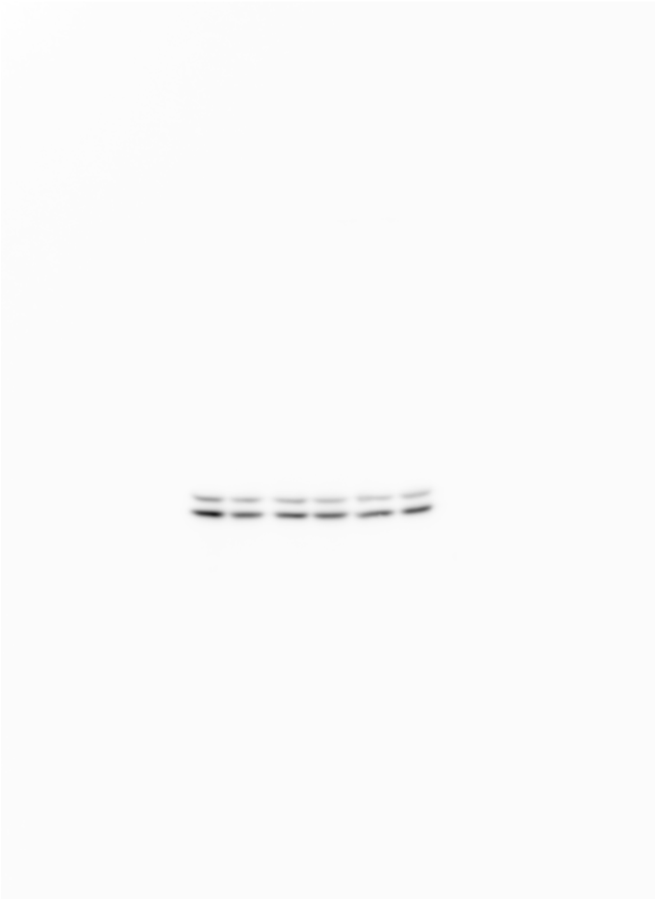

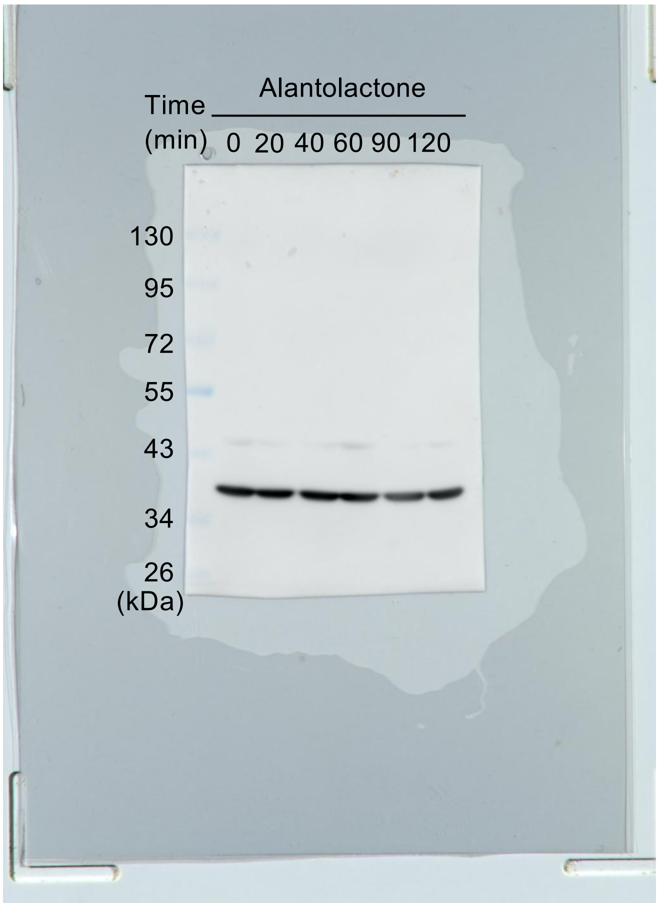

WB: GAPDH (reprobed)

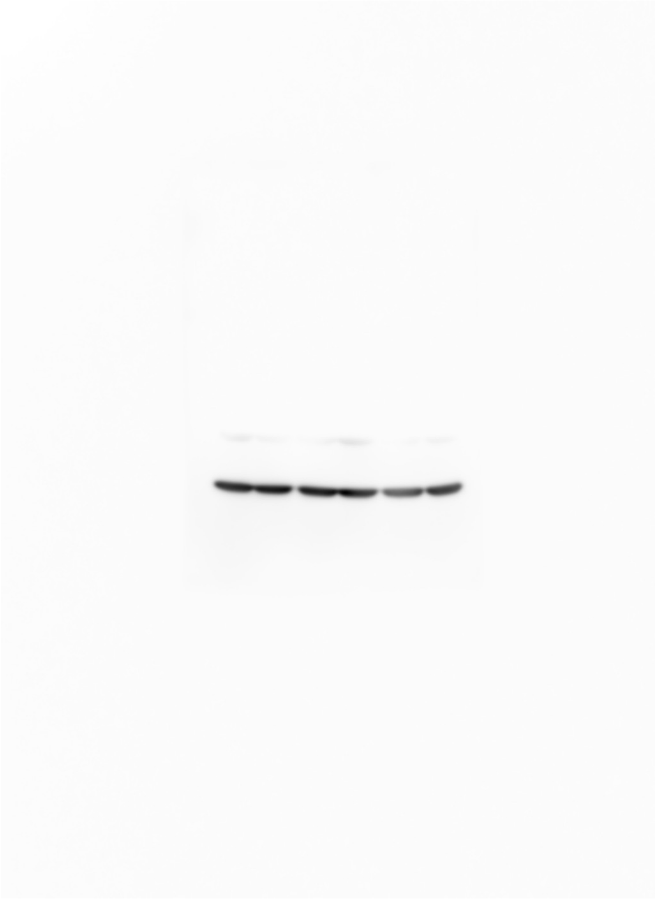

Figure S50: Original blots (3) in Figure 6C,D

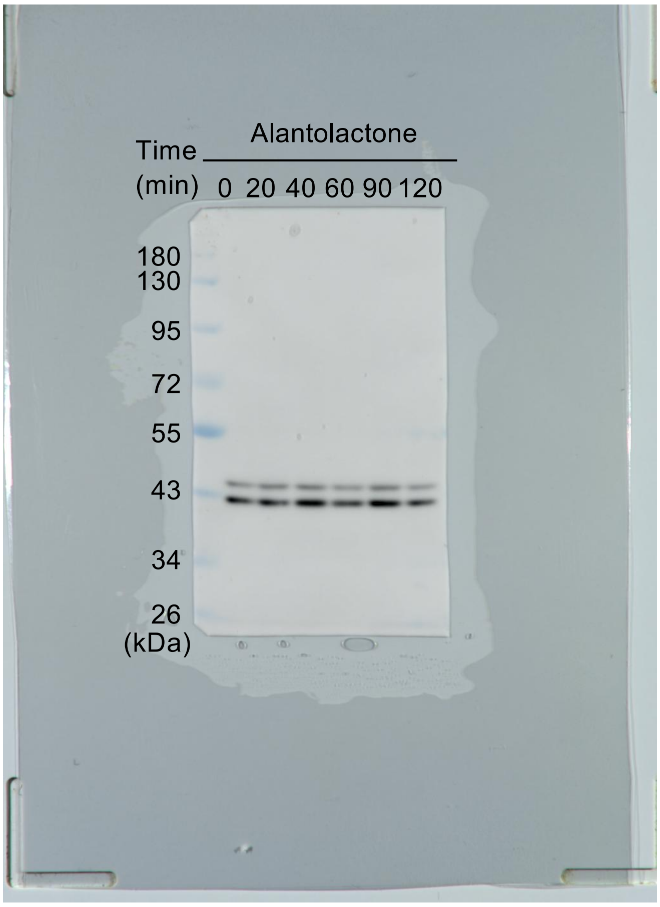

WB: ERK

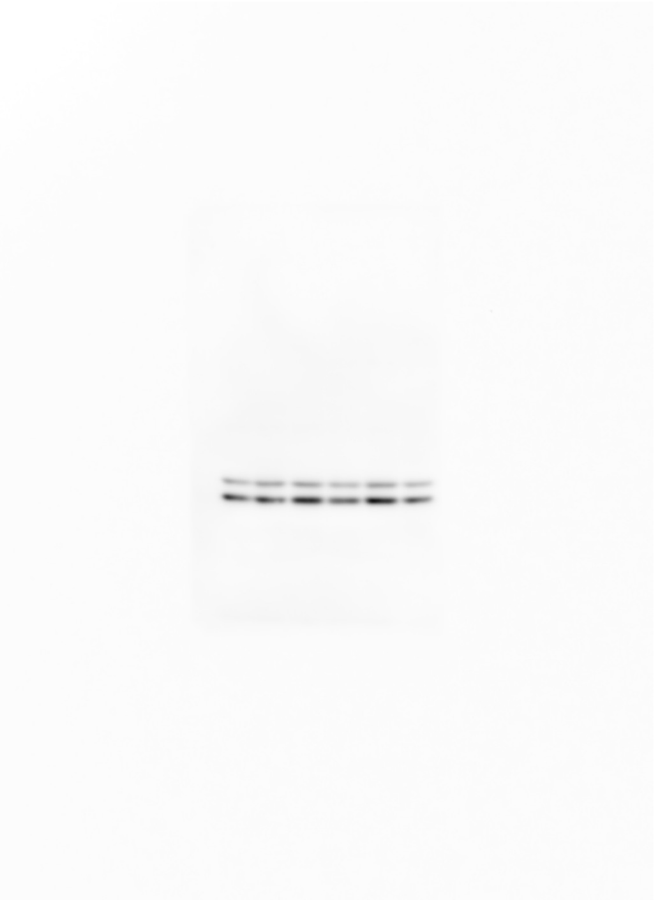

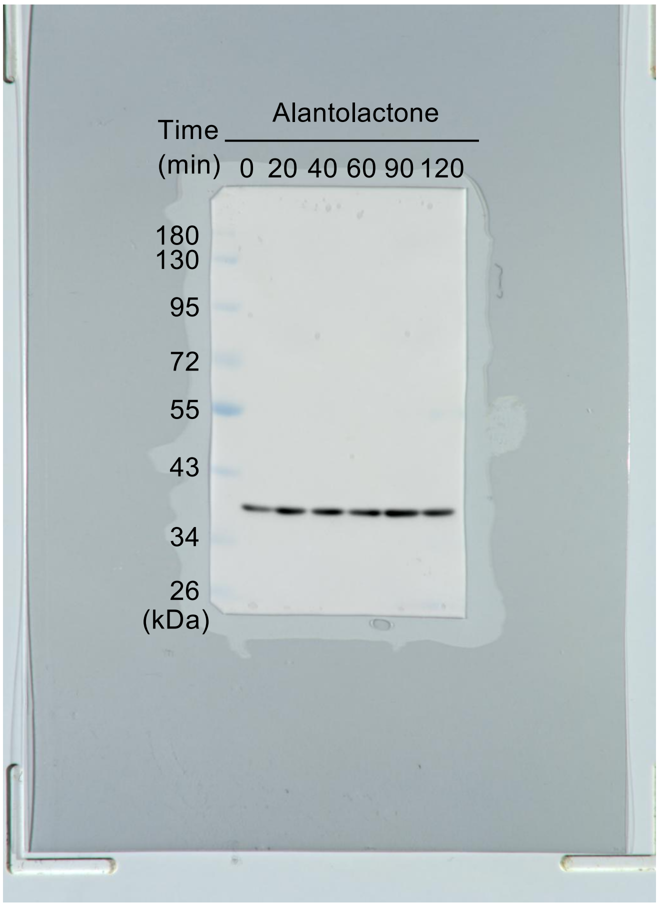

WB: GAPDH (reprobed)

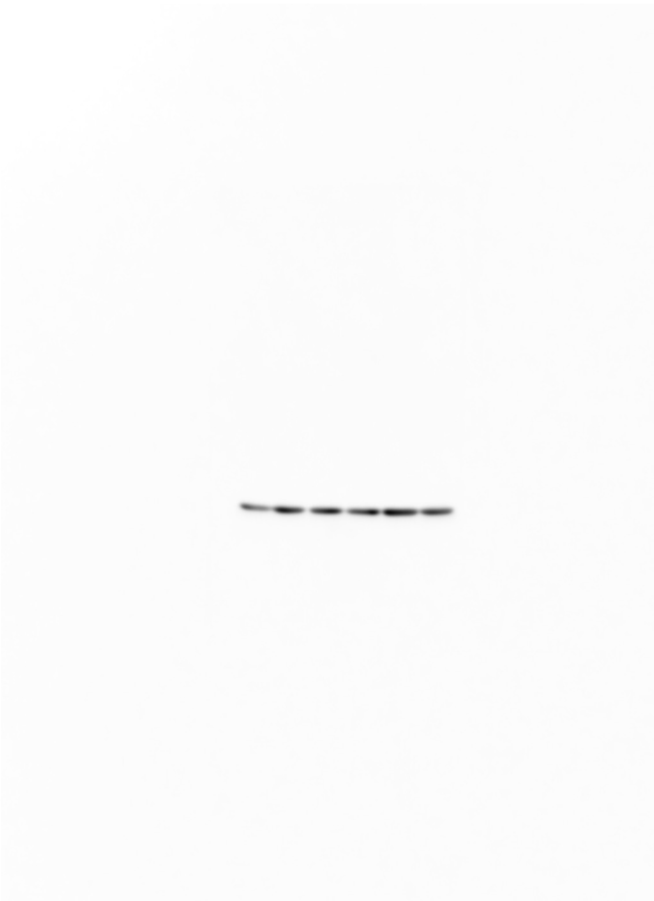

Figure S51: Original blots in Figure 6E (Phospho-p38 MAPK)

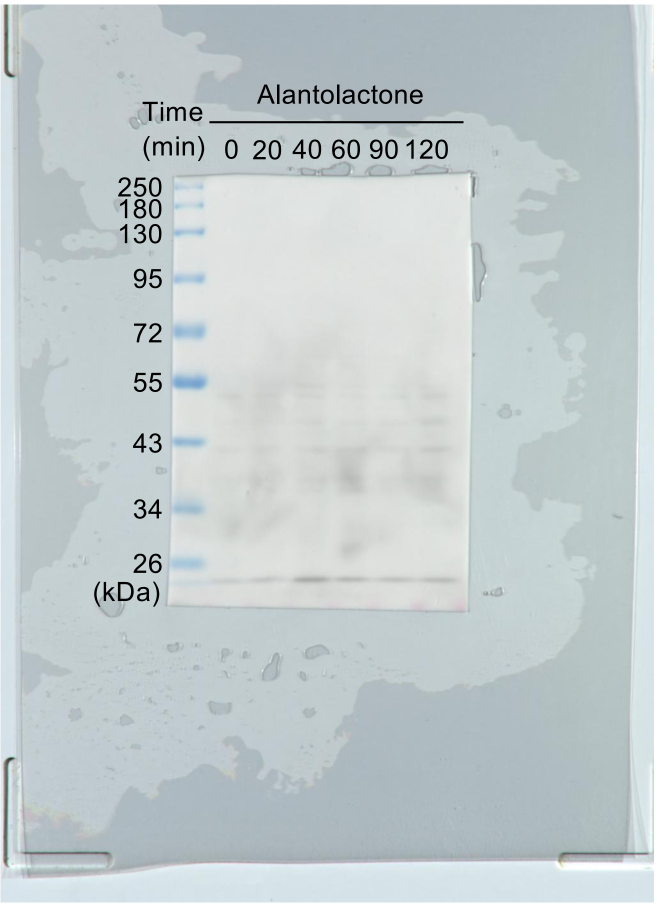

WB: Phospho-p38 MAPK

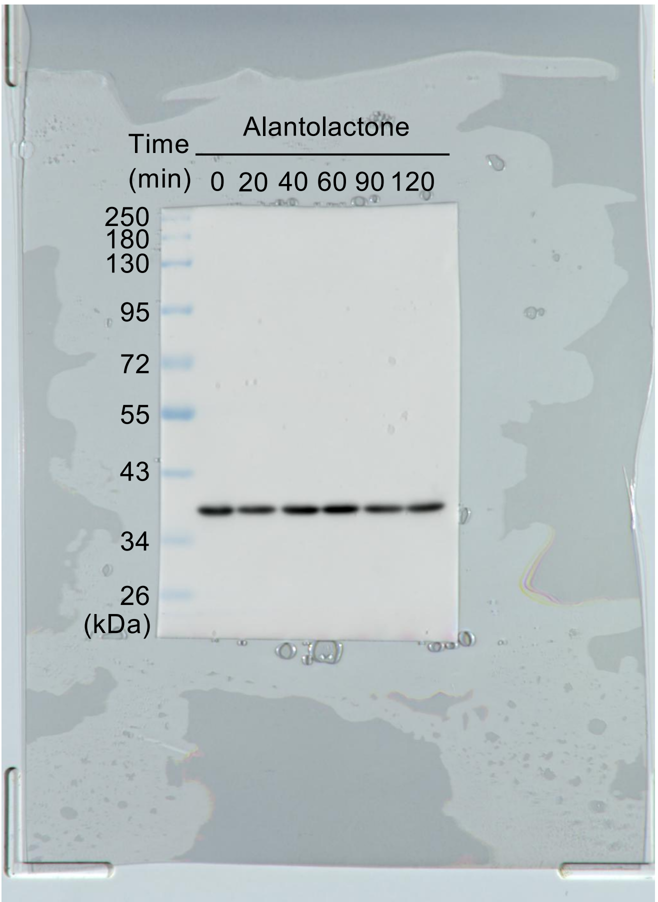

WB: GAPDH (reprobed)

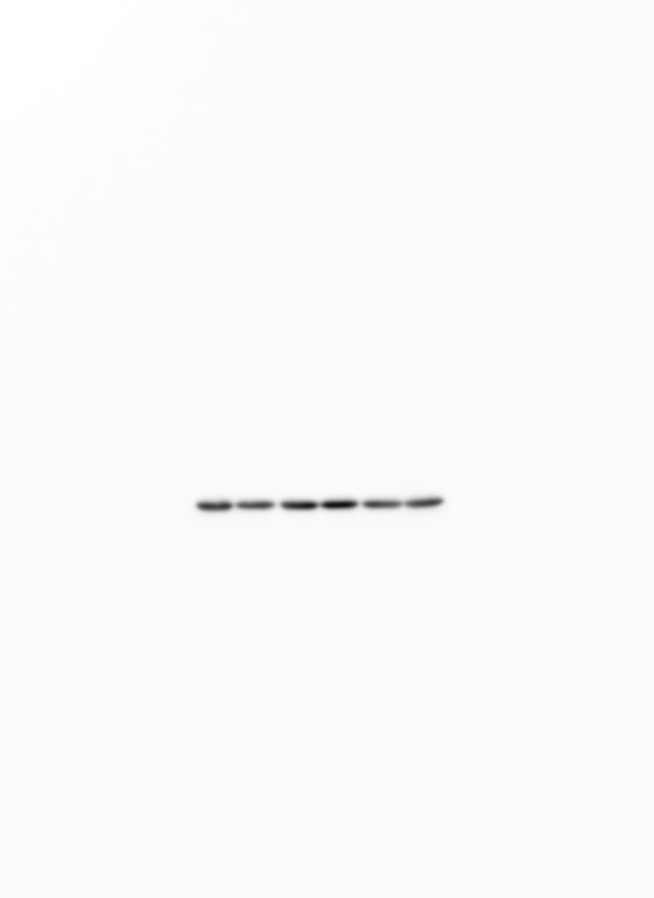

Figure S52: Original blots in Figure 6E (p38 MAPK)

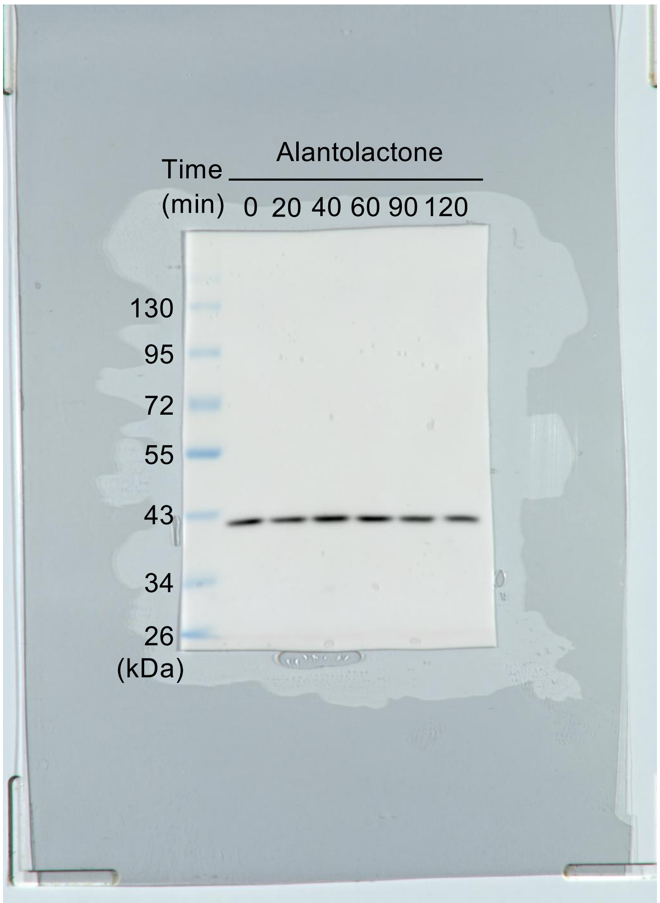

WB: p38 MAPK

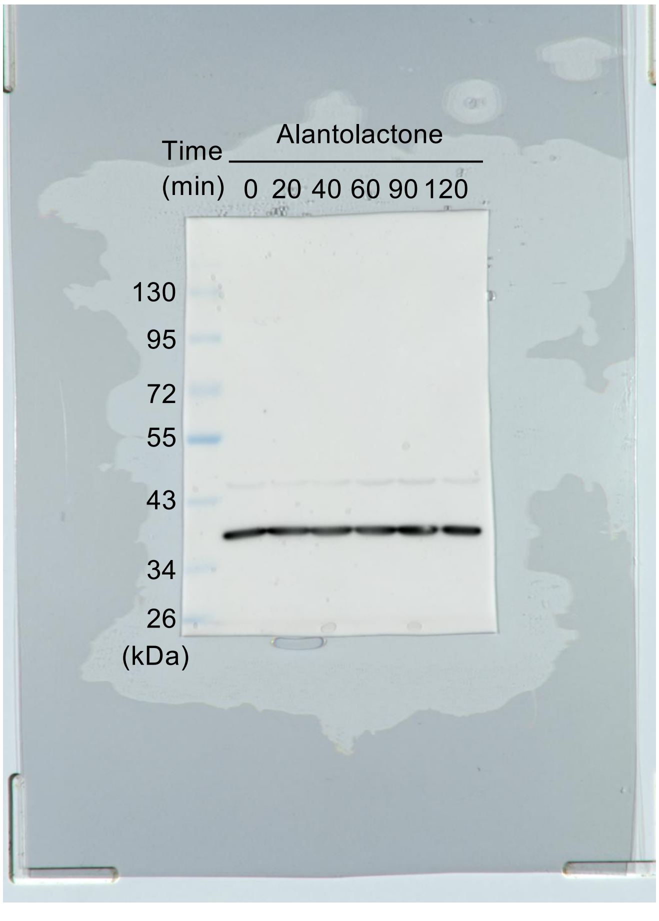

WB: GAPDH (reprobed)

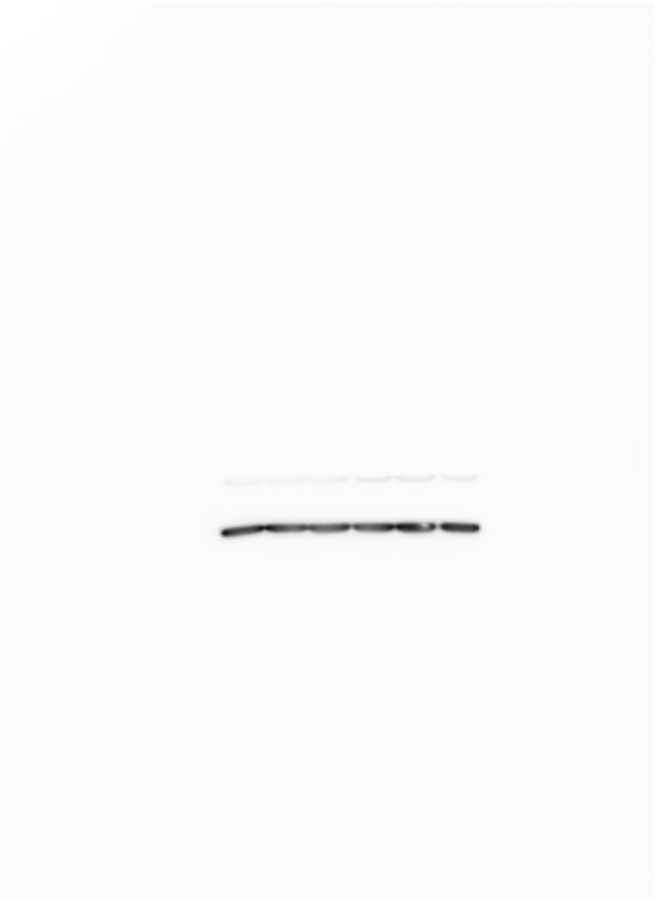

Figure S53: Original blots (1) in Figure 6F,H

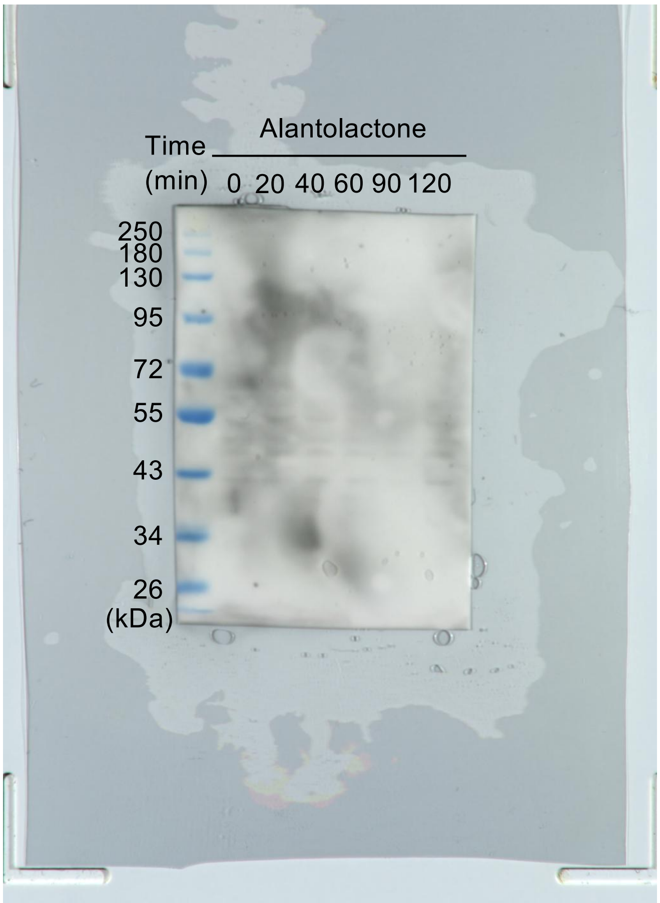

WB: Phospho-p38 MAPK

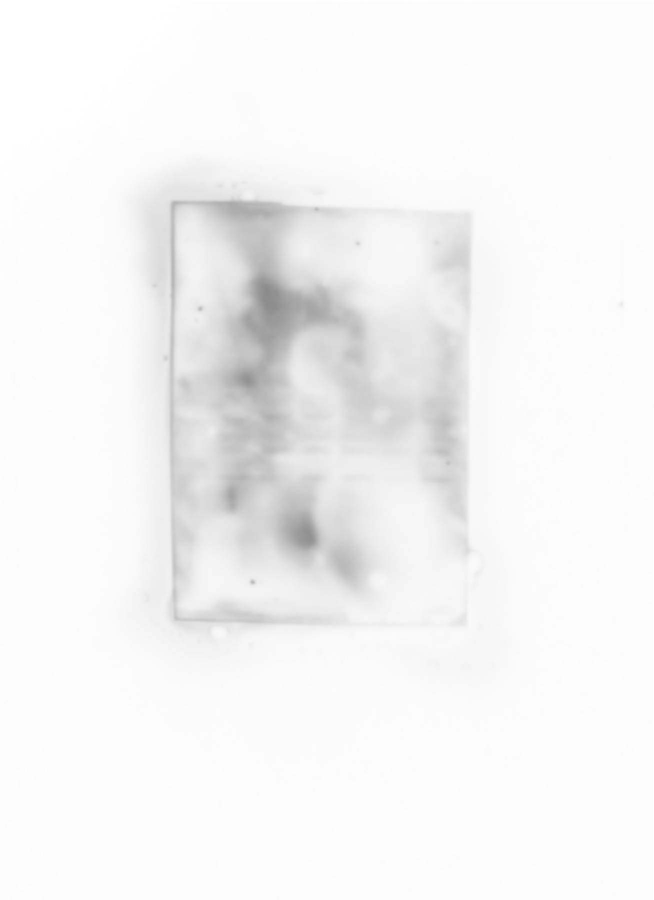

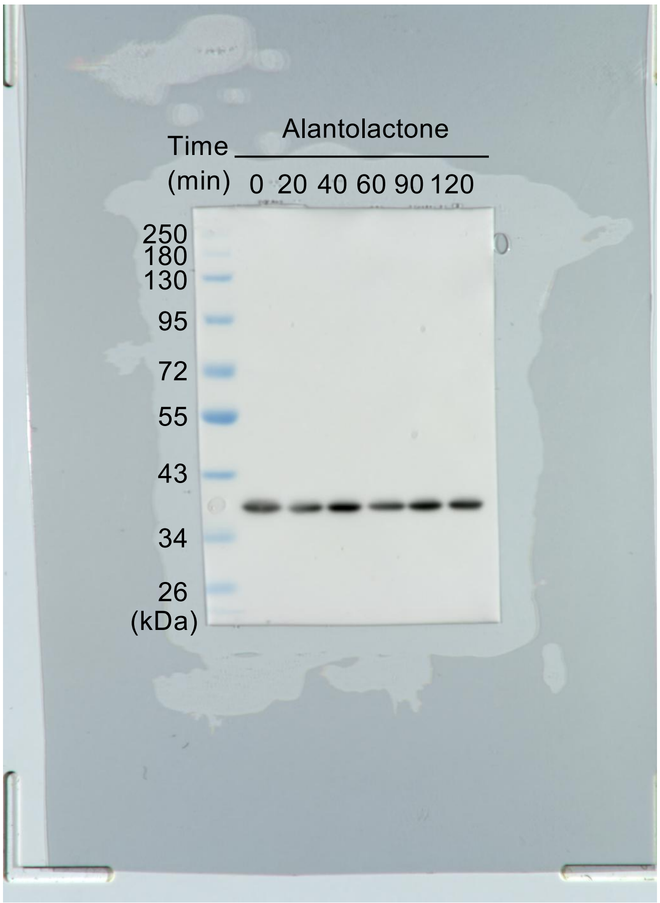

WB: GAPDH (reprobed)

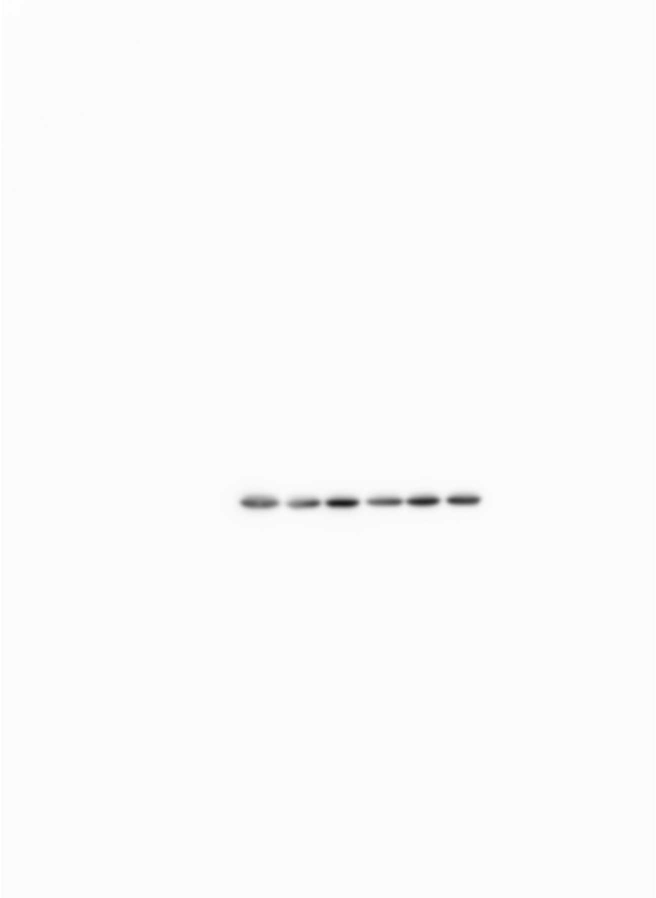

Figure S54: Original blots (2) in Figure 6F,H

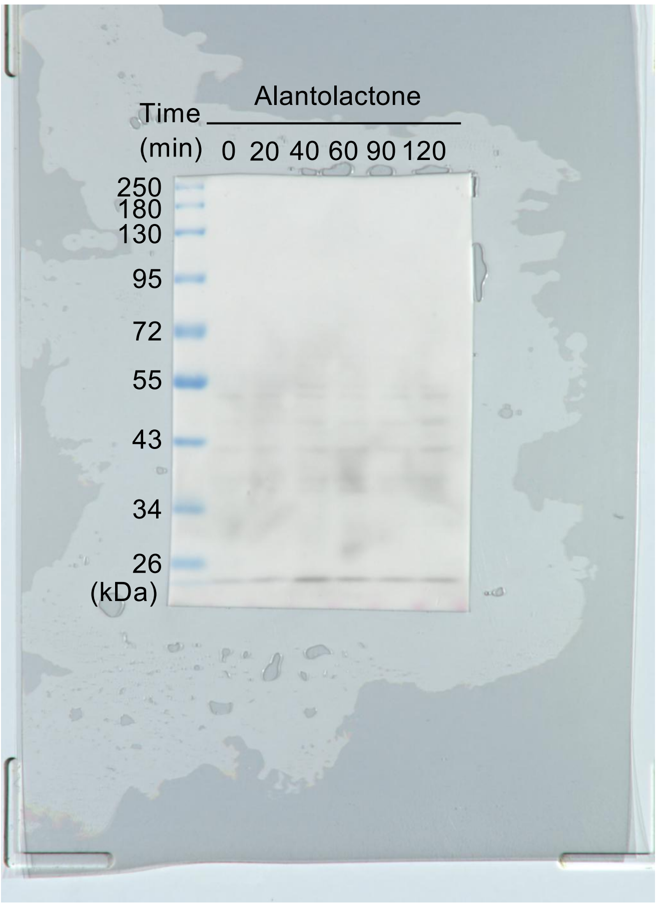

WB: Phospho-p38 MAPK

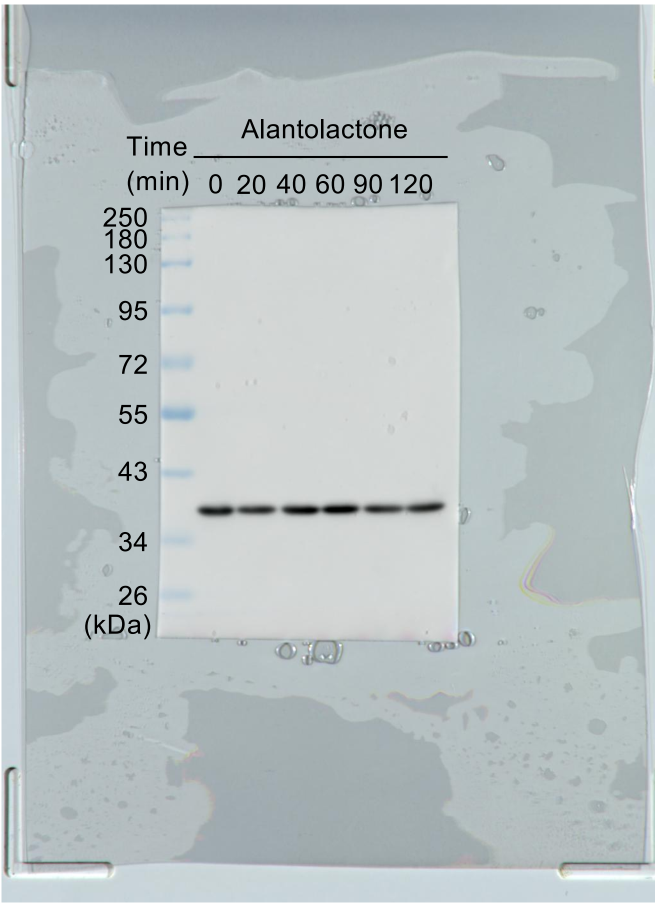

WB: GAPDH (reprobed)

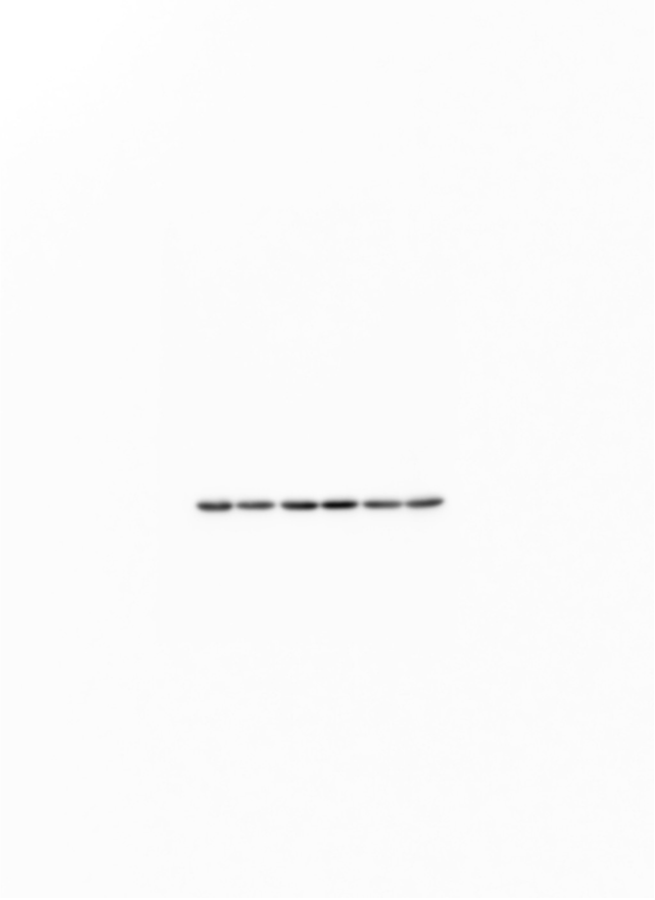

Figure S55: Original blots (3) in Figure 6F,H

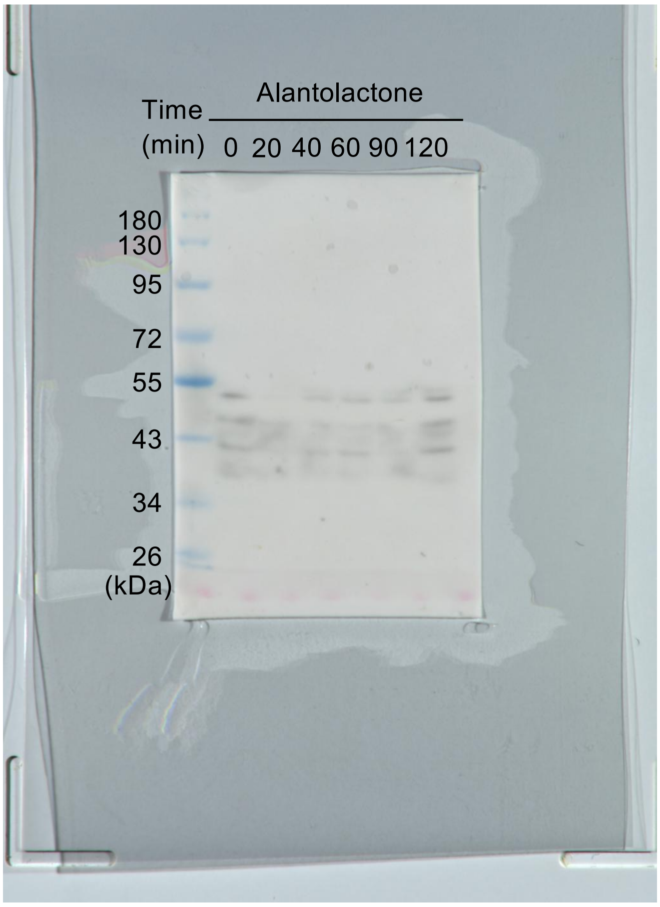

WB: Phospho-p38 MAPK

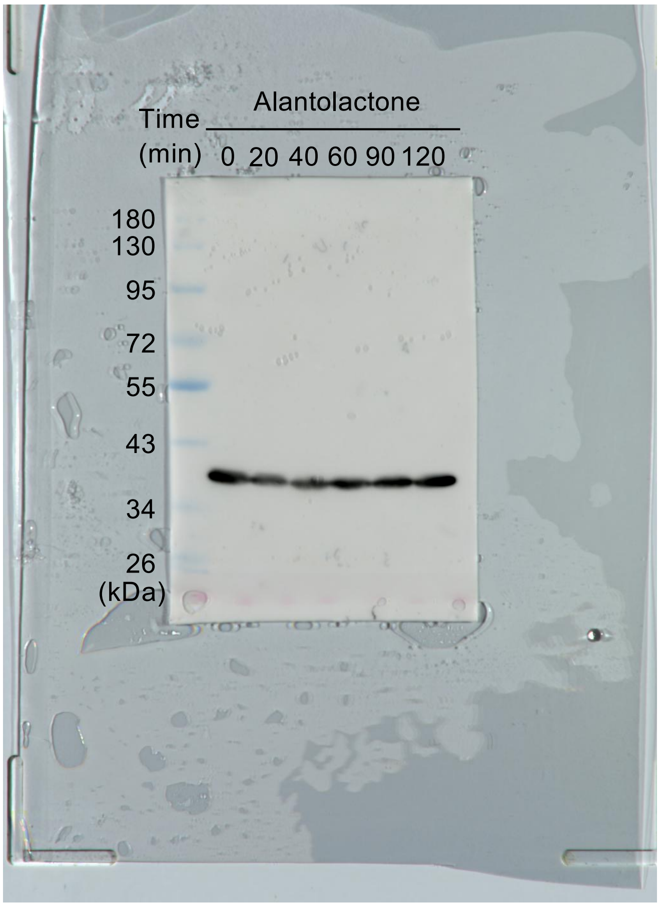

WB: GAPDH (reprobed)

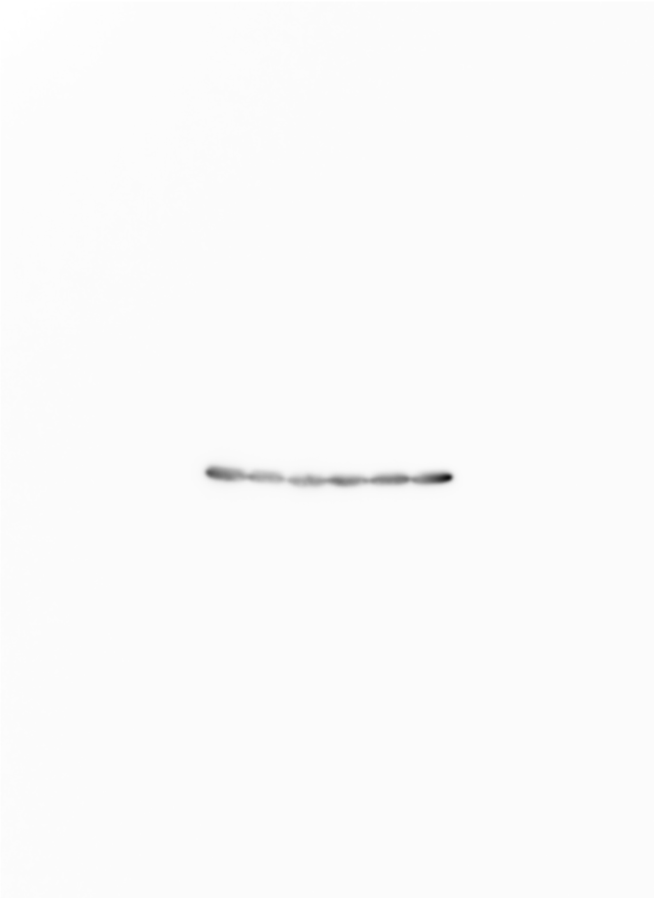

Figure S56: Original blots (1) in Figure 6G,H

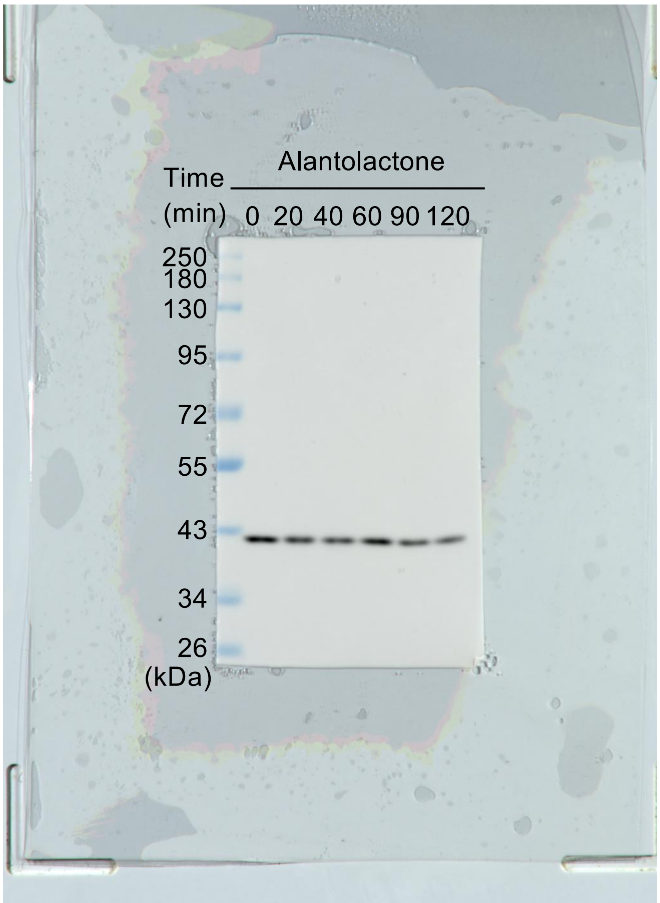

WB: p38 MAPK

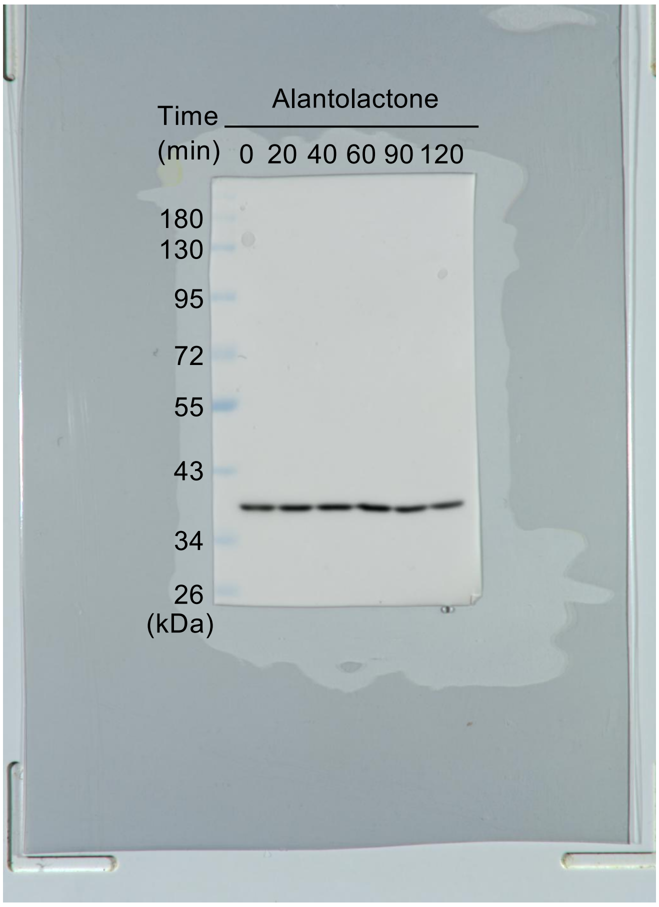

WB: GAPDH (reprobed)

Figure S57: Original blots (2) in Figure 6G,H

WB: p38 MAPK

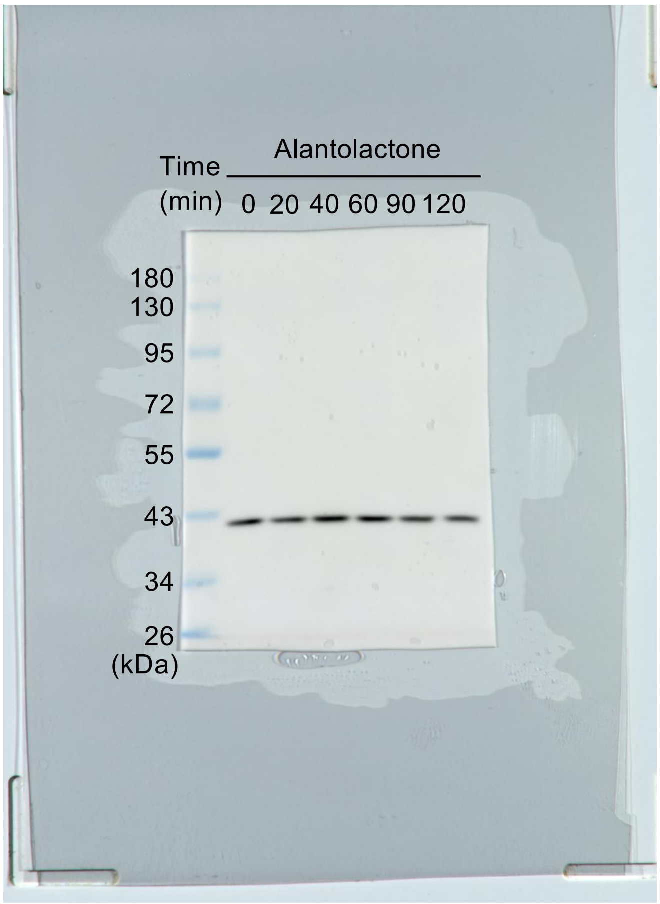

WB: GAPDH (reprobed)

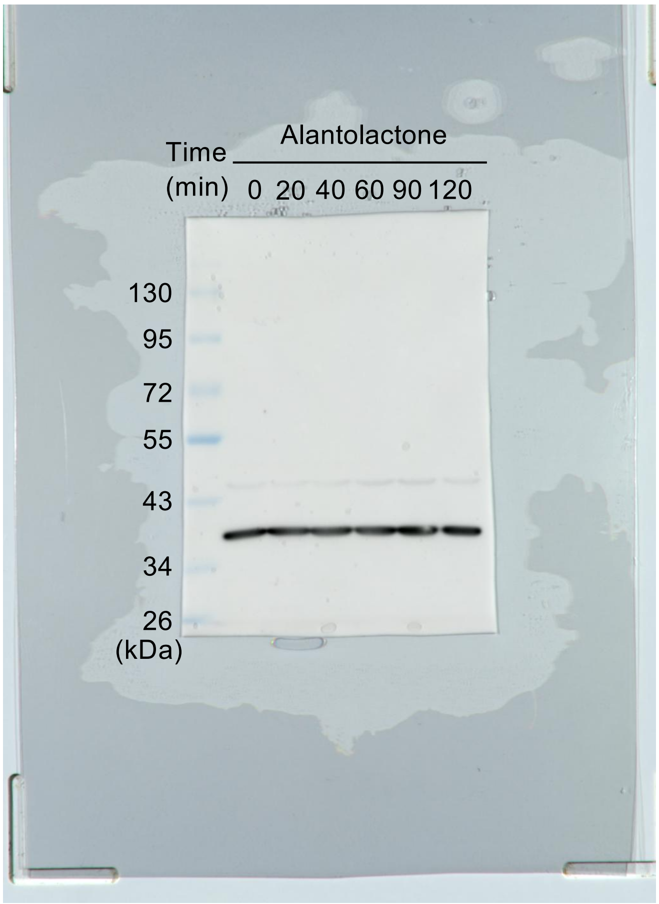

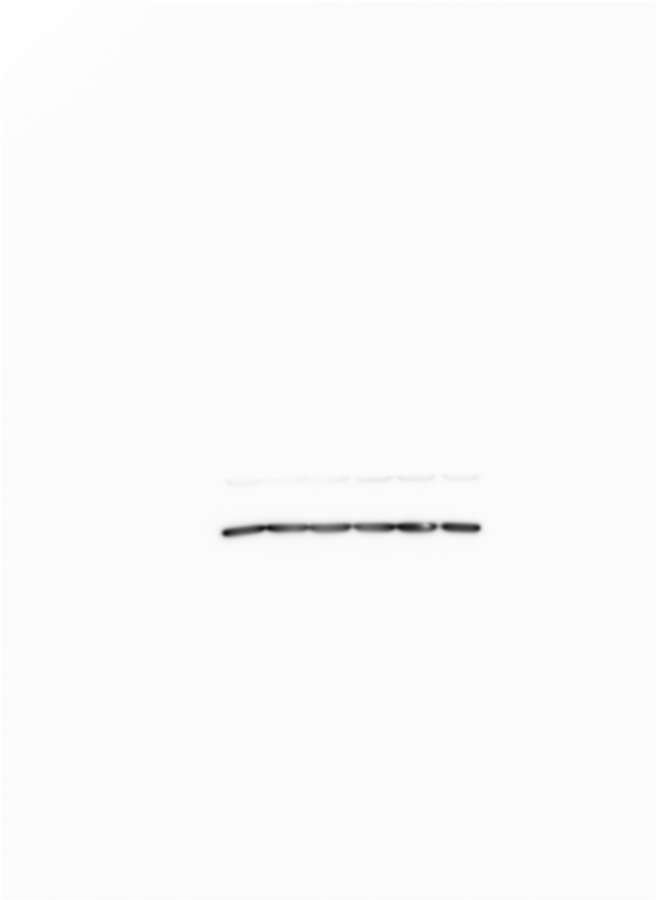

Figure S58: Original blots (3) in Figure 6G,H

WB: p38 MAPK

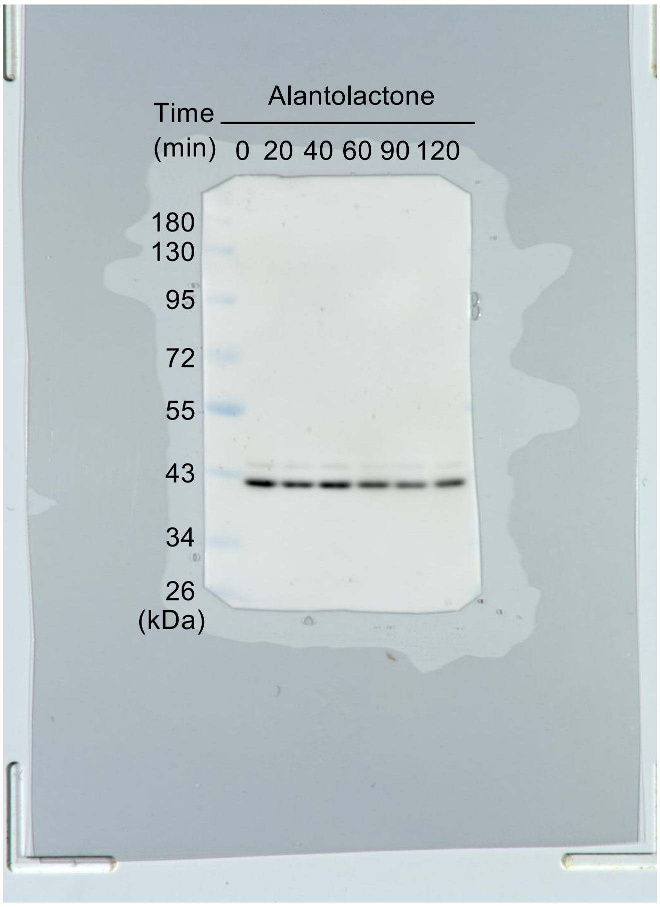

WB: GAPDH (reprobed)

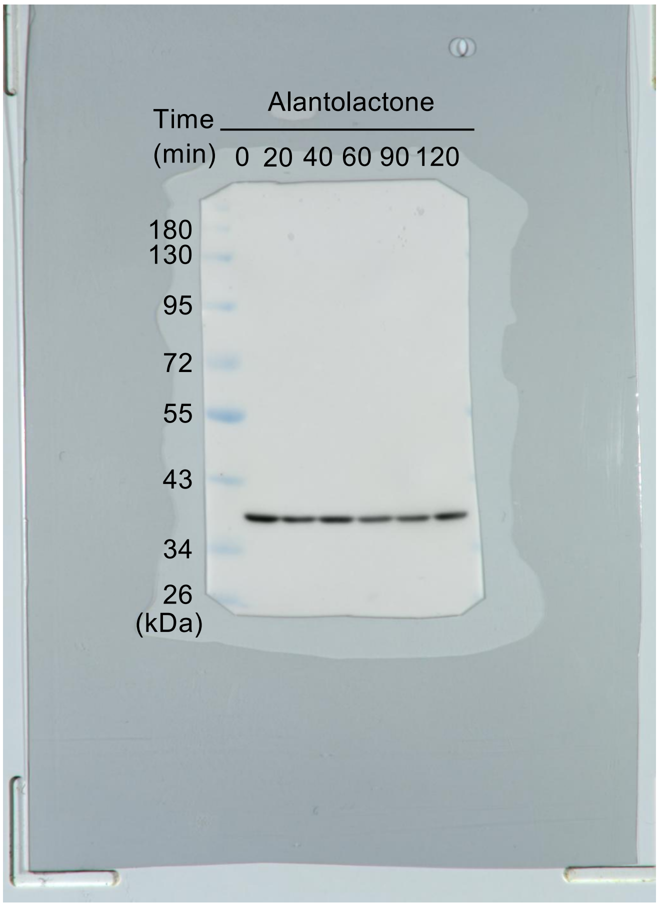

Figure S59: Original blots in Figure 6I (Phospho-JNK)

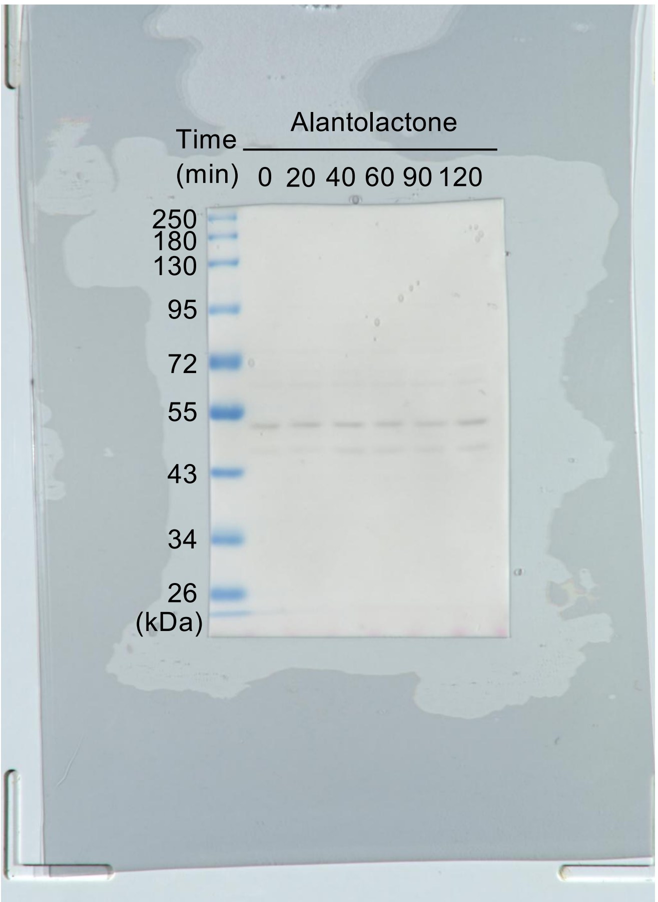

WB: Phospho-JNK

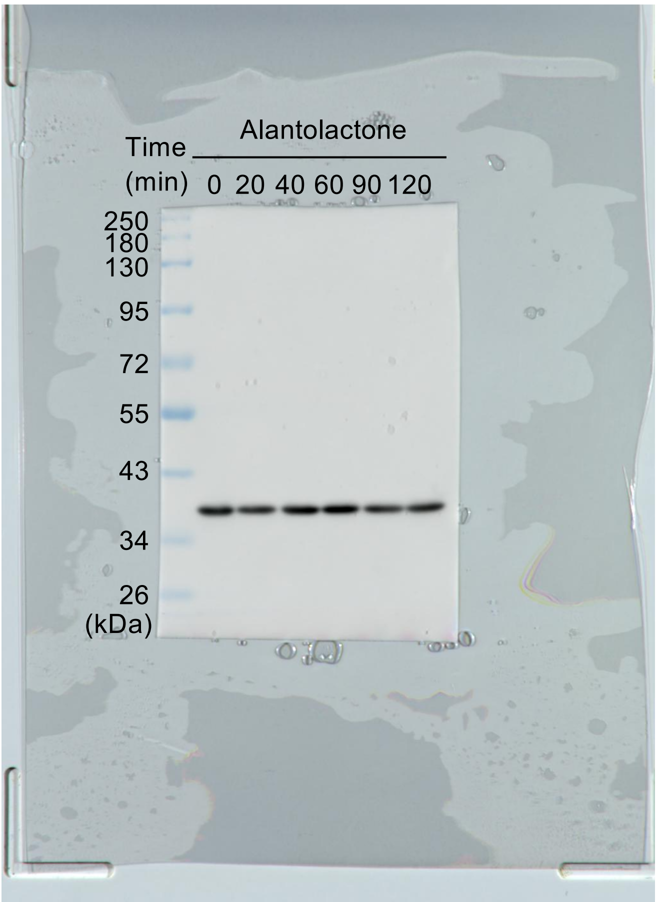

WB: GAPDH (reprobed)

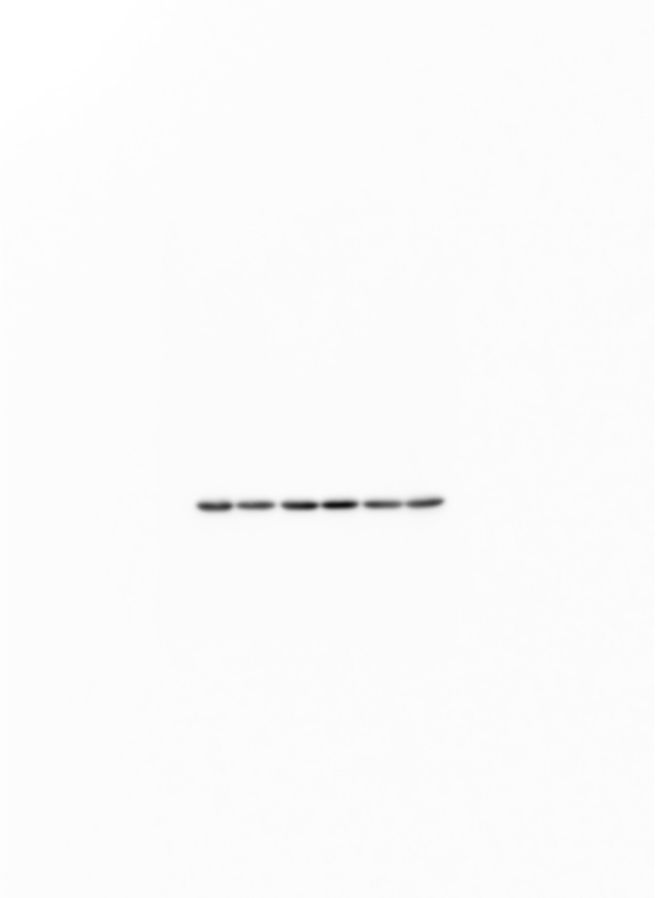

Figure S60: Original blots in Figure 6I (JNK)

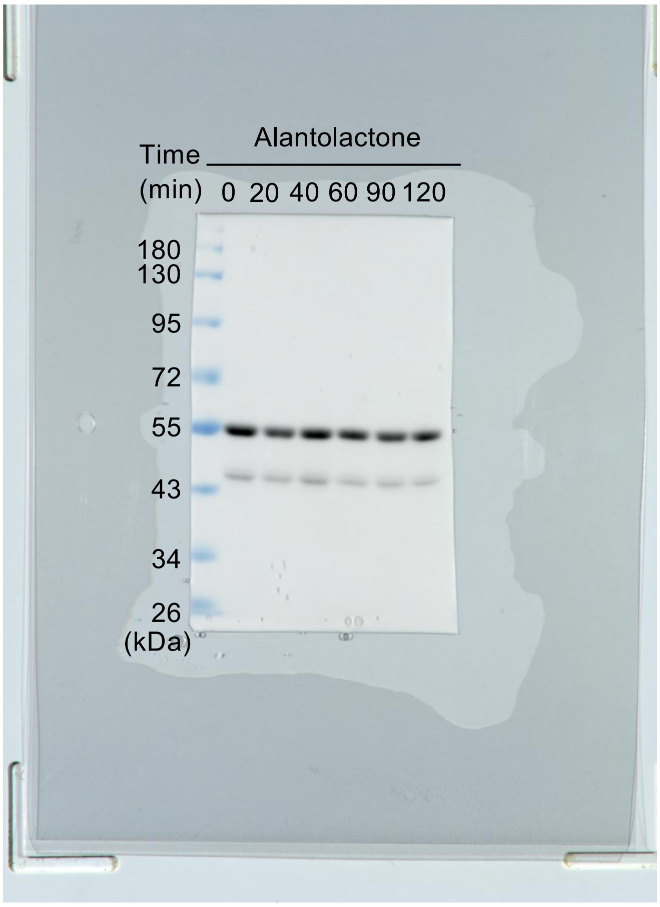

WB: JNK

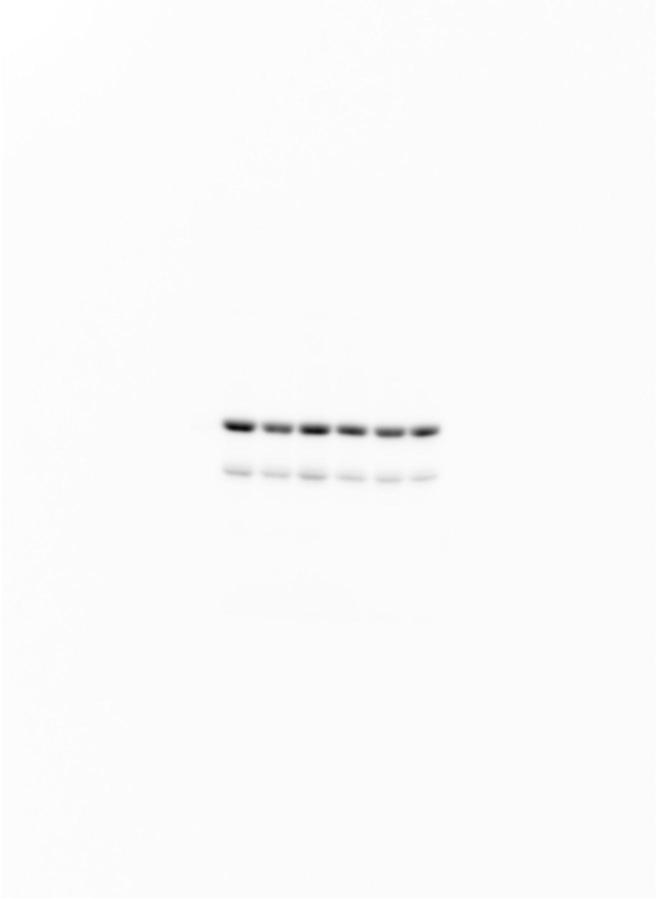

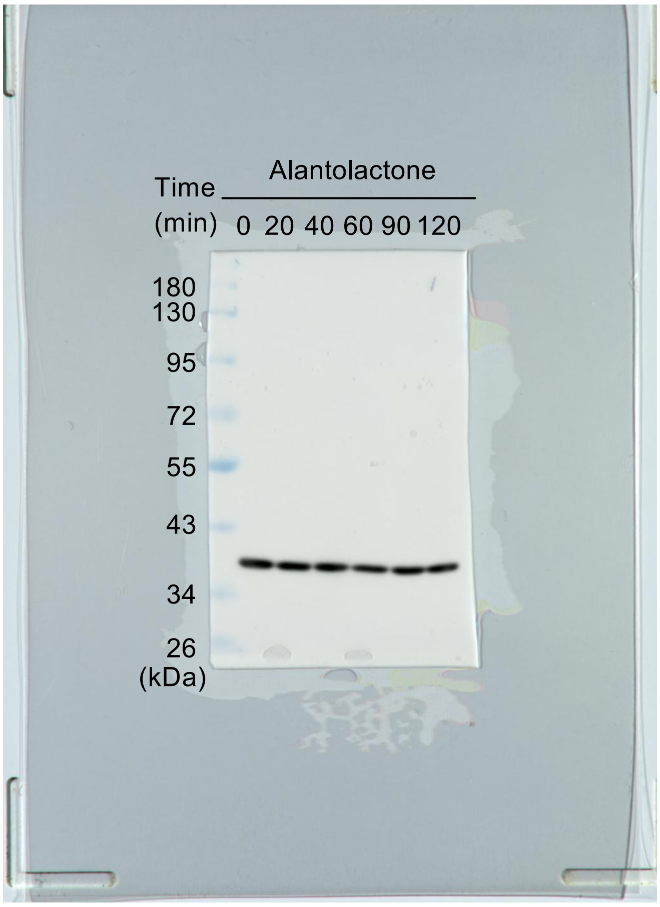

WB: GAPDH (reprobed)

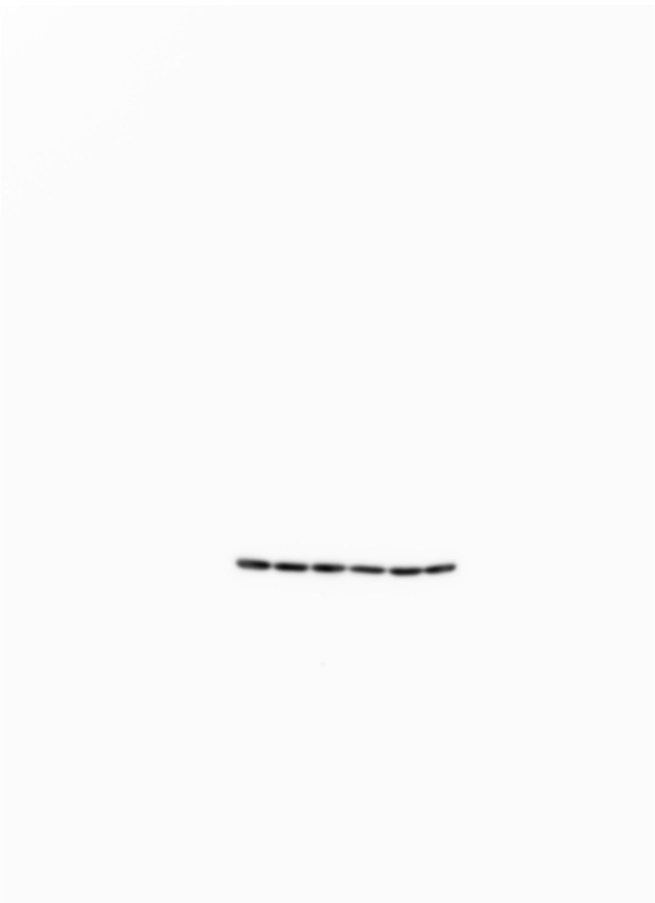

Figure S61: Original blots (1) in Figure 6J,L,M,O

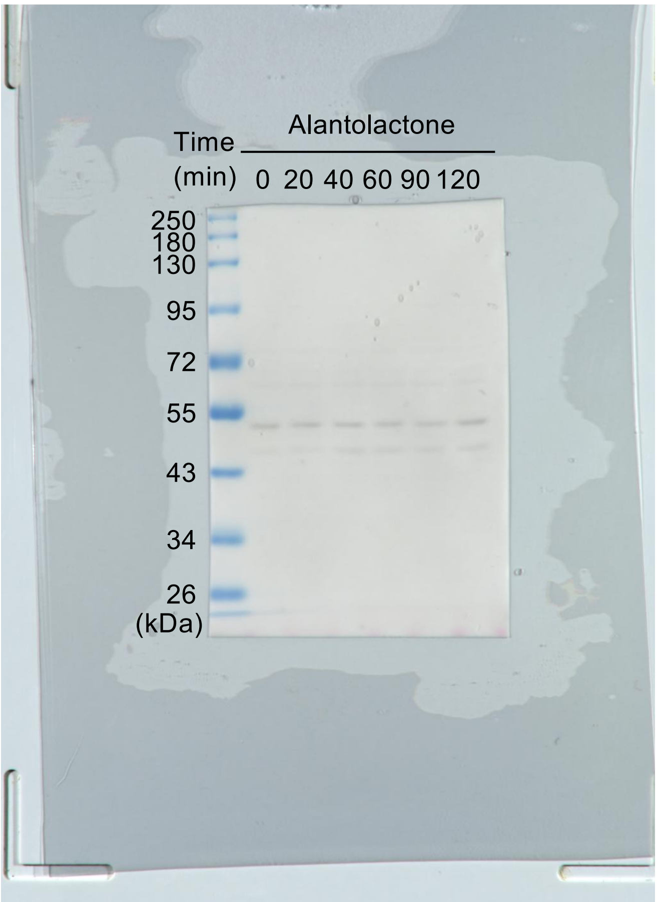

WB: Phospho-JNK

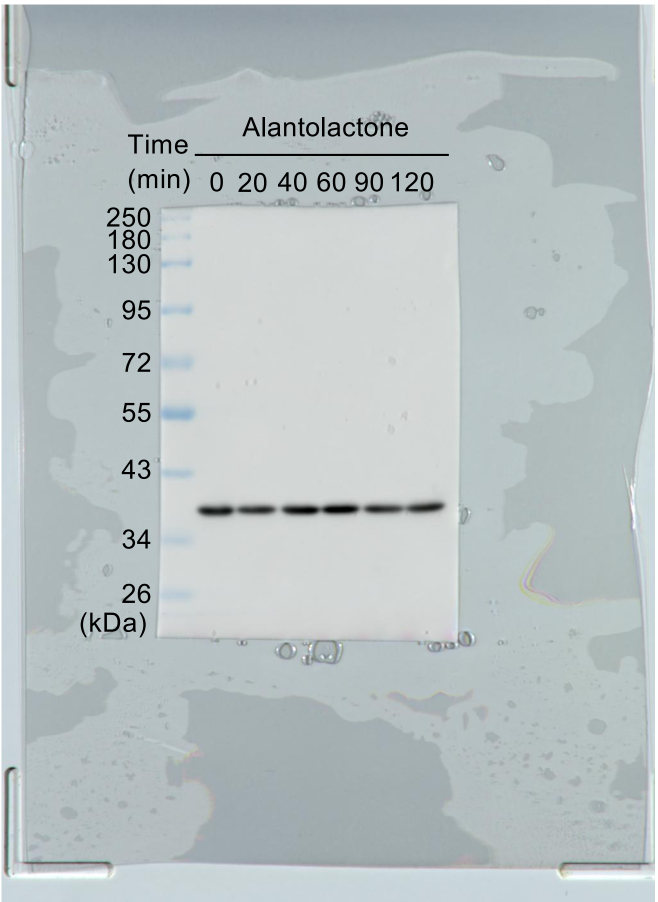

WB: GAPDH (reprobed)

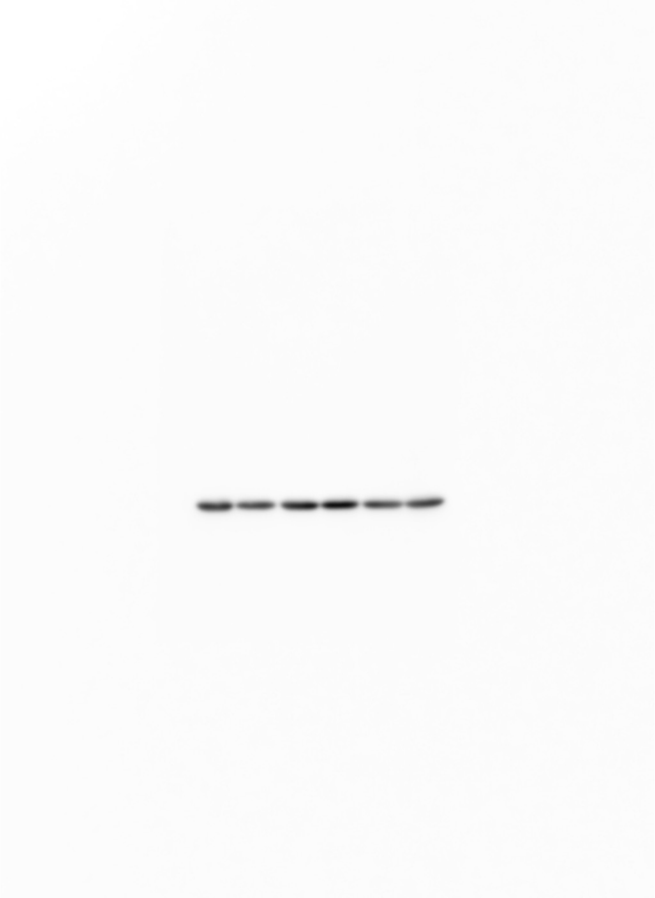

Figure S62: Original blots (2) in Figure 6J,L,M,O

WB: Phospho-JNK

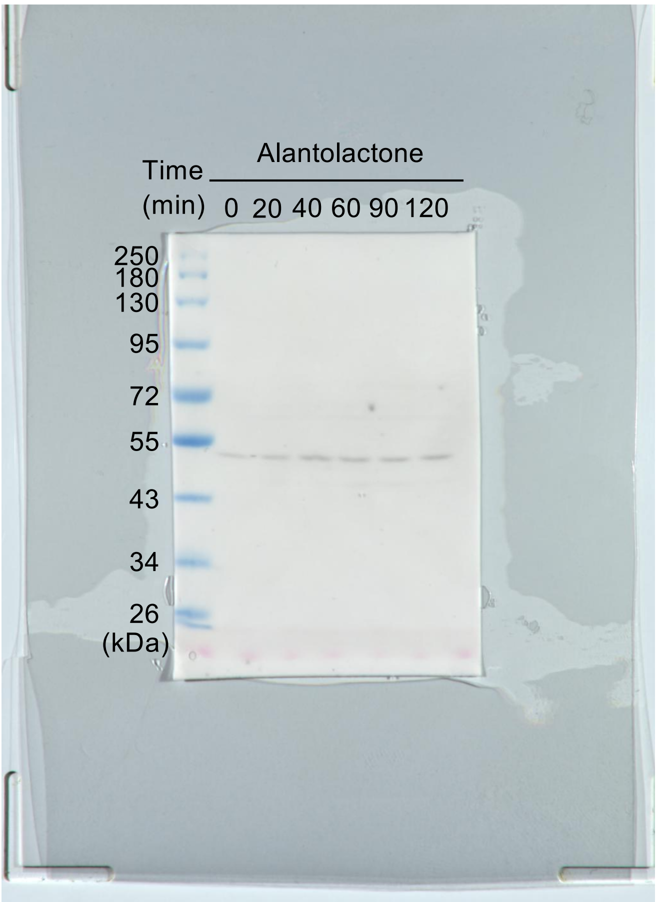

WB: GAPDH (reprobed)

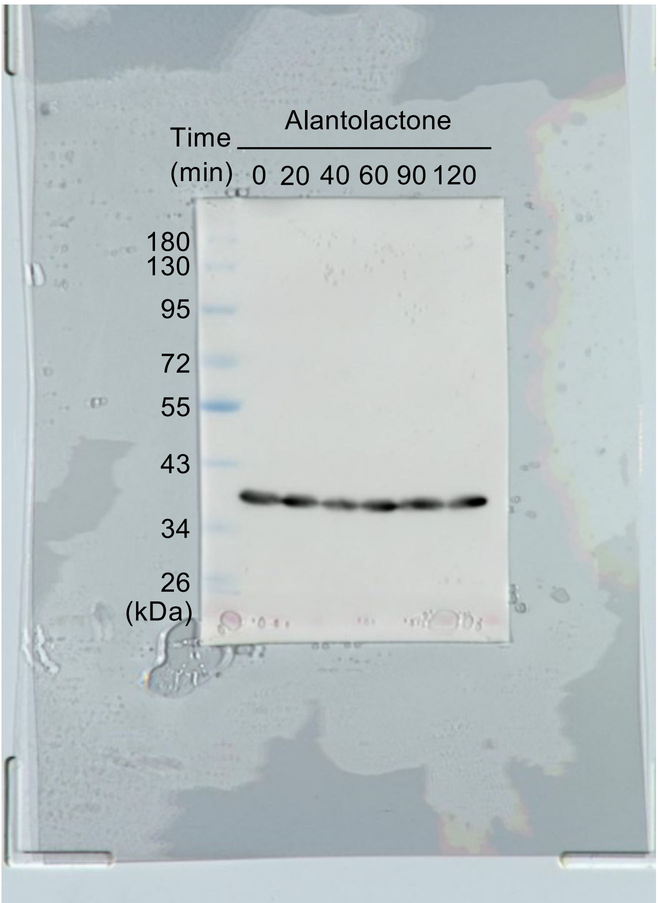

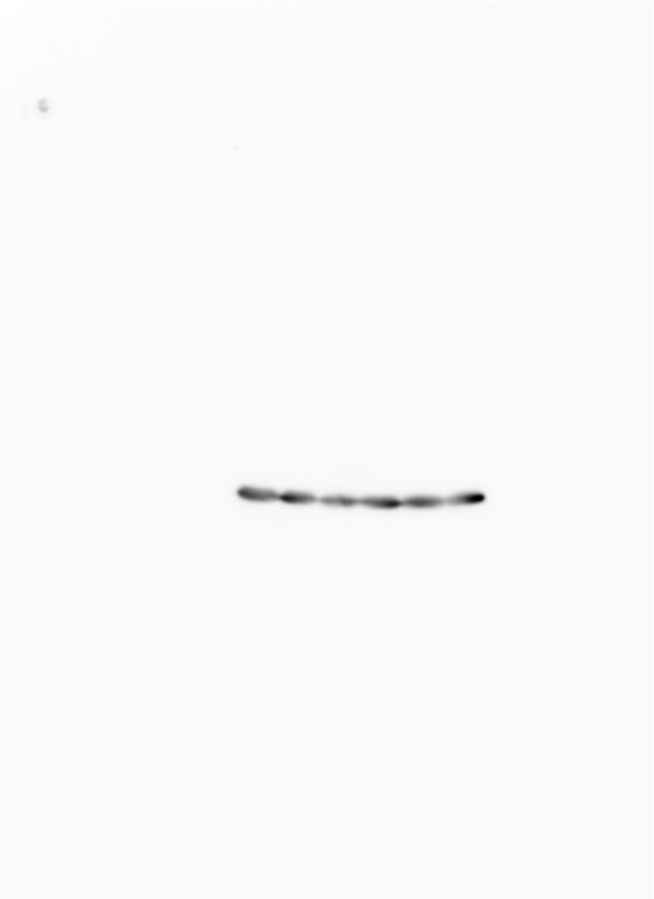

Figure S63: Original blots (3) in Figure 6J,L,M,O

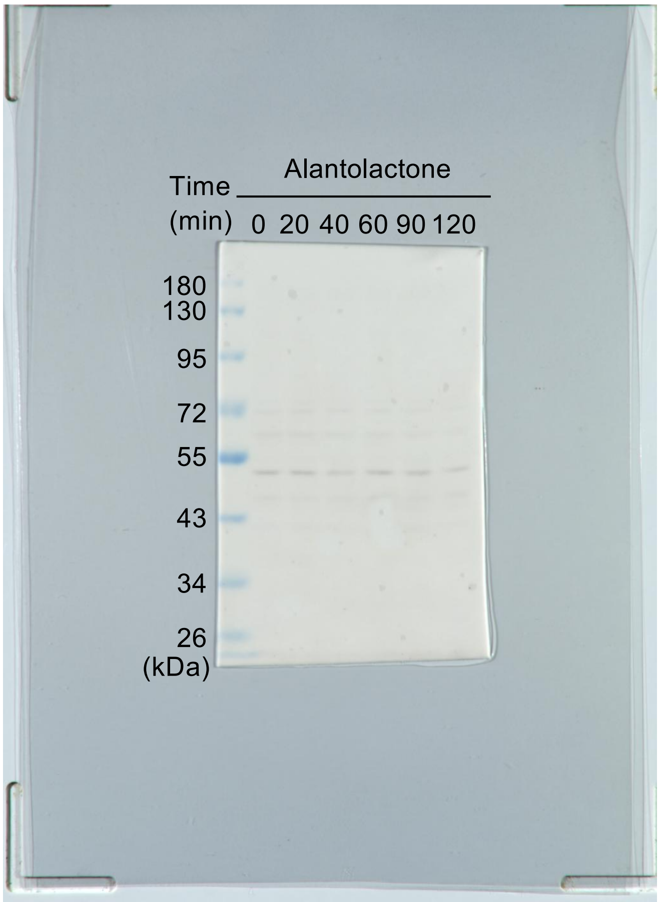

WB: Phospho-JNK

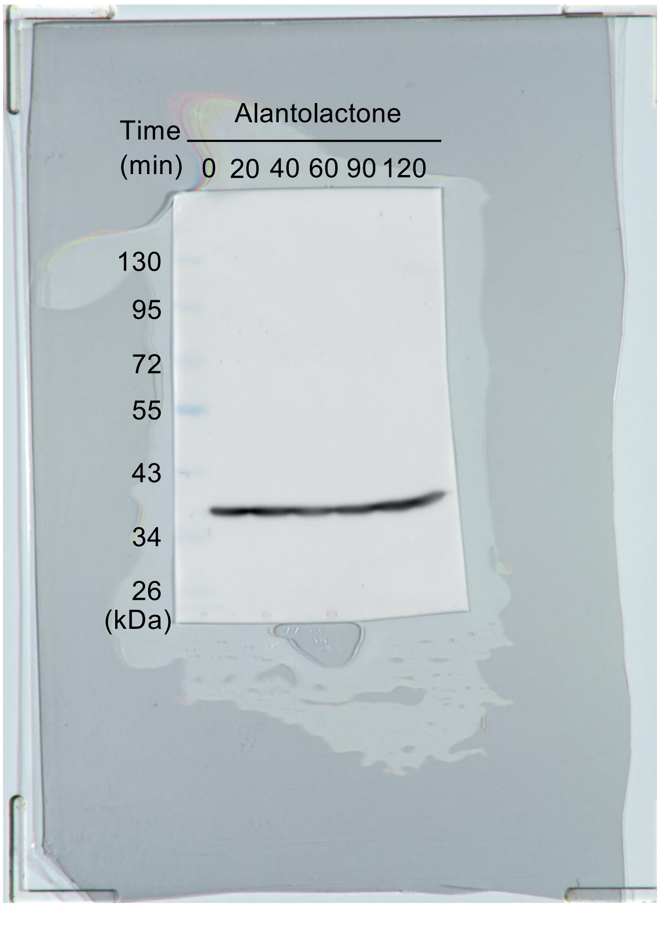

WB: GAPDH (reprobed)

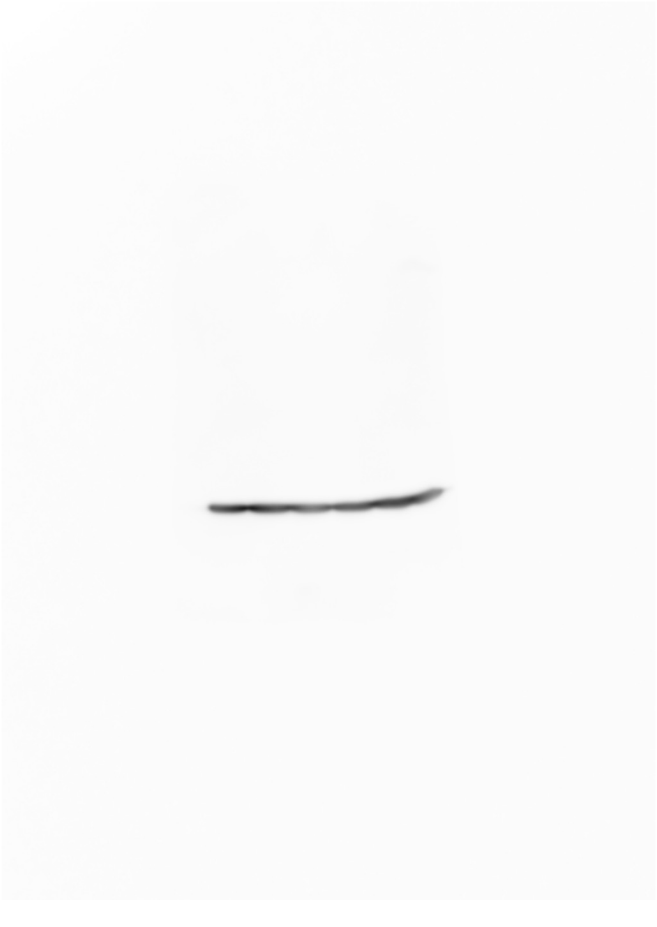

Figure S64: Original blots (1) in Figure 6K,L,N,O

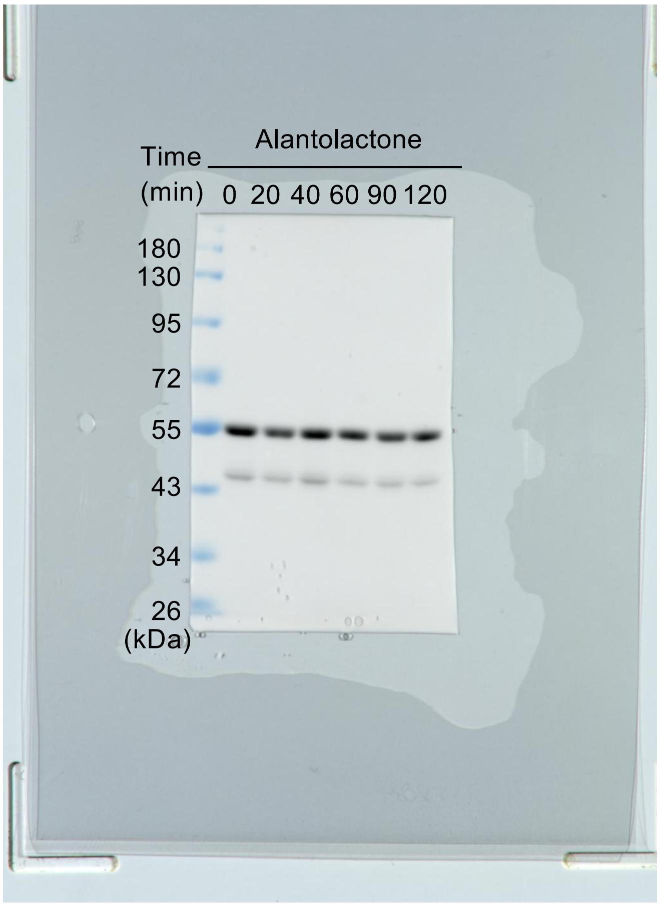

WB: JNK

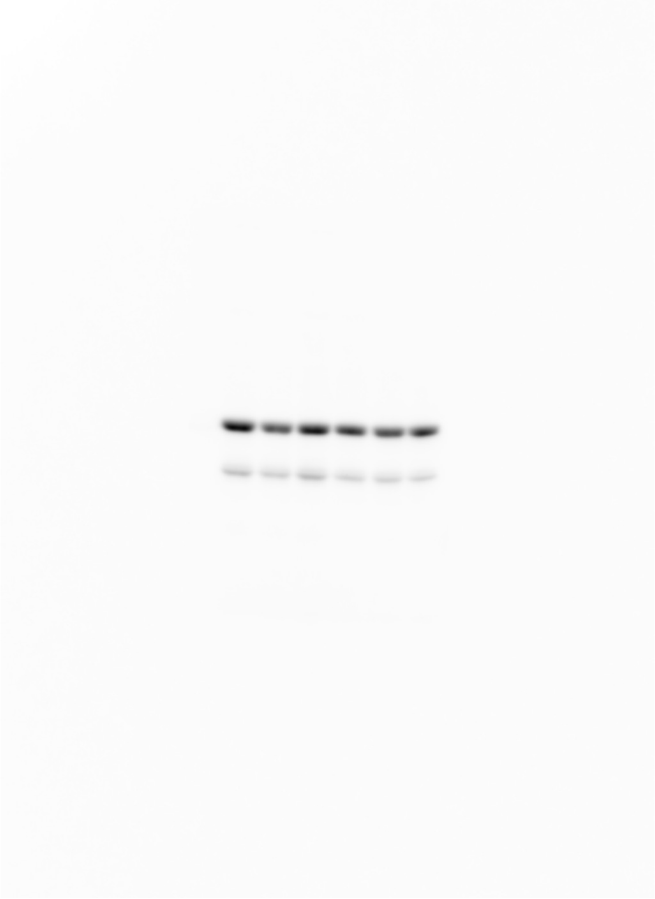

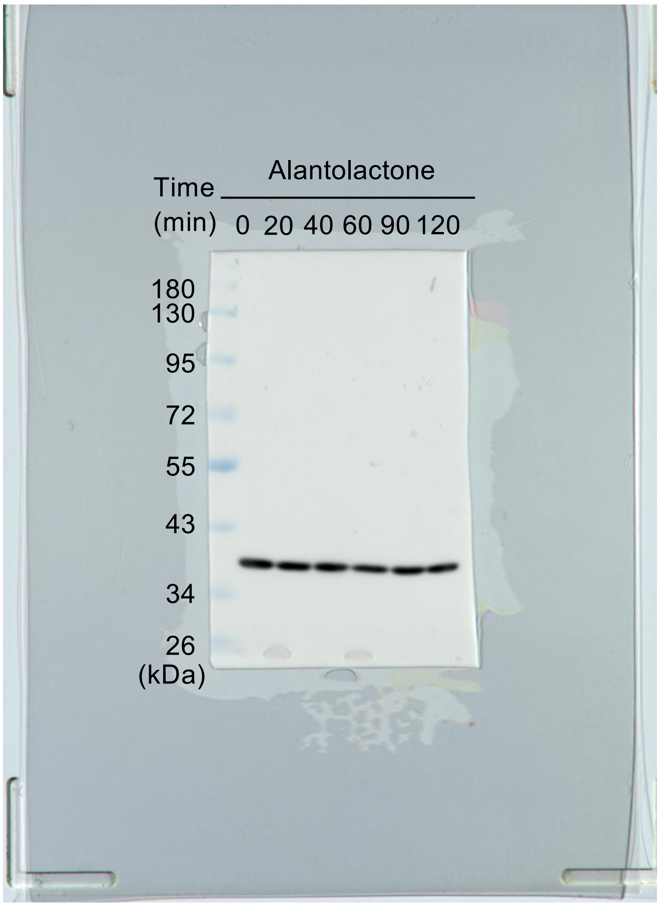

WB: GAPDH (reprobed)

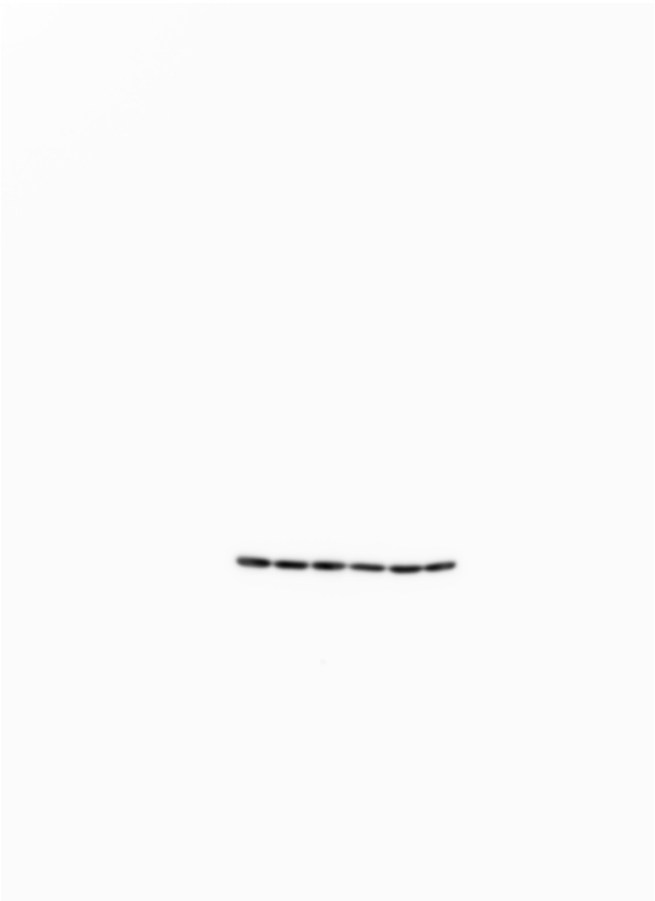

Figure S65: Original blots (2) in Figure 6K,L,N,O

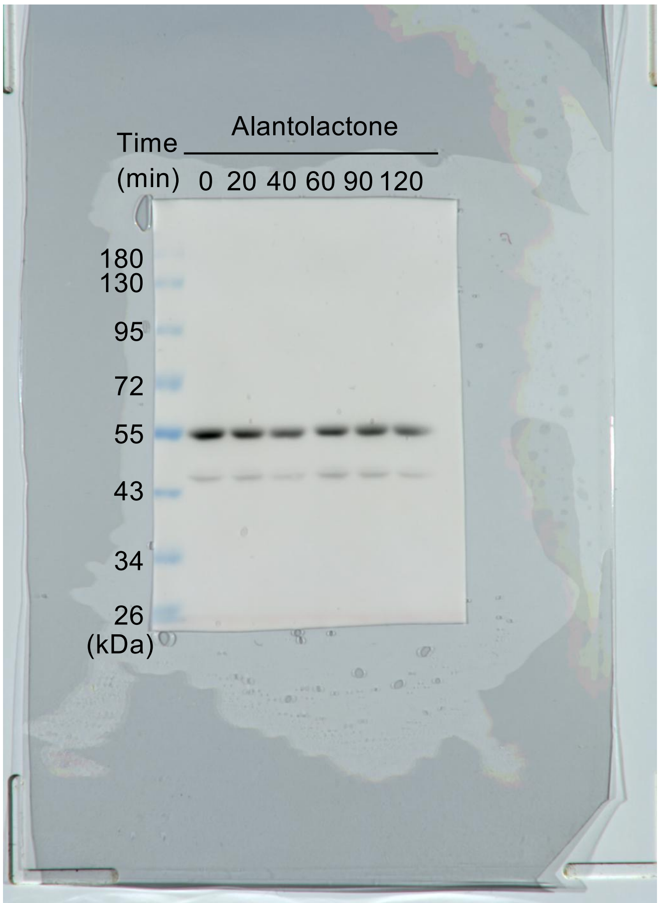

WB: JNK

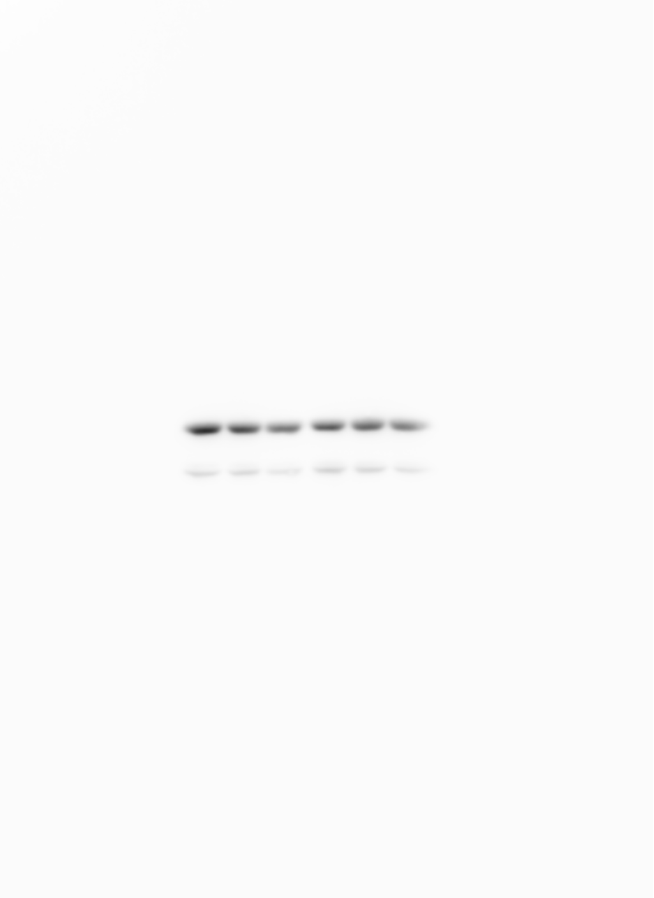

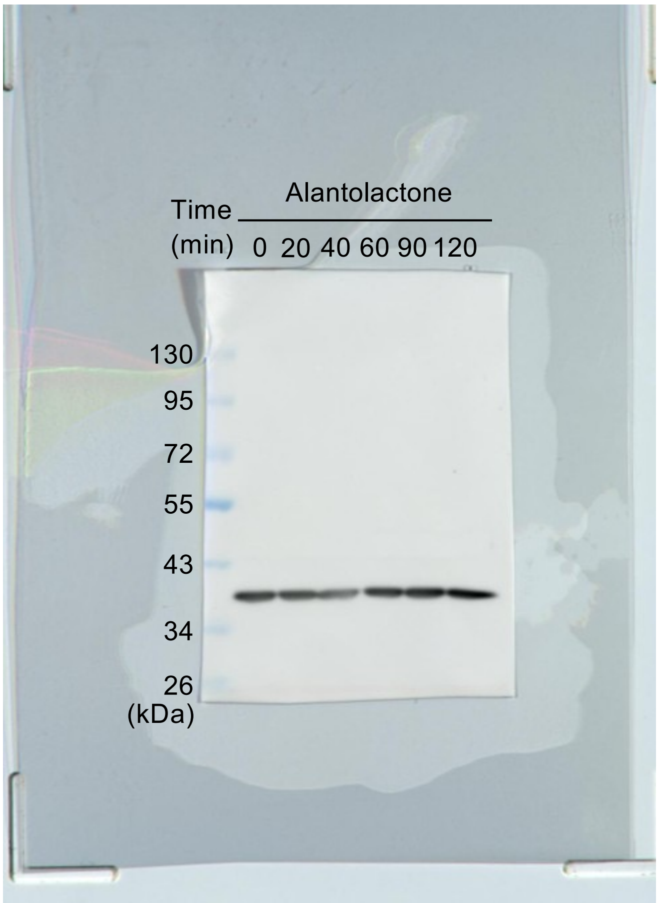

WB: GAPDH (reprobed)

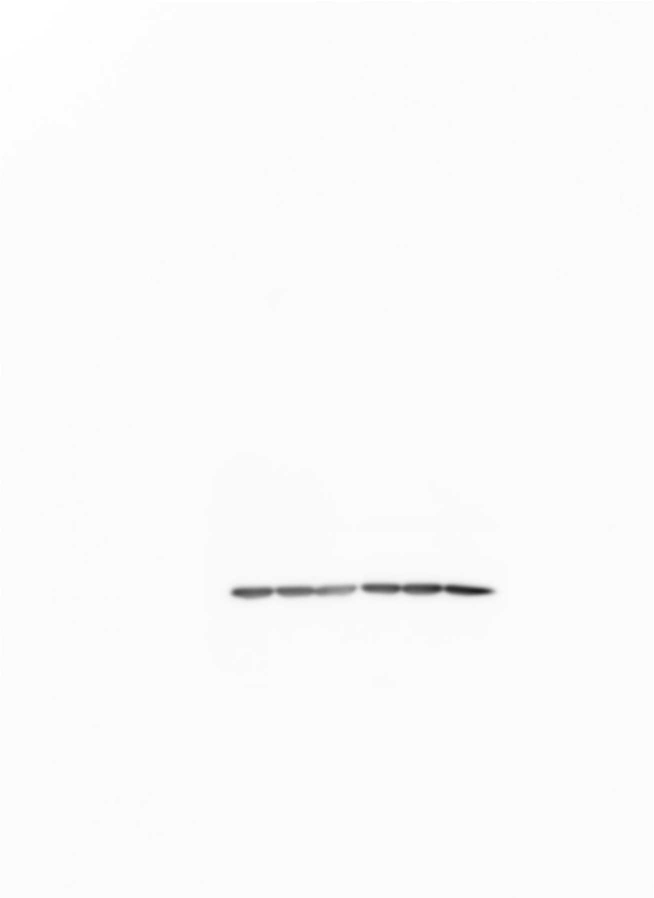

Figure S66: Original blots (3) in Figure 6K,L,N,O

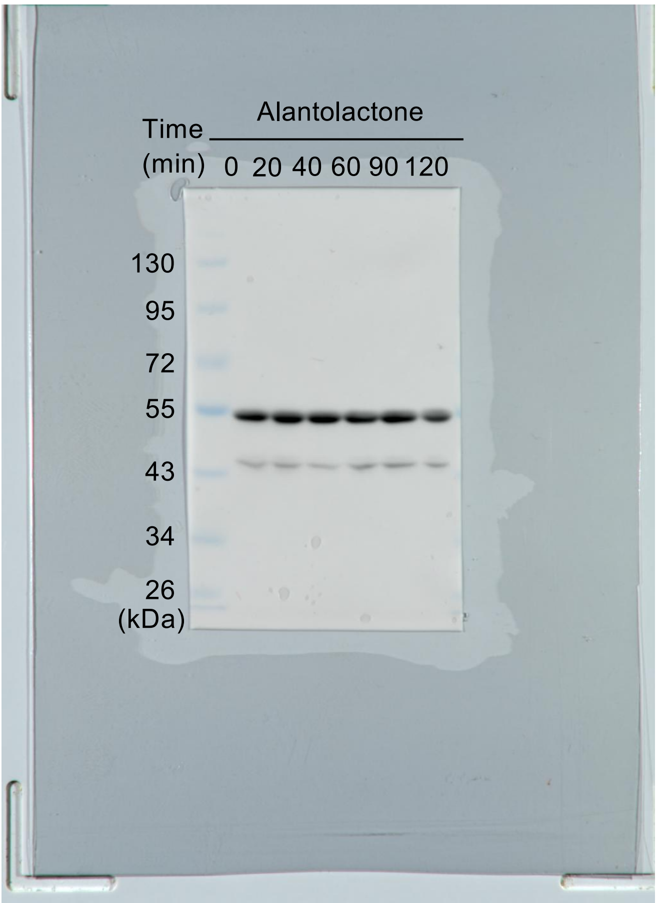

WB: JNK

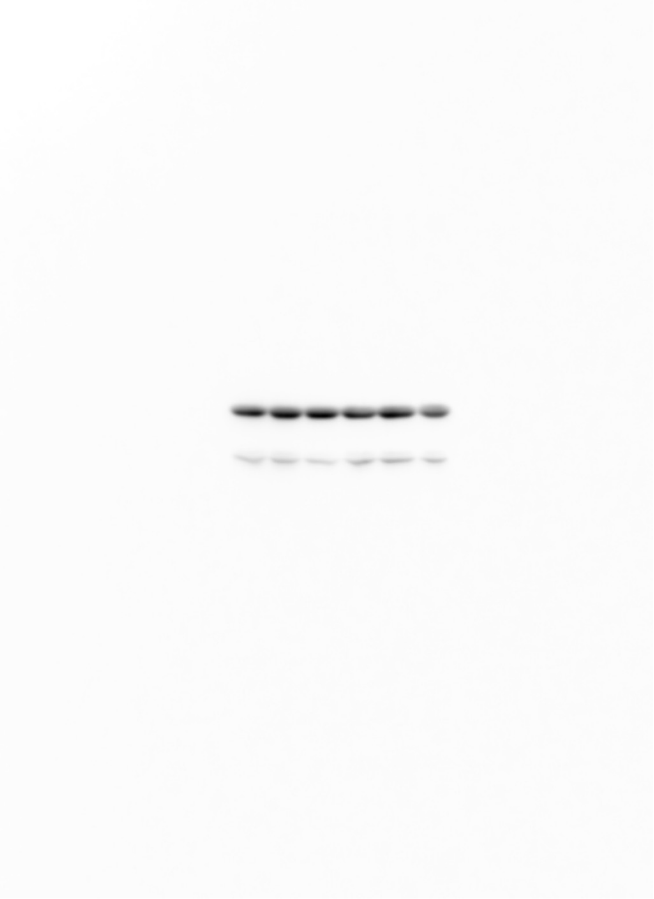

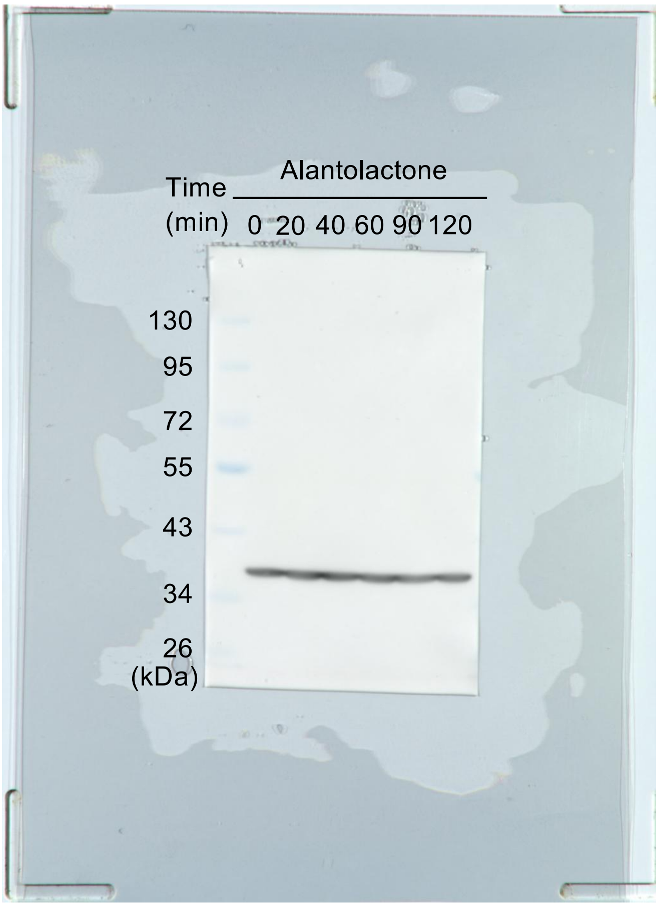

WB: GAPDH (reprobed)

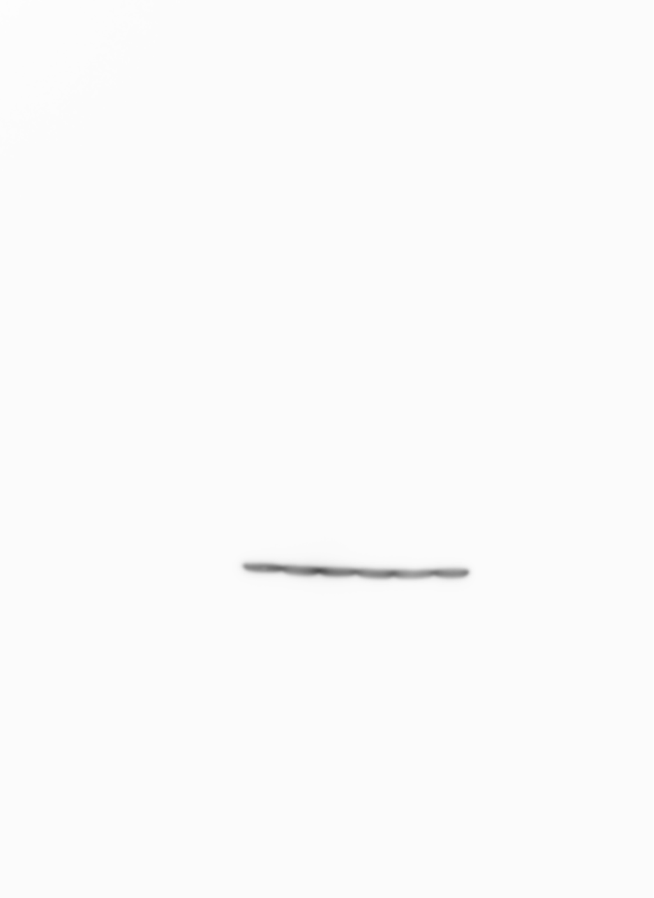

Figure S67: Original blots in Figure 7A (Phospho-RAF1)

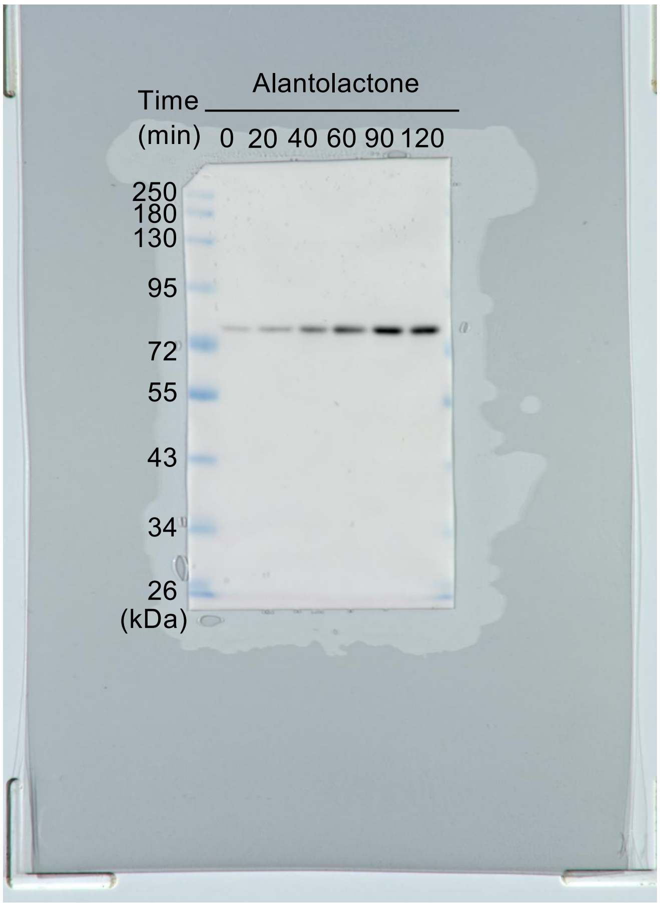

WB: Phospho-RAF1

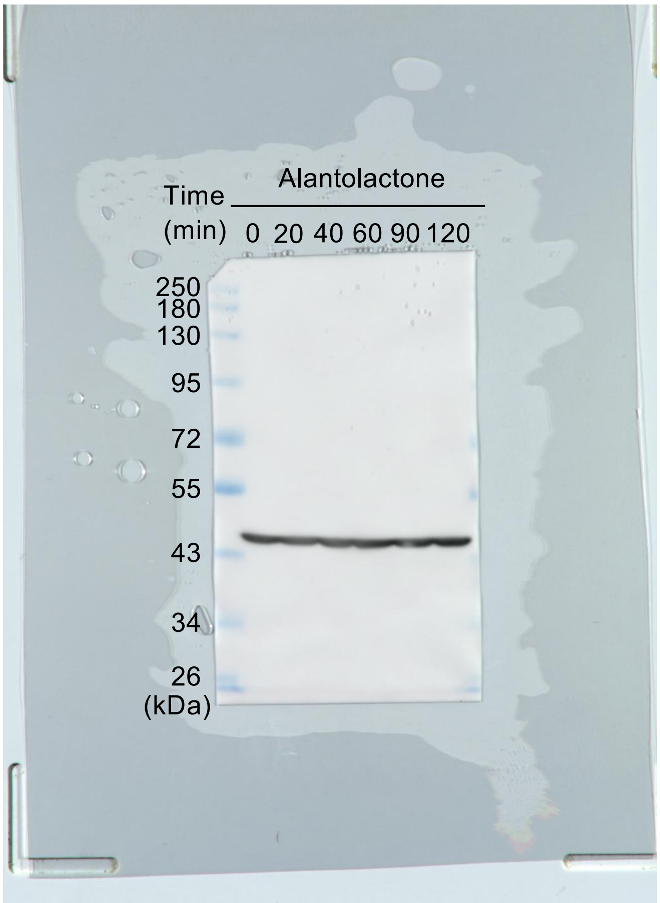

WB: β-Actin (reprobed)

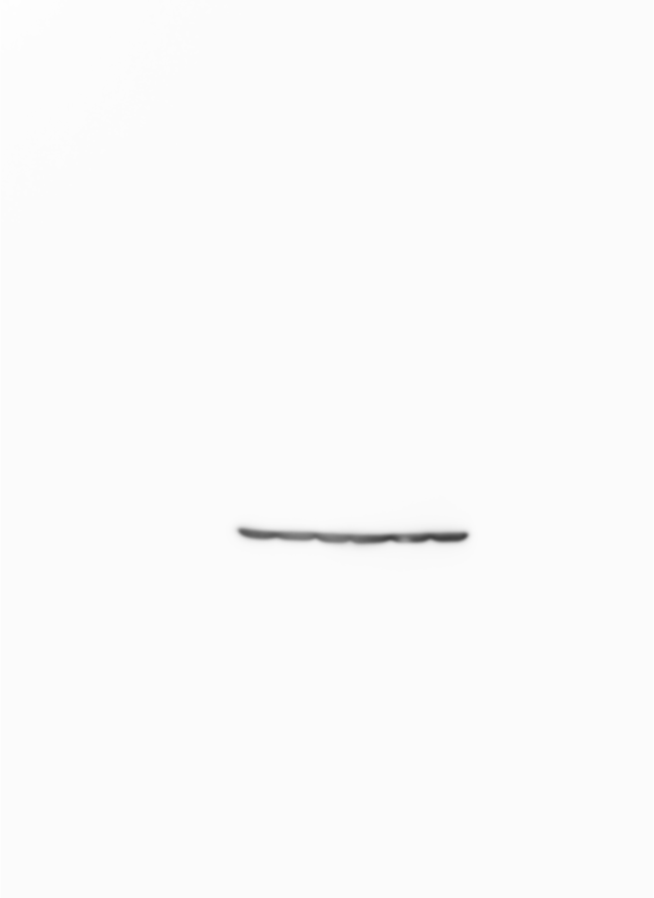

Figure S68: Original blots in Figure 7A (RAF1)

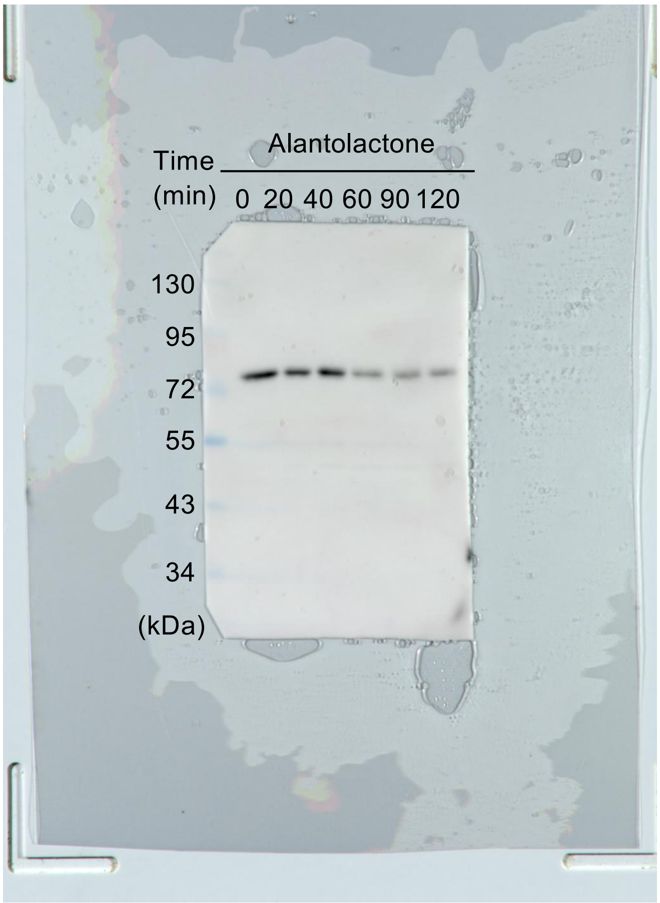

WB: RAF1

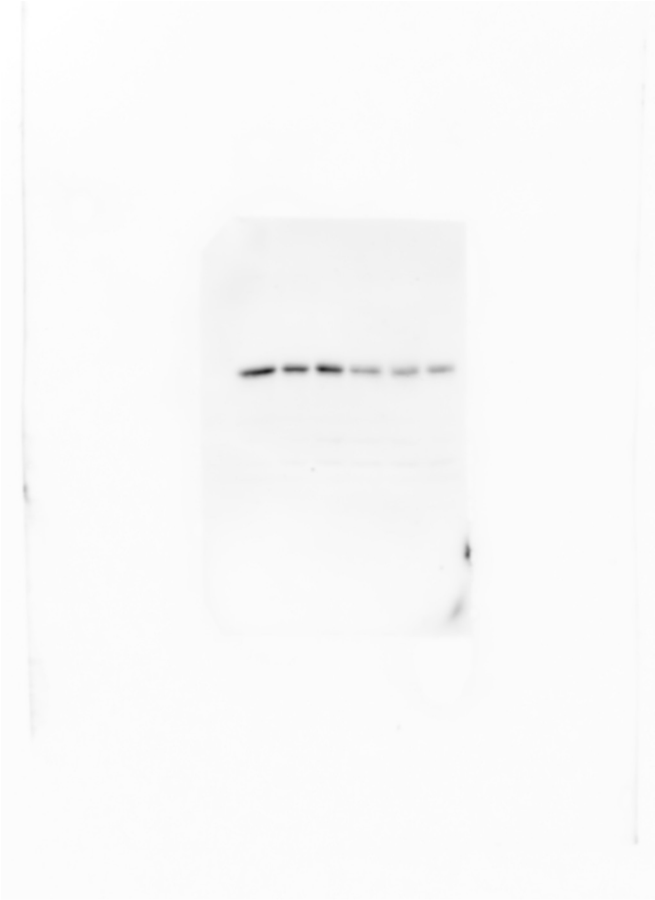

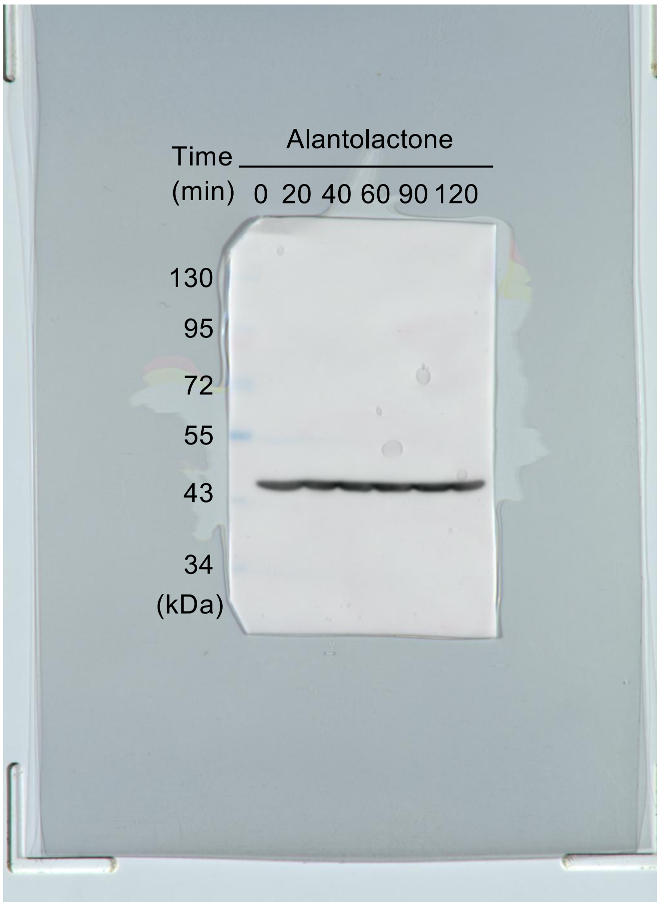

WB: β-Actin (reprobed)

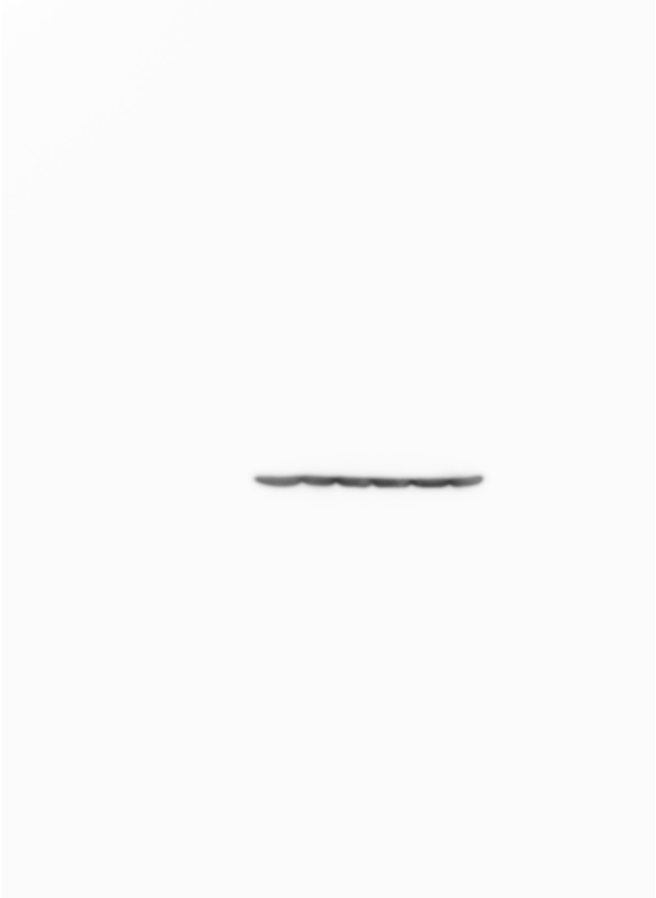

Figure S69: Original blots (1) in Figure 7B,D

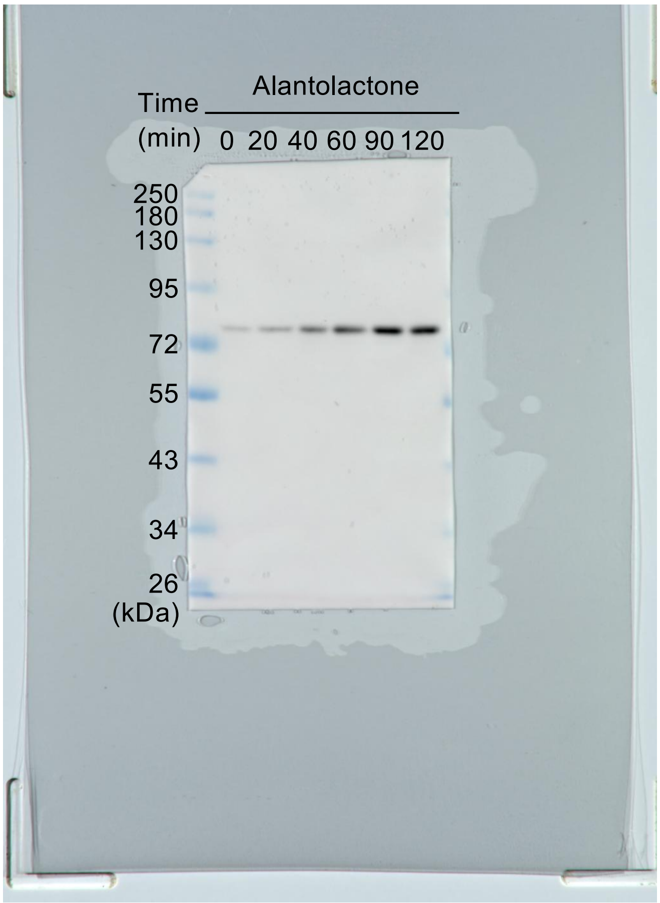

WB: Phospho-RAF1

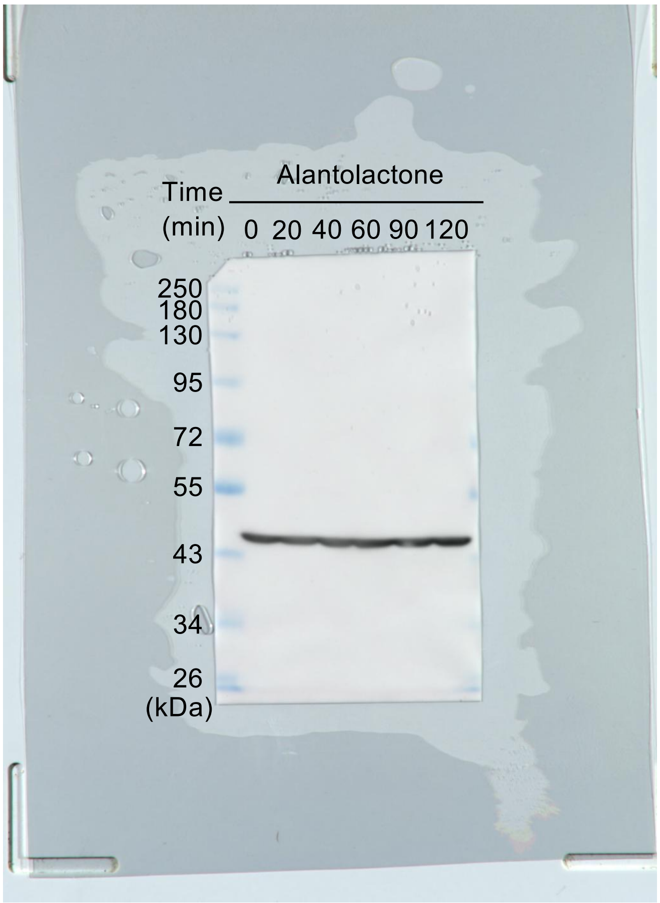

WB: β-Actin (reprobed)

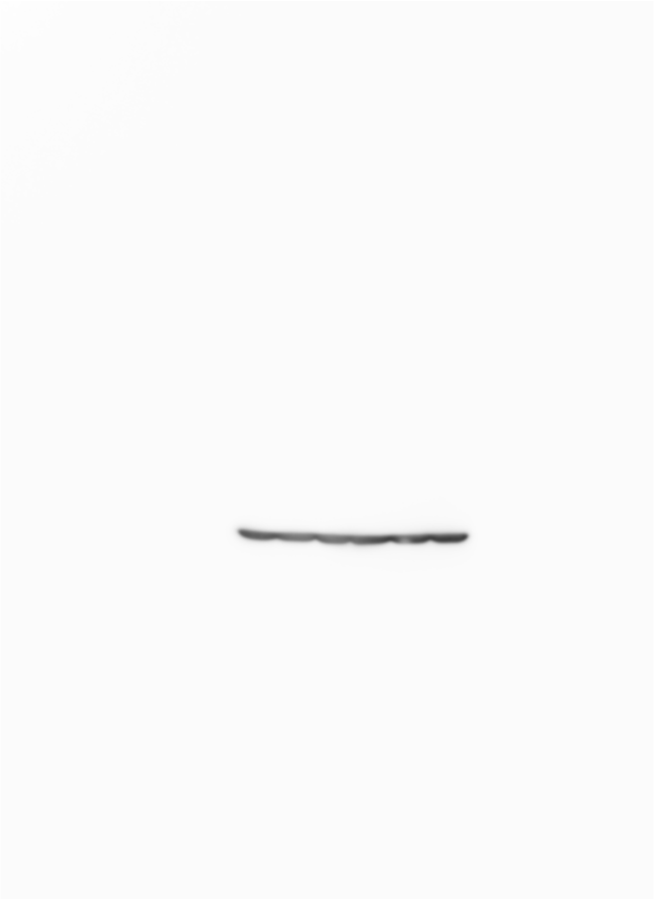

Figure S70: Original blots (2) in Figure 7B,D

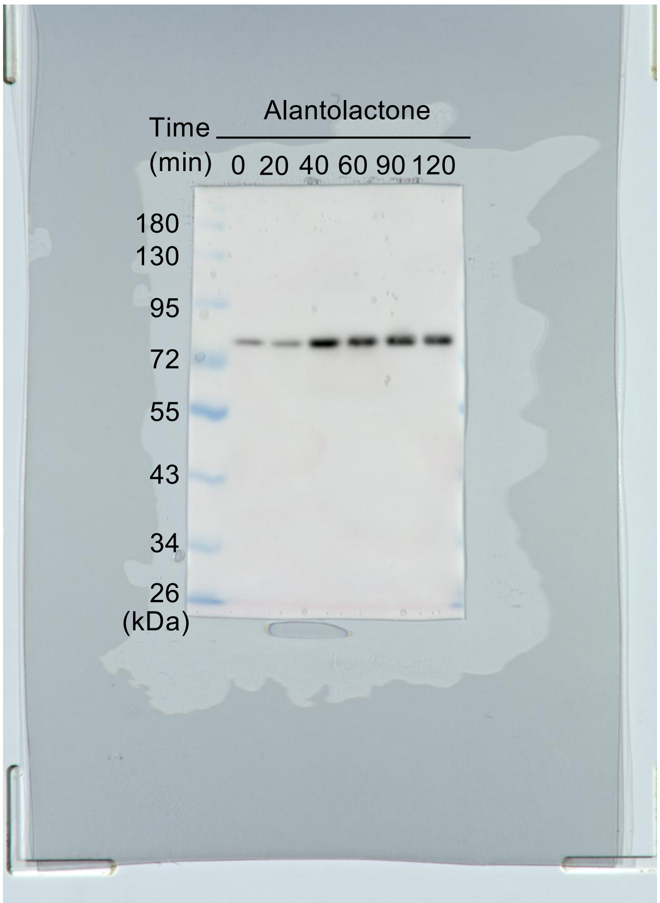

WB: Phospho-RAF1

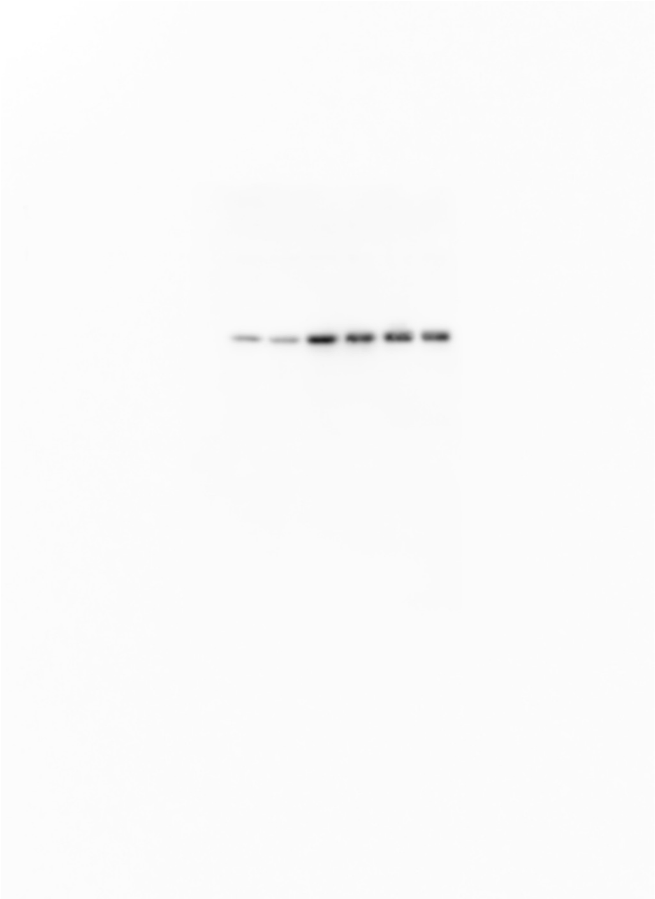

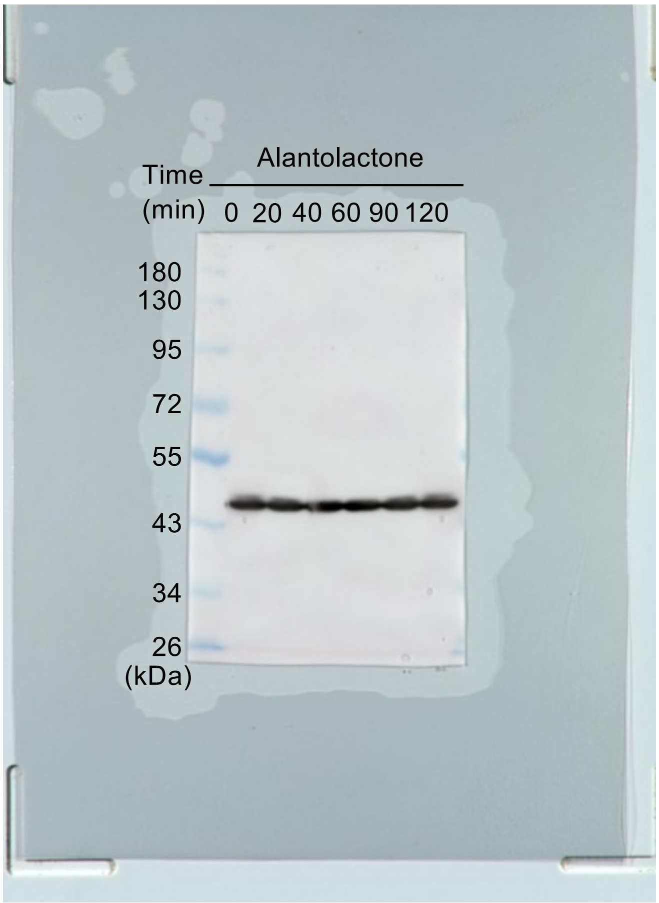

WB: β-Actin (reprobed)

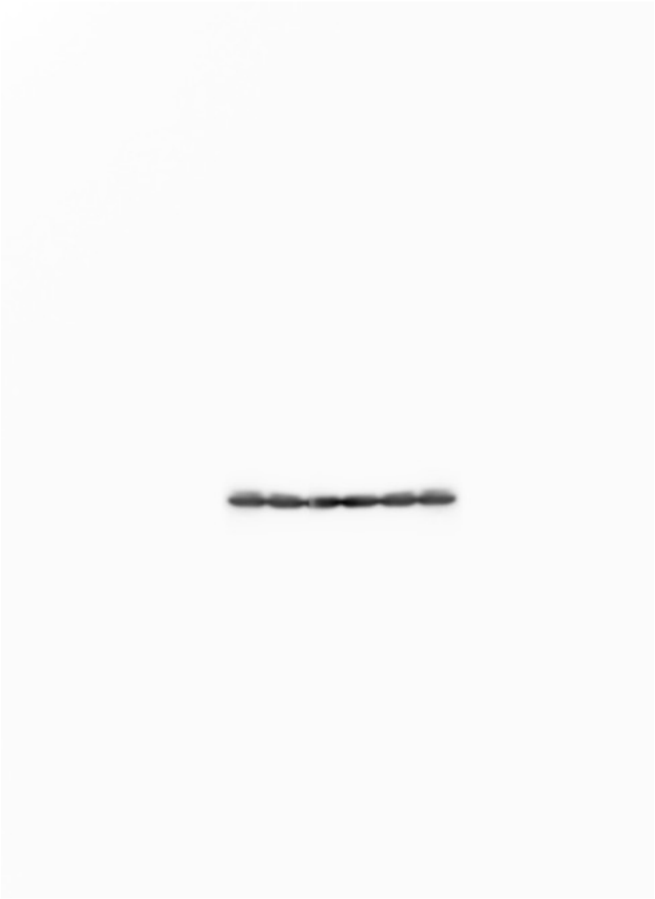

Figure S71: Original blots (3) in Figure 7B,D

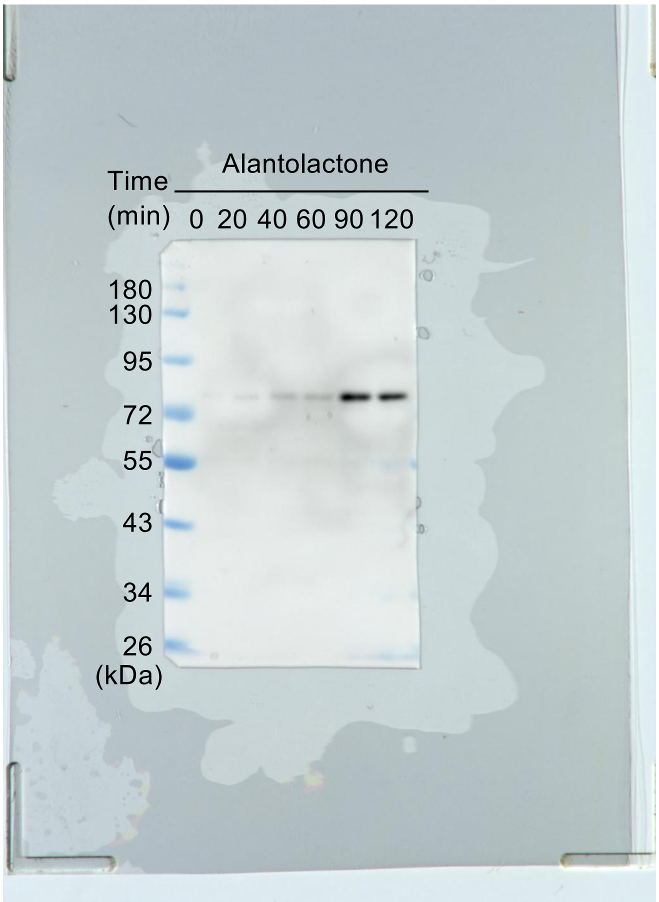

WB: Phospho-RAF1

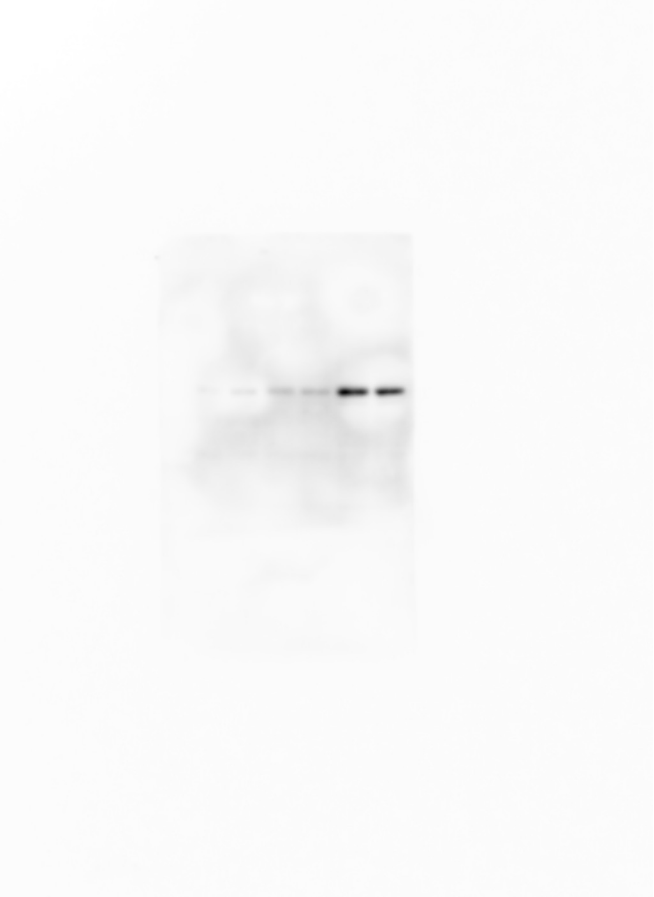

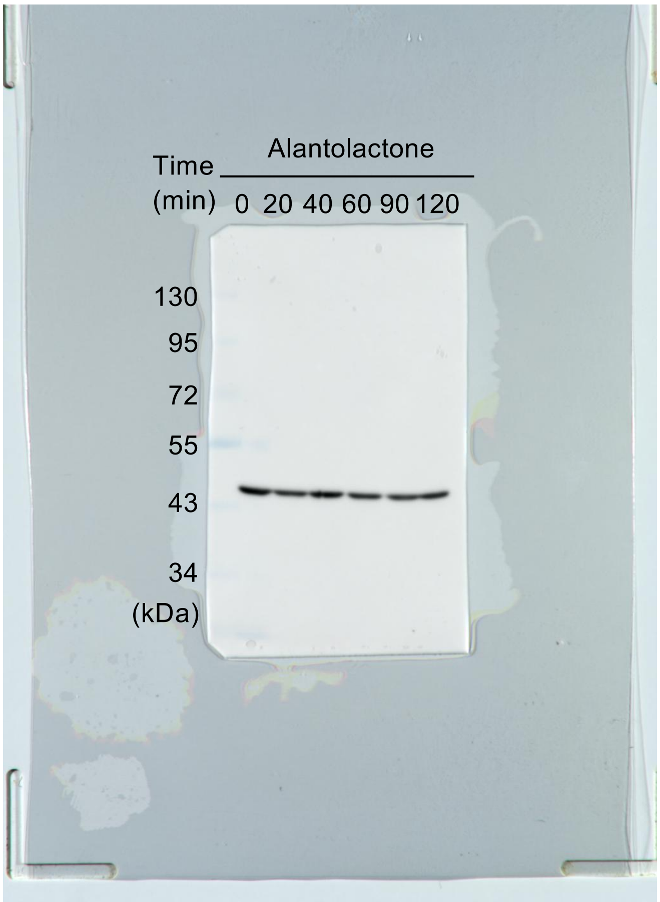

WB: β-Actin (reprobed)

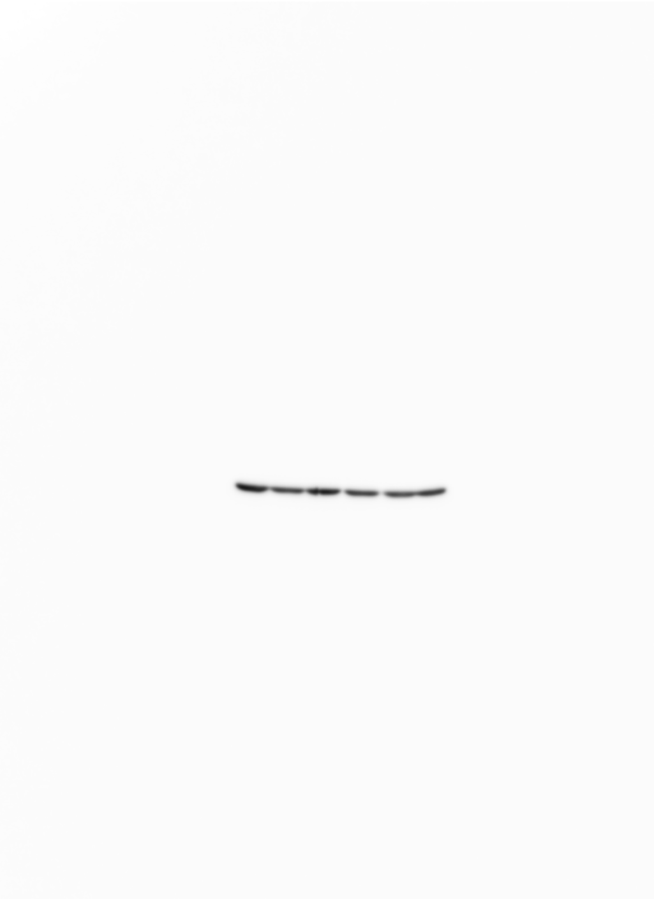

Figure S72: Original blots (1) in Figure 7C,D

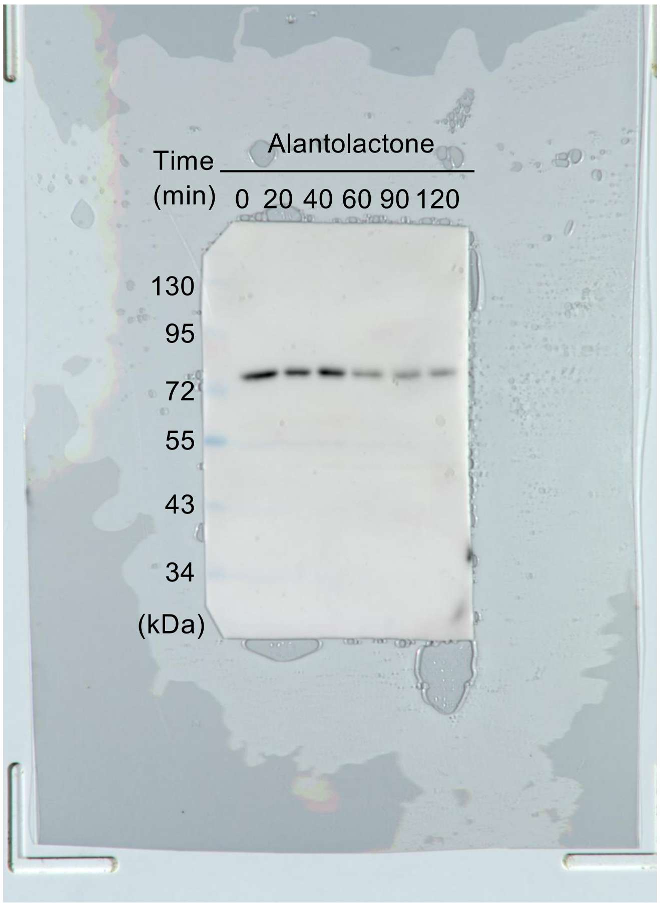

WB: RAF1

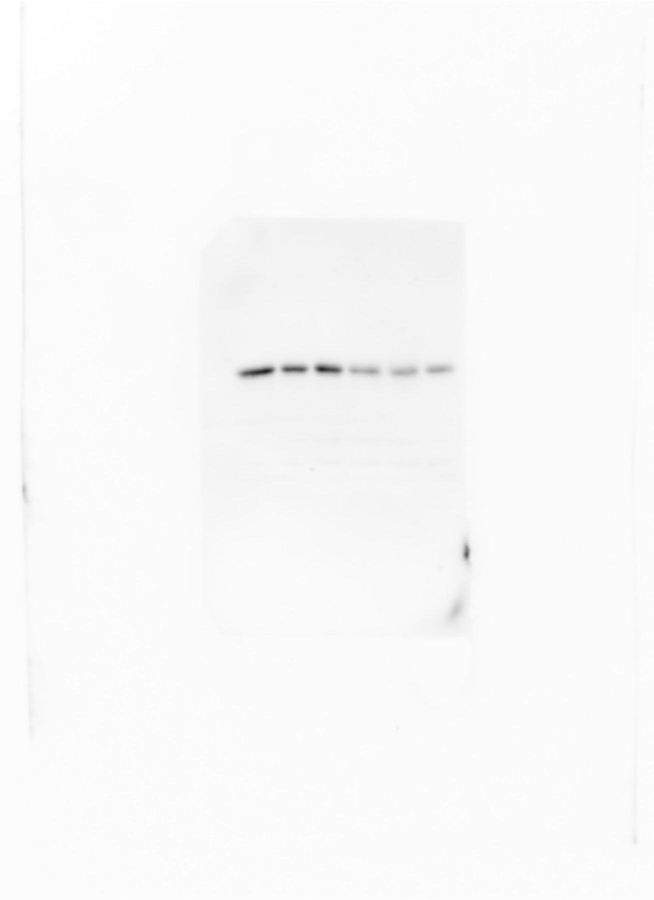

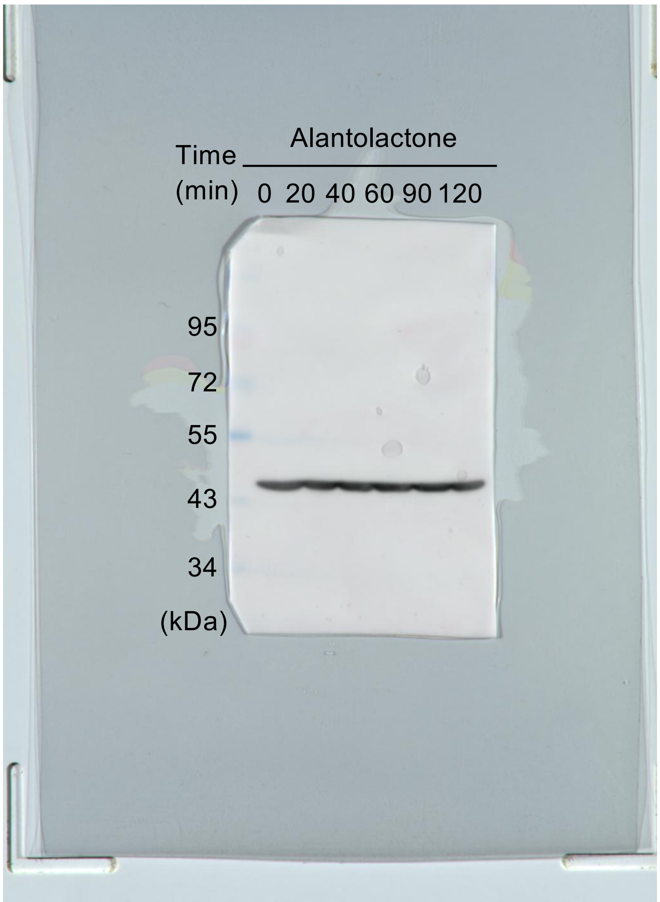

WB: β-Actin (reprobed)

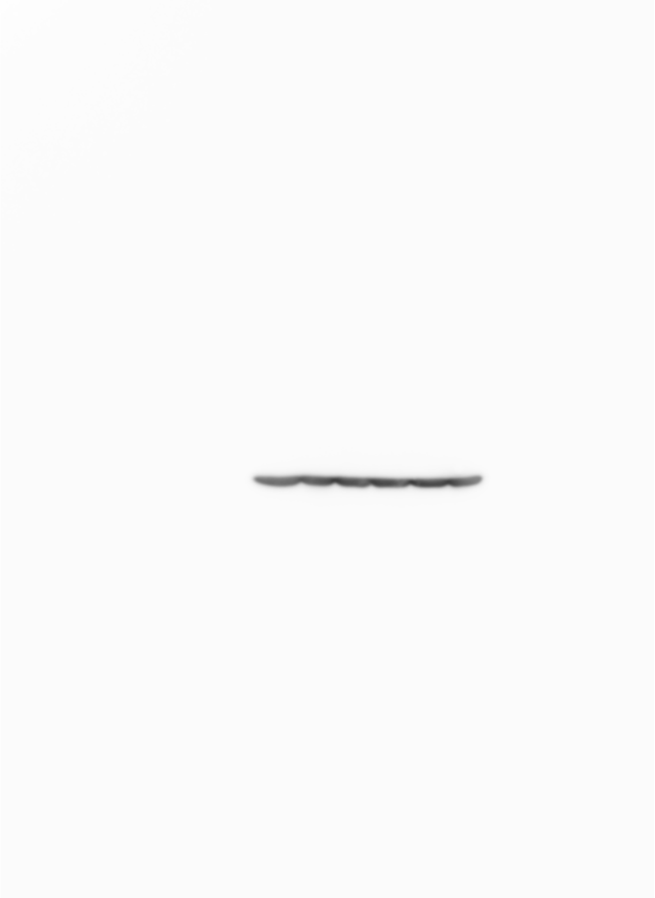

Figure S73: Original blots (2) in Figure 7C,D

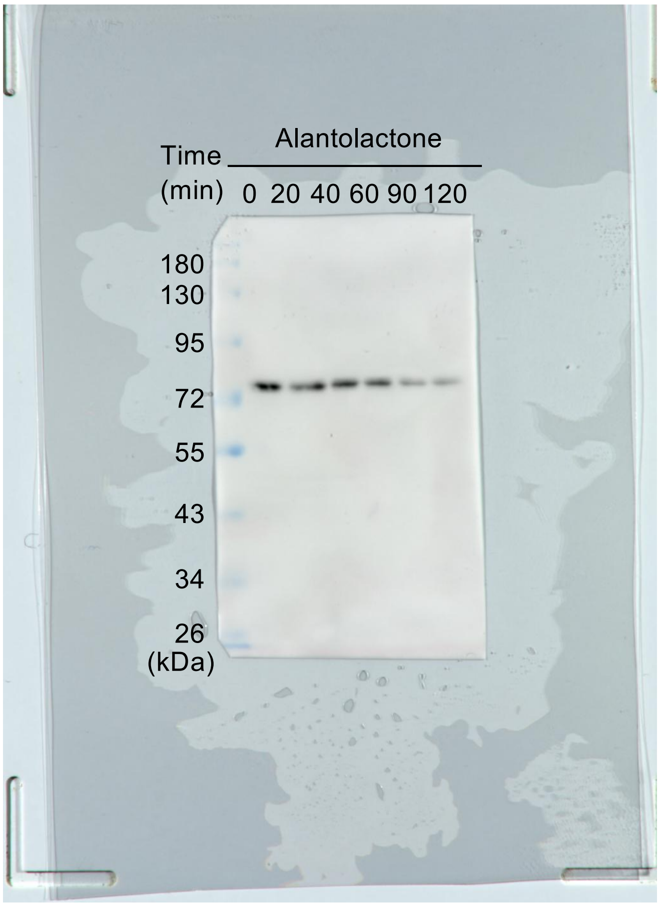

WB: RAF1

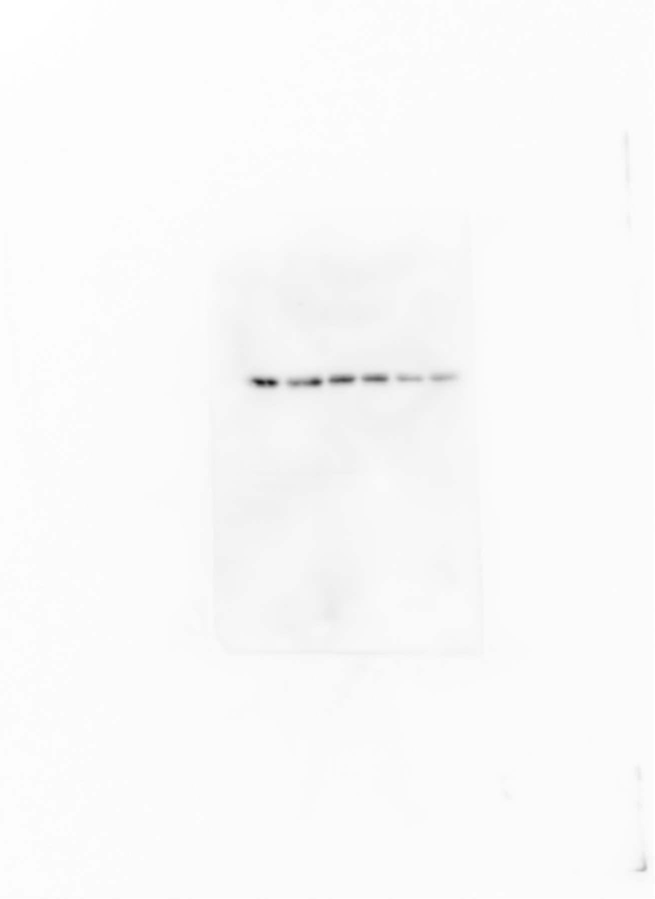

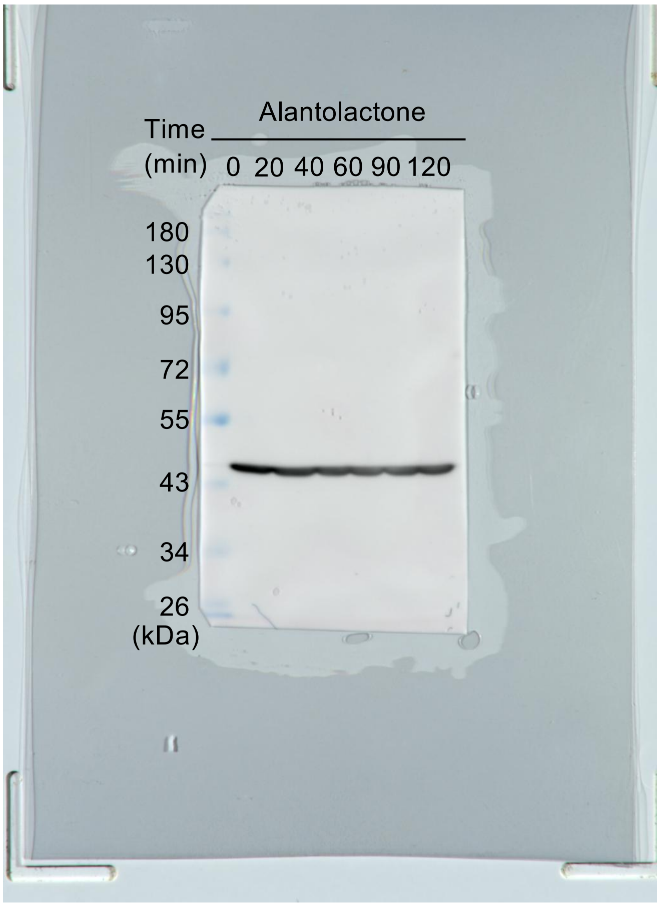

WB: β-Actin (reprobed)

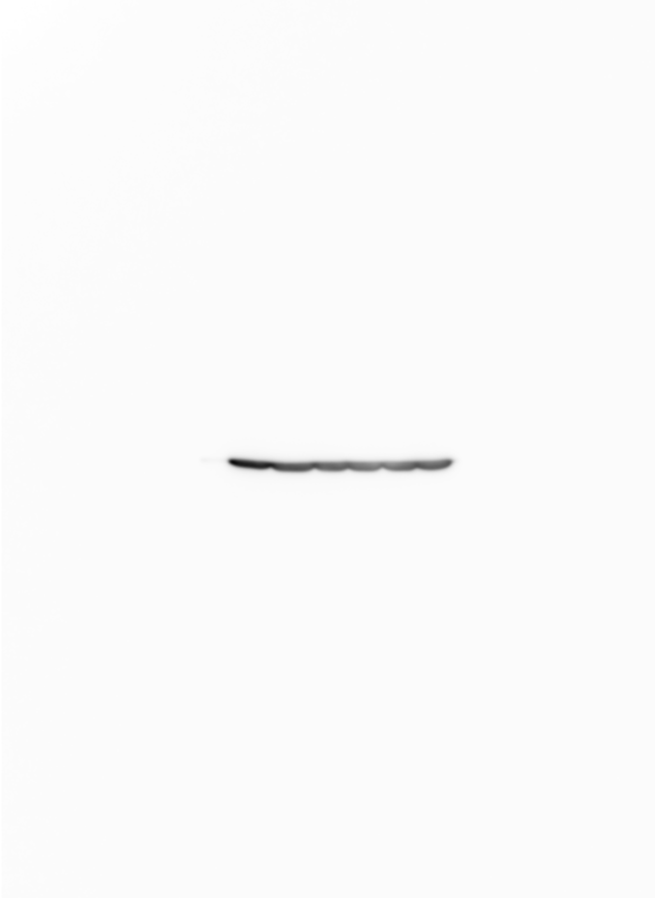

Figure S74: Original blots (3) in Figure 7C,D

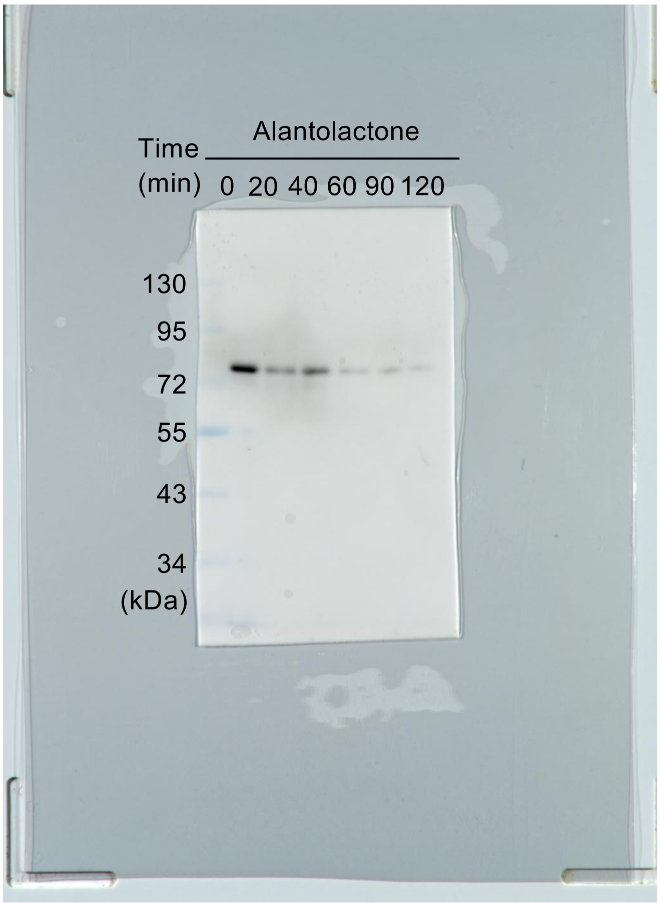

WB: RAF1

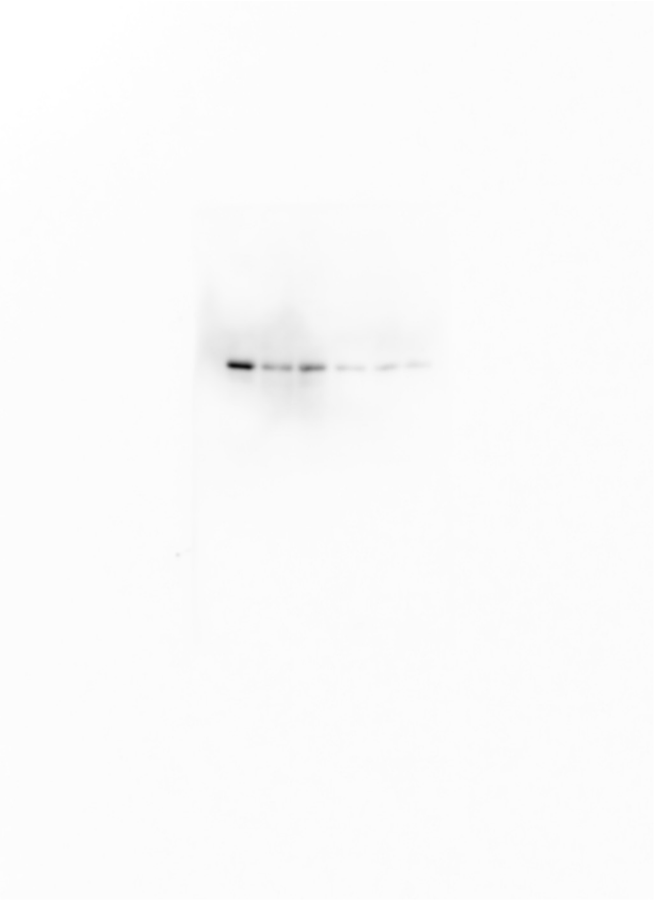

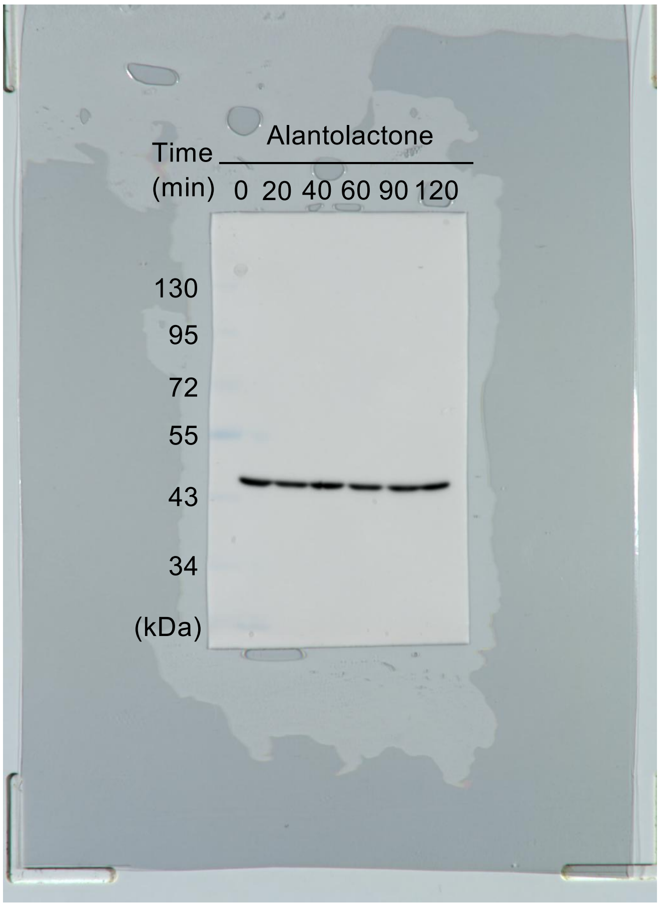

WB: β-Actin (reprobed)

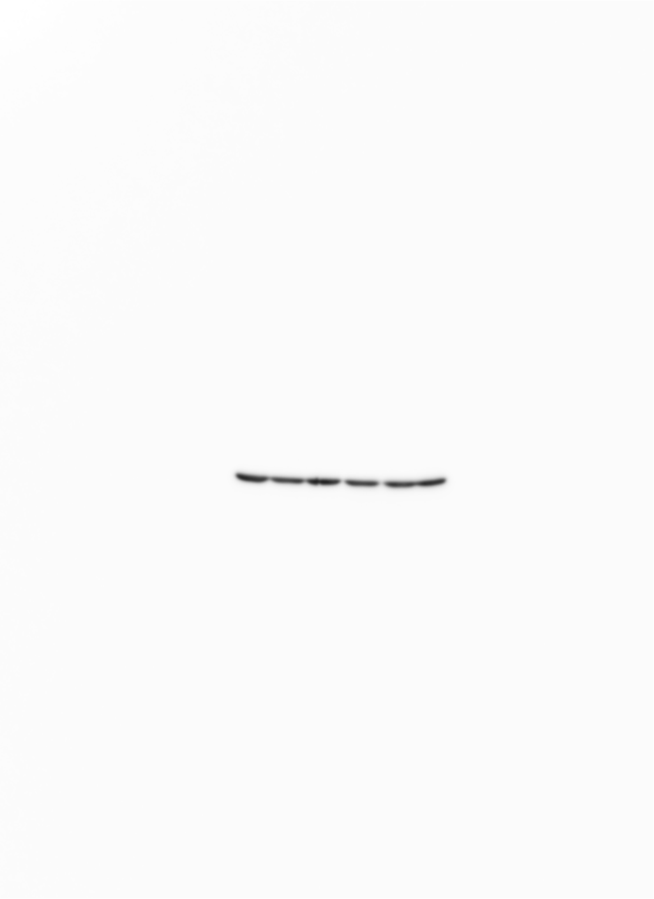

Supplement: Supplementary file 1 [file molecules-31-03296-s001.zip › molecules-4528547-supplementary.pdf]
